# Supplementary material for: ZSWIM4 regulates embryonic patterning and BMP signaling by promoting nuclear Smad1 degradation
Source: EMBO Rep. 2024 Jan 2;25(2):14. doi: 10.1038/s44319-023-00046-w (PMC10897318; doi:10.1038/s44319-023-00046-w)
Supplement: Supplementary file 1 — Appendix [file 44319_2023_46_MOESM1_ESM.pdf]

Table of Contents

APPENDIX FIGURE S1 2

APPENDIX FIGURE S2 2

APPENDIX FIGURE S3 3

APPENDIX FIGURE S4 3

APPENDIX FIGURE S5 4

APPENDIX FIGURE S6 4

APPENDIX FIGURE S7 5

APPENDIX TABLE S1 6

APPENDIX TABLE S2 6

APPENDIX TABLE S3 8

Appendix Figures and legends

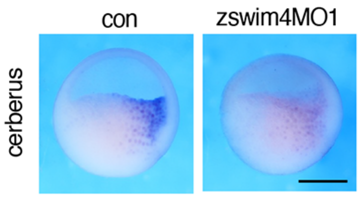

Appendix Figure S1

Knockdown of *zswim4* repressed *cerberus* expression (61.5%, 24 out of 39 injected embryos). After injected with zswim4MO1 (2ng/embryo), the *X. tropicalis* was collected at stage 10.5, and bisected for WISH. n =2, biological replicates. One representative blot was shown. Scale bar = 1000 μm.

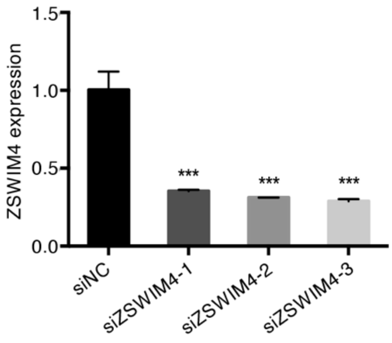

Appendix Figure S2

Quantitative PCR showing *ZSWIM4* expression in HEK293T cells transfected separately with three different *ZSWIM4* siRNAs or a control siRNA (80 nM). n =3, biological replicates. Error bars show mean ± standard deviation (SD). Statistical analysis was performed using an unpaired Student's *t*-test. ***p < 0.001.

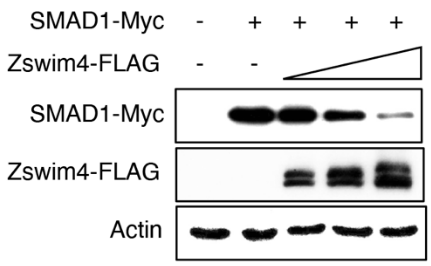

Appendix Figure S3

Overexpression of *zswim4-FLAG* reduced the exogenous SMAD1-Myc protein in a dose-dependent manner in HEK293T cells. HEK293T cells were transfected with *SMAD1-Myc* and different doses of *Zswim4-FLAG* (200, 300, and 400 ng/ml medium). After 48 hours, cells were lysed for western blot. n =2, biological replicates. One representative blot was shown.

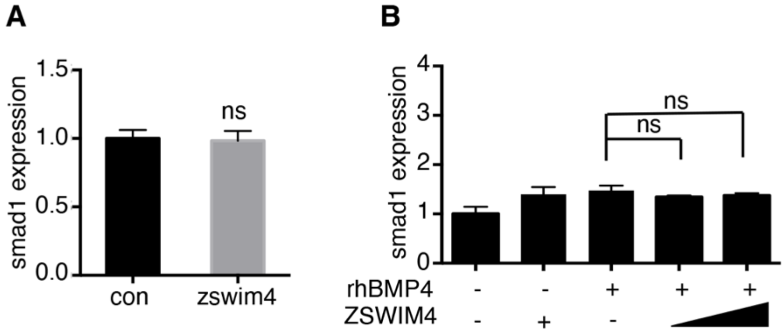

Appendix Figure S4

Quantitative PCR showing *ZSWIM4* expression in *X. laevis* embryos injected with *zswim4* mRNA (300 pg/embryo) (A) or HEK293T cells transfected with *ZSWIM4* construct (300 ng/ml medium) (B). n =3, biological replicates. Error bars show mean ± standard deviation (SD). Statistical analysis was performed using an unpaired Student's *t*-test. ns indicates "not significant" ($p > 0.05$).

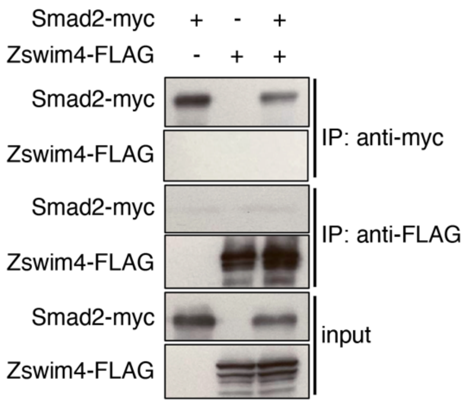

Appendix Figure S5

Co-IP assay showed that there was no interaction between SMAD2-Myc and ZSWIM4-FLAG.

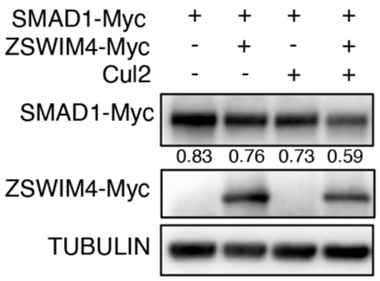

Appendix Figure S6

Co-expression of *ZSWIM4* and *Cul2* (150 ng ZSWIM4-Myc/ml medium, 150 ng *cul2*/ml medium) reduced the protein level of SMAD1-Myc in HEK293T cells. n =2, biological replicates. One representative blot was shown.

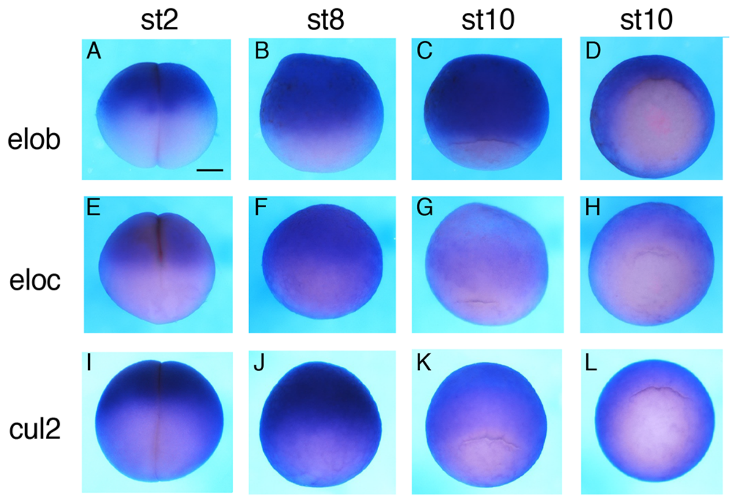

Appendix Figure S7

Expression patterns of CRL components, *elob*, *eloc* and *cul2*, in *Xenopus* embryos. A, B, E, F, I, J lateral view; C, G, K dorsal view; D, H, L vegetal view with dorsal side on the top. Scale bar = 250 µm.

Supplementary Table

Appendix Table S1

Antisense morpholino oligos used in this study

Name	Sequence (5'-->3')	Targe
zswim4MO1	GGGACGGATATAGCAGCGAACCC	*X. tropicalis* zswim4
zswim4MO2	CTCCTCGACCTGAAACAGAGGGAAC	*X. tropicalis* zswim4
zwwim4MO	GATTCACTCCTAATCCTTCCCCAGT	*X. laevis* zswim4
z7MO	CCATCTCCCCATACAGTTCACATTA	*X. tropicalis* zswim7

Appendix Table S2

Primers and oligos used in this study

Fw, forward; Re, reverse; xt, *Xenopus tropicalis*; xl, *Xenopus laevis*

Name	Sequence (5'-->3')	Purpose
xt sizzled Fw	AGAAGTCGTGCCCAAGTCAG	quantitative RT-PCR
xt sizzled Re	GCAGGGCTGGATGAACGTAT	
xt sox2 Fw	TACATGAACGGCTCGCCTAC	
xt sox2 Re	AGCCCGTGAATGGGAAGAAG	
xt otx2 Fw	GCGGATTGTGCTCCCTGATA	
xt otx2 Re	TGGGGGTCGCGGGATA	
xt xhox3 Fw	TCATCTTGAAGGTGGCGGAC	
xt xhox3 Re	GGGAACACCATTGCCCTCTT	
xt zswim1 Fw	TCAGACTCCAGCGTGAAACC	
xt zswim1 Re	TGCAGCGCATAACTCCTTCA	
xt zswim3 Fw	ACACGGCCCTTCTGAAAGAG	
xt zswim3 Re	AGCCACTCCTTGGTTGTAGC	
xt zswim5 Fw	GACTATTCGCAGCGTGAGGA	
xt zswim5 Re	TCCACCGCAATCTGATAGGC	
xt zswim6 Fw	TCGGACAGTGTTCACACGAG	
xt zswim6 Re	CCCACGAGAATCGAAGAGGG	
xt zswim7 Fw	TATTGTTCCTGCCCCGCTTT	
xt zswim7 Re	TGGGGAACAGTATGTCGCAC	
xt zswim8 Fw	TTATCGGGGCGCAATATCCC	
xt zswim8 Re	TCTCGGCATGGTATGGATGC	

xt zswim9 Fw	ACCCAAAAAGGGACTGCCAA	
xt zswim9 Re	AACCTGCAGACGTATCAGCC	
xt odc Fw	AGGCCACACTGGCAACTCA	
xt odc Re	TGCGCTCAGTTCTGGTACTTCA	
xl vent1 Fw	AGCCCATTATCCATTTCTGGTT	
xl vent1 Re	TGCTGAAGGGAATCCTGCTC	
xl sizzled Fw	ACACGTACCATGATTGAACA	
xl sizzled Re	CATACAGTGAAGTACAAGAACTG	
xl zswim4 Fw	CCCCATGGCTCCGATATTCT	
xl zswim4 Re	CGTCTTGGGCCAACTTGAAA	
xl odc Fw	TGCACATGTCAAGCCAGTTC	
xl odc Re	CTACGATACGATCCAGCCCA	
Human *ID1* Fw	CTGCTCTACGACATGAACGGC	
Human *ID1* Re	TGACGTGCTGGAGAATCTCCA	
Human *ID2* Fw	GACCCGATGAGCCTGCTATAC	
Human *ID2* Re	CGTGCTGCAGGATTTCCATCT	
Human *ZSWIM4* Fw	GTCGTTTCCACGCAGTGAAC	
Human *ZSWIM4* Re	GATGTTTCCGCTCAGGTGGA	
Human *SMAD1* Fw	ATTCGTGAGTTCGCGGTTGA	
Human *SMAD1* Re	CCAAATGCAAAAGGACAGCAGA	
Human b-actin Fw	AGGATGCAGAAGGAGATCACTG	
Human b-actin Re	GGGTGTAACGCAACTAAGTCATAG	
xl nr3 Fw	ACATCGTTTCTTGGATTCCT	RT-PCR
xl nr3 Re	AACTGGAAAACAGAGCCAAT	
xl sox2 Fw	GAGGATGGACACTTATGCCCAC	
xl sox2 Re	GGACATGCTGTAGGTAGGCGA	
xl sox3 Fw	TGATGCAGGACCAGTTGGGC	
xl sox3 Re	TGAAGTGAAGGGTCGCTGGC	
xl xbra Fw	GCTGGAAGTATGTGAATGGAG	
xl xbra Re	TTAAGTGCTGTAATCTCTTCA	
xl wnt8 Fw	TATCTGGAAGTTGCAGCATACA	
xl wnt8 Re	GCAGGCACTCTCGTCCCTCTGT	
xl xhox3 Fw	ATATGATGAGCCACGCAGCAG	
xl xhox3 Re	CAGATGCTGCAGCTCTTTGGC	
xl otx2 Fw	GGATGGATTTGTTACATCCGTC	
xl otx2 Re	CACTCTCCGAGCTCACTTCCC	
xl epiker Fw	TGGCAGAAGCAGCAAGTTCC	
xl epiker Re	CTTCCAGGTCGTTATTGGCG	
xl msx1 Fw	TGTATGGATCGCACTCCCCT	
xl msx1 Re	CGGCTTCACACCAGTTTGTT	
xl odc Fw	CAGCTAGCTGTGGTGTGG	
xl odc Re	CAACATGGAAACTCACACC	
xt zswim4 ts1 Fw	TAGGGCACCTTCTCTTGGGGGC	CRISPR
xt zswim4 ts1 Re	AAACGCCCCCAAGAGAAGGTGC	

xt zswim4 ts2 Fw	TAGGTACCTGGGGCTGAGGCCC	
xt zswim4 ts2 Re	AAACGGGCCTCAGCCCCAGGTA	
xt zswim4 ts3 Fw	TAGGTGCCCGGGCCTCAGCCCC	
xt zswim4 ts3 Re	AAACGGGGCTGAGGCCCGGGCA	
xt zswim4 ts4 Fw	TAGGATCTCAGCGCCAAGAAGG	
xt zswim4 ts4 Re	AAACCCTTCTTGGCGCTGAGAT	
xt zswim4 ts5 Fw	TAGGCCCCCAAGAGAAGGTGCC	
xt zswim4 ts5 Re	AAACGGCACCTTCTCTTGGGGG	
human *ZSWIM4* ts Fw	CACCGGGGCCGGATGGAACCCCCCG	
human *ZSWIM4* ts Re	AAACCGGGGGGTTCCATCCGGCCCC	
xt zswim4 Fw	GTACGTGGGATTGTGGCTG	Genotyping T7E1 assay
xt zswim4 Re	GGAGTTGTAGTCCCACAAGAG	
human *ZSWIM4* Fw	AGAGTCTTAAAGGGGCCGCA	
human *ZSWIM4* Re	GGTGTTTCCAGGCAGGGTTA	

Appendix Table S3

List of antibodies used in this study

Antibody	Host species	Source
FLAG	mouse	Sigma, #F1804
FLAG	rabbit	Cell Signaling Technology, #1479S
Myc	mouse	Cell Signaling Technology, #2276S
Myc	rabbit	Cell Signaling Technology, #2272S
HA	mouse	Sigma, #H3663
β tubulin	rabbit	Abcam, #ab6064
GAPDH	mouse	Santa Cruz, #sc47724
Zswim4	rabbit	St John's Laboratory, #STJ195218
Smad1	rabbit	Invitrogen, #385400
phosphorylated Smad1/5/8	rabbit	Cell Signaling Technology, #13820S
anti-mouse-HRP	sheep	GE Healthcare, #NA931
anti-rabbit-HRP	goat	Abcam, #ab6721
anti-mouse-AF568	goat	Invitrogen, #A11004
anti-rabbit-AF488	goat	Invitrogen, #A11034
