# Supplementary figures and images for: ZSWIM4 regulates embryonic patterning and BMP signaling by promoting nuclear Smad1 degradation (part 1 of 2)
Source: EMBO Rep. 2024 Jan 2;25(2):14. doi: 10.1038/s44319-023-00046-w (PMC10897318; doi:10.1038/s44319-023-00046-w)

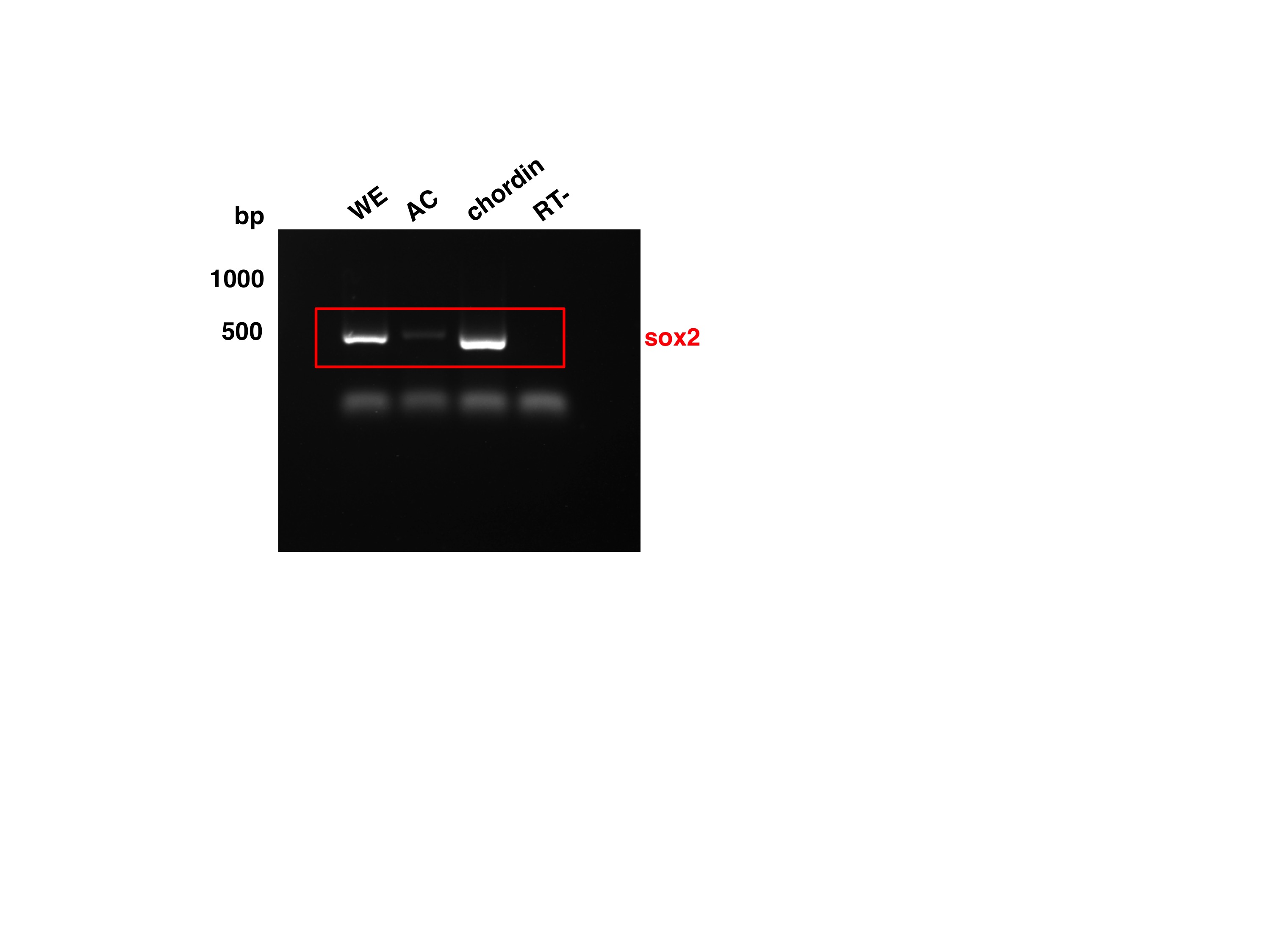

Supplement: Supplementary file 2 — Source Data Fig. 1 [file 44319_2023_46_MOESM2_ESM.zip › Figure 1/1H/DNA gel 1H sox2.jpg]

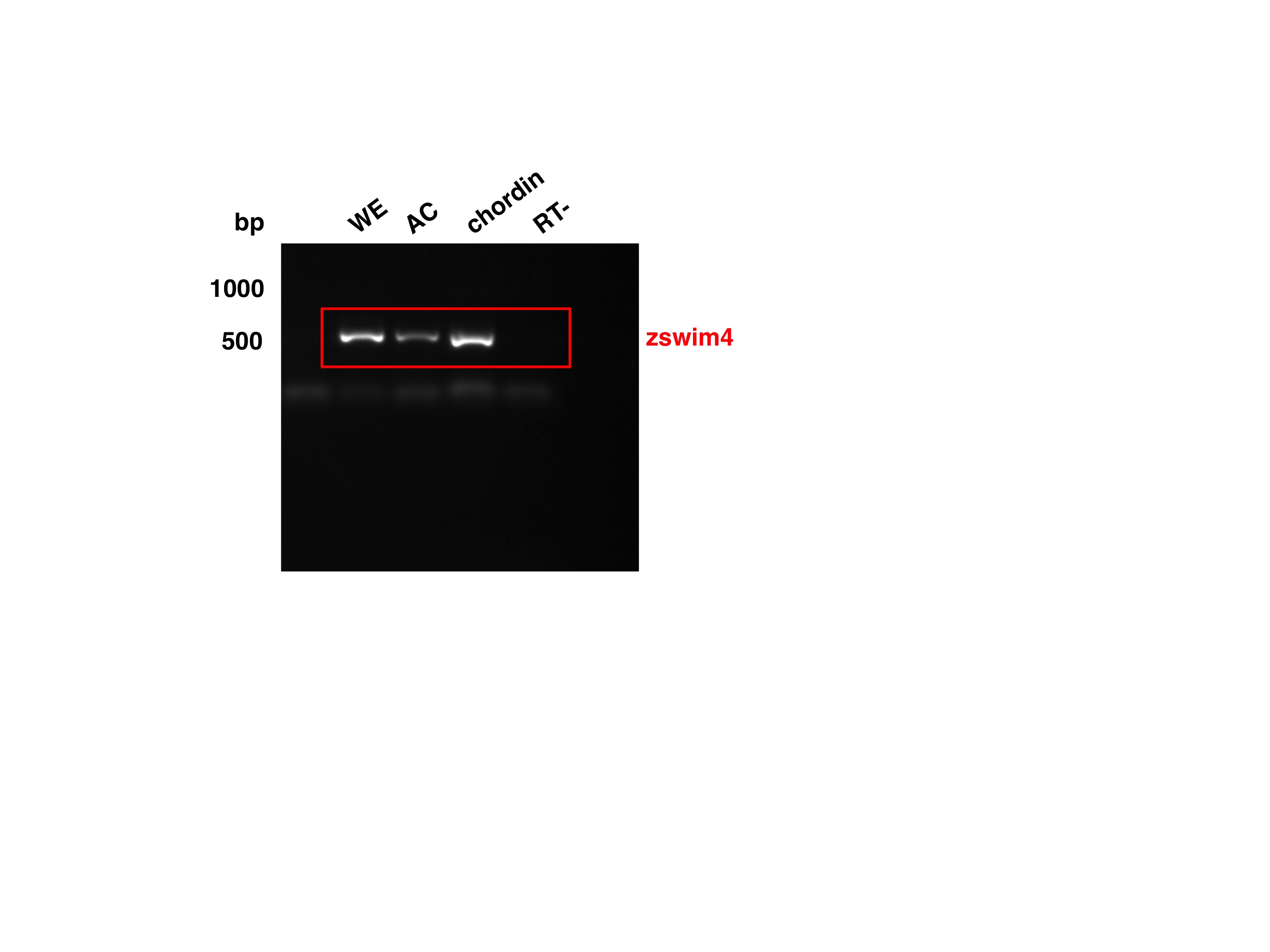

Supplement: Supplementary file 2 — Source Data Fig. 1 [file 44319_2023_46_MOESM2_ESM.zip › Figure 1/1H/DNA gel 1H zswim4.jpg]

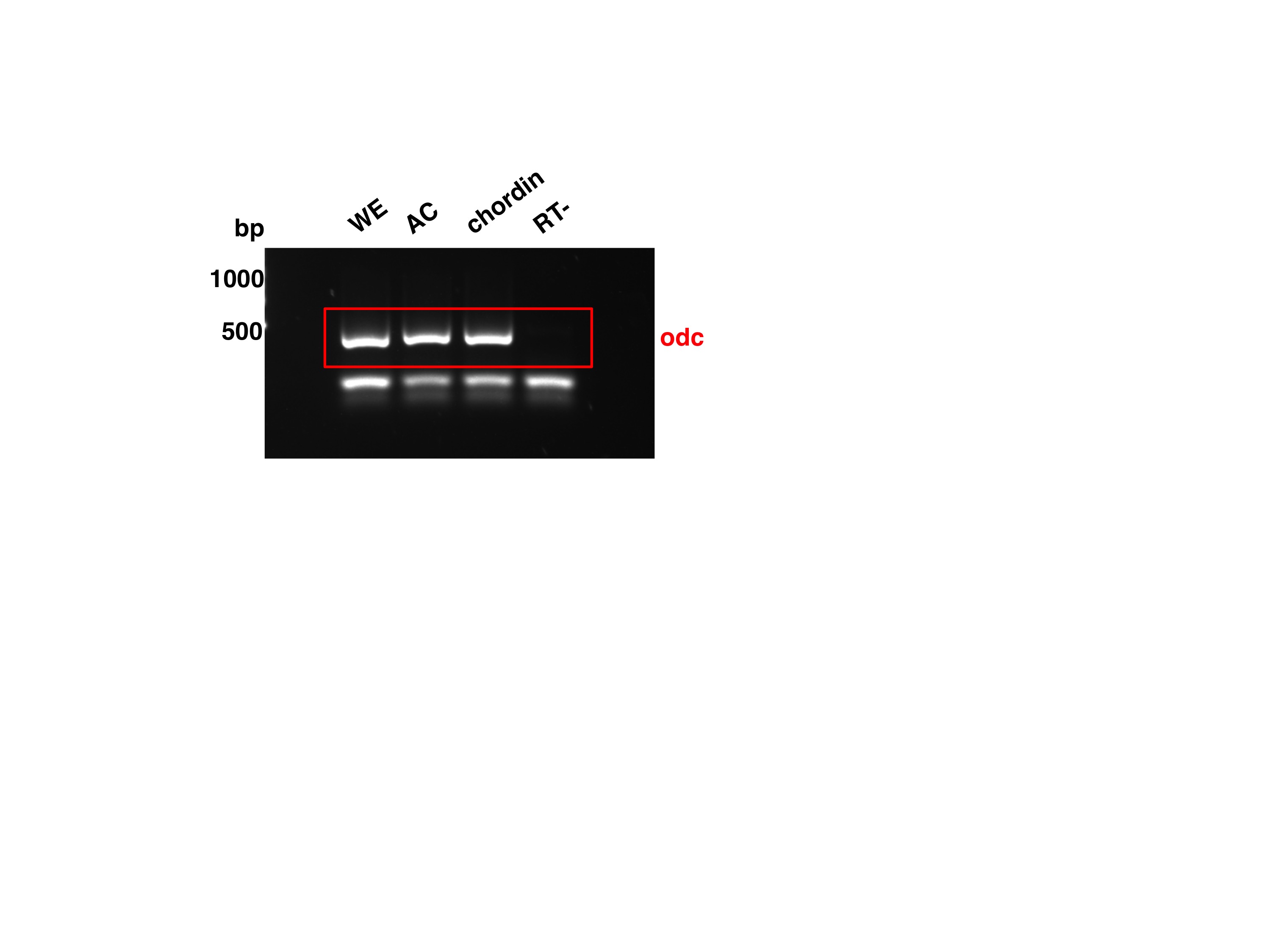

Supplement: Supplementary file 2 — Source Data Fig. 1 [file 44319_2023_46_MOESM2_ESM.zip › Figure 1/1H/DNA gel 1H odc.jpg]

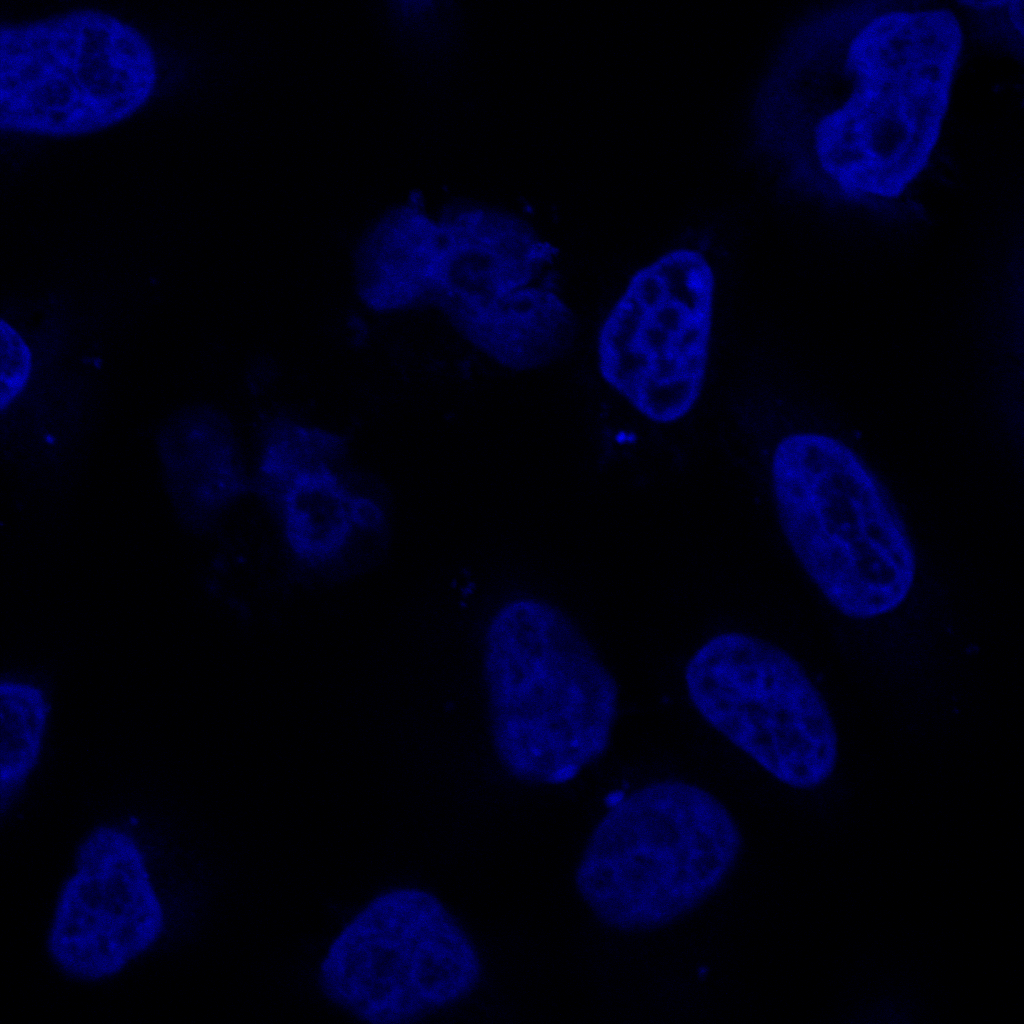

Supplement: Supplementary file 2 — Source Data Fig. 1 [file 44319_2023_46_MOESM2_ESM.zip › Figure 1/1F/image 1F DAPI.tif]

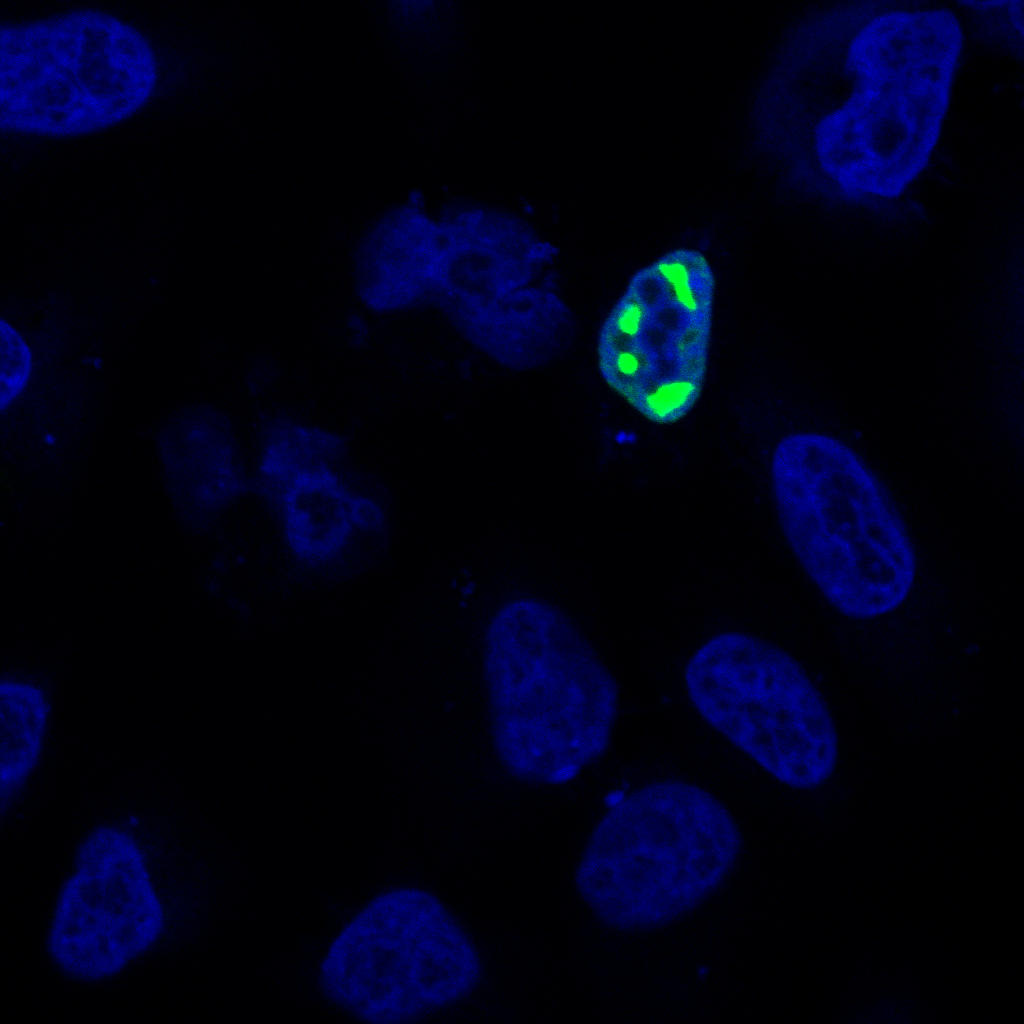

Supplement: Supplementary file 2 — Source Data Fig. 1 [file 44319_2023_46_MOESM2_ESM.zip › Figure 1/1F/image 1F merge.tif]

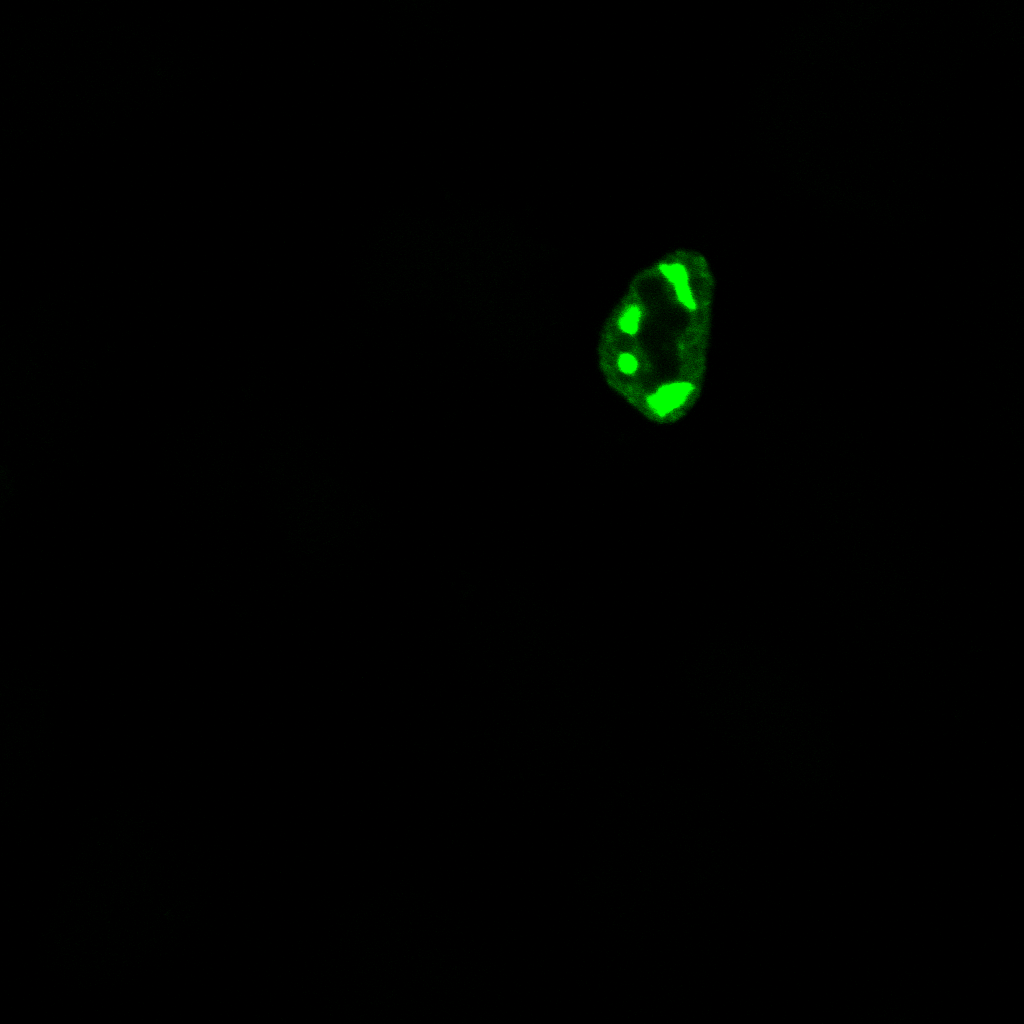

Supplement: Supplementary file 2 — Source Data Fig. 1 [file 44319_2023_46_MOESM2_ESM.zip › Figure 1/1F/image 1F ZSWIM4-FLAG.tif]

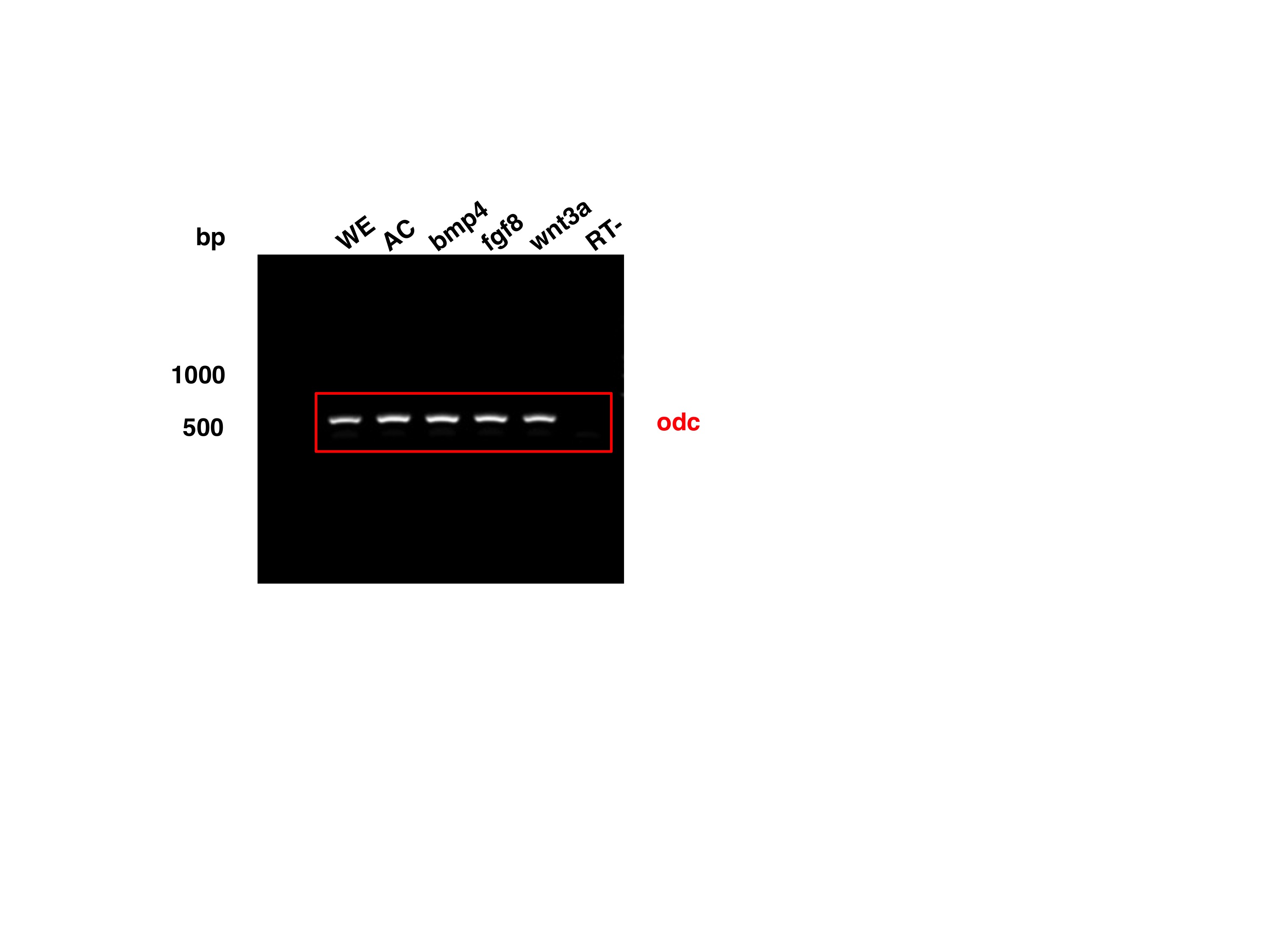

Supplement: Supplementary file 2 — Source Data Fig. 1 [file 44319_2023_46_MOESM2_ESM.zip › Figure 1/1G/DNA gel 1G odc.jpg]

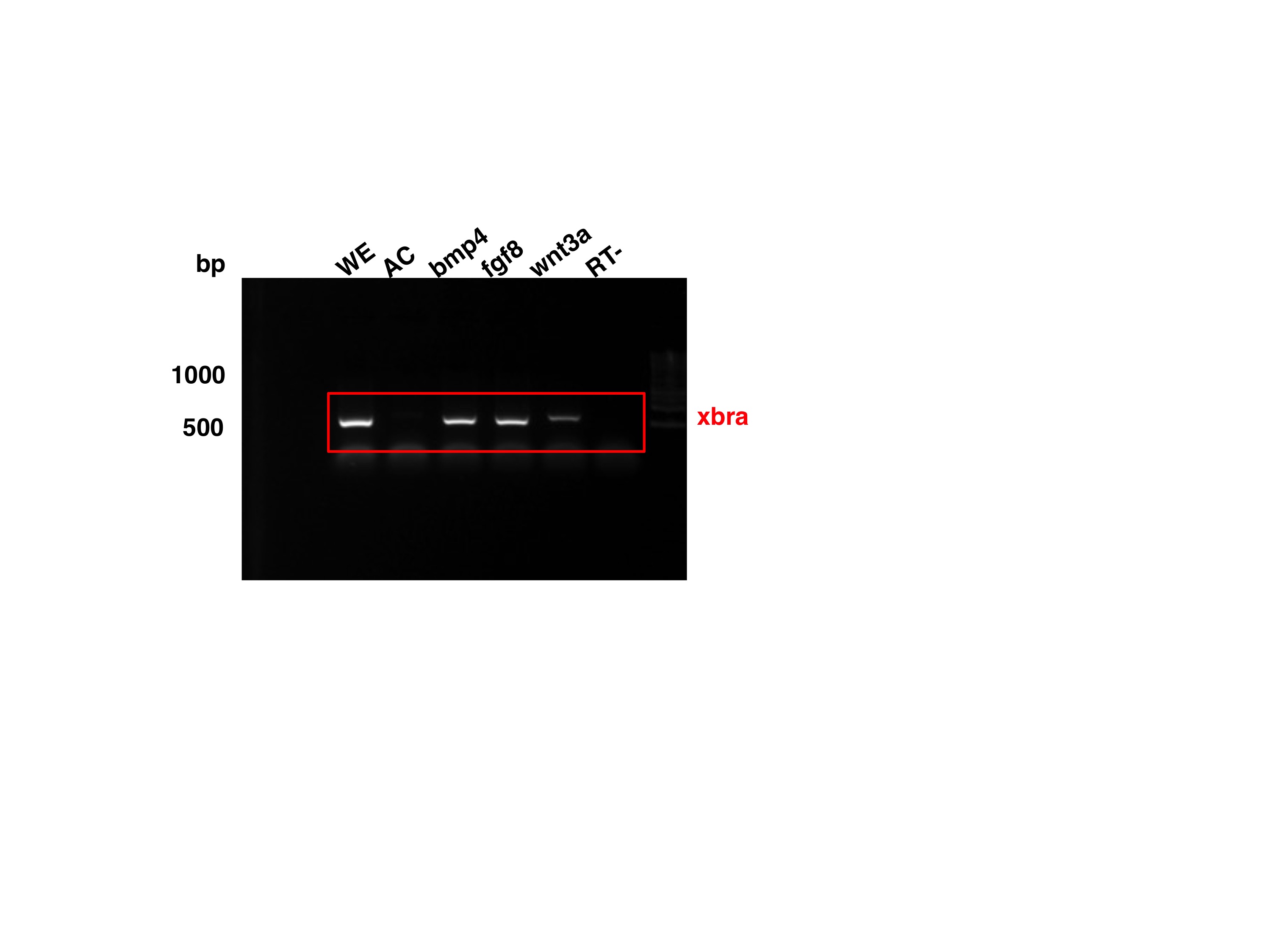

Supplement: Supplementary file 2 — Source Data Fig. 1 [file 44319_2023_46_MOESM2_ESM.zip › Figure 1/1G/DNA gel 1G xbra.jpg]

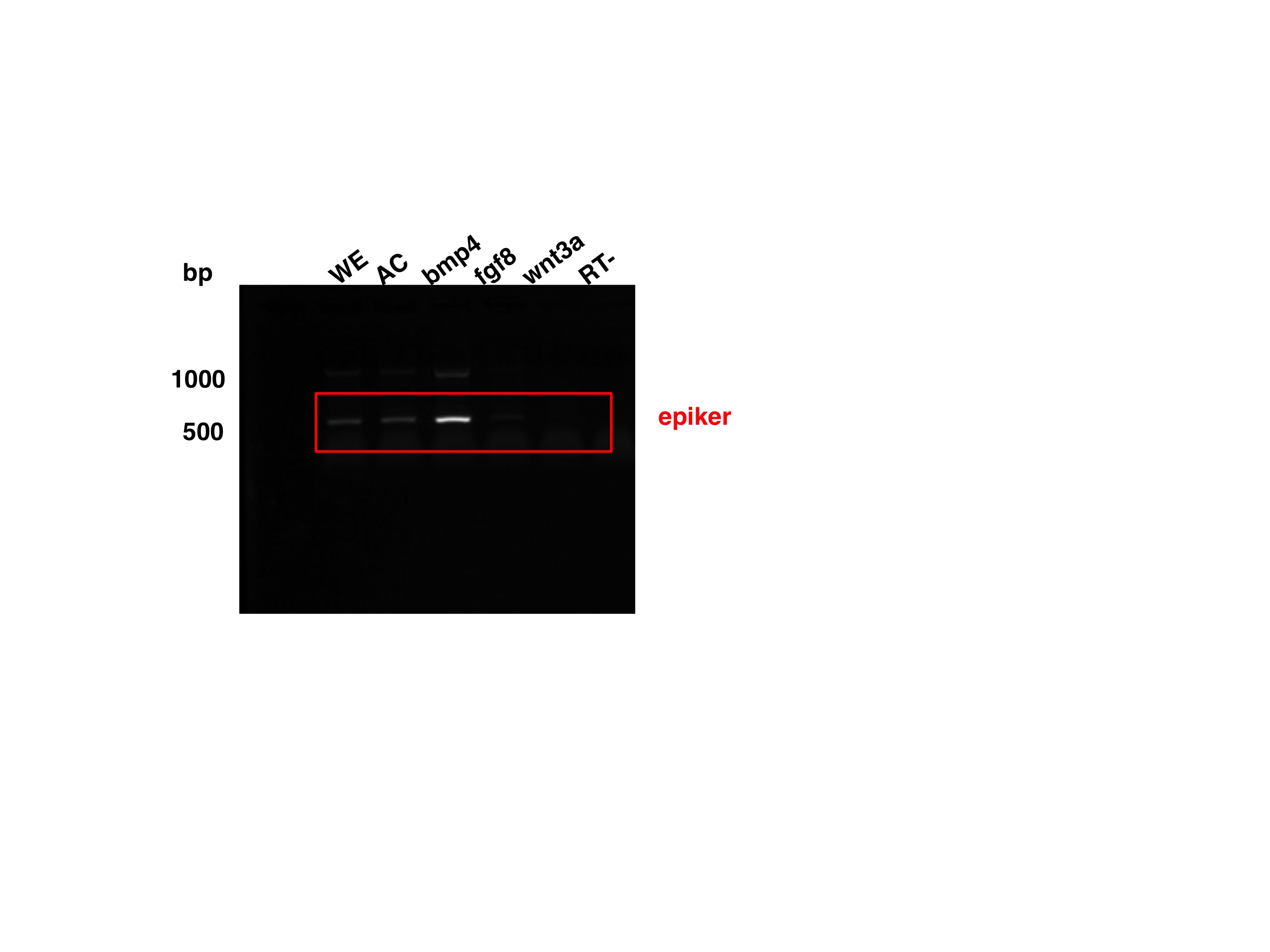

Supplement: Supplementary file 2 — Source Data Fig. 1 [file 44319_2023_46_MOESM2_ESM.zip › Figure 1/1G/DNA gel 1G epiker.jpg]

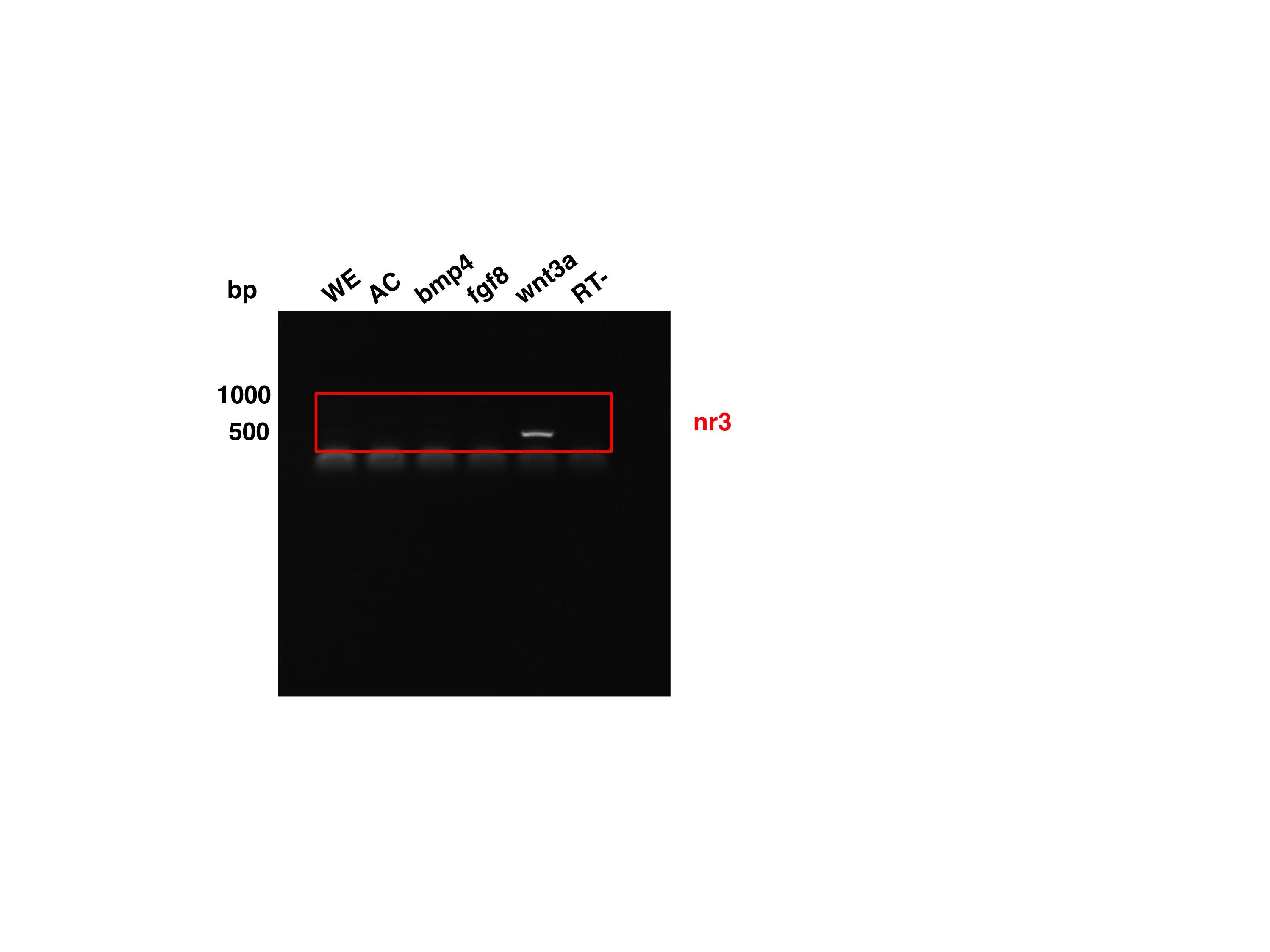

Supplement: Supplementary file 2 — Source Data Fig. 1 [file 44319_2023_46_MOESM2_ESM.zip › Figure 1/1G/DNA gel 1G nr3.jpg]

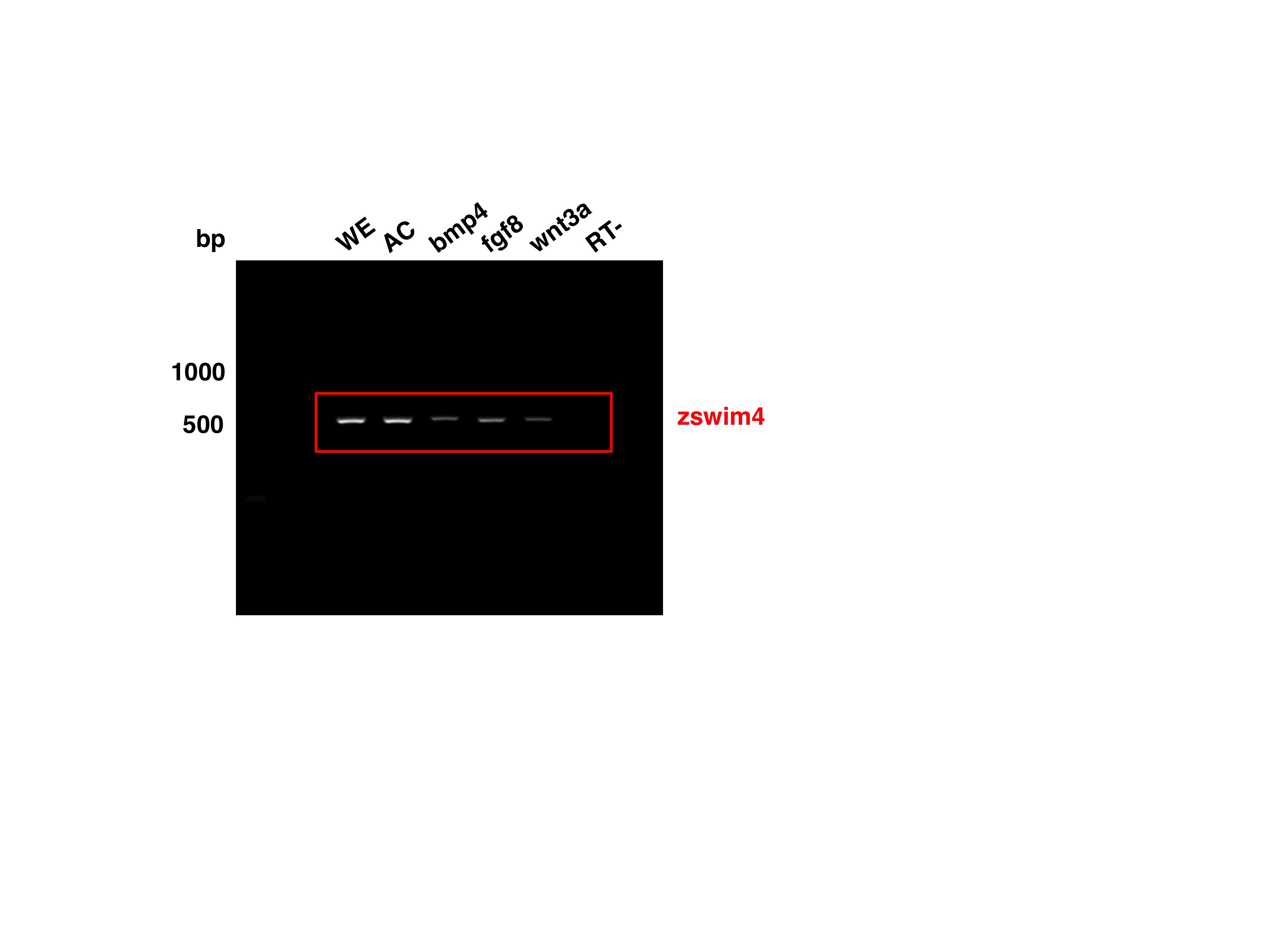

Supplement: Supplementary file 2 — Source Data Fig. 1 [file 44319_2023_46_MOESM2_ESM.zip › Figure 1/1G/DNA gel 1G zswim4.jpg]

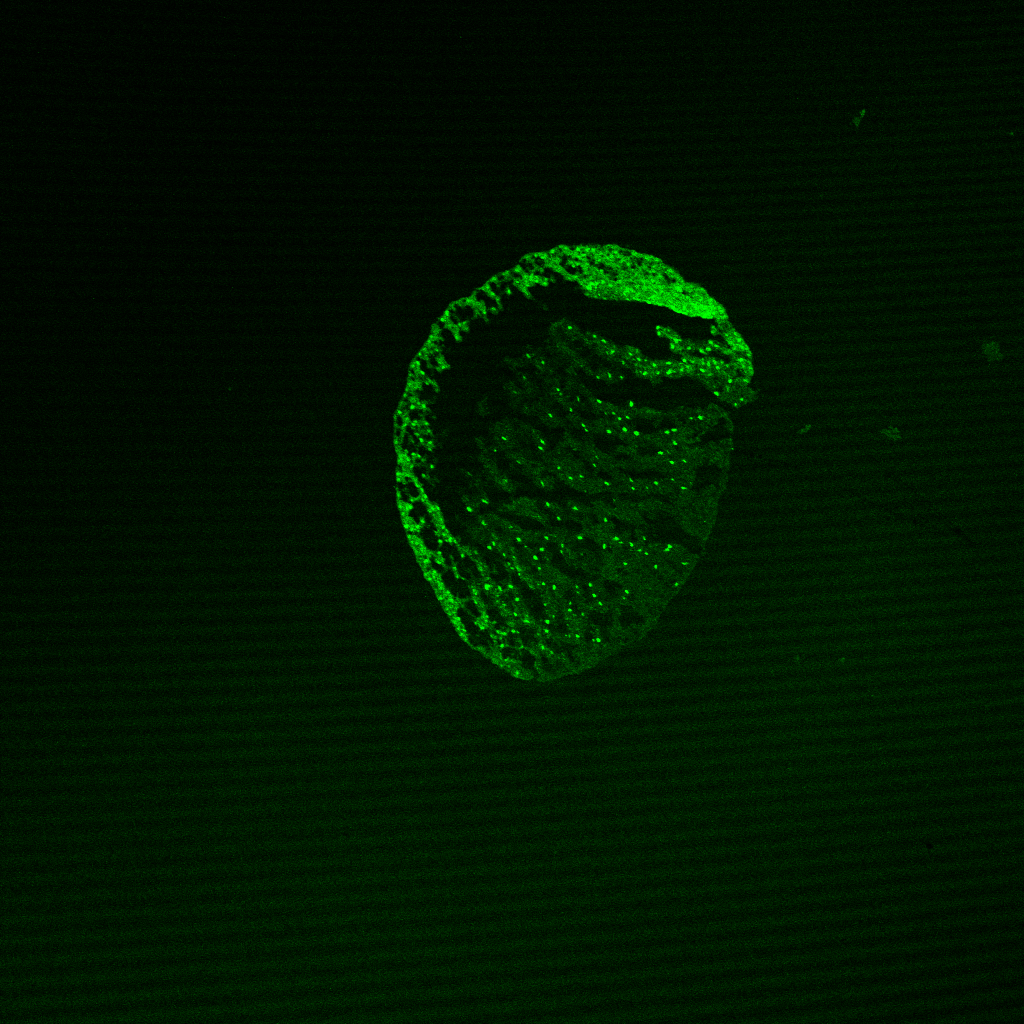

Supplement: Supplementary file 2 — Source Data Fig. 1 [file 44319_2023_46_MOESM2_ESM.zip › Figure 1/1E/image 1E Zswim4.tif]

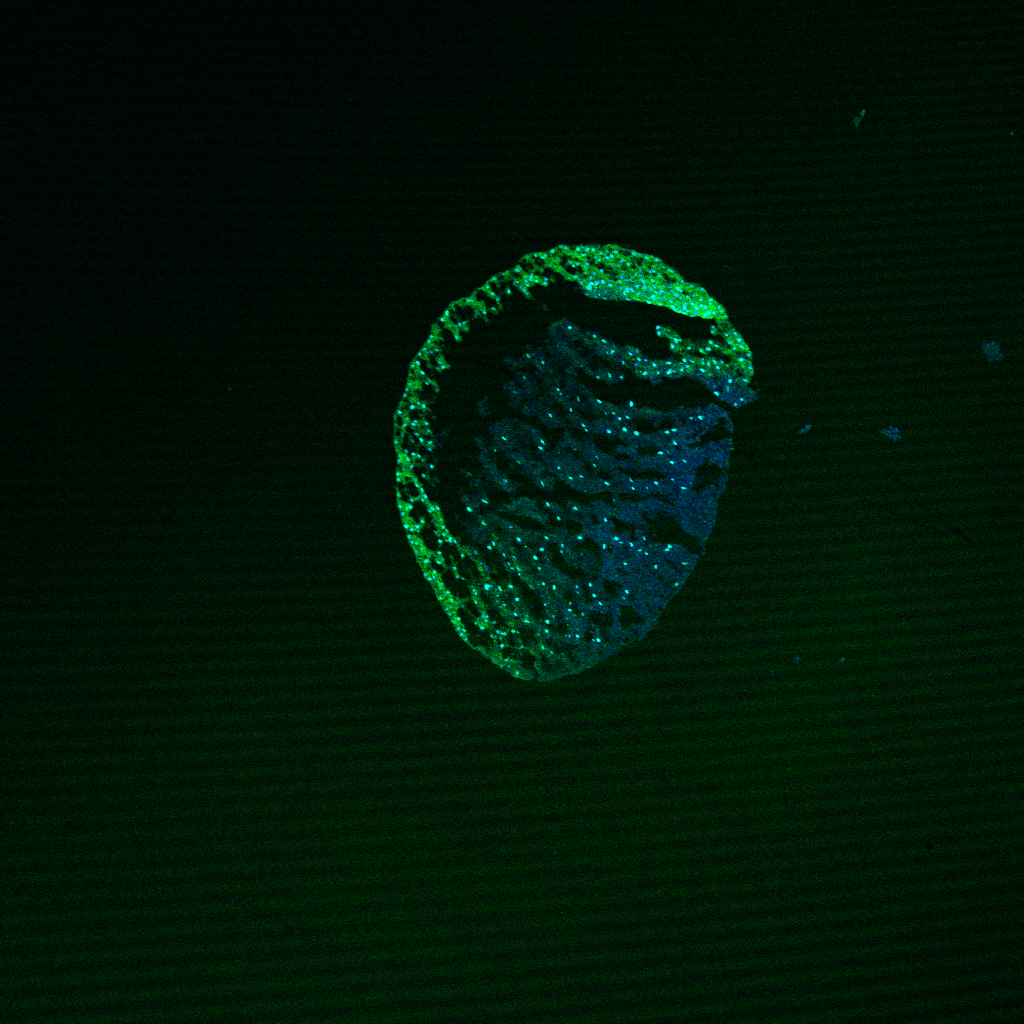

Supplement: Supplementary file 2 — Source Data Fig. 1 [file 44319_2023_46_MOESM2_ESM.zip › Figure 1/1E/image 1E merge.tif]

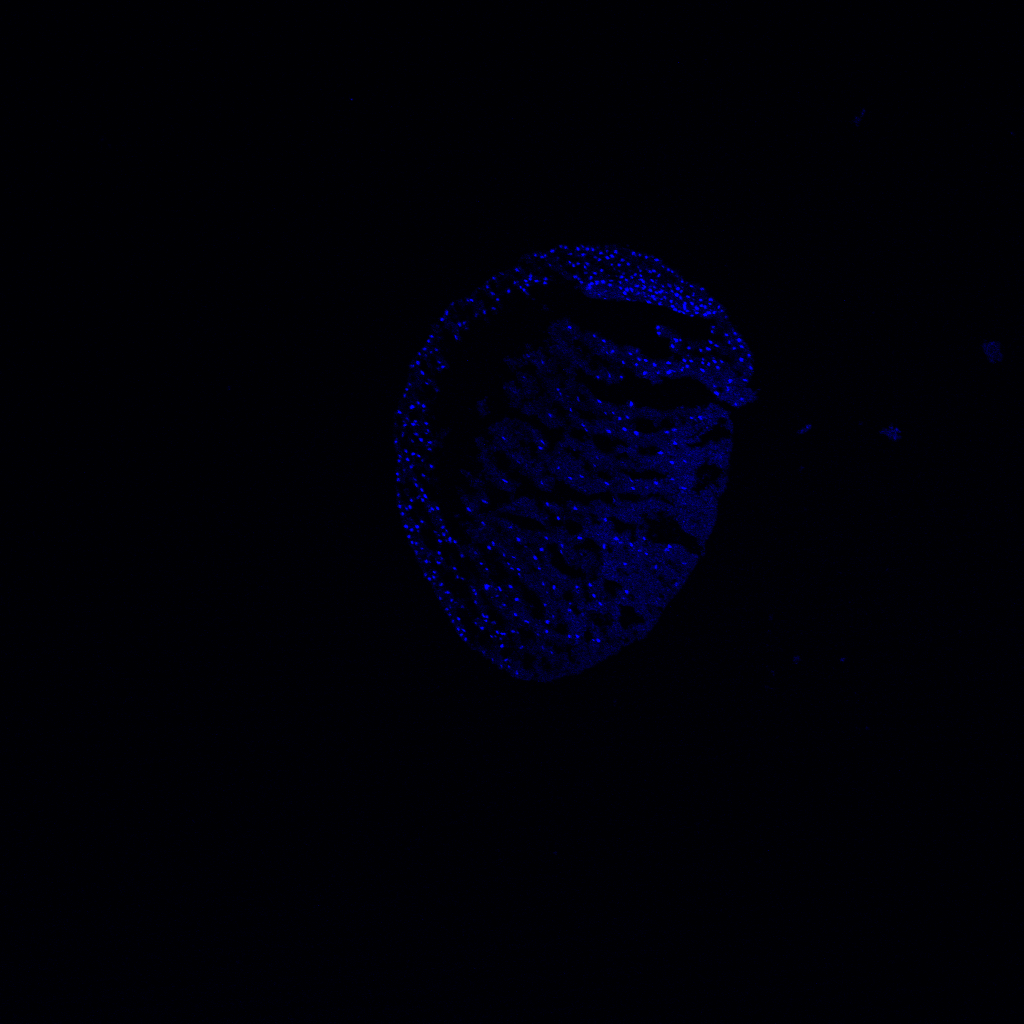

Supplement: Supplementary file 2 — Source Data Fig. 1 [file 44319_2023_46_MOESM2_ESM.zip › Figure 1/1E/image 1E DAPI.tif]

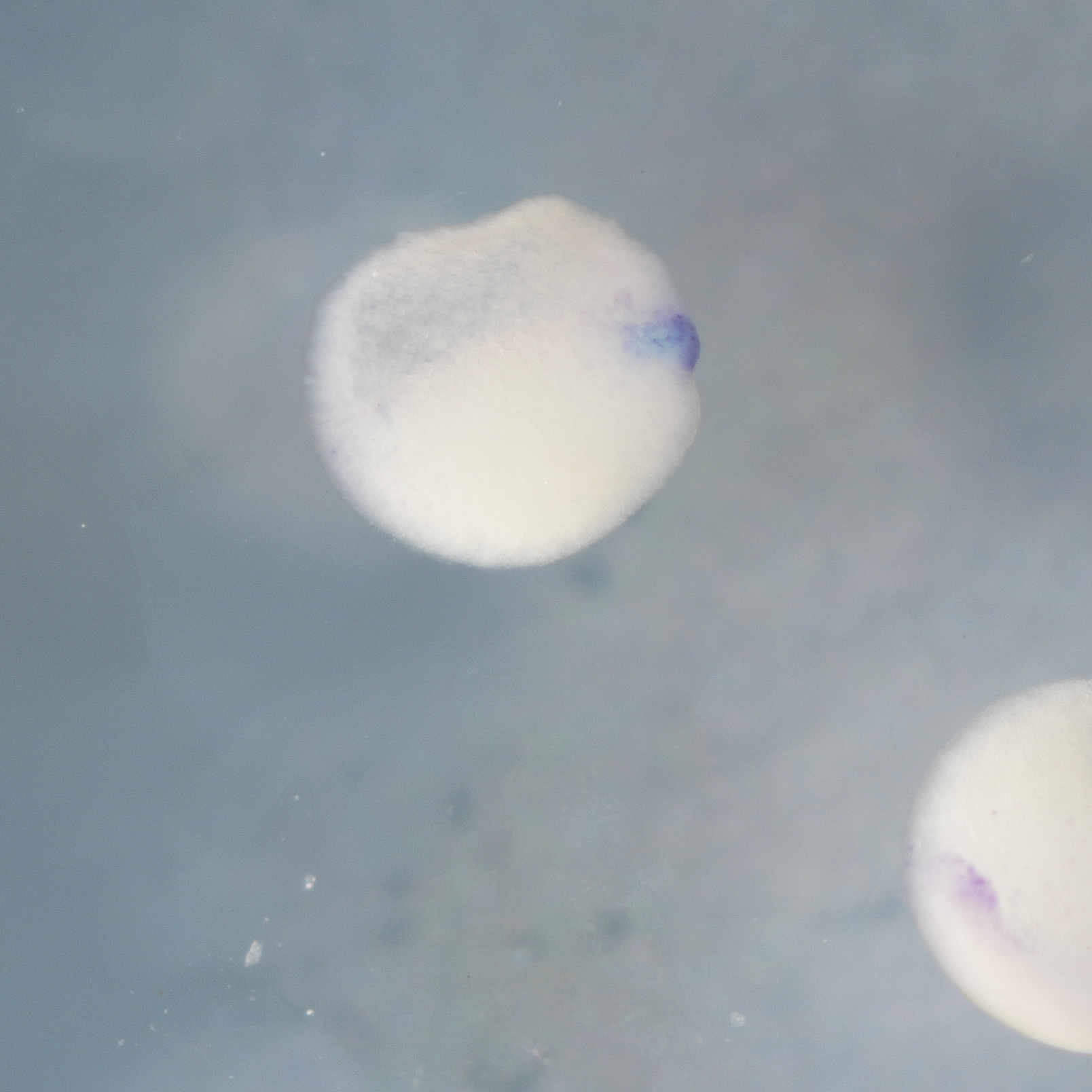

Supplement: Supplementary file 2 — Source Data Fig. 1 [file 44319_2023_46_MOESM2_ESM.zip › Figure 1/1C/image 1C st10.5.tif]

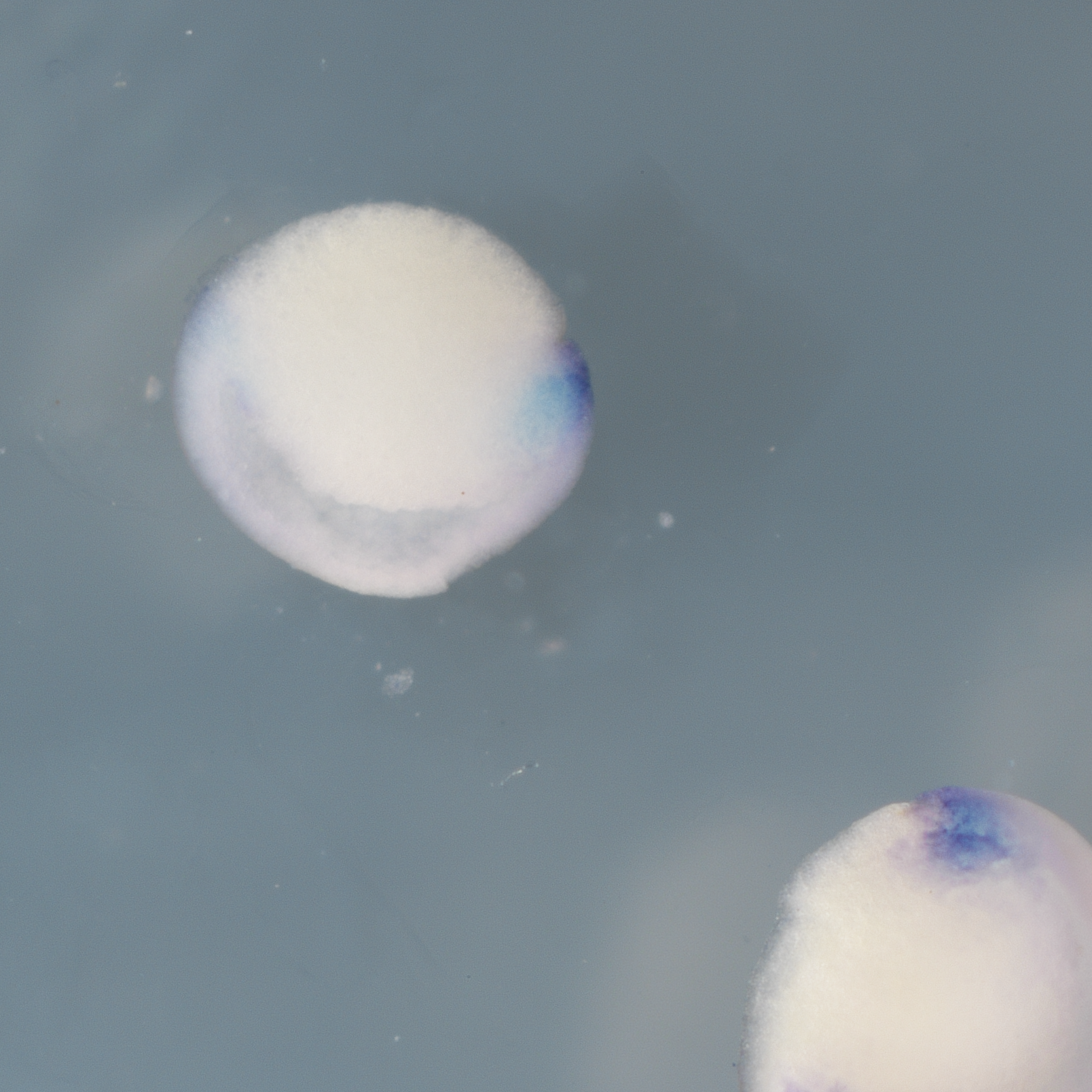

Supplement: Supplementary file 2 — Source Data Fig. 1 [file 44319_2023_46_MOESM2_ESM.zip › Figure 1/1C/image 1C st11.tif]

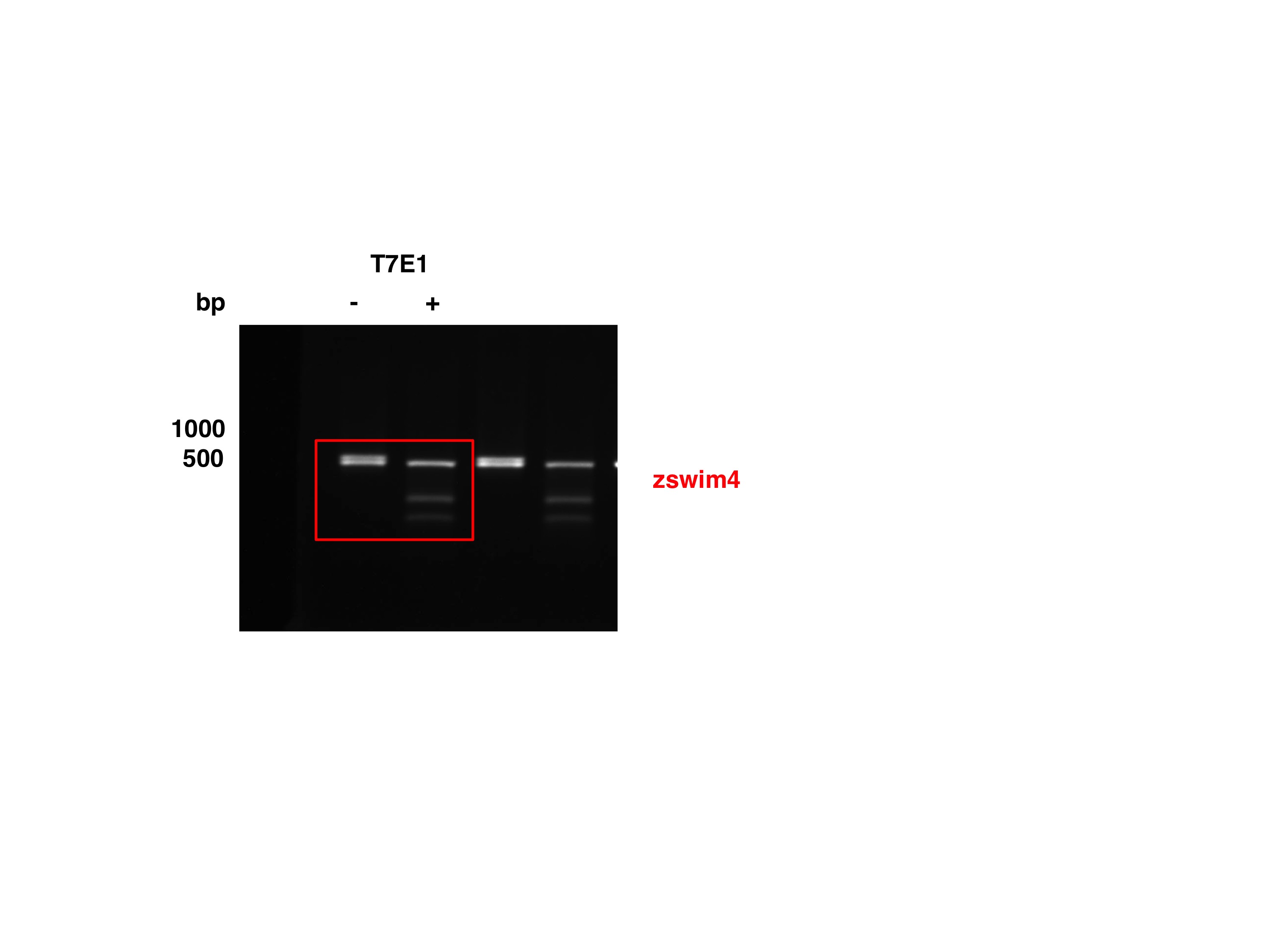

Supplement: Supplementary file 3 — Source Data Fig. 2 [file 44319_2023_46_MOESM3_ESM.zip › Figure 2/2G/DNA gel 2G T7E1.jpg]

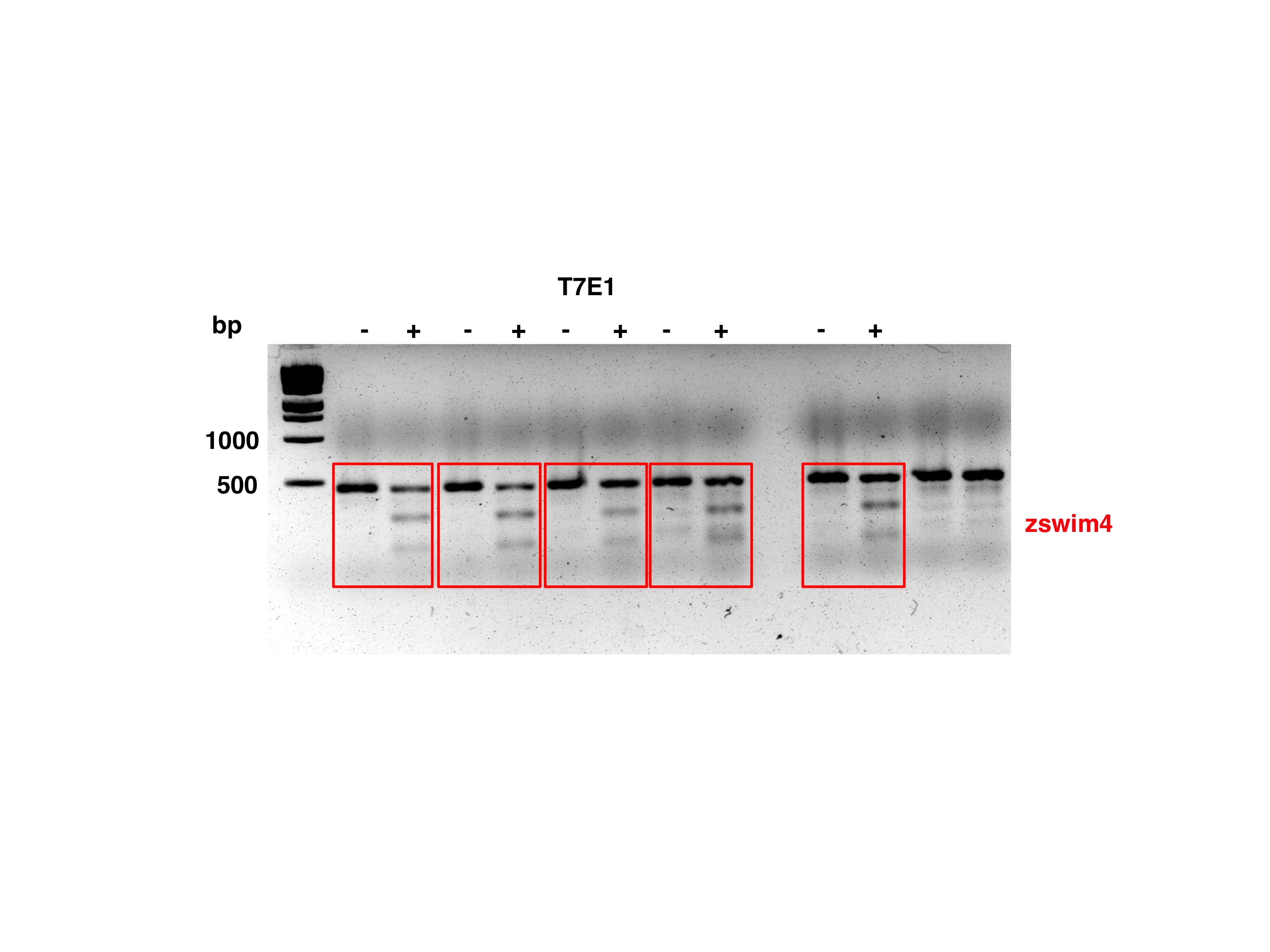

Supplement: Supplementary file 3 — Source Data Fig. 2 [file 44319_2023_46_MOESM3_ESM.zip › Figure 2/2I/DNA gel 2I T7E1.jpg]

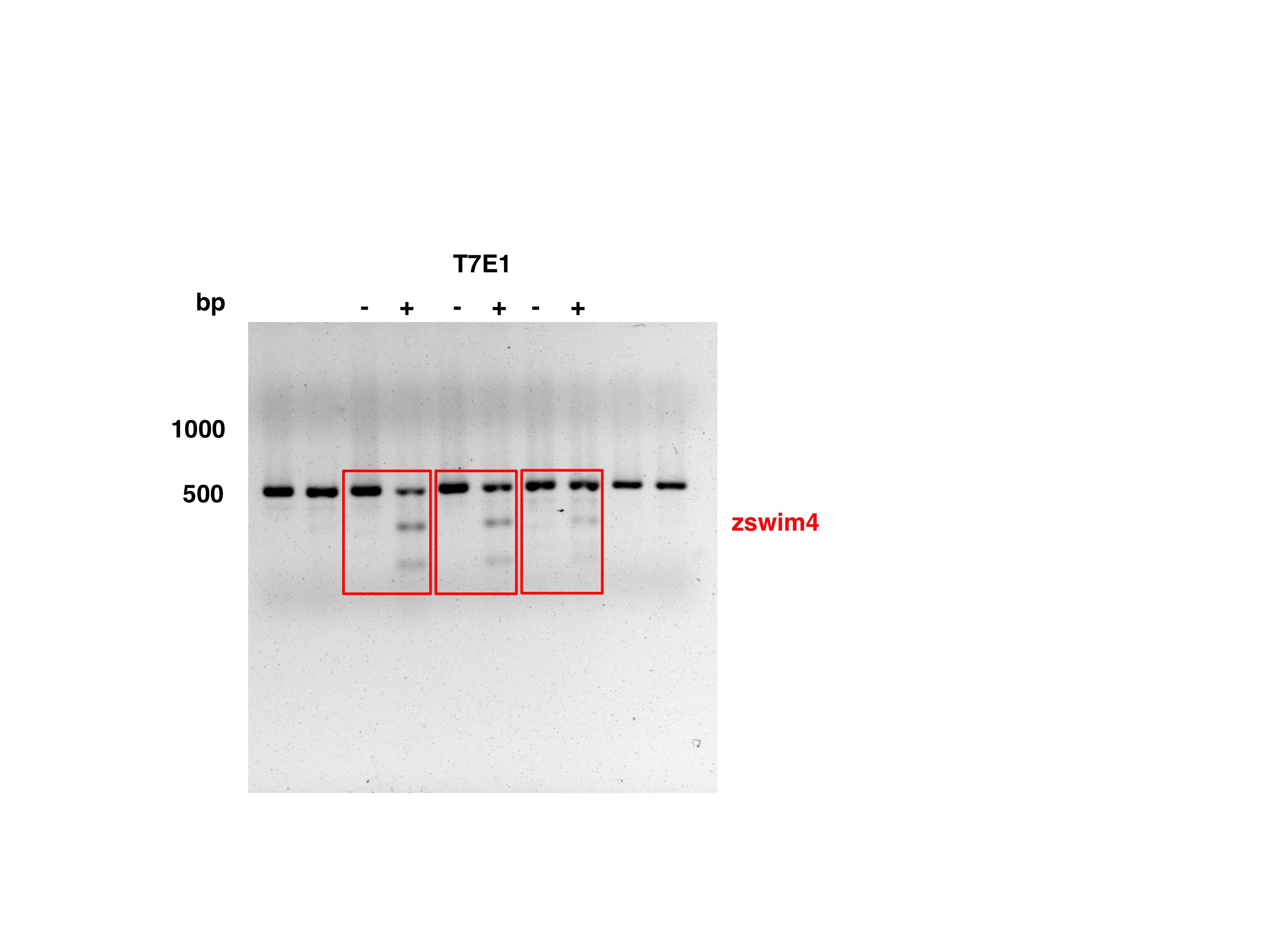

Supplement: Supplementary file 3 — Source Data Fig. 2 [file 44319_2023_46_MOESM3_ESM.zip › Figure 2/2H/DNA gel 2H T7E1.jpg]

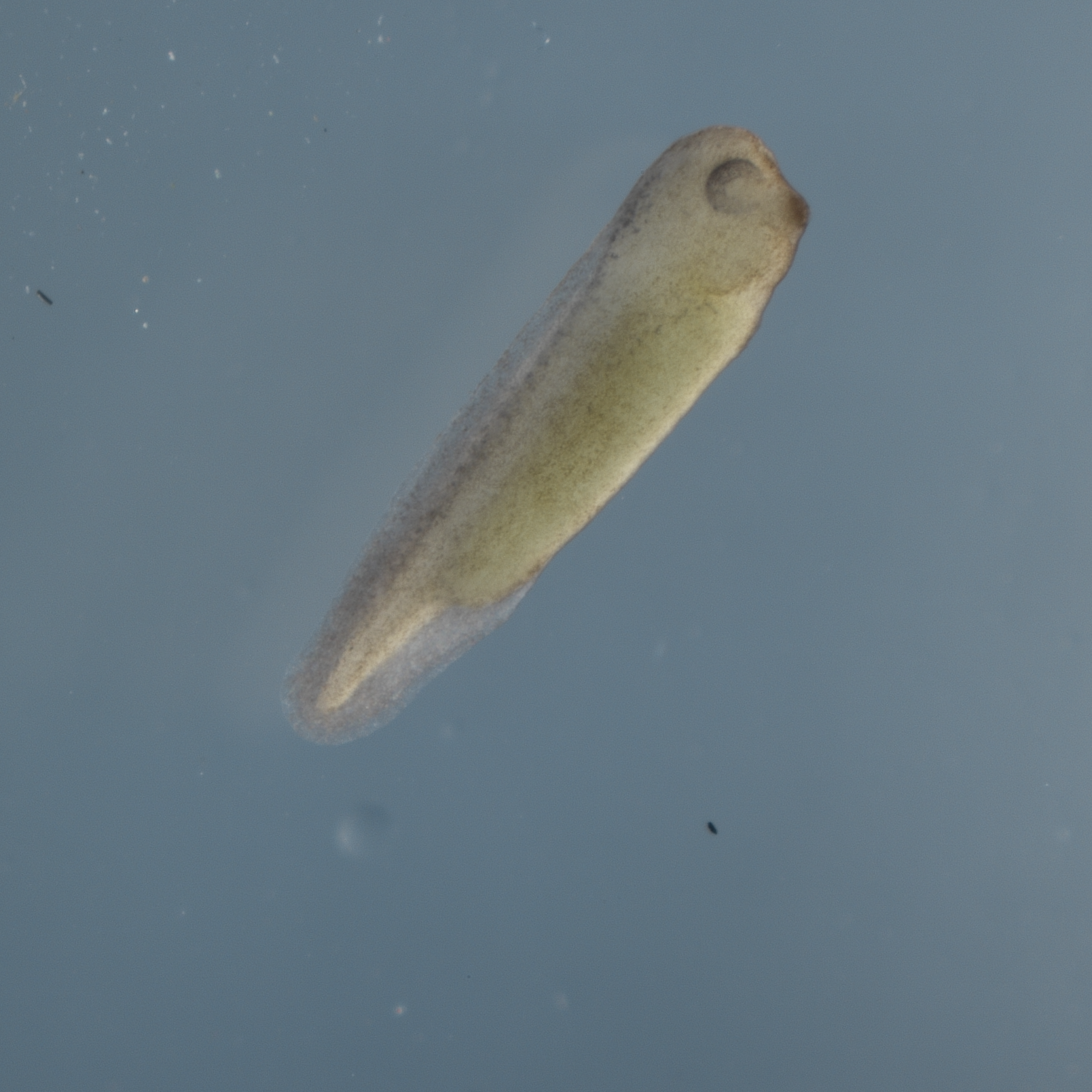

Supplement: Supplementary file 3 — Source Data Fig. 2 [file 44319_2023_46_MOESM3_ESM.zip › Figure 2/2A/image 2A normal.tif]

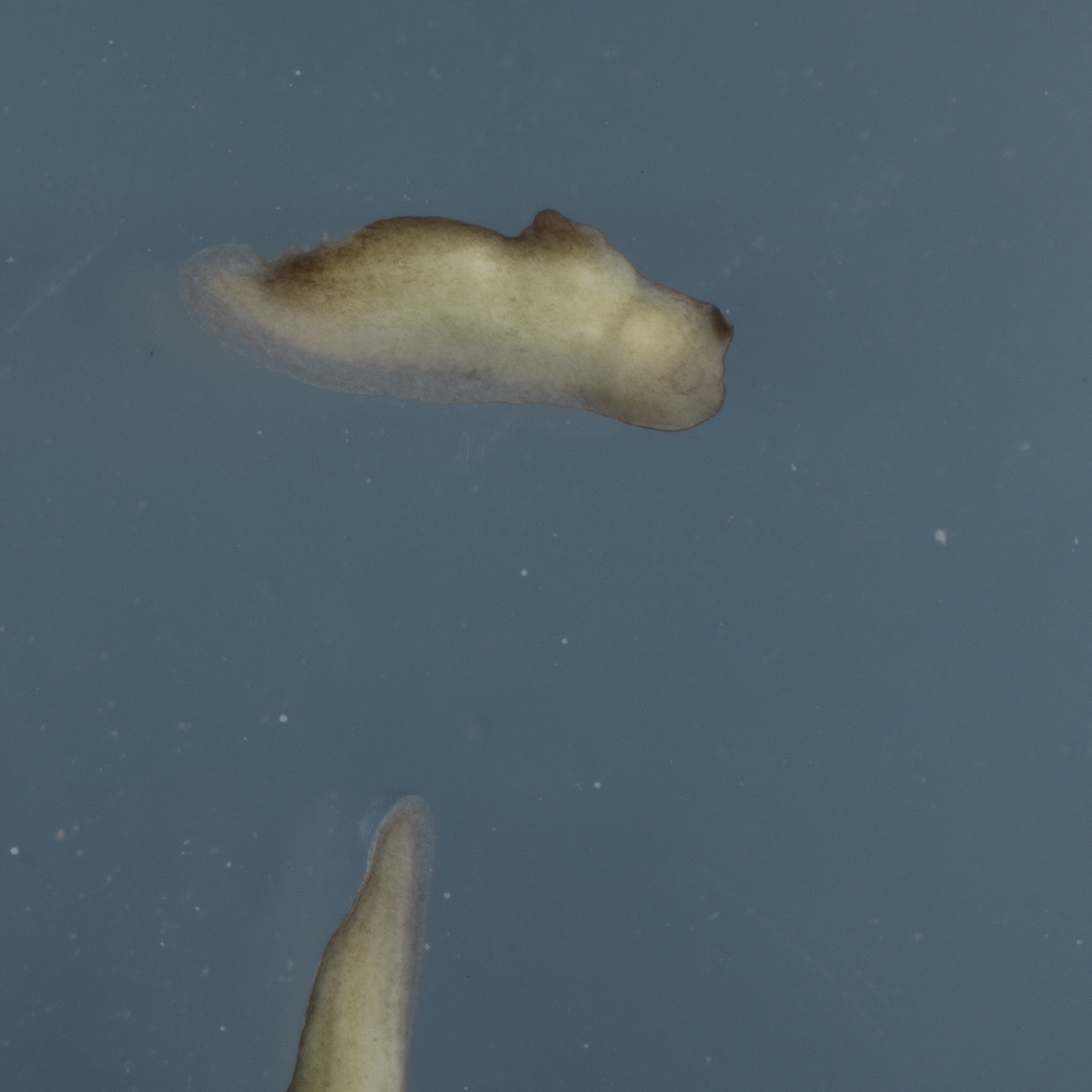

Supplement: Supplementary file 3 — Source Data Fig. 2 [file 44319_2023_46_MOESM3_ESM.zip › Figure 2/2A/image 2A moderate.tif]

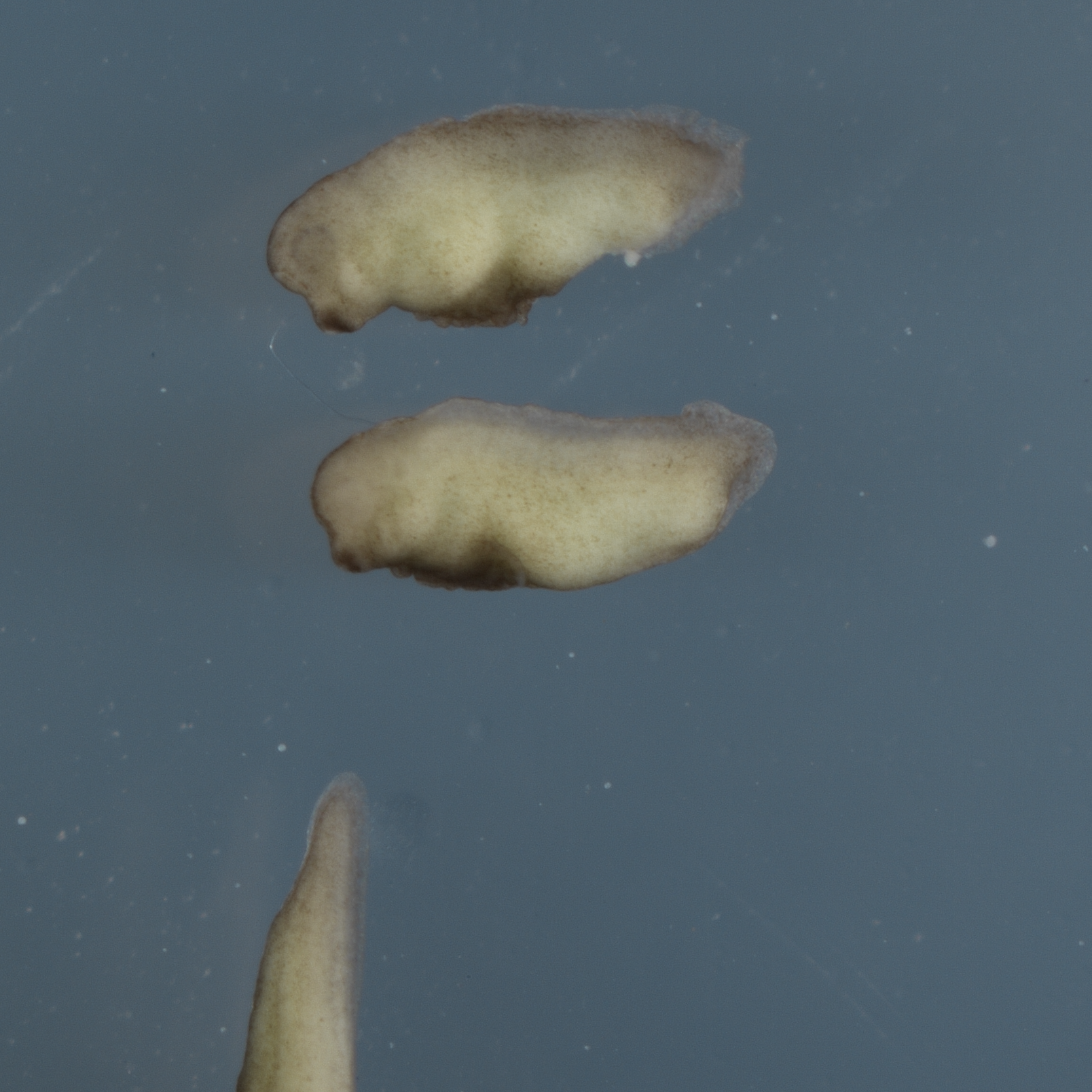

Supplement: Supplementary file 3 — Source Data Fig. 2 [file 44319_2023_46_MOESM3_ESM.zip › Figure 2/2A/image 2A severe.tif]

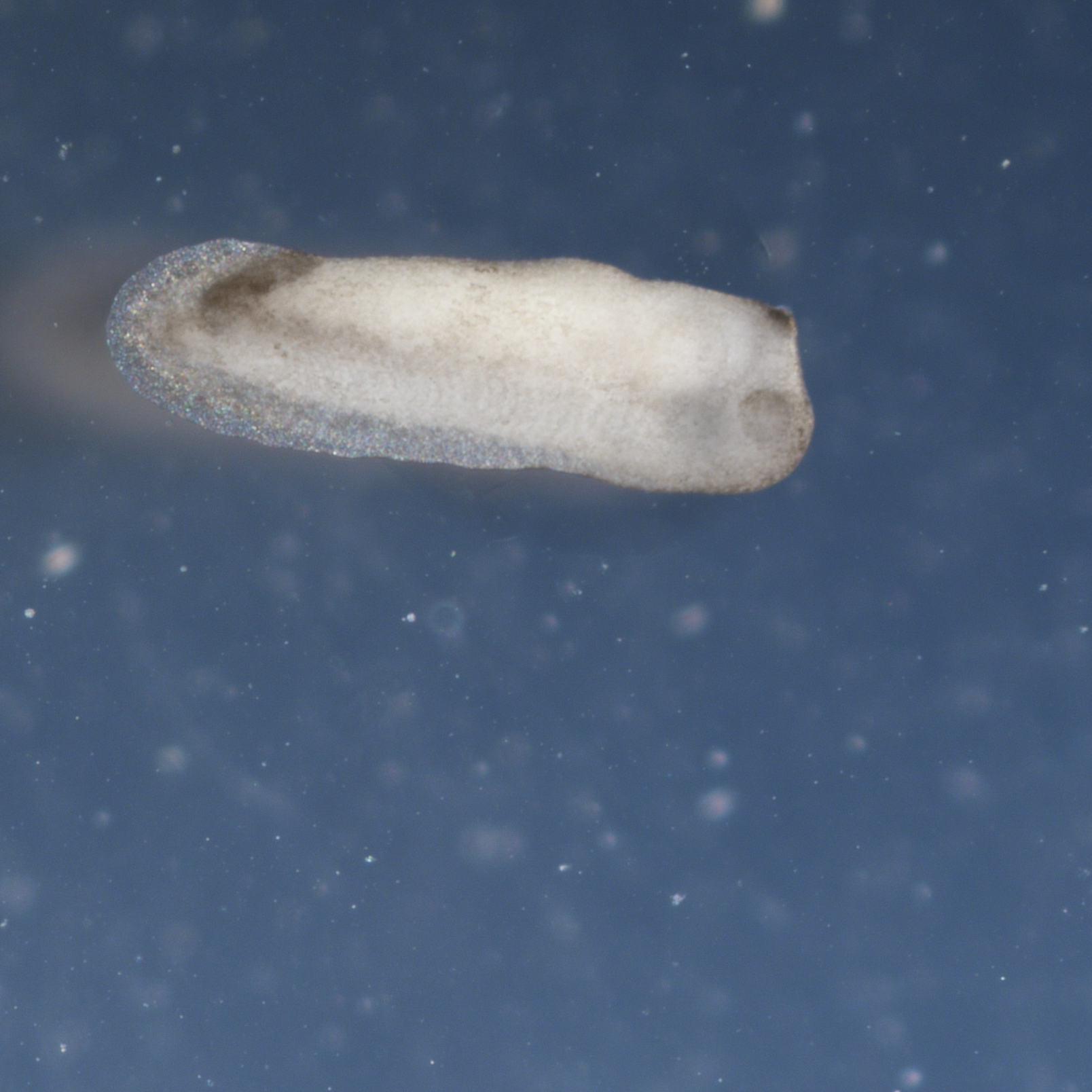

Supplement: Supplementary file 3 — Source Data Fig. 2 [file 44319_2023_46_MOESM3_ESM.zip › Figure 2/2F/image 2F con-st28.tif]

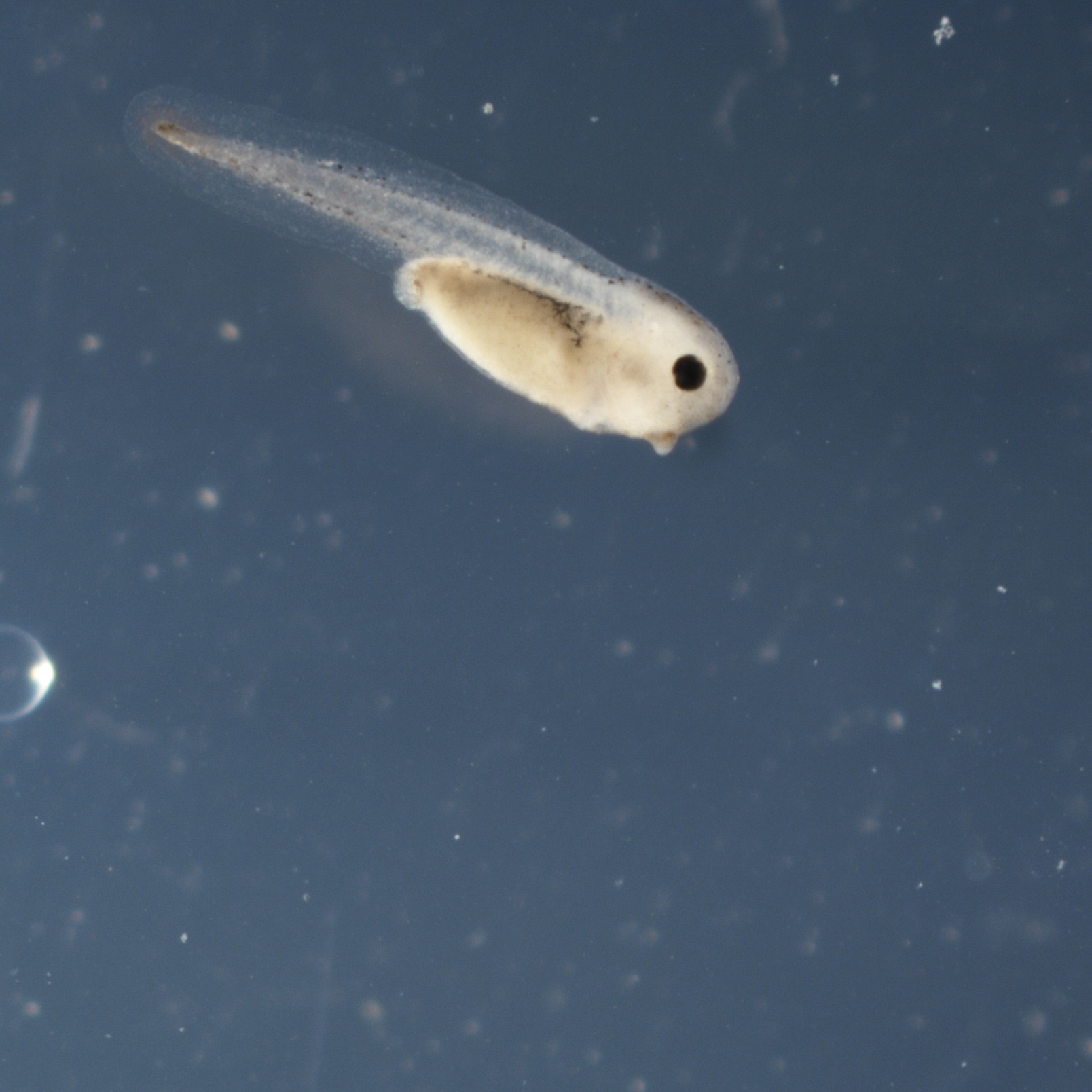

Supplement: Supplementary file 3 — Source Data Fig. 2 [file 44319_2023_46_MOESM3_ESM.zip › Figure 2/2F/image con-st38.tif]

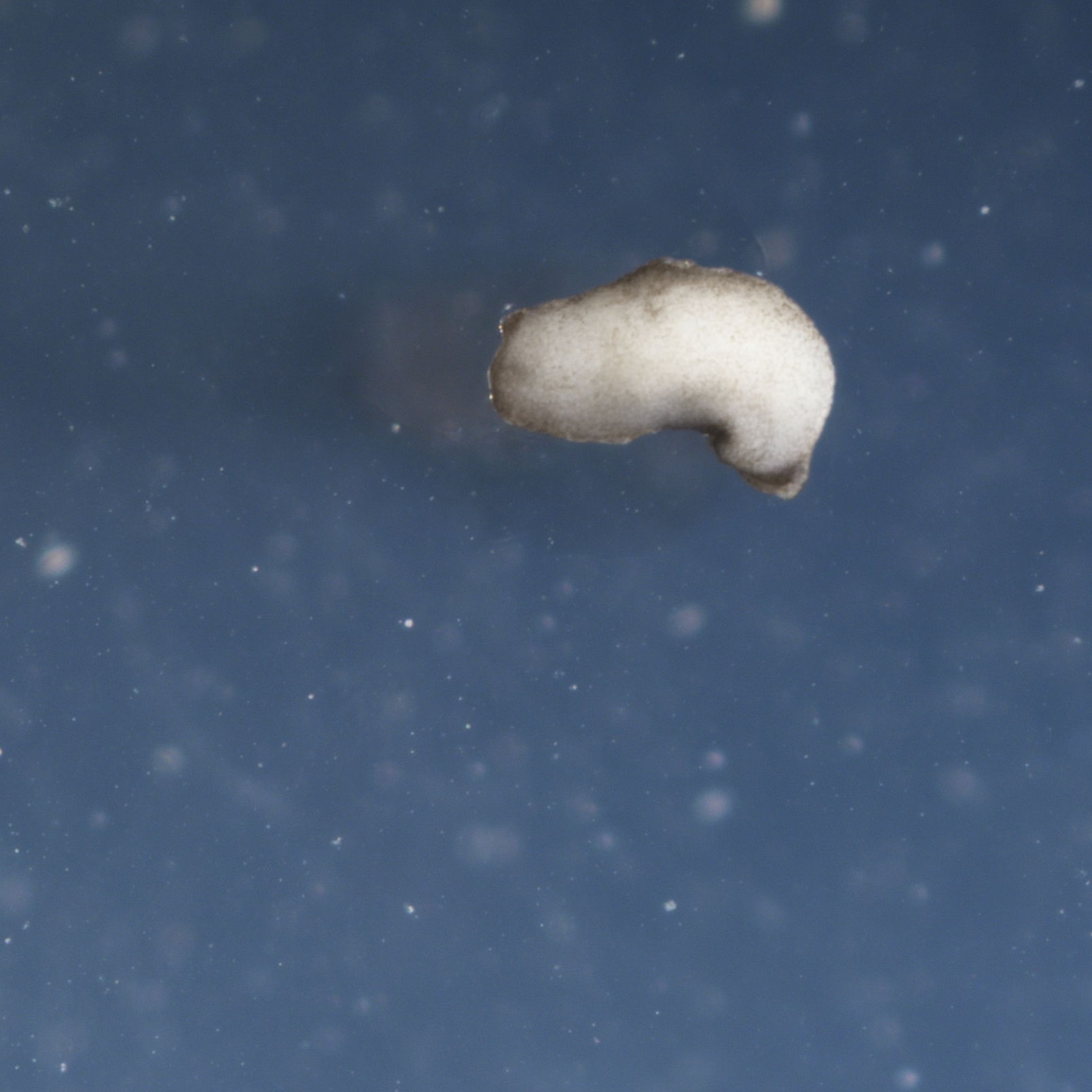

Supplement: Supplementary file 3 — Source Data Fig. 2 [file 44319_2023_46_MOESM3_ESM.zip › Figure 2/2F/image 2F zswim4-CRISPR-st28.tif]

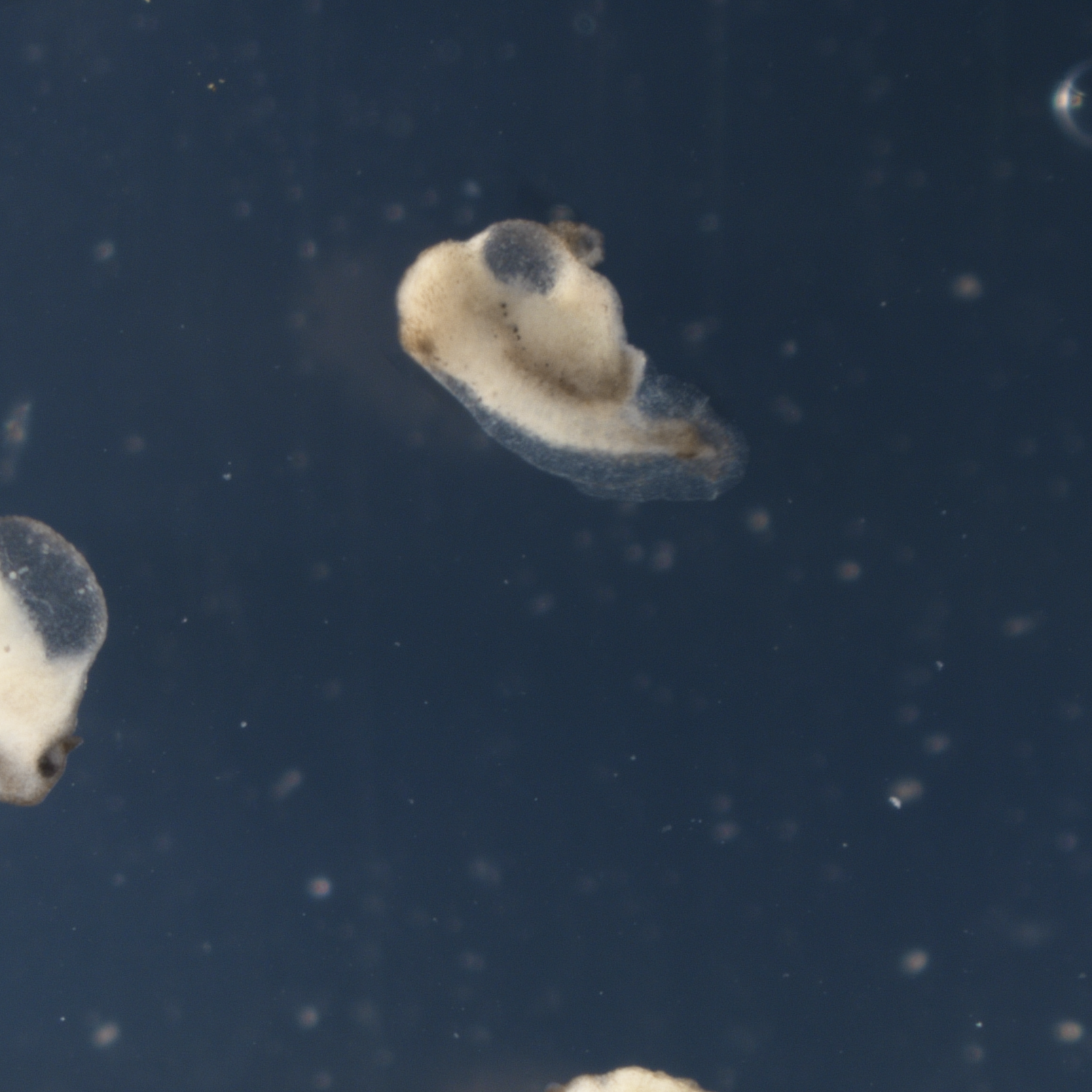

Supplement: Supplementary file 3 — Source Data Fig. 2 [file 44319_2023_46_MOESM3_ESM.zip › Figure 2/2F/image zswim4-CRISPR-st38.tif]

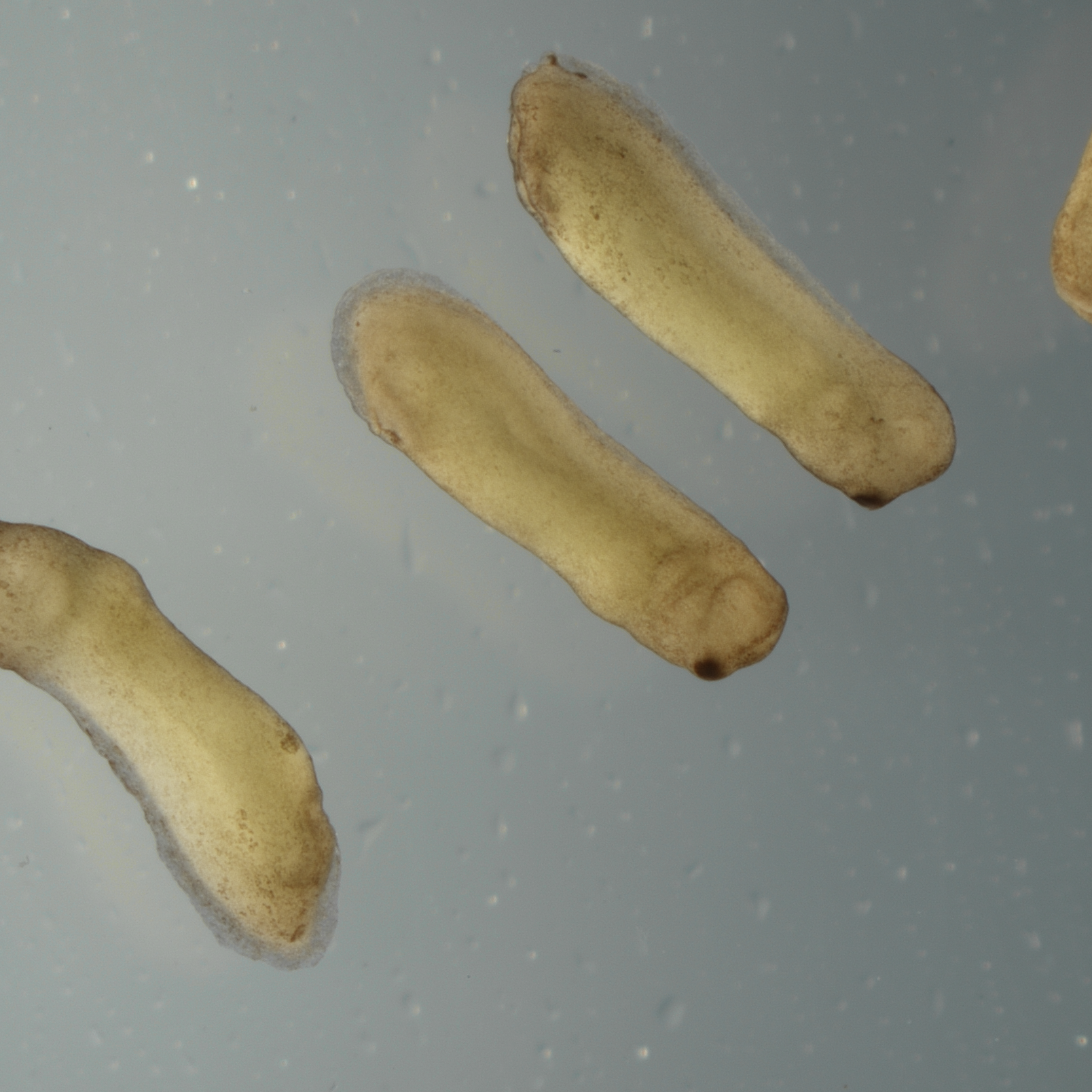

Supplement: Supplementary file 3 — Source Data Fig. 2 [file 44319_2023_46_MOESM3_ESM.zip › Figure 2/2C/image 2C normal.tif]

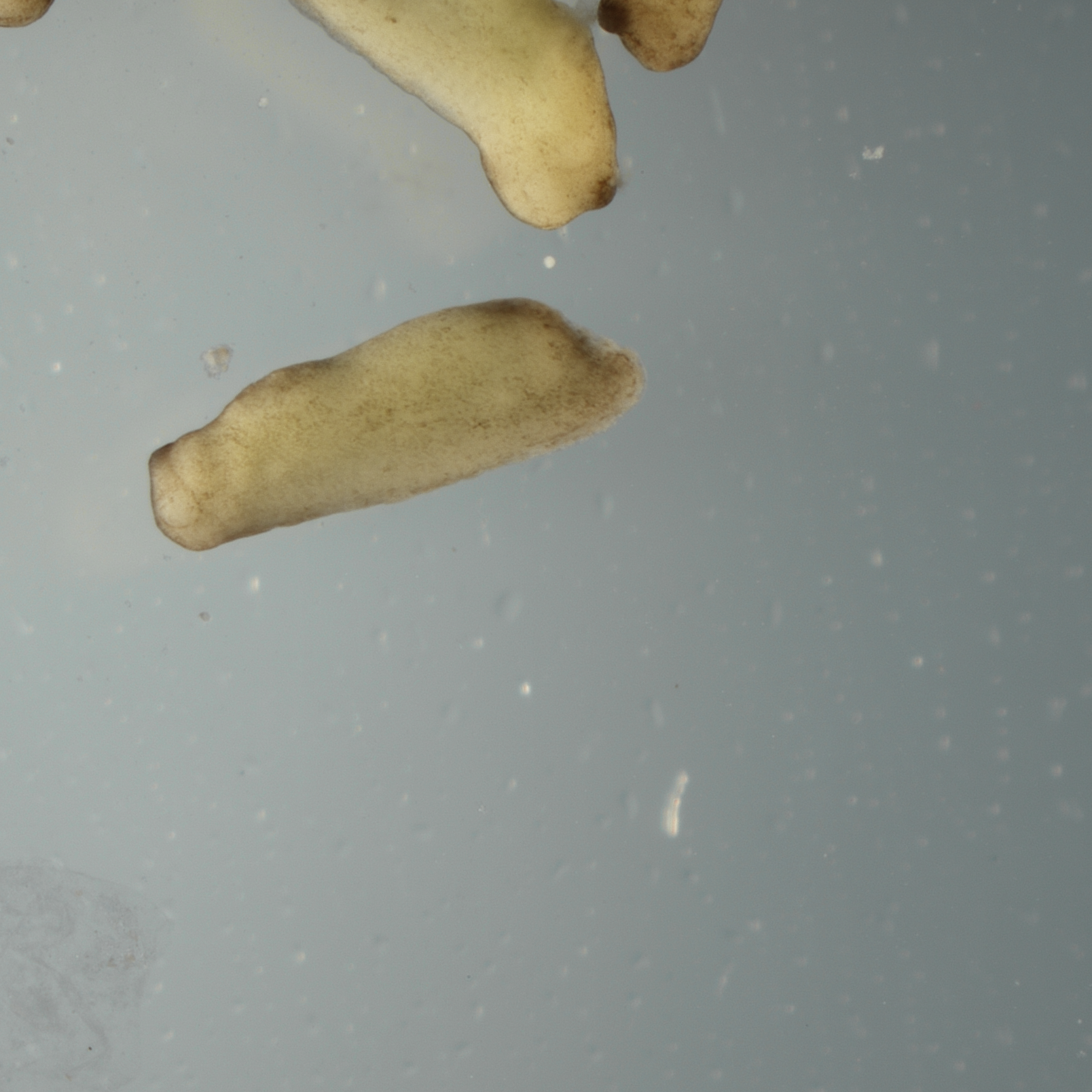

Supplement: Supplementary file 3 — Source Data Fig. 2 [file 44319_2023_46_MOESM3_ESM.zip › Figure 2/2C/image 2C mild.tif]

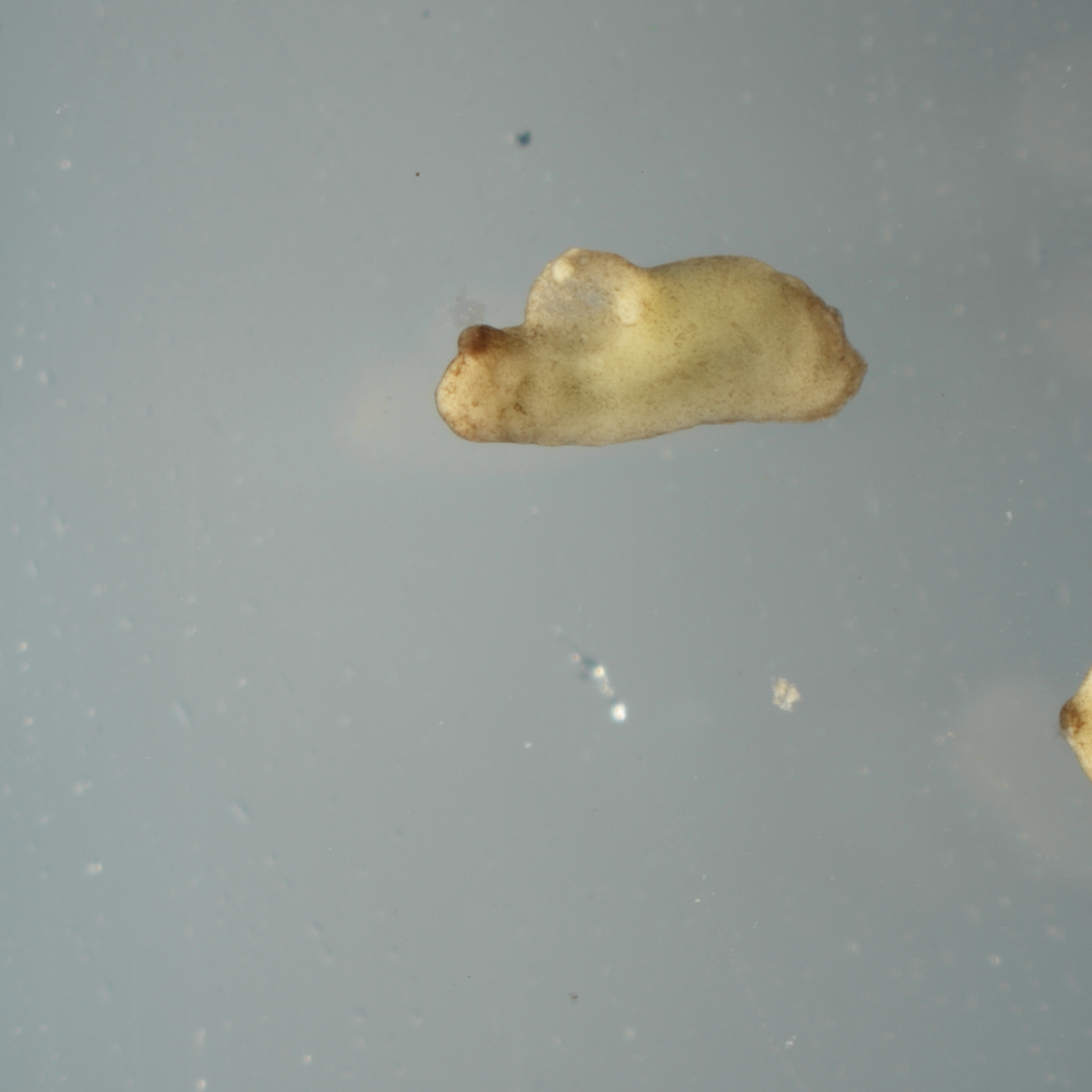

Supplement: Supplementary file 3 — Source Data Fig. 2 [file 44319_2023_46_MOESM3_ESM.zip › Figure 2/2C/image 2C moderate.tif]

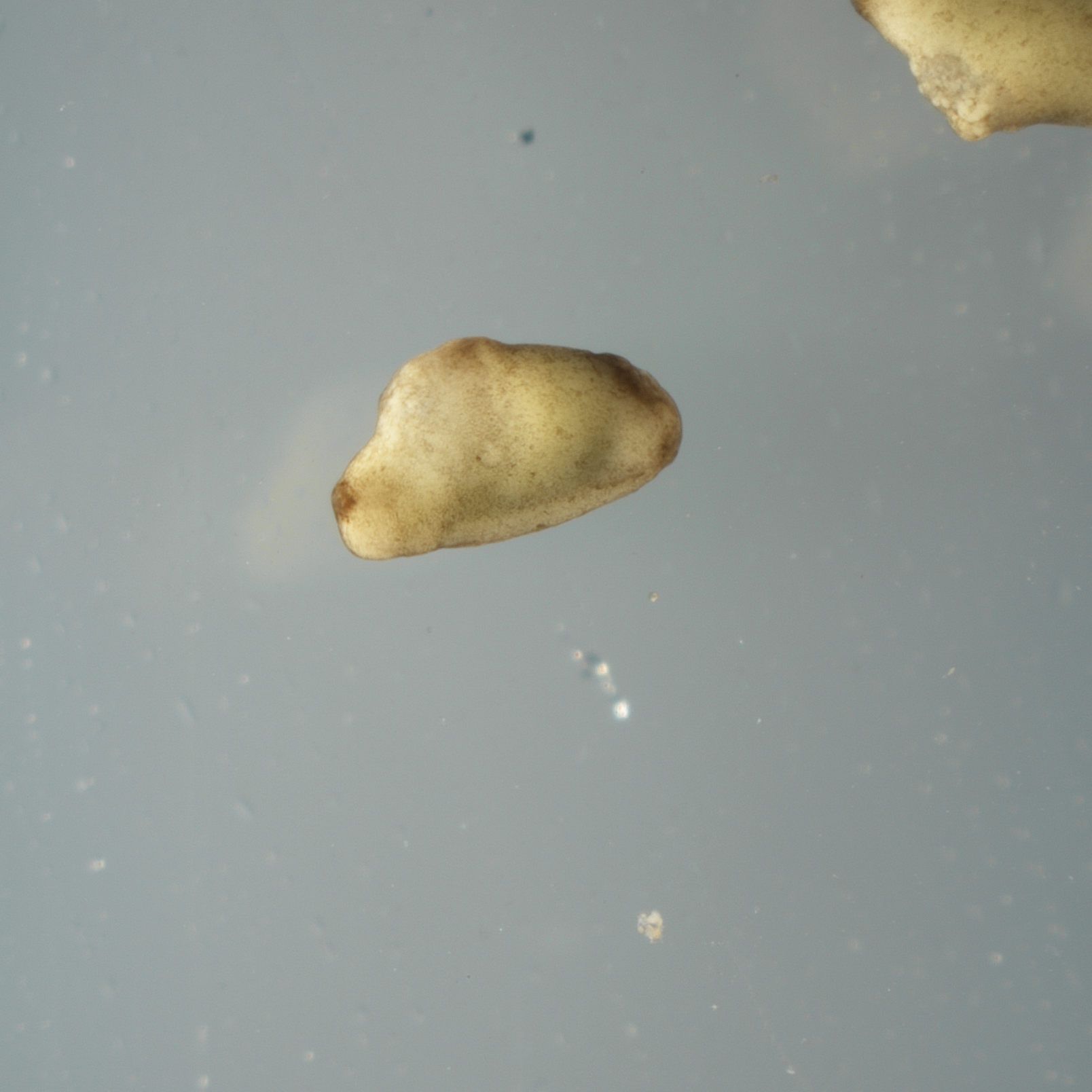

Supplement: Supplementary file 3 — Source Data Fig. 2 [file 44319_2023_46_MOESM3_ESM.zip › Figure 2/2C/image 2C severe.tif]

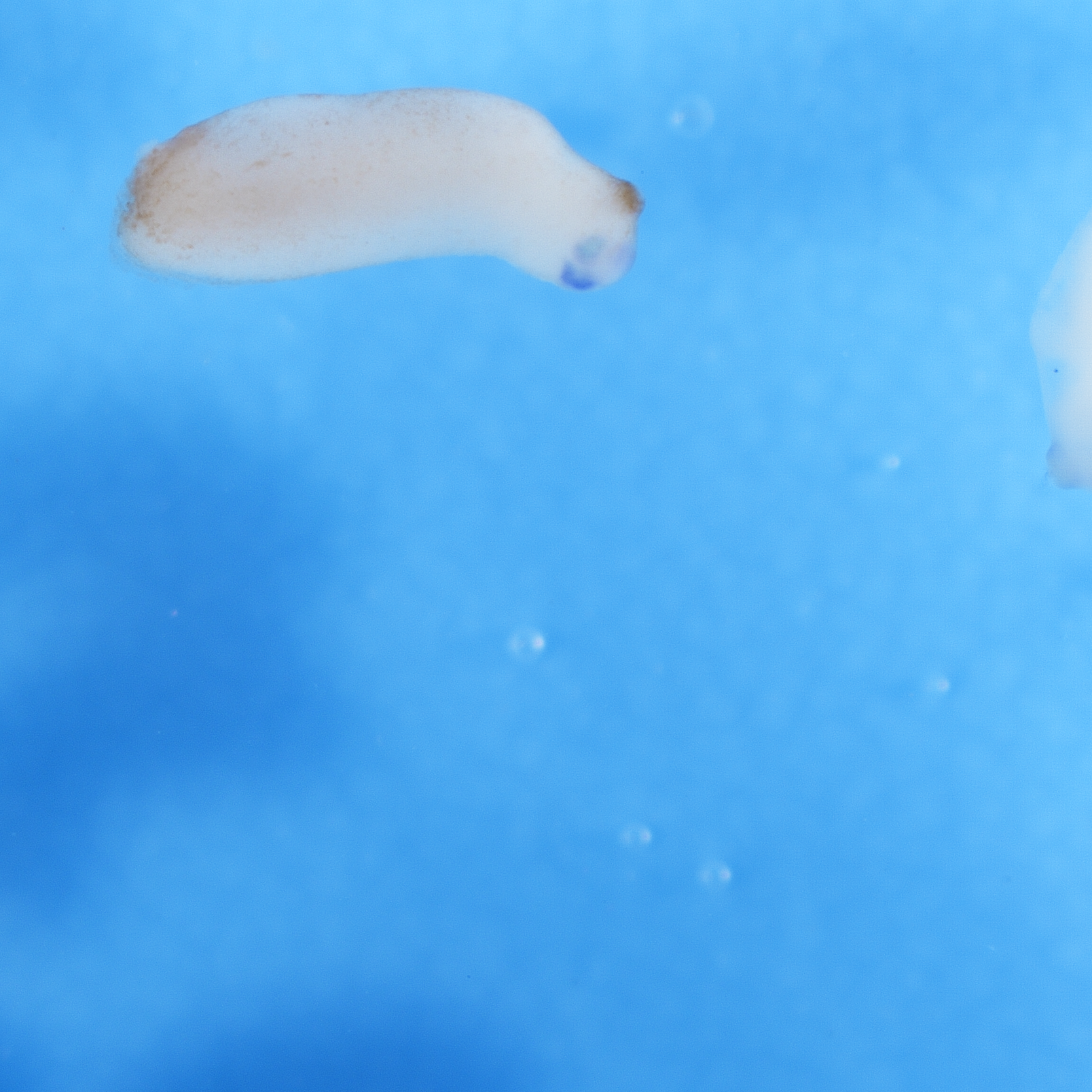

Supplement: Supplementary file 3 — Source Data Fig. 2 [file 44319_2023_46_MOESM3_ESM.zip › Figure 2/2D/image 2D mild.tif]

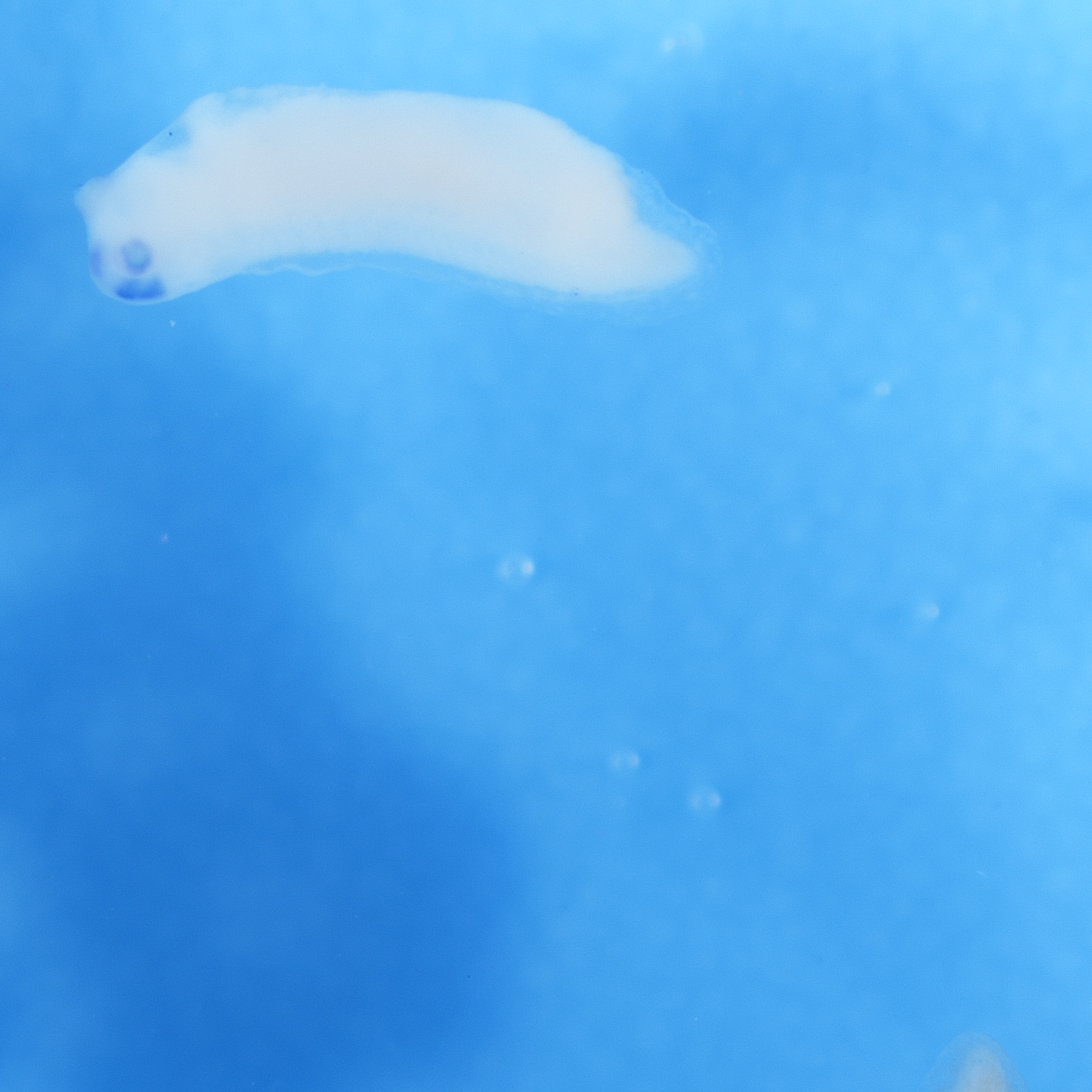

Supplement: Supplementary file 3 — Source Data Fig. 2 [file 44319_2023_46_MOESM3_ESM.zip › Figure 2/2D/image 2D normal.tif]

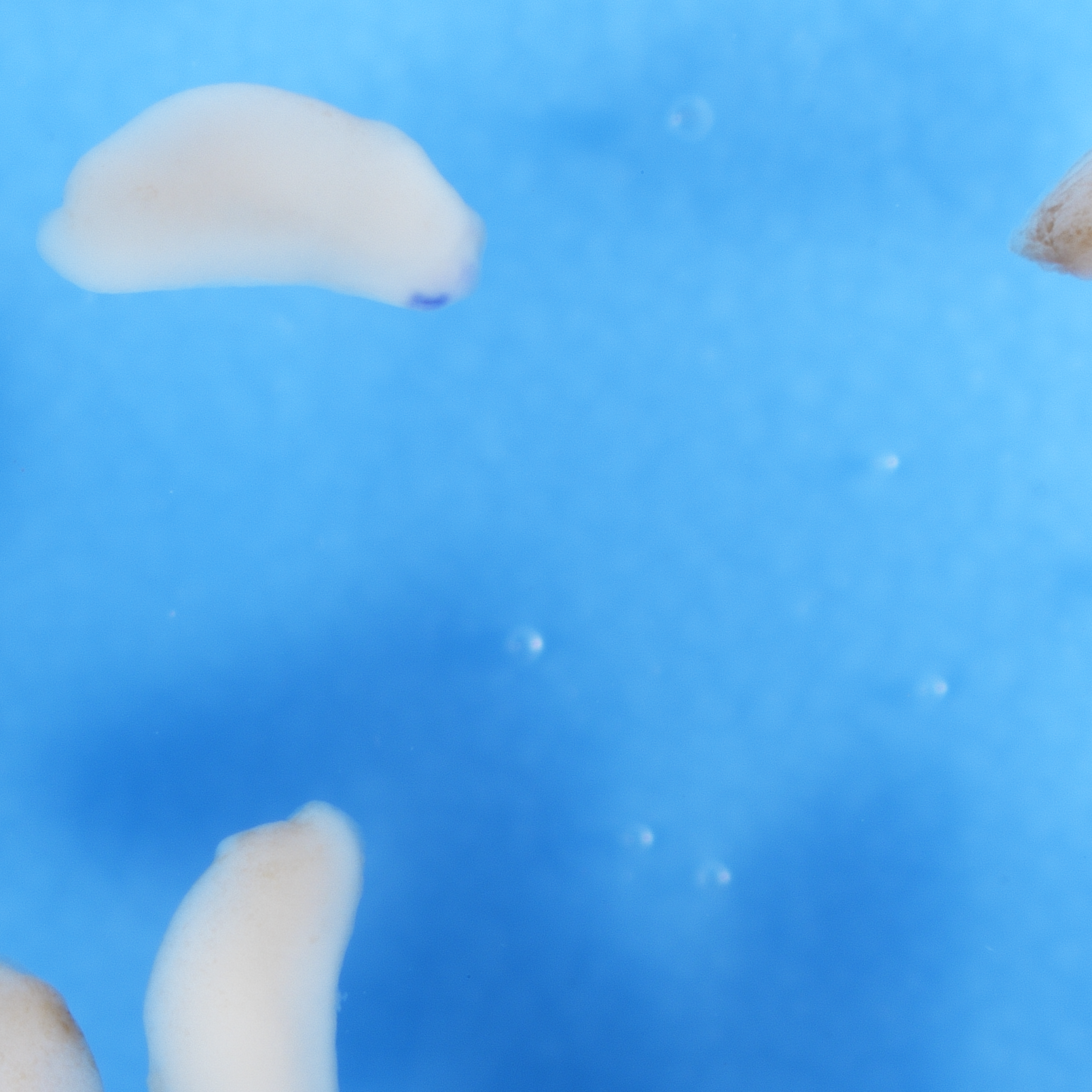

Supplement: Supplementary file 3 — Source Data Fig. 2 [file 44319_2023_46_MOESM3_ESM.zip › Figure 2/2D/image 2D severe.tif]

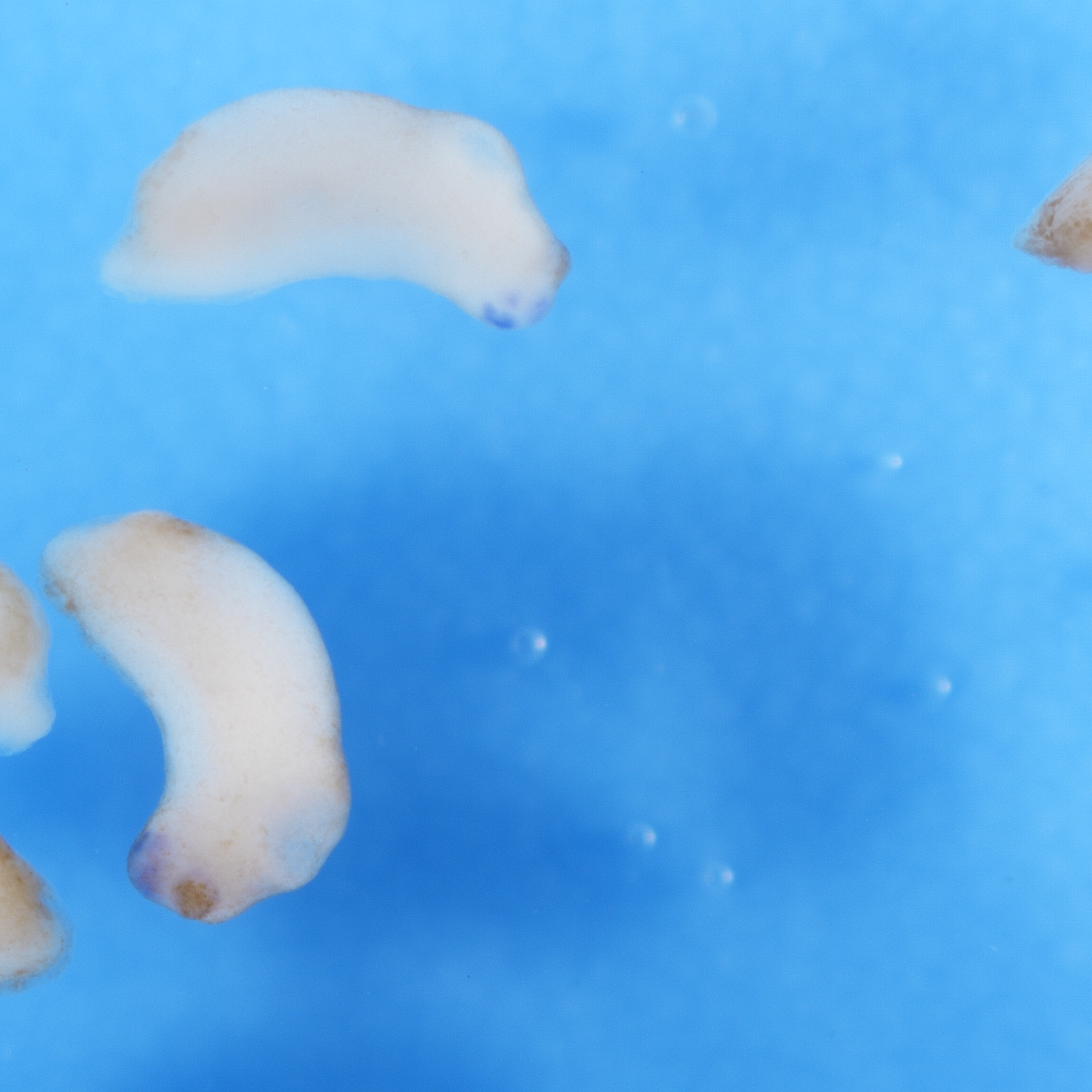

Supplement: Supplementary file 3 — Source Data Fig. 2 [file 44319_2023_46_MOESM3_ESM.zip › Figure 2/2D/image 2D moderate.tif]

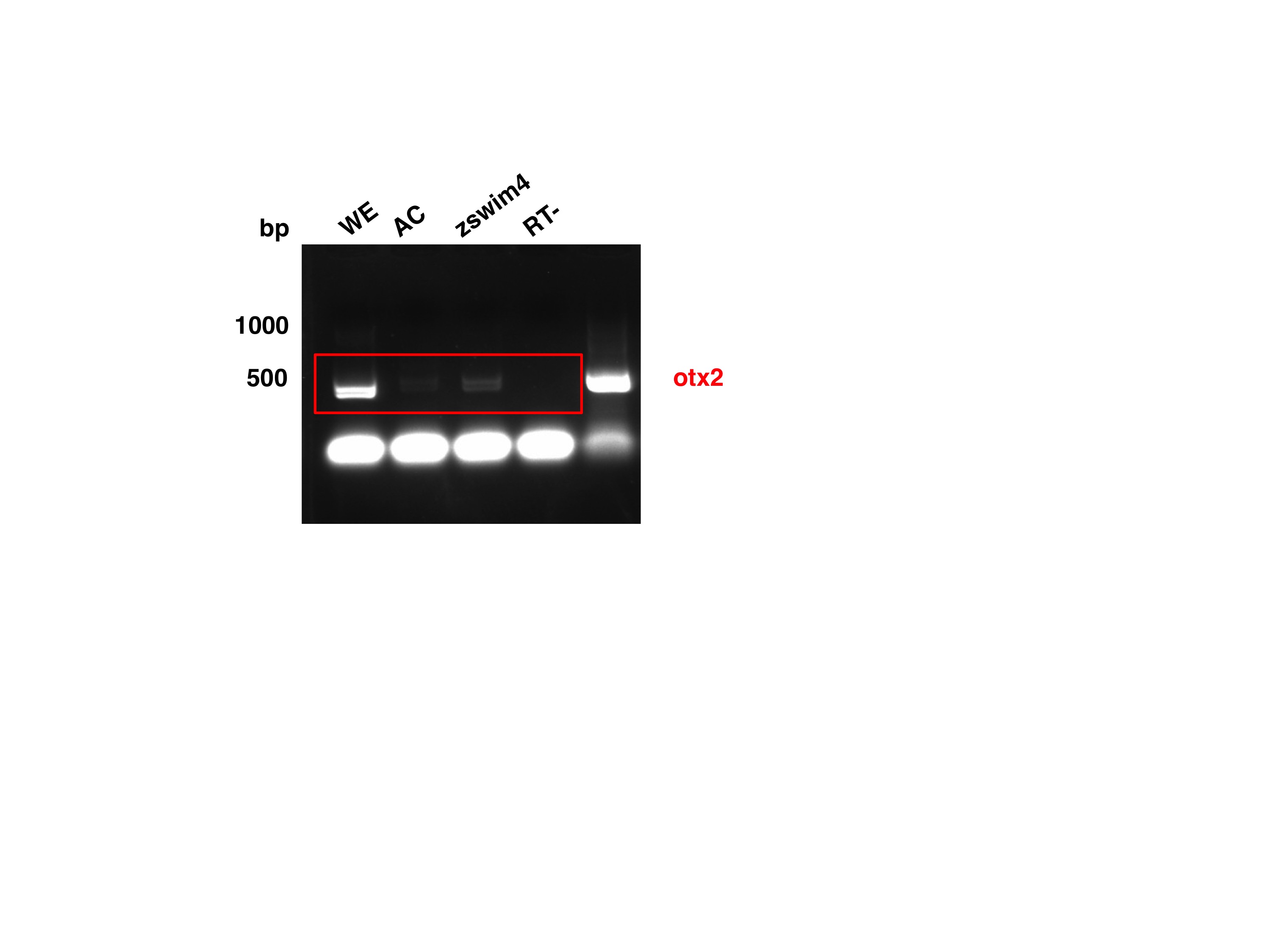

Supplement: Supplementary file 4 — Source Data Fig. 3 [file 44319_2023_46_MOESM4_ESM.zip › Figure 3/3K/DNA gel 3K otx2.jpg]

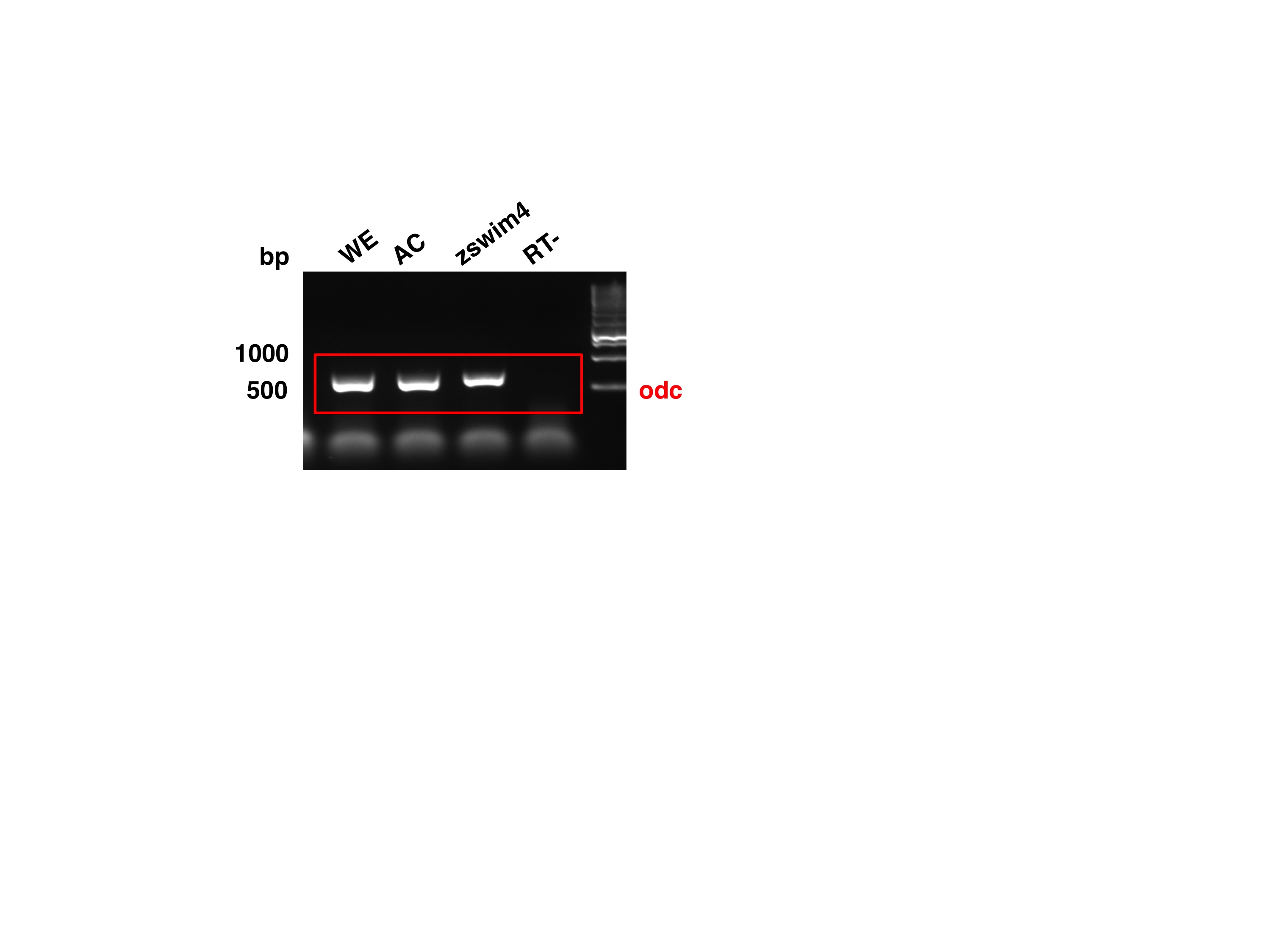

Supplement: Supplementary file 4 — Source Data Fig. 3 [file 44319_2023_46_MOESM4_ESM.zip › Figure 3/3K/DNA gel 3K odc.jpg]

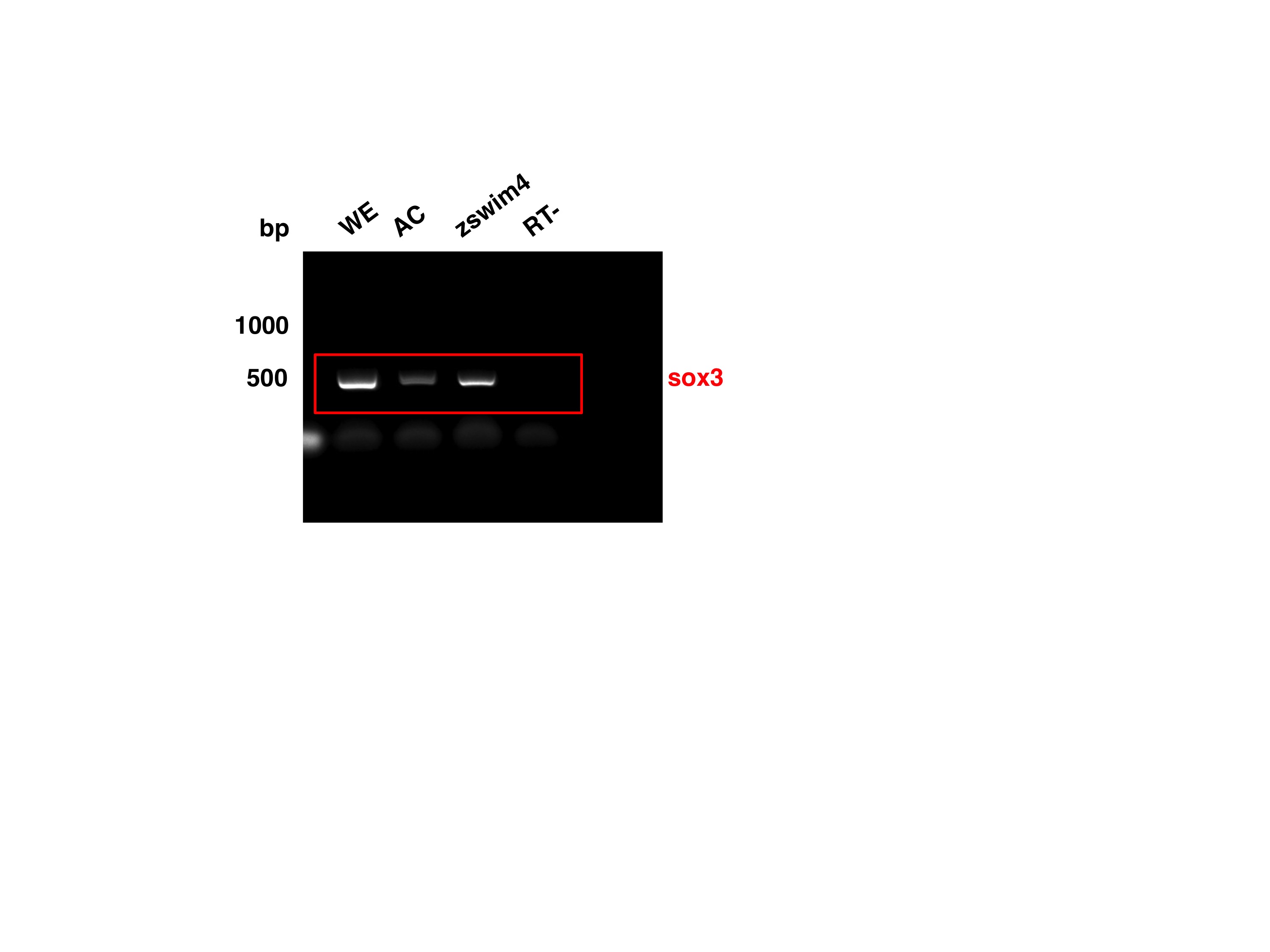

Supplement: Supplementary file 4 — Source Data Fig. 3 [file 44319_2023_46_MOESM4_ESM.zip › Figure 3/3K/DNA gel 3K sox3.jpg]

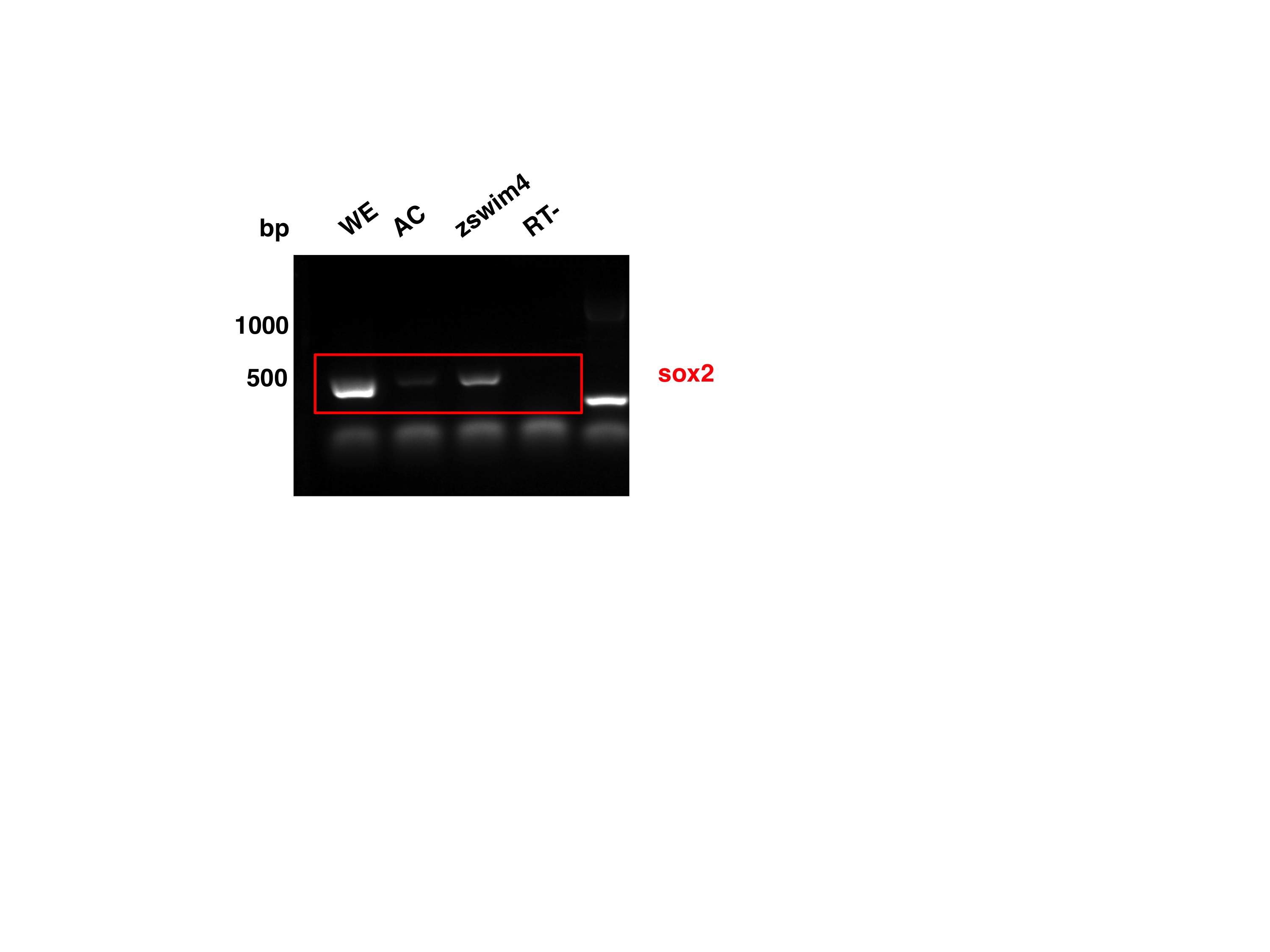

Supplement: Supplementary file 4 — Source Data Fig. 3 [file 44319_2023_46_MOESM4_ESM.zip › Figure 3/3K/DNA gel 3K sox2.jpg]

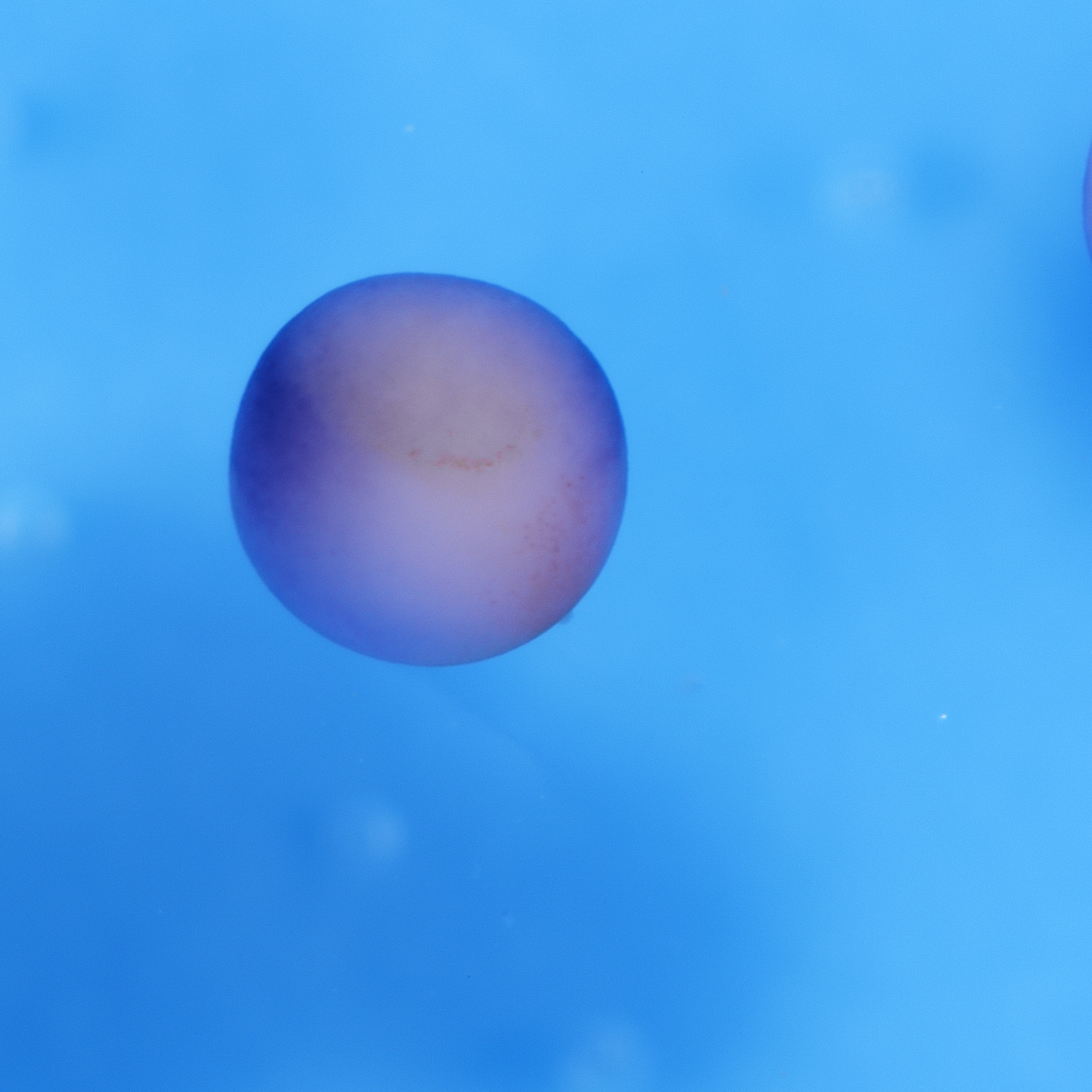

Supplement: Supplementary file 4 — Source Data Fig. 3 [file 44319_2023_46_MOESM4_ESM.zip › Figure 3/3A-G/image 3B vent1.tif]

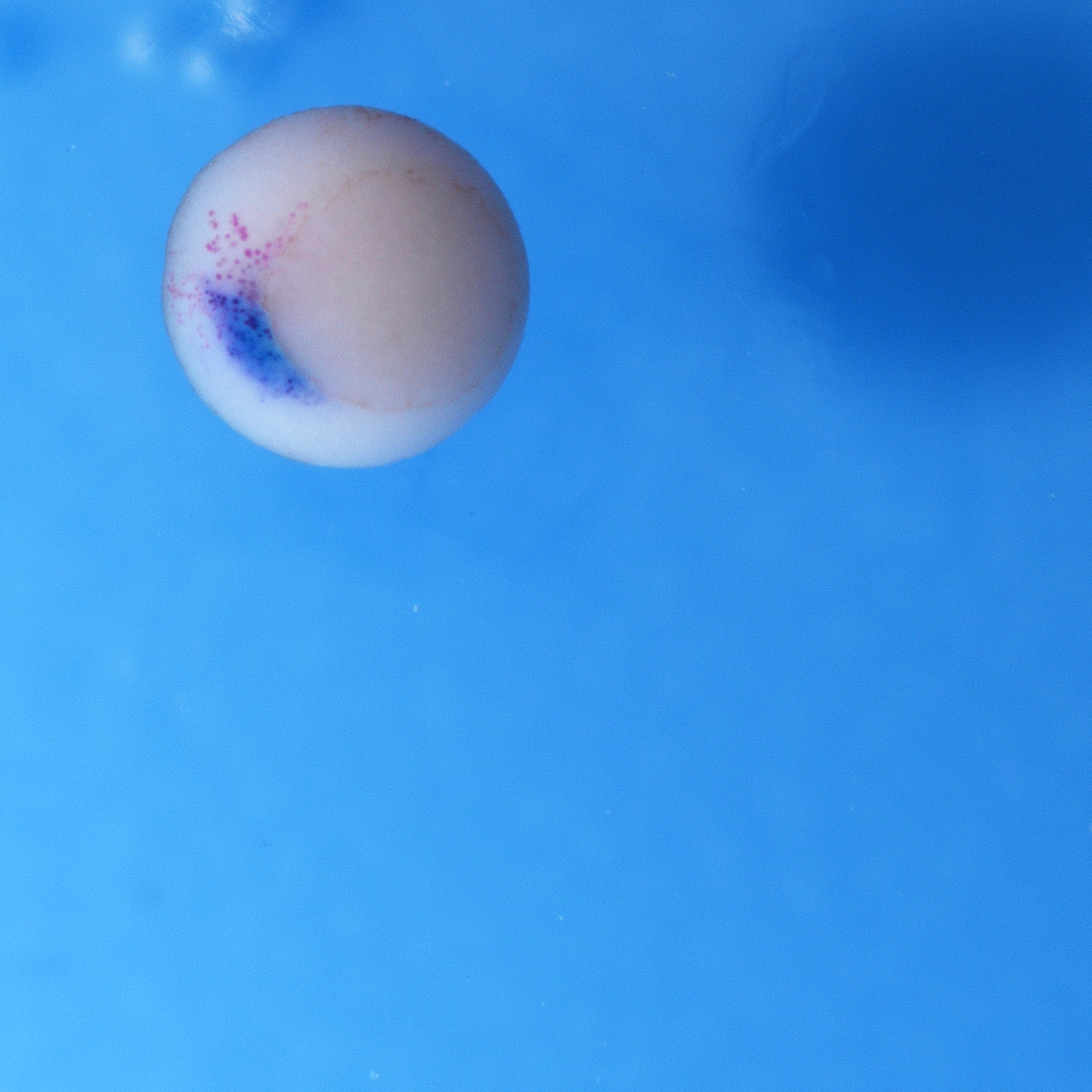

Supplement: Supplementary file 4 — Source Data Fig. 3 [file 44319_2023_46_MOESM4_ESM.zip › Figure 3/3A-G/image 3G chordin.tif]

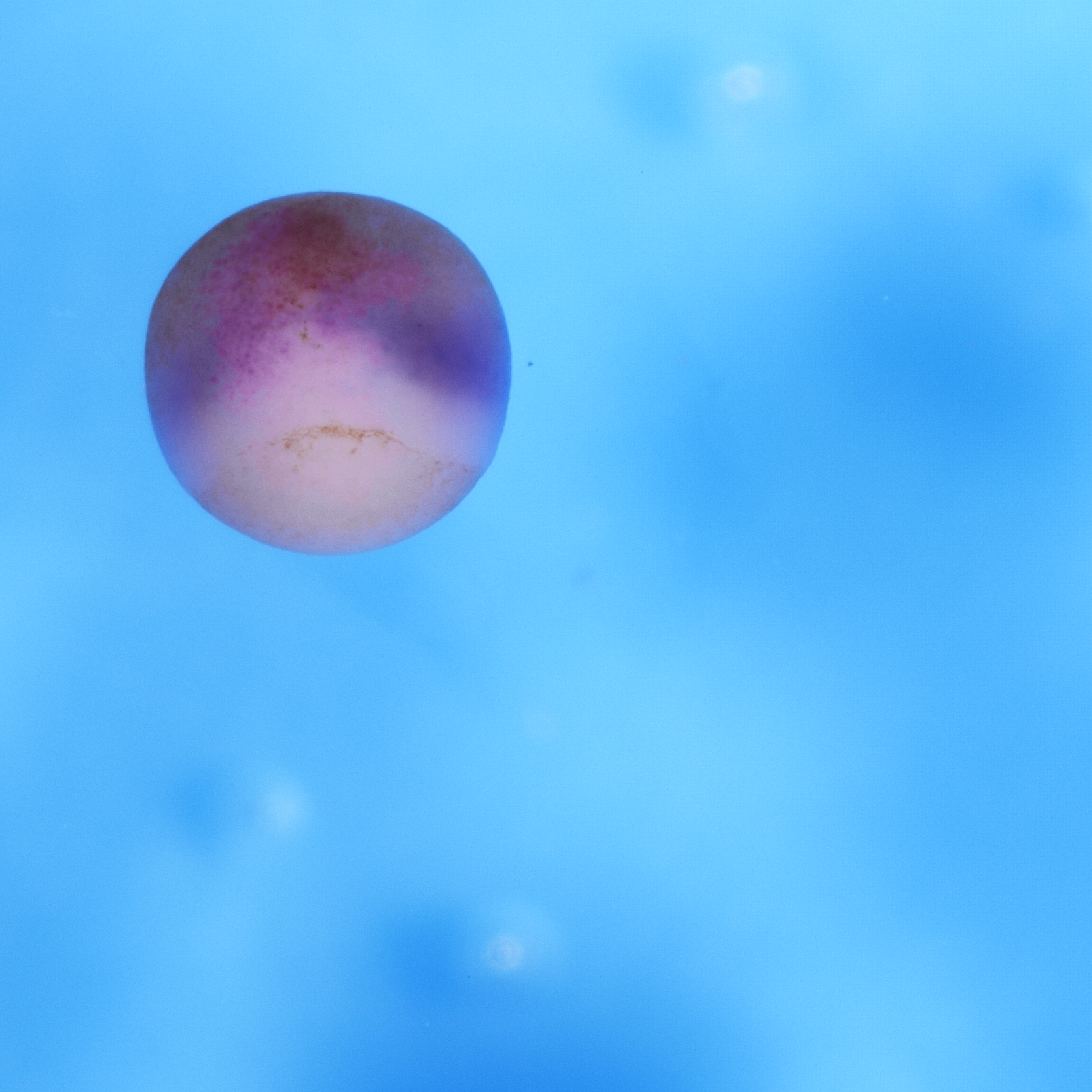

Supplement: Supplementary file 4 — Source Data Fig. 3 [file 44319_2023_46_MOESM4_ESM.zip › Figure 3/3A-G/image 3E sox2.tif]

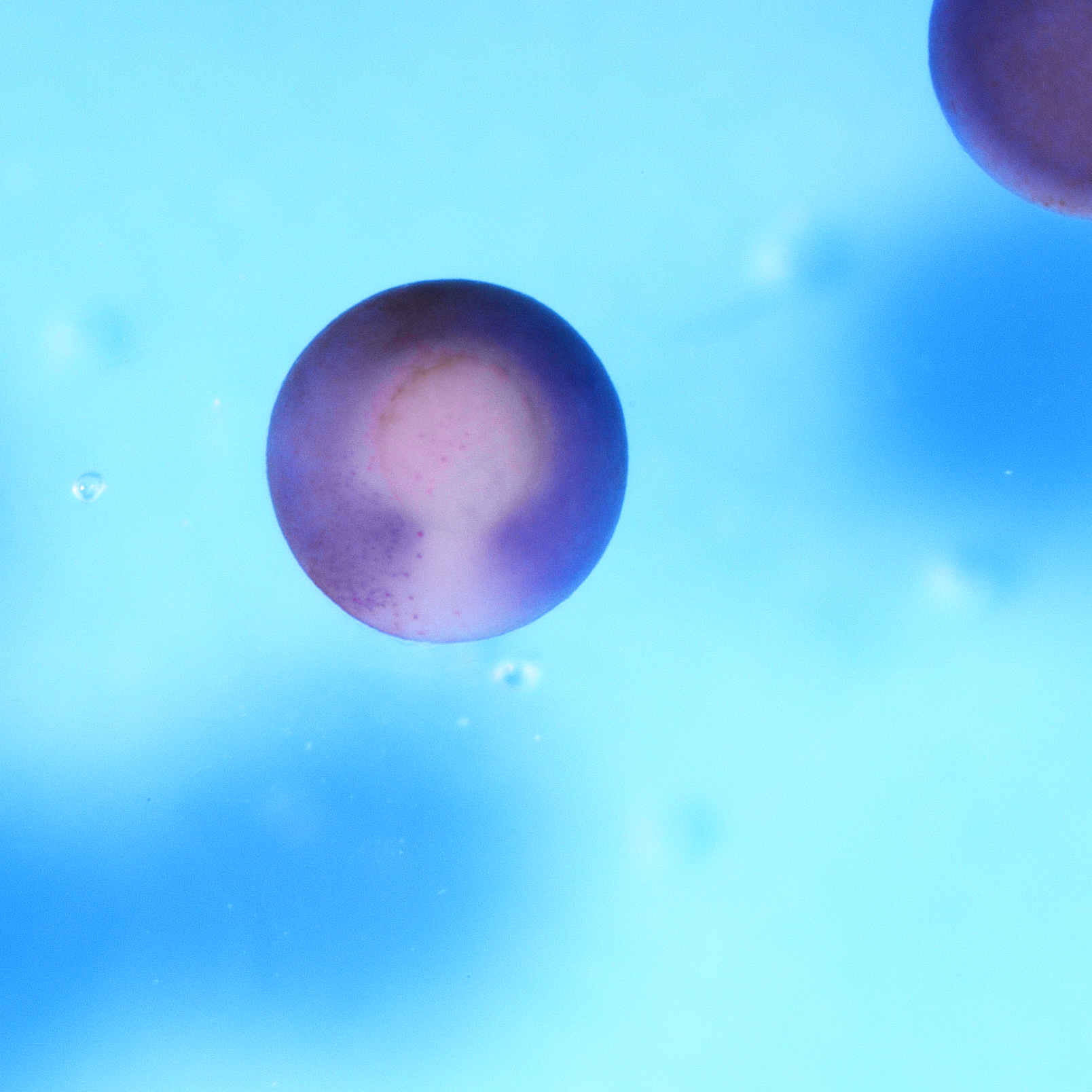

Supplement: Supplementary file 4 — Source Data Fig. 3 [file 44319_2023_46_MOESM4_ESM.zip › Figure 3/3A-G/image 3C vent2.tif]

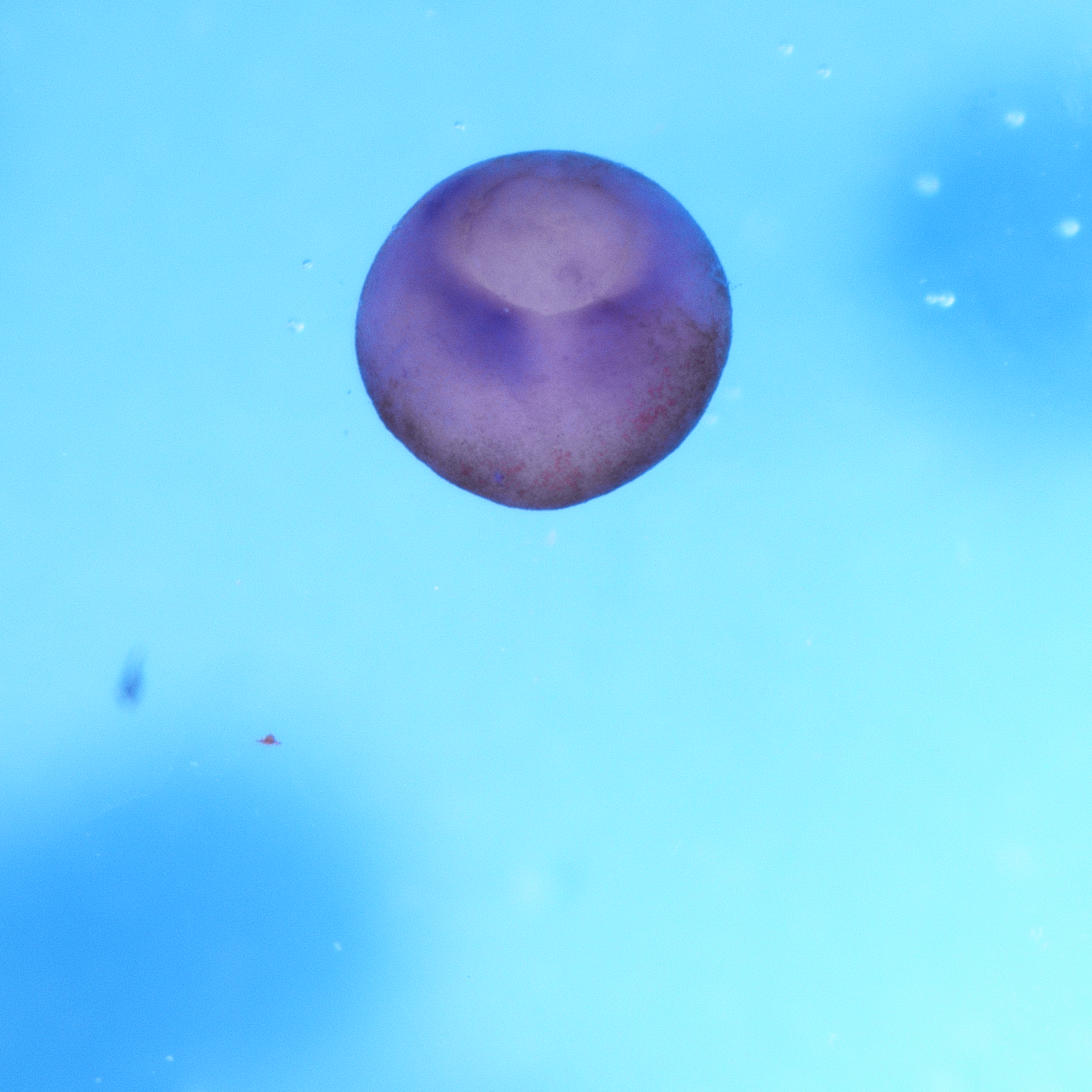

Supplement: Supplementary file 4 — Source Data Fig. 3 [file 44319_2023_46_MOESM4_ESM.zip › Figure 3/3A-G/image 3D myod.tif]

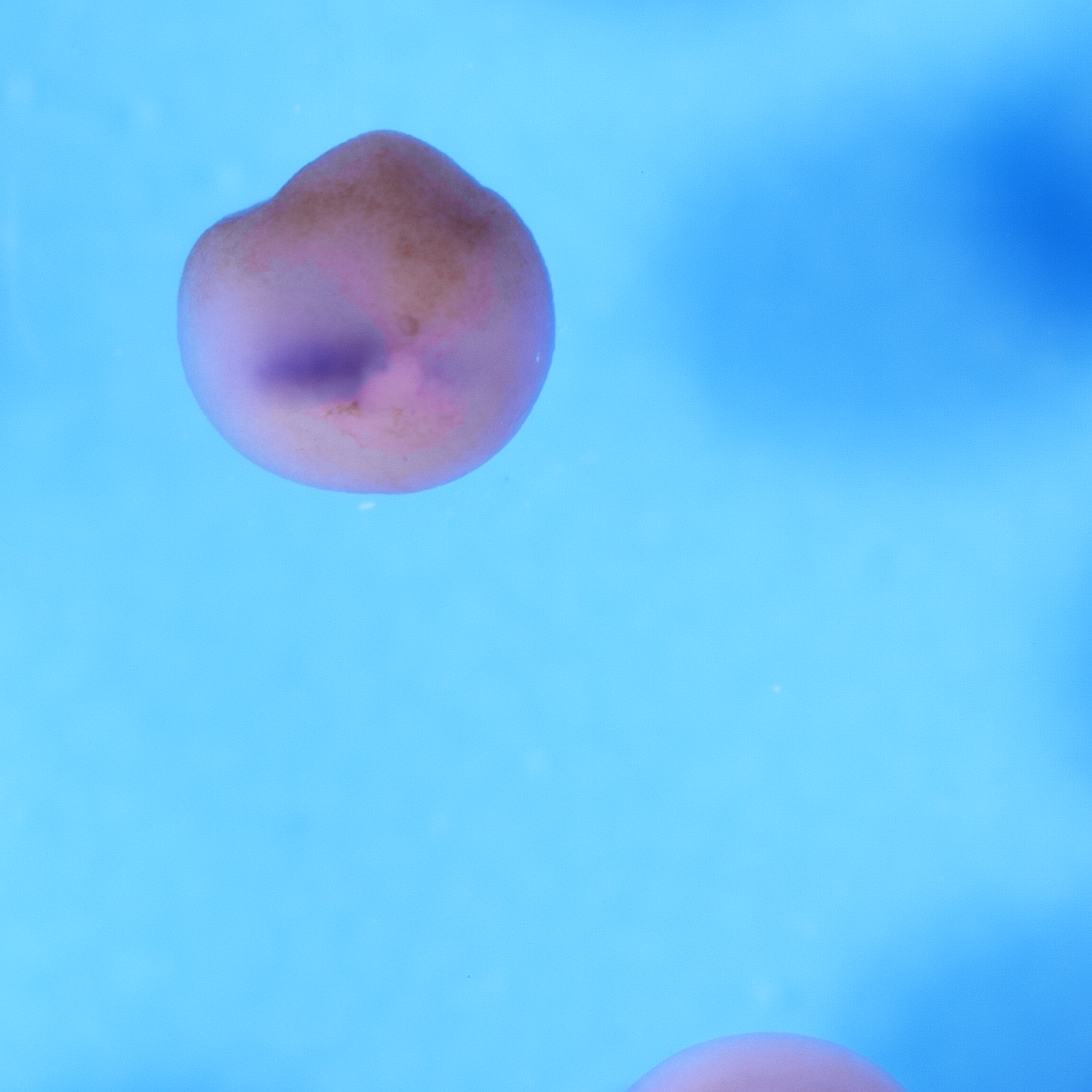

Supplement: Supplementary file 4 — Source Data Fig. 3 [file 44319_2023_46_MOESM4_ESM.zip › Figure 3/3A-G/image 3F gsc.tif]

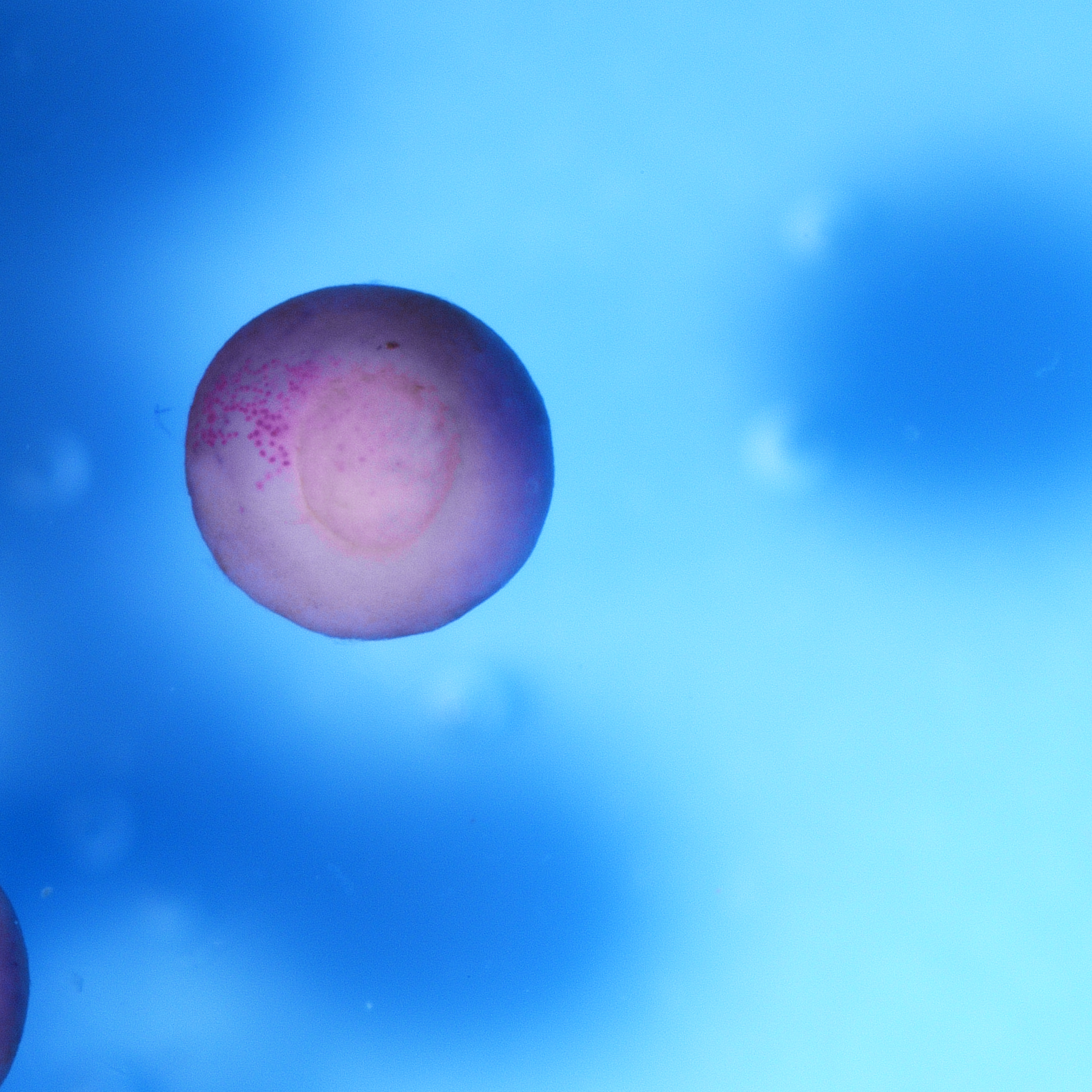

Supplement: Supplementary file 4 — Source Data Fig. 3 [file 44319_2023_46_MOESM4_ESM.zip › Figure 3/3A-G/image 3A sizzled.tif]

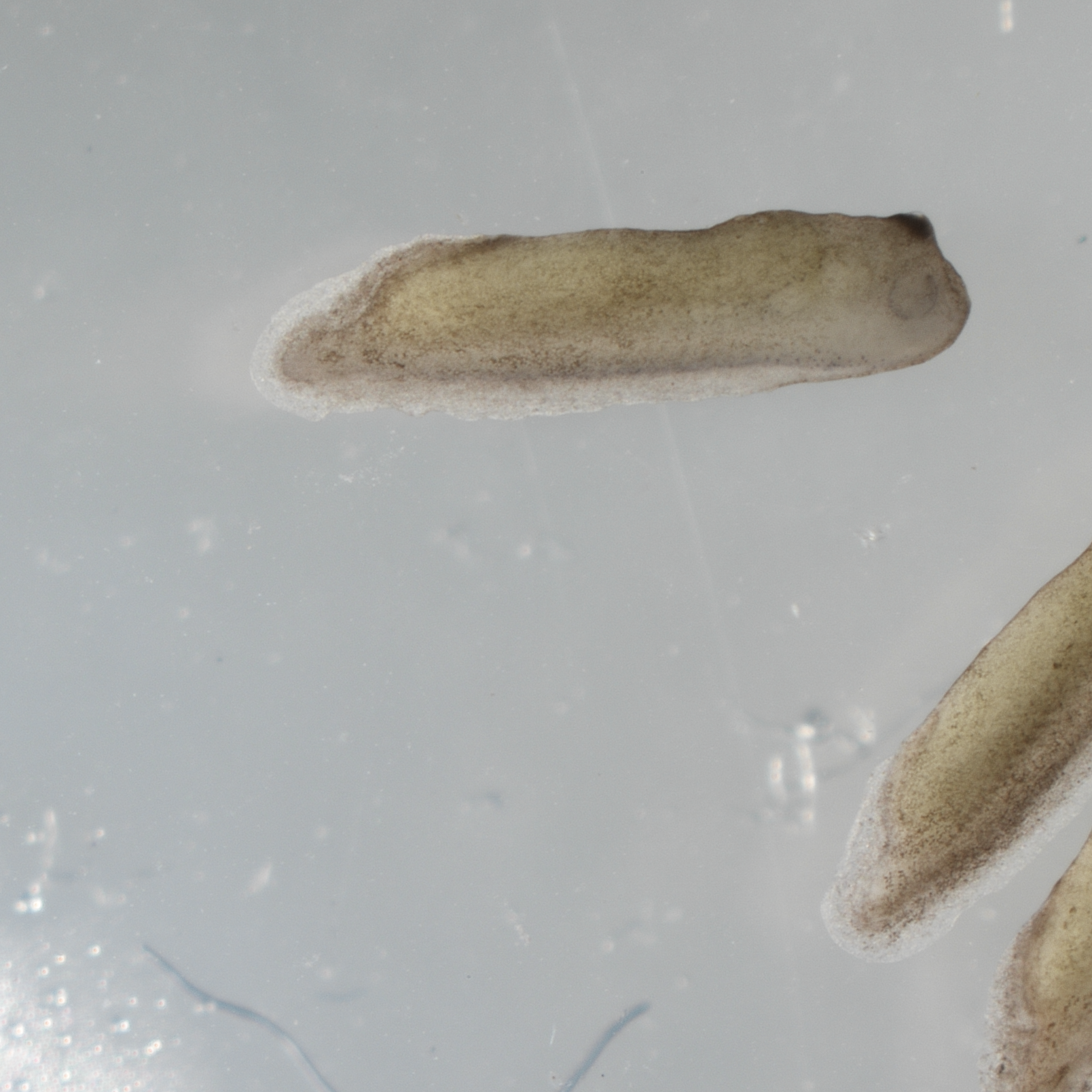

Supplement: Supplementary file 4 — Source Data Fig. 3 [file 44319_2023_46_MOESM4_ESM.zip › Figure 3/3M/image 3M normal.tif]

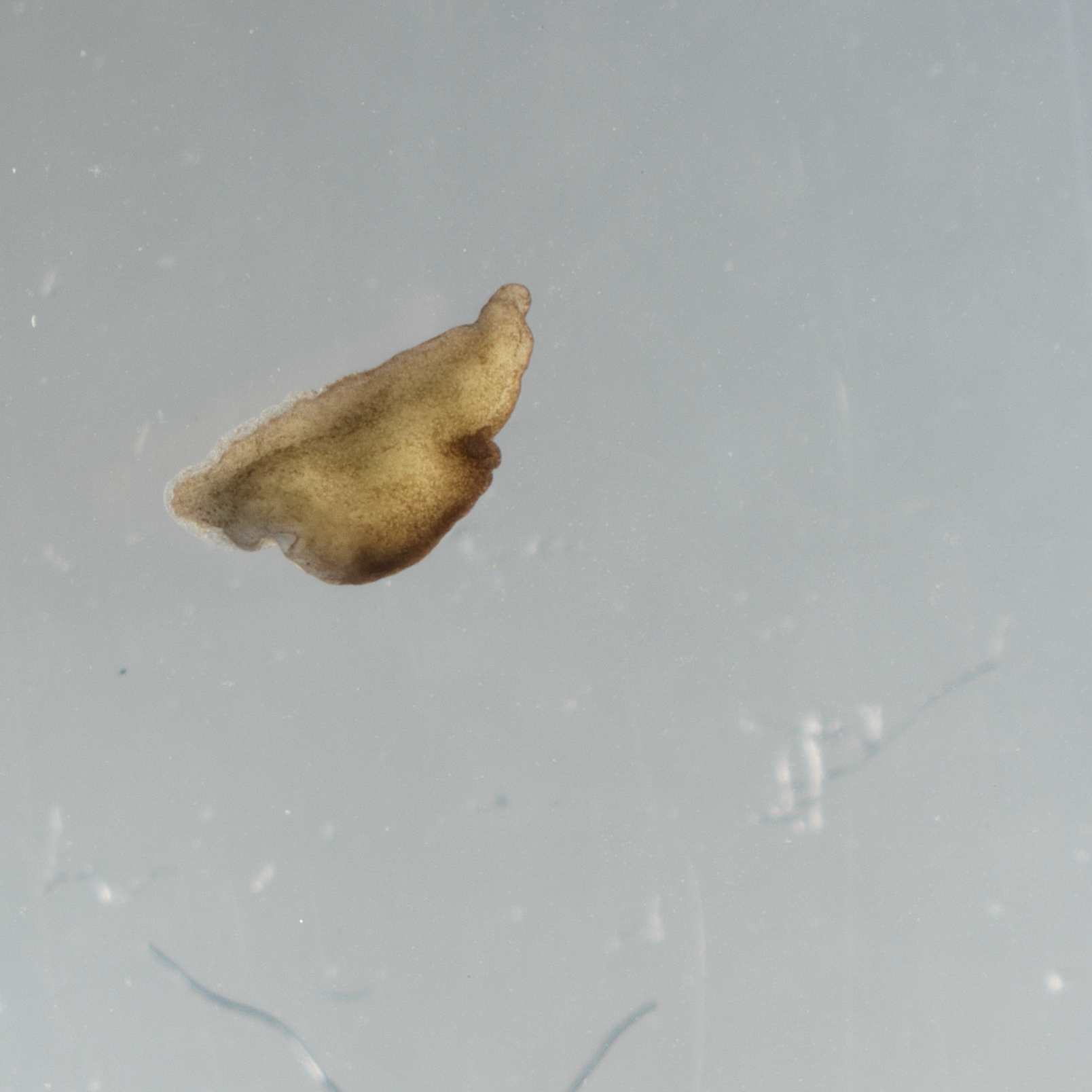

Supplement: Supplementary file 4 — Source Data Fig. 3 [file 44319_2023_46_MOESM4_ESM.zip › Figure 3/3M/image 3M severe.tif]

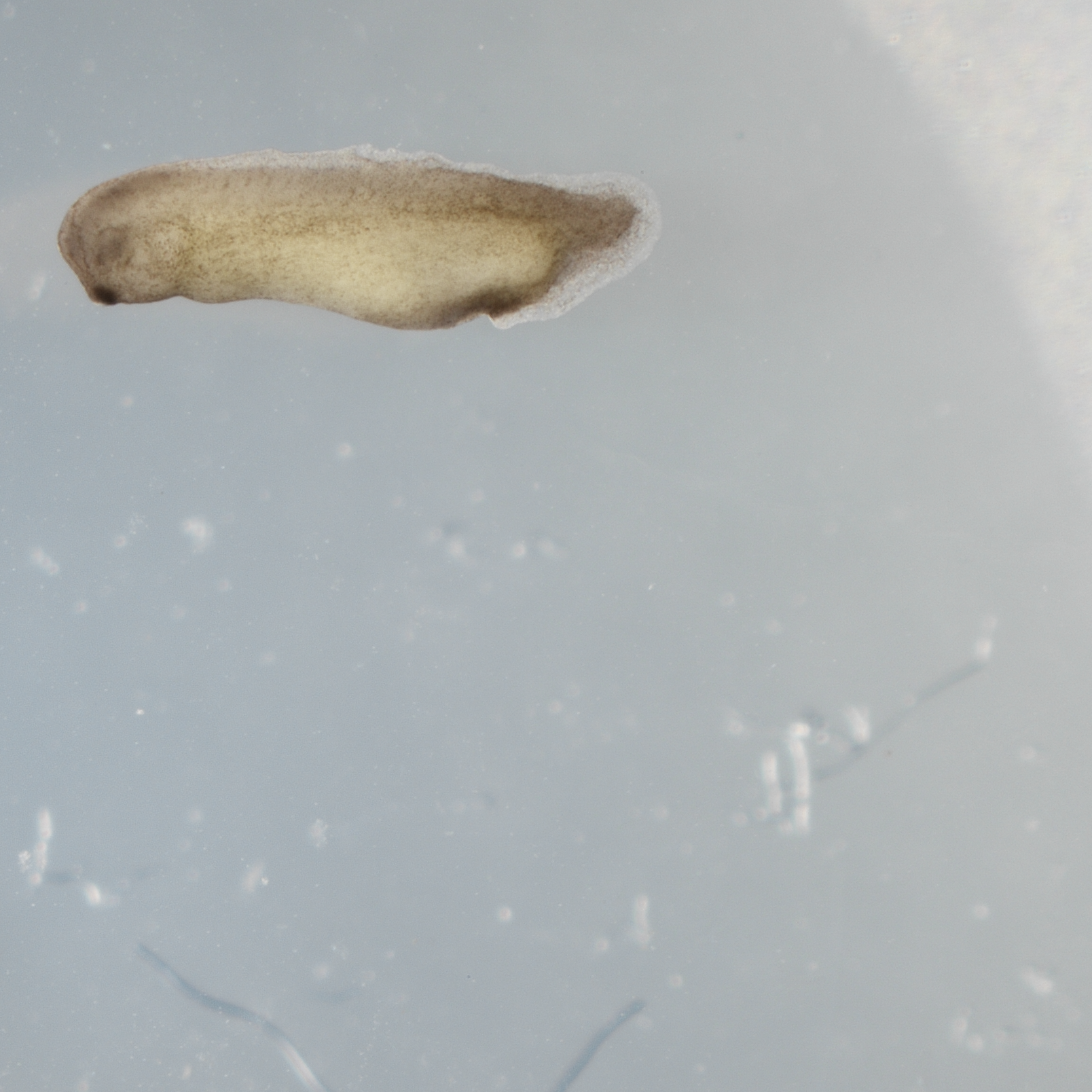

Supplement: Supplementary file 4 — Source Data Fig. 3 [file 44319_2023_46_MOESM4_ESM.zip › Figure 3/3M/image 3M mild.tif]

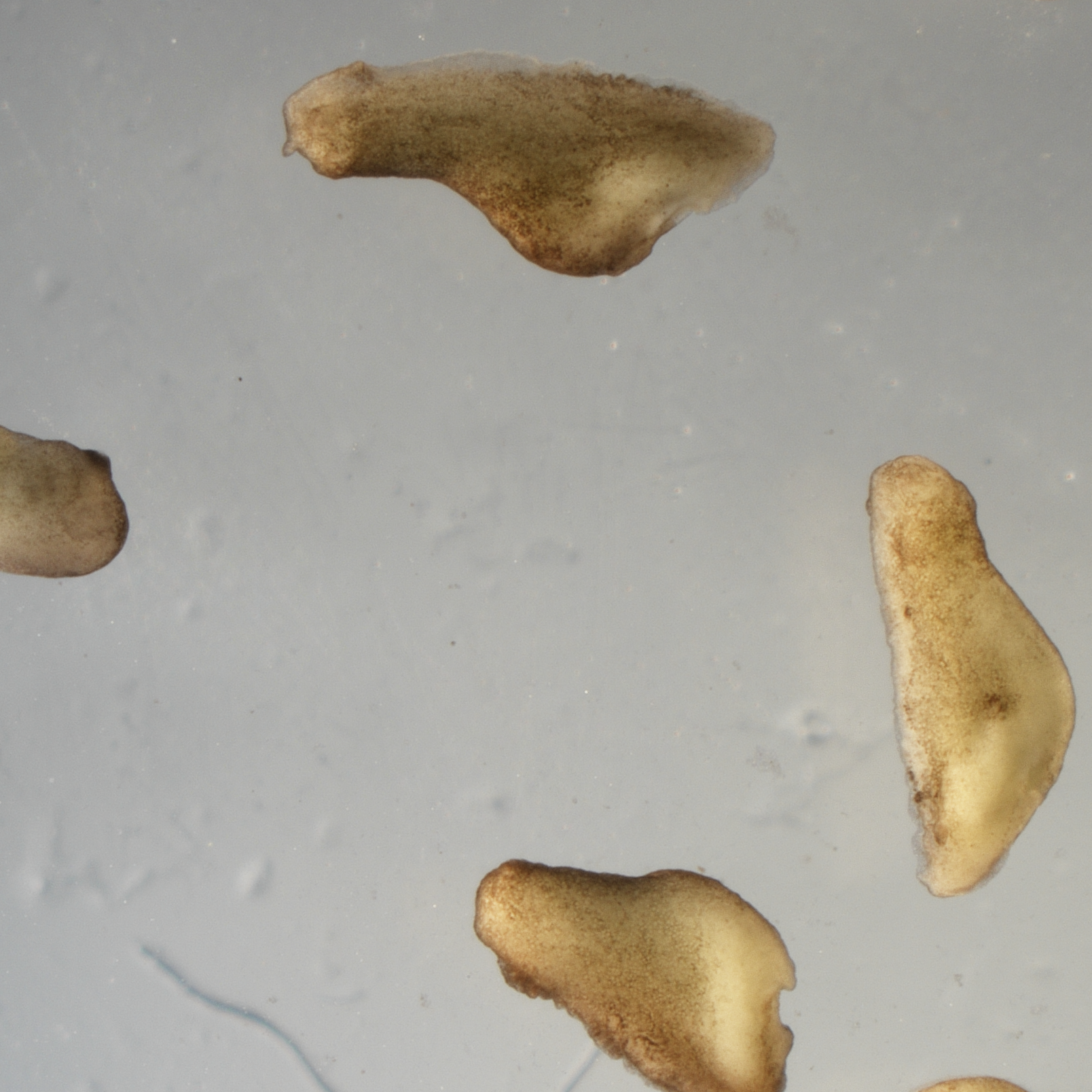

Supplement: Supplementary file 4 — Source Data Fig. 3 [file 44319_2023_46_MOESM4_ESM.zip › Figure 3/3M/image 3M moderate.tif]

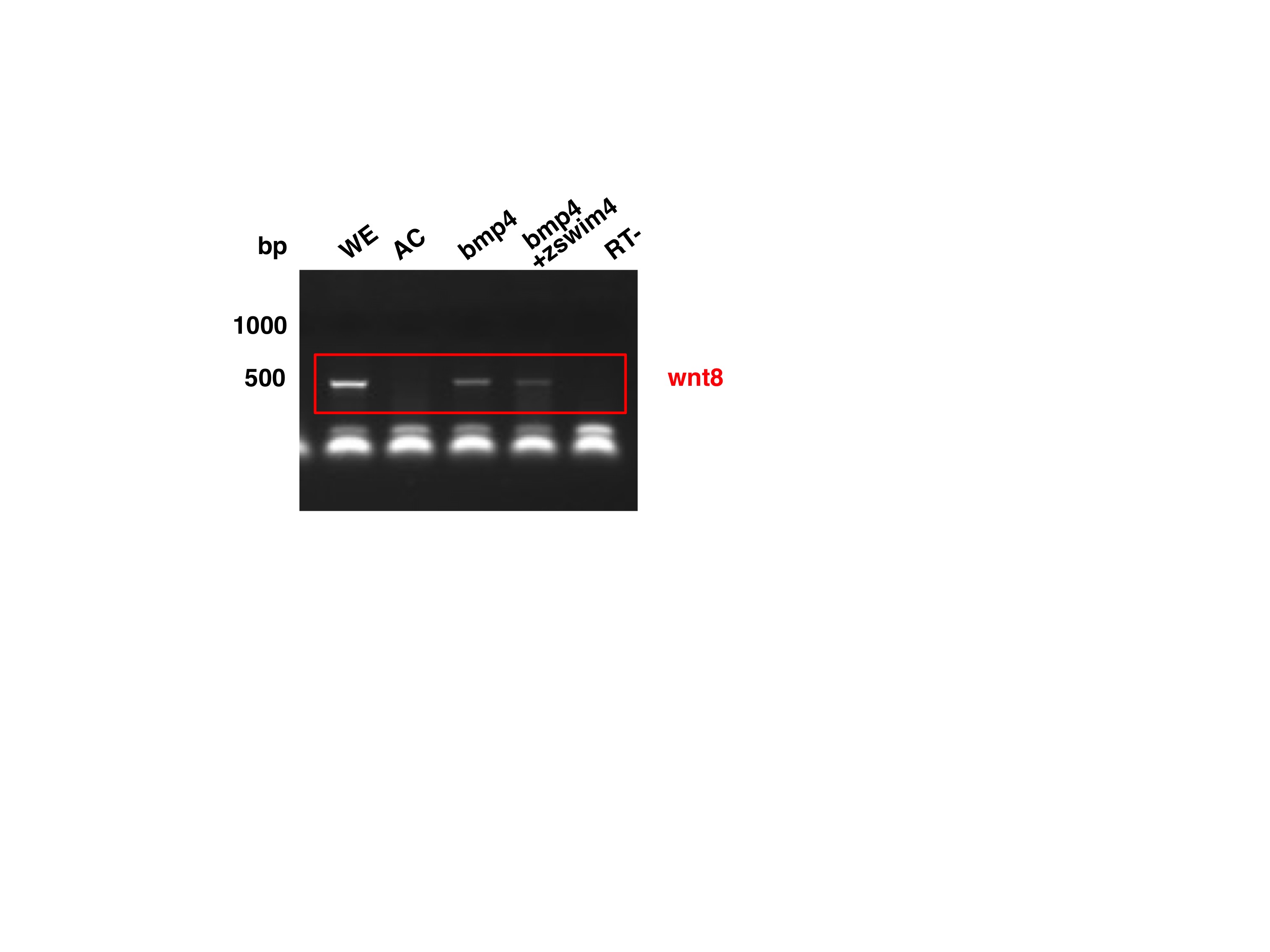

Supplement: Supplementary file 4 — Source Data Fig. 3 [file 44319_2023_46_MOESM4_ESM.zip › Figure 3/3J/DNA gel 3J wnt8.jpg]

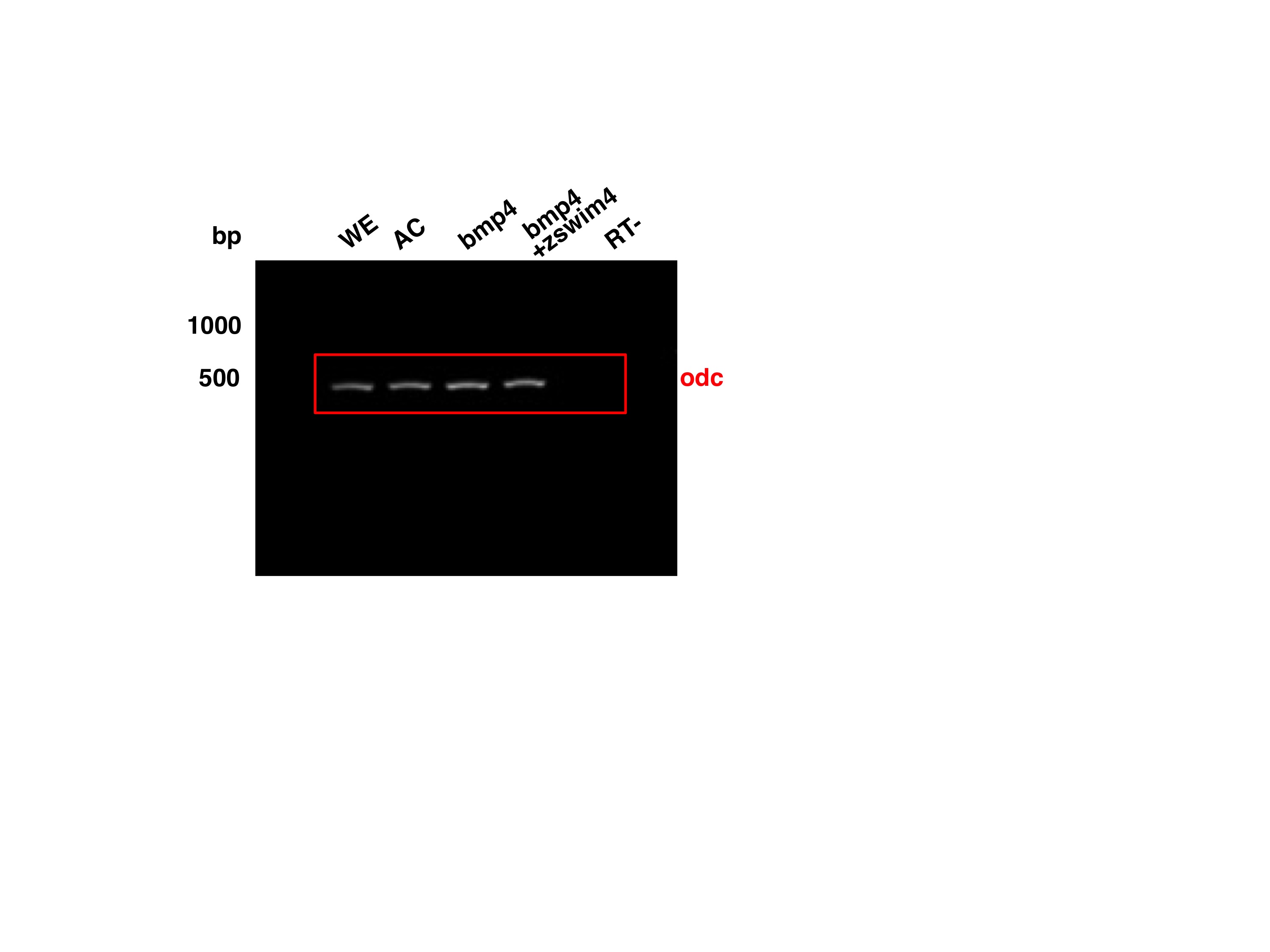

Supplement: Supplementary file 4 — Source Data Fig. 3 [file 44319_2023_46_MOESM4_ESM.zip › Figure 3/3J/DNA gel 3J odc.jpg]

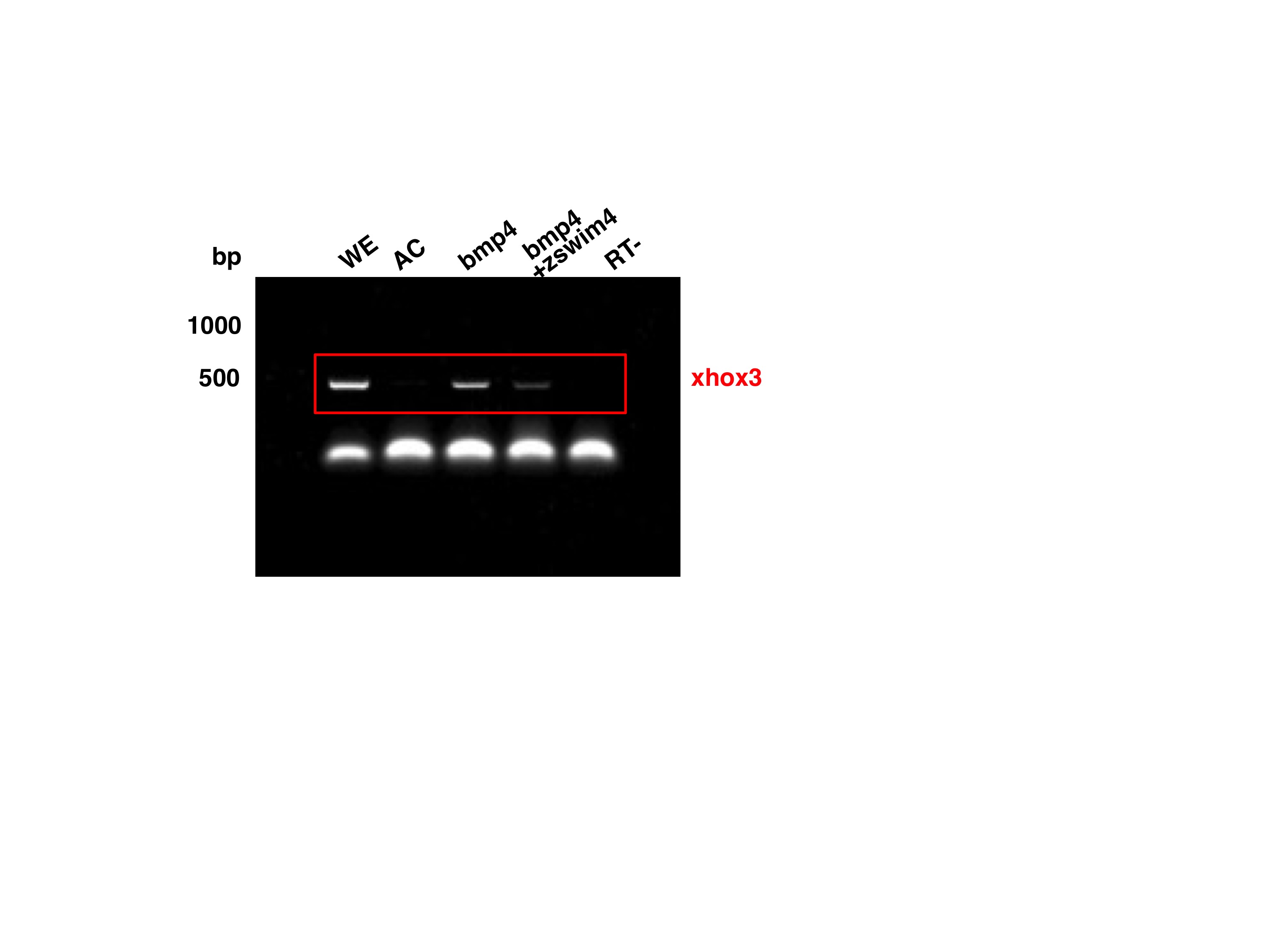

Supplement: Supplementary file 4 — Source Data Fig. 3 [file 44319_2023_46_MOESM4_ESM.zip › Figure 3/3J/DNA gel 3J xhox3.jpg]

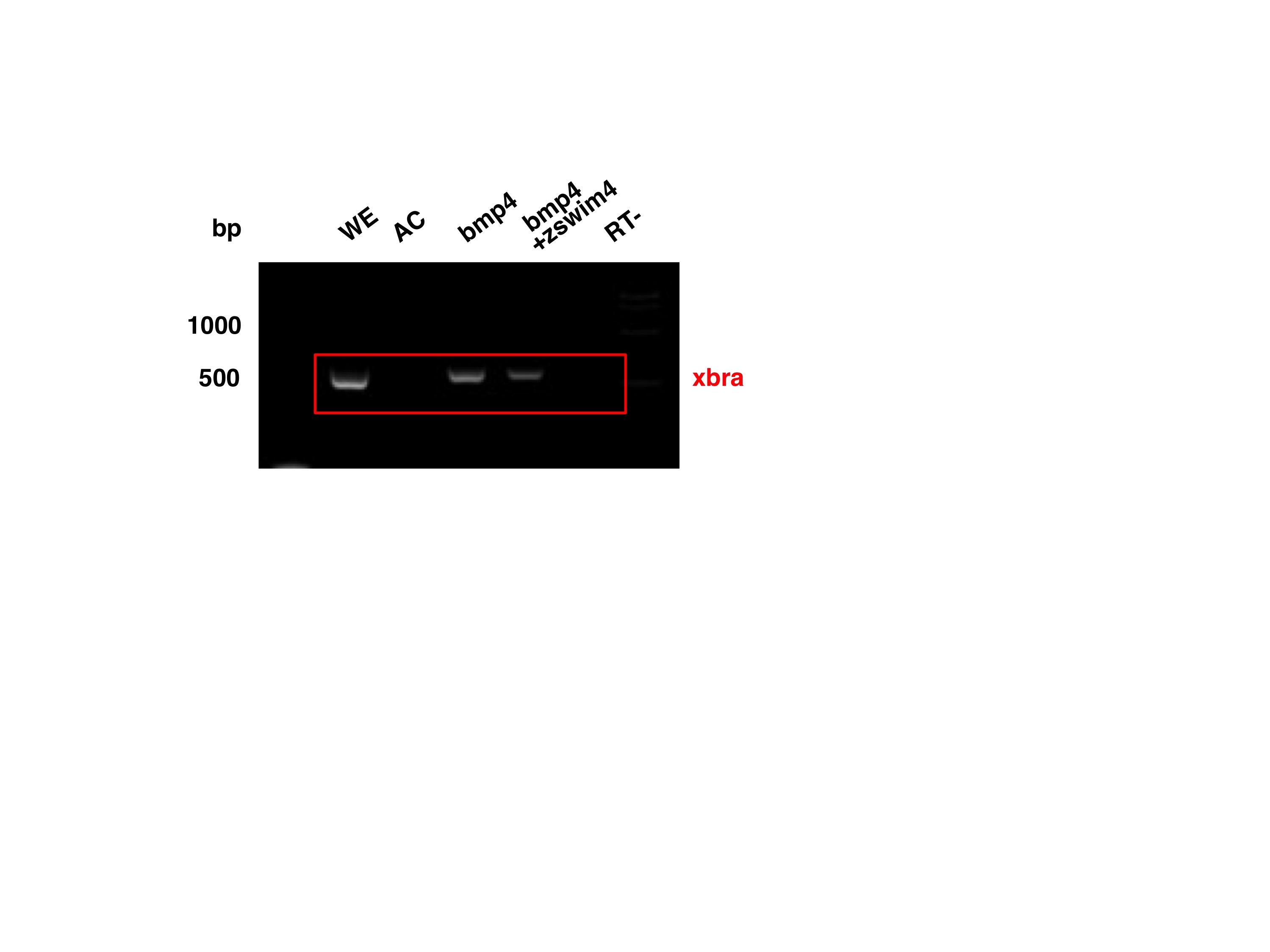

Supplement: Supplementary file 4 — Source Data Fig. 3 [file 44319_2023_46_MOESM4_ESM.zip › Figure 3/3J/DNA gel 3J xbra.jpg]

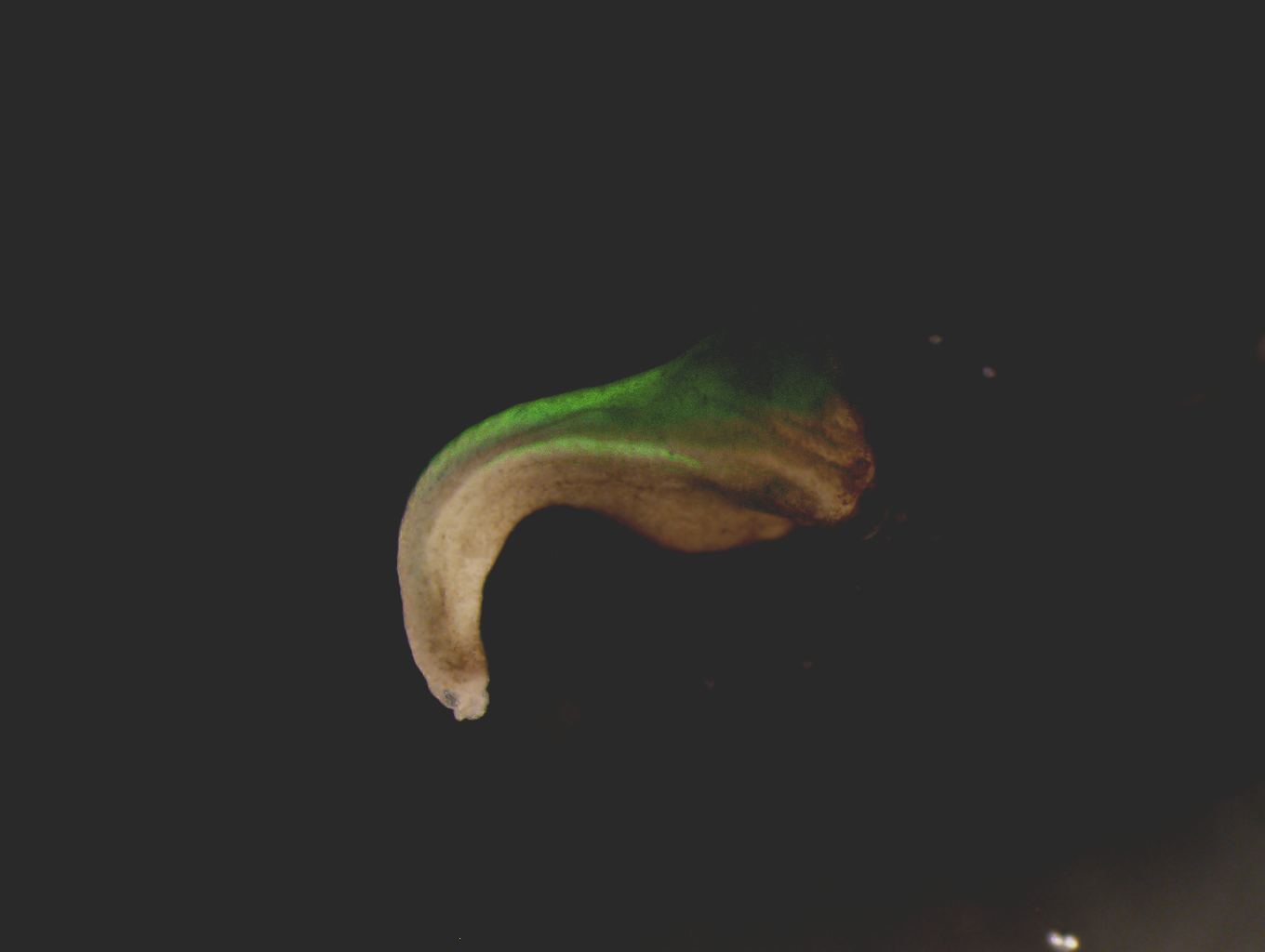

Supplement: Supplementary file 4 — Source Data Fig. 3 [file 44319_2023_46_MOESM4_ESM.zip › Figure 3/3Q/image 3Q dual-axis merge.tif]

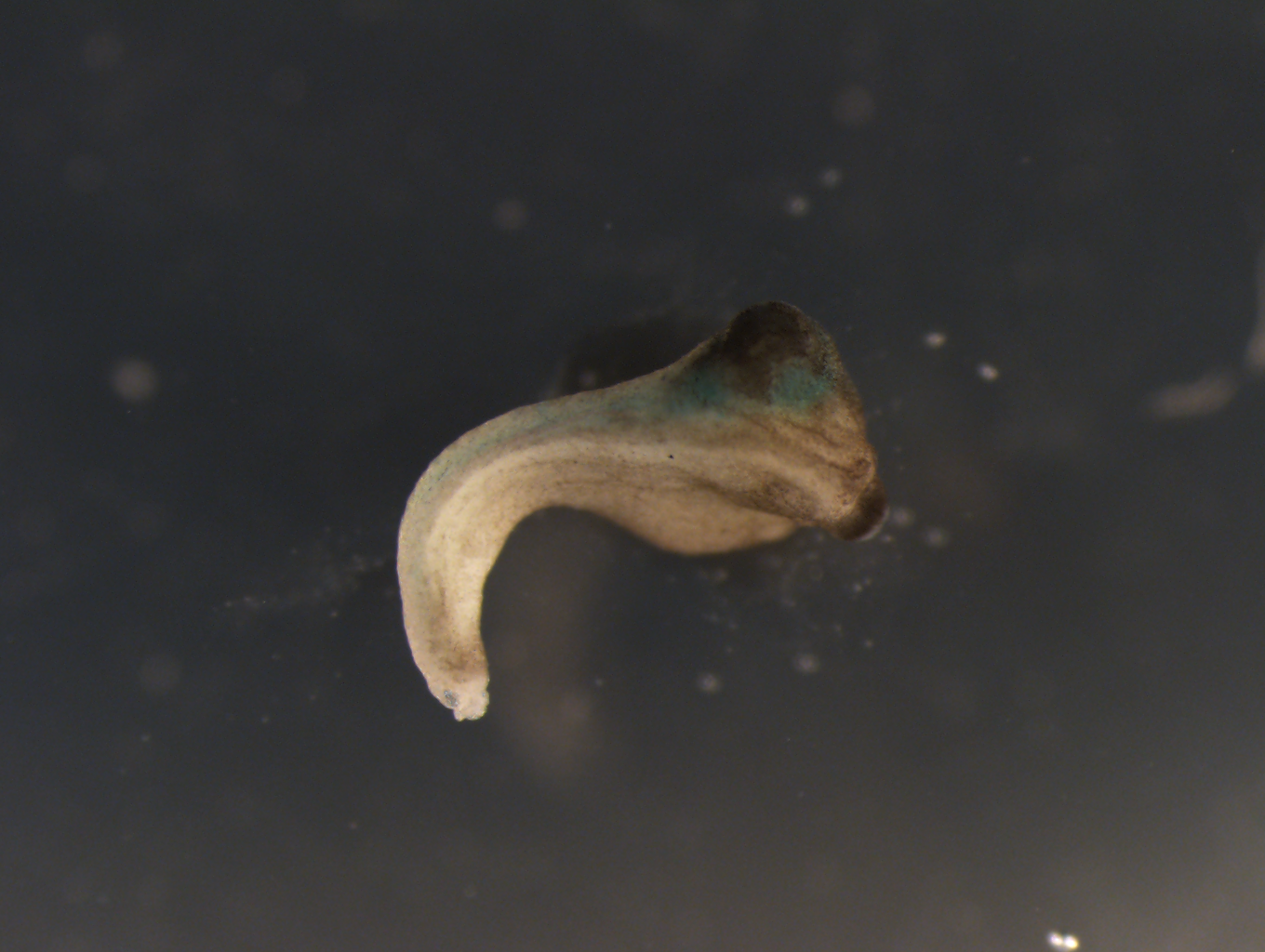

Supplement: Supplementary file 4 — Source Data Fig. 3 [file 44319_2023_46_MOESM4_ESM.zip › Figure 3/3Q/image 3Q dual-axis.tif]

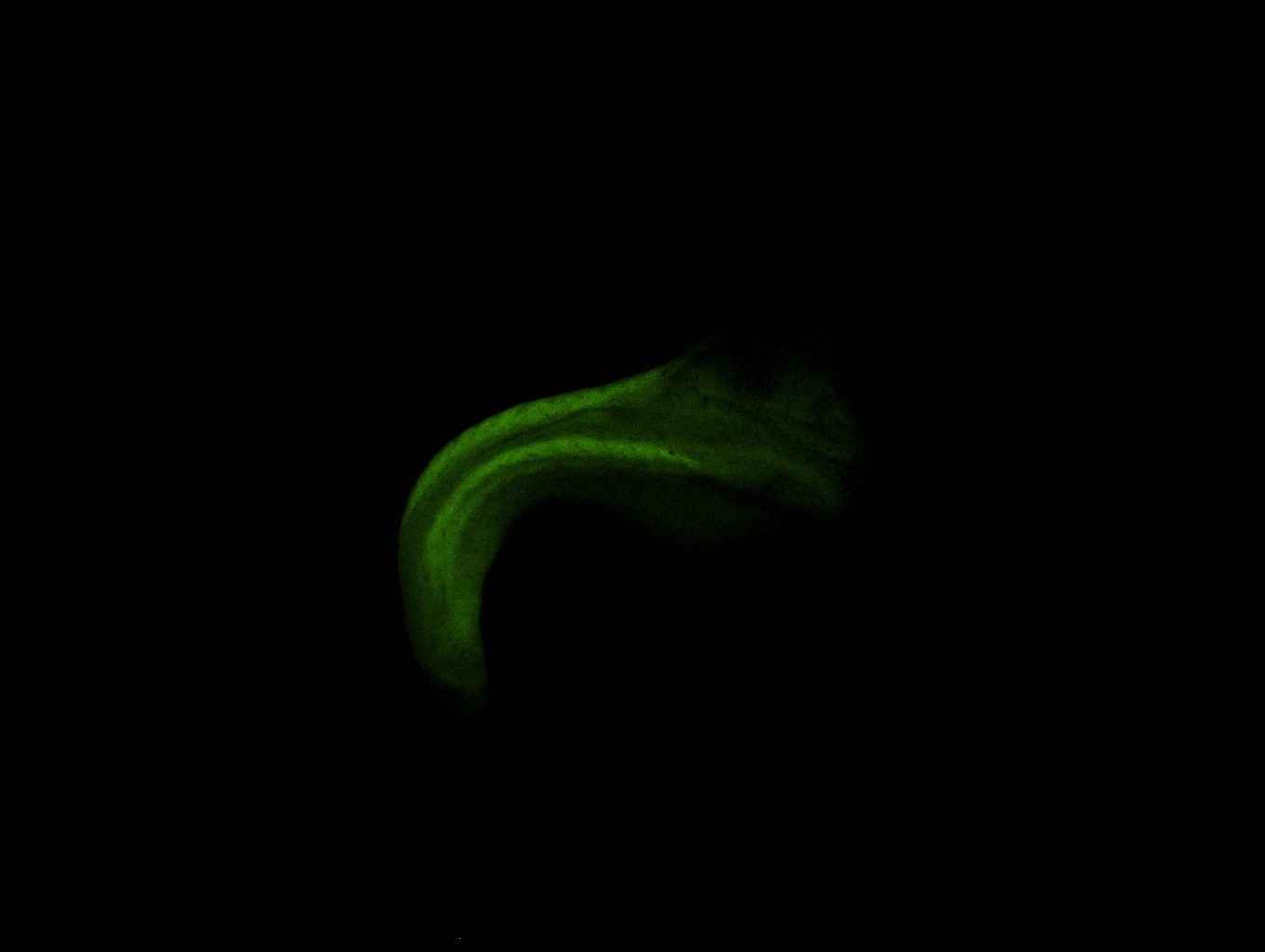

Supplement: Supplementary file 4 — Source Data Fig. 3 [file 44319_2023_46_MOESM4_ESM.zip › Figure 3/3Q/image 3Q dual-axis staining.tif]

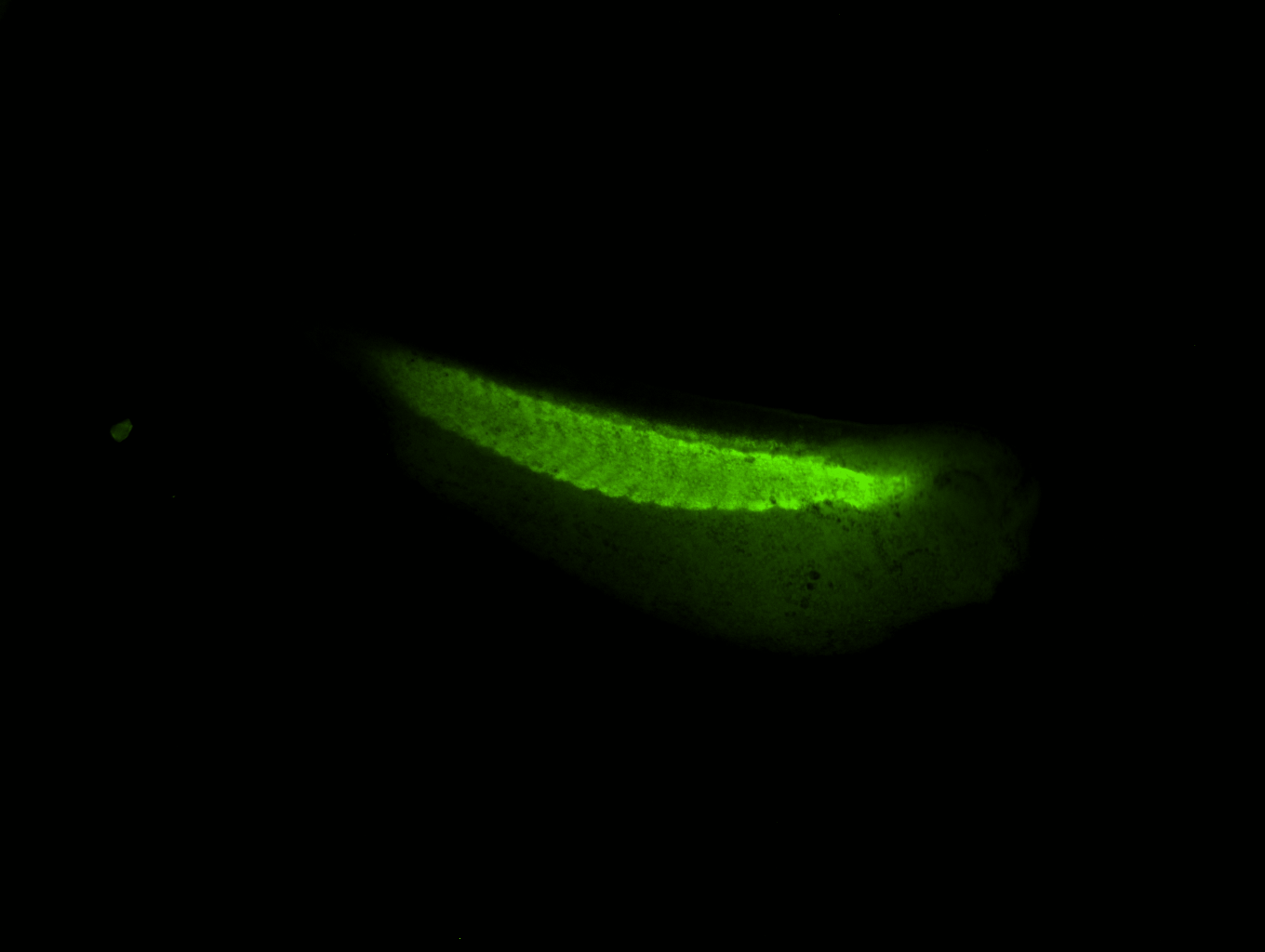

Supplement: Supplementary file 4 — Source Data Fig. 3 [file 44319_2023_46_MOESM4_ESM.zip › Figure 3/3Q/image 3Q one-axis staining.tif]

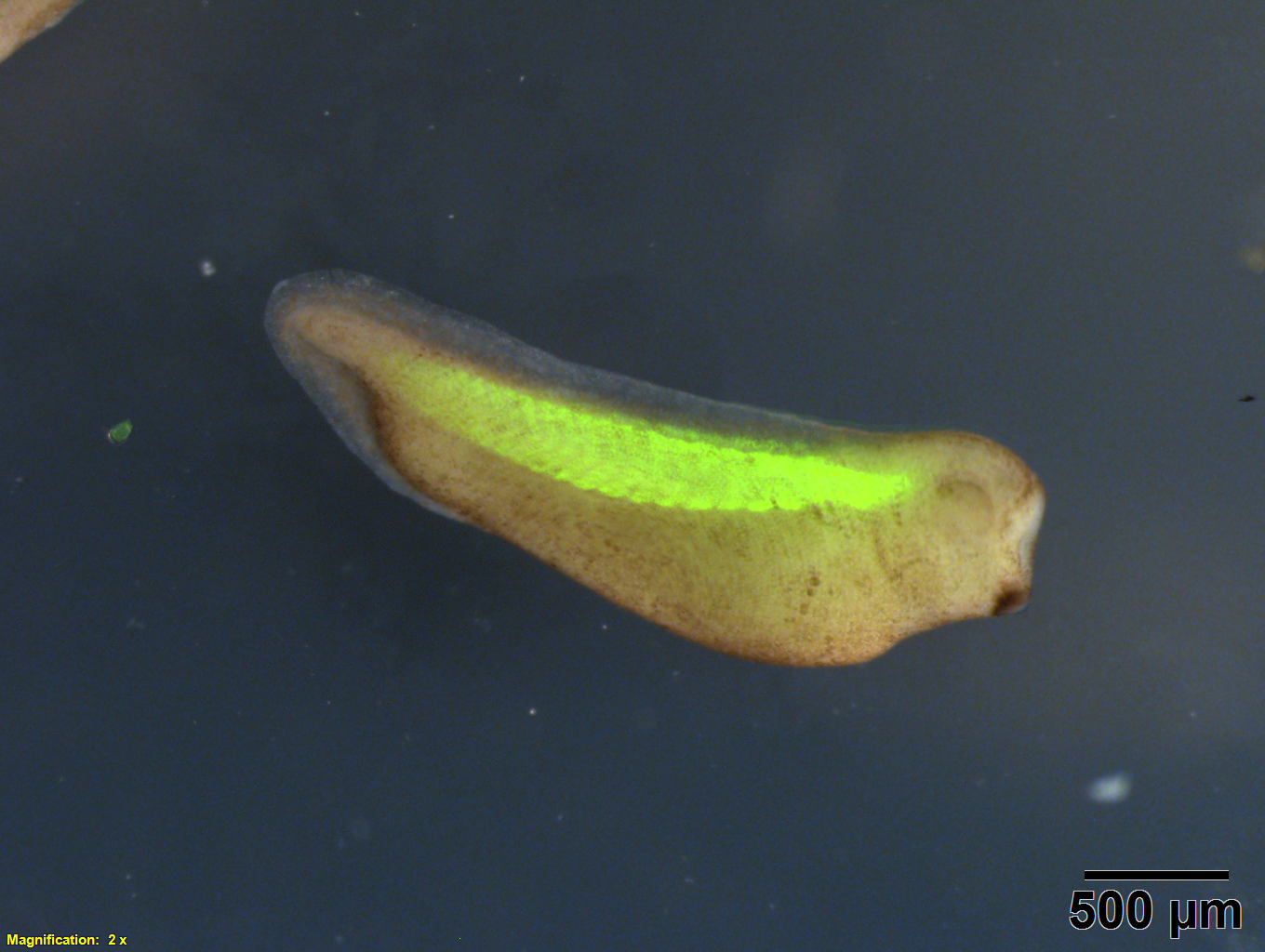

Supplement: Supplementary file 4 — Source Data Fig. 3 [file 44319_2023_46_MOESM4_ESM.zip › Figure 3/3Q/image 3Q one-axis merge.tif]

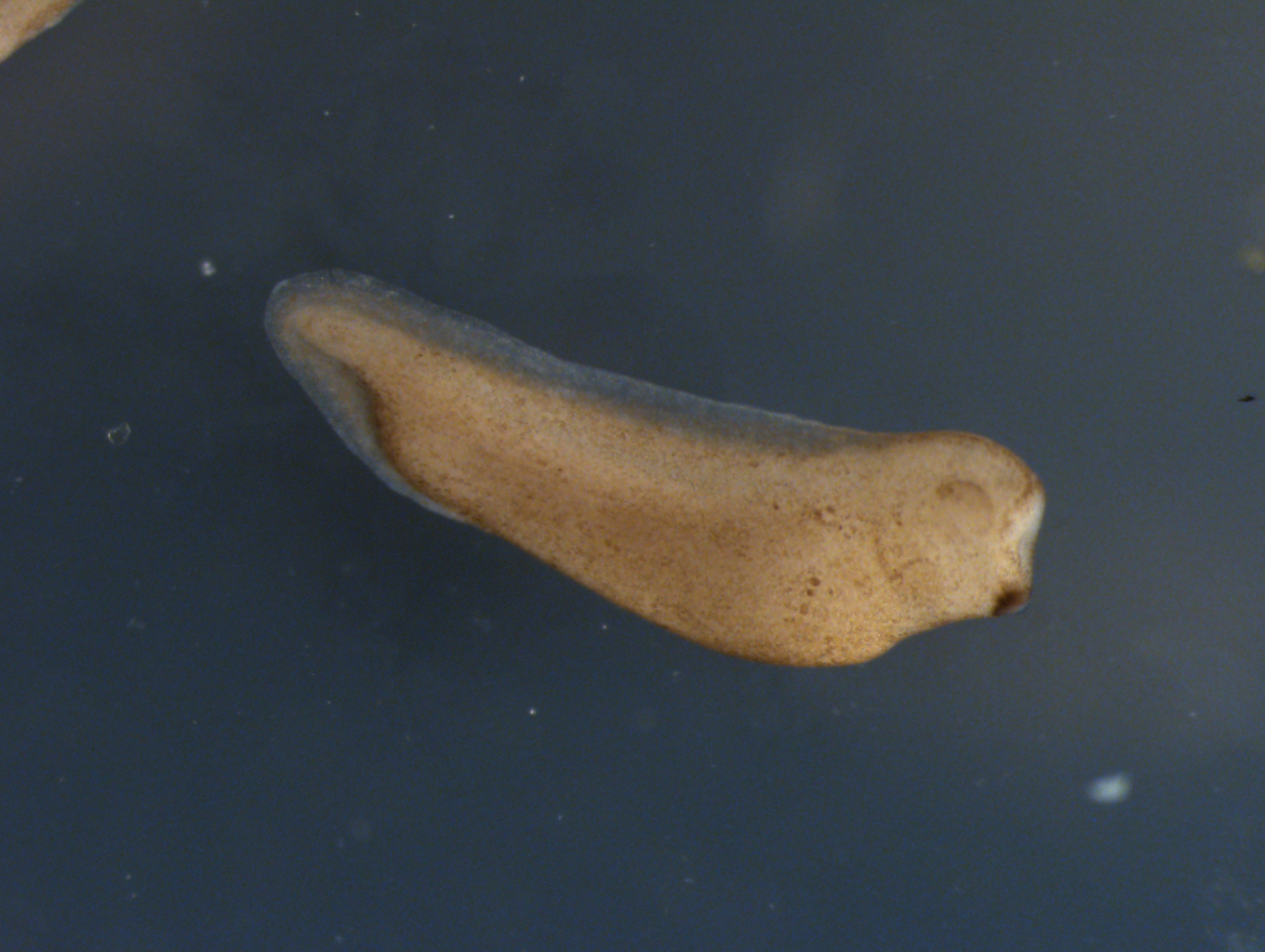

Supplement: Supplementary file 4 — Source Data Fig. 3 [file 44319_2023_46_MOESM4_ESM.zip › Figure 3/3Q/image 3Q one-axis.tif]

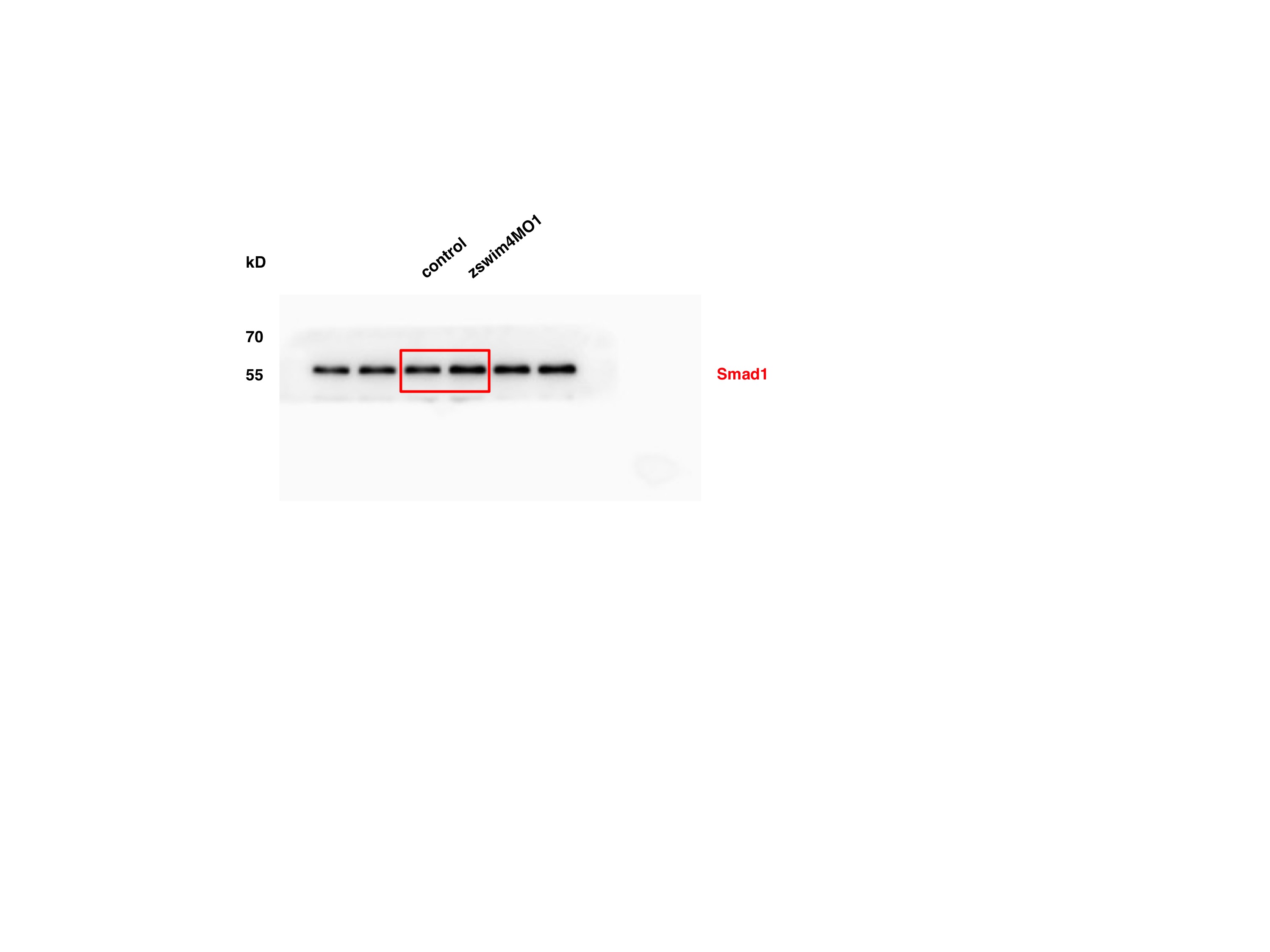

Supplement: Supplementary file 4 — Source Data Fig. 3 [file 44319_2023_46_MOESM4_ESM.zip › Figure 3/3T/western 3T Smad1.jpg]

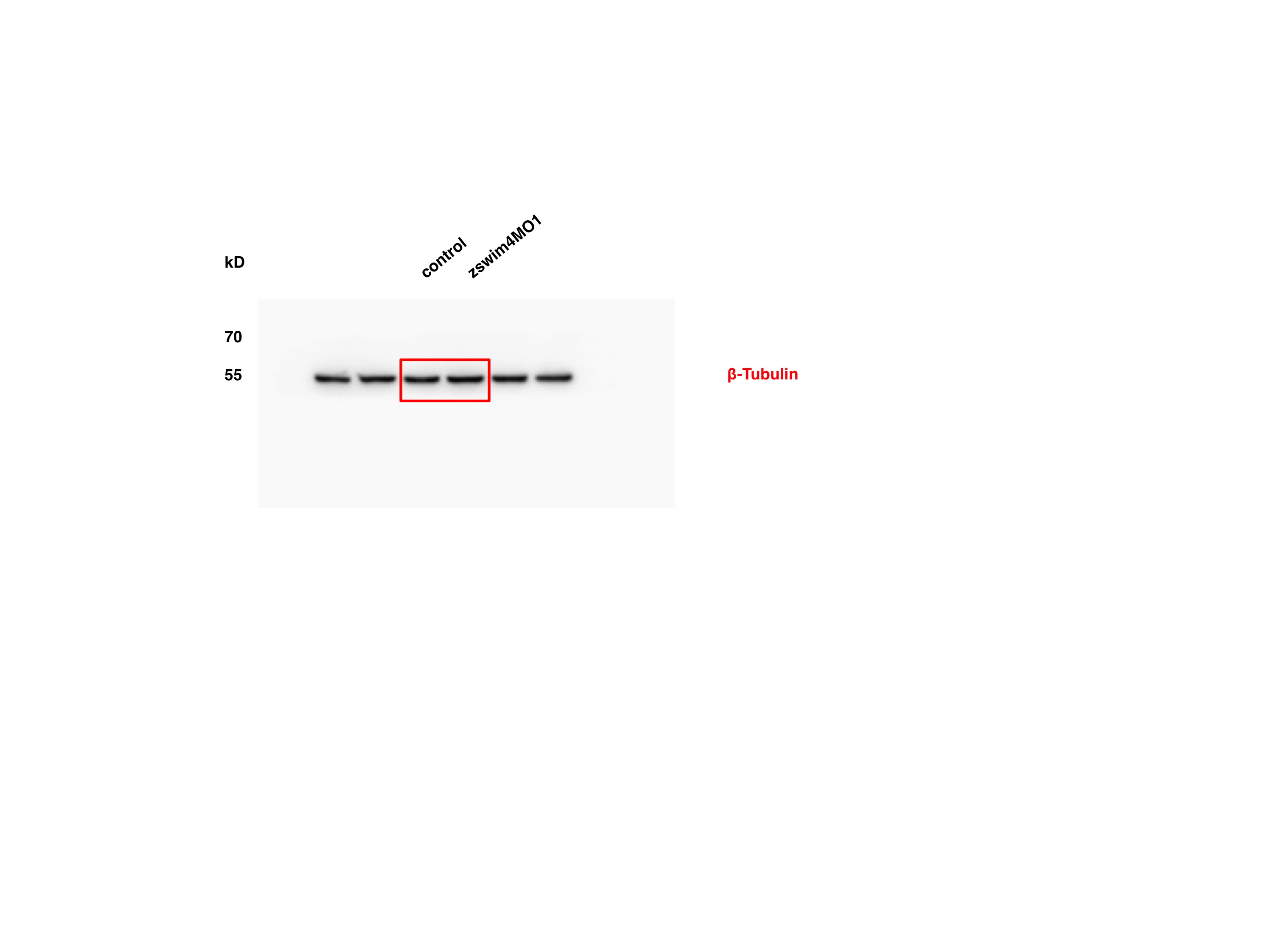

Supplement: Supplementary file 4 — Source Data Fig. 3 [file 44319_2023_46_MOESM4_ESM.zip › Figure 3/3T/western 3T tubulin.jpg]

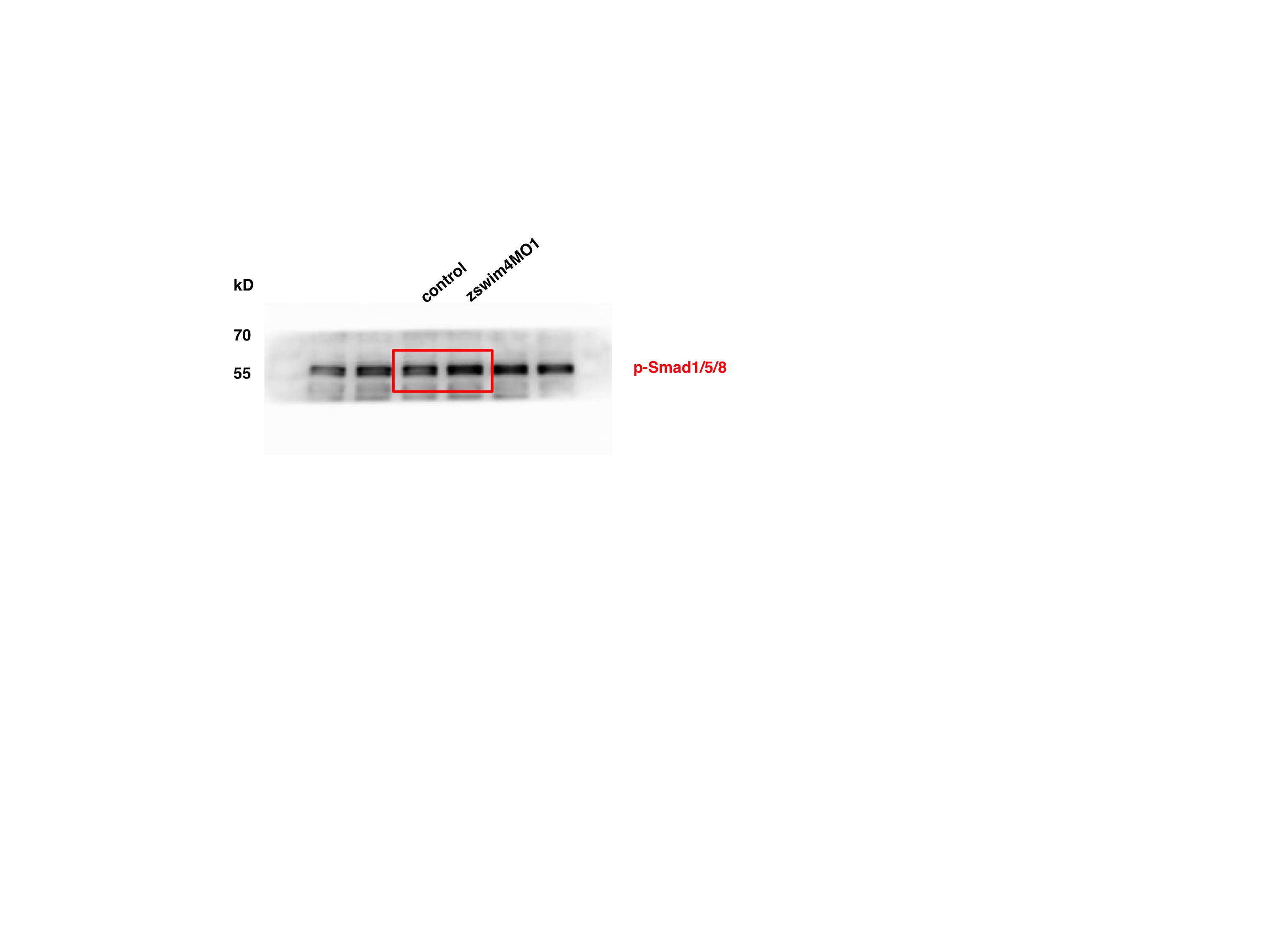

Supplement: Supplementary file 4 — Source Data Fig. 3 [file 44319_2023_46_MOESM4_ESM.zip › Figure 3/3T/western 3T p-Smad1.jpg]

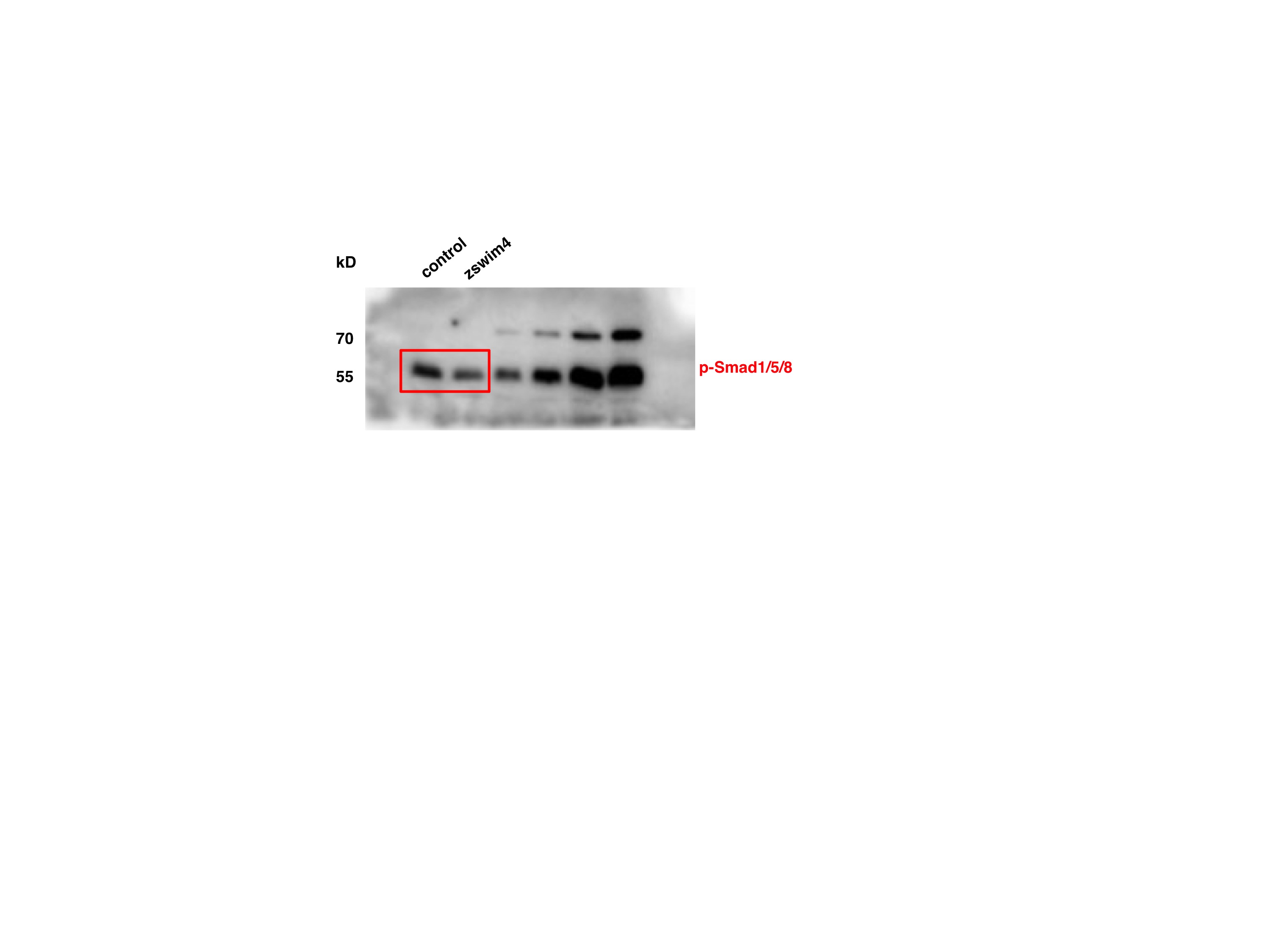

Supplement: Supplementary file 4 — Source Data Fig. 3 [file 44319_2023_46_MOESM4_ESM.zip › Figure 3/3S/western 3S p-SMAD1.jpg]

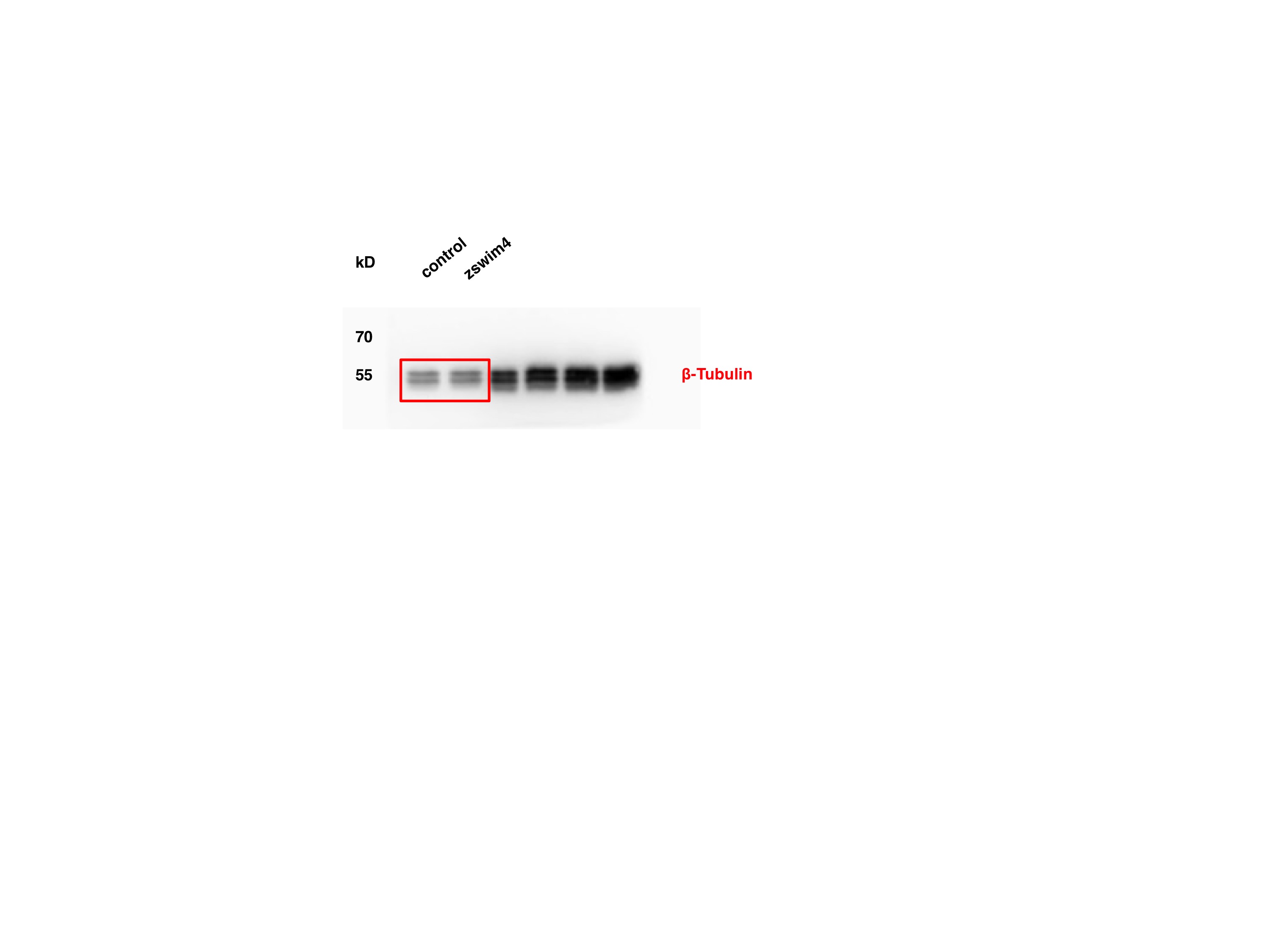

Supplement: Supplementary file 4 — Source Data Fig. 3 [file 44319_2023_46_MOESM4_ESM.zip › Figure 3/3S/western 3S tubulin.jpg]

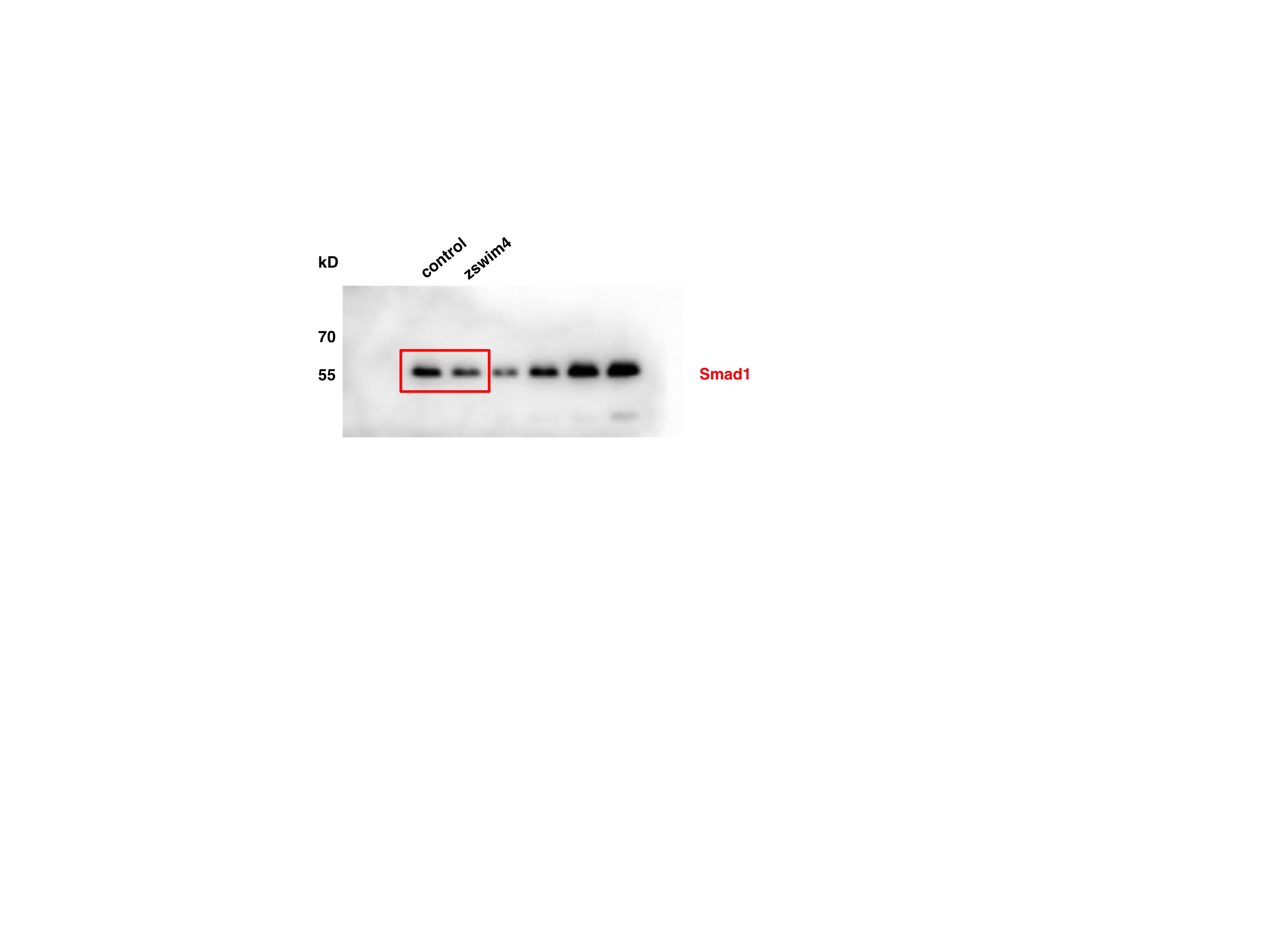

Supplement: Supplementary file 4 — Source Data Fig. 3 [file 44319_2023_46_MOESM4_ESM.zip › Figure 3/3S/western 3S Smad1.jpg]

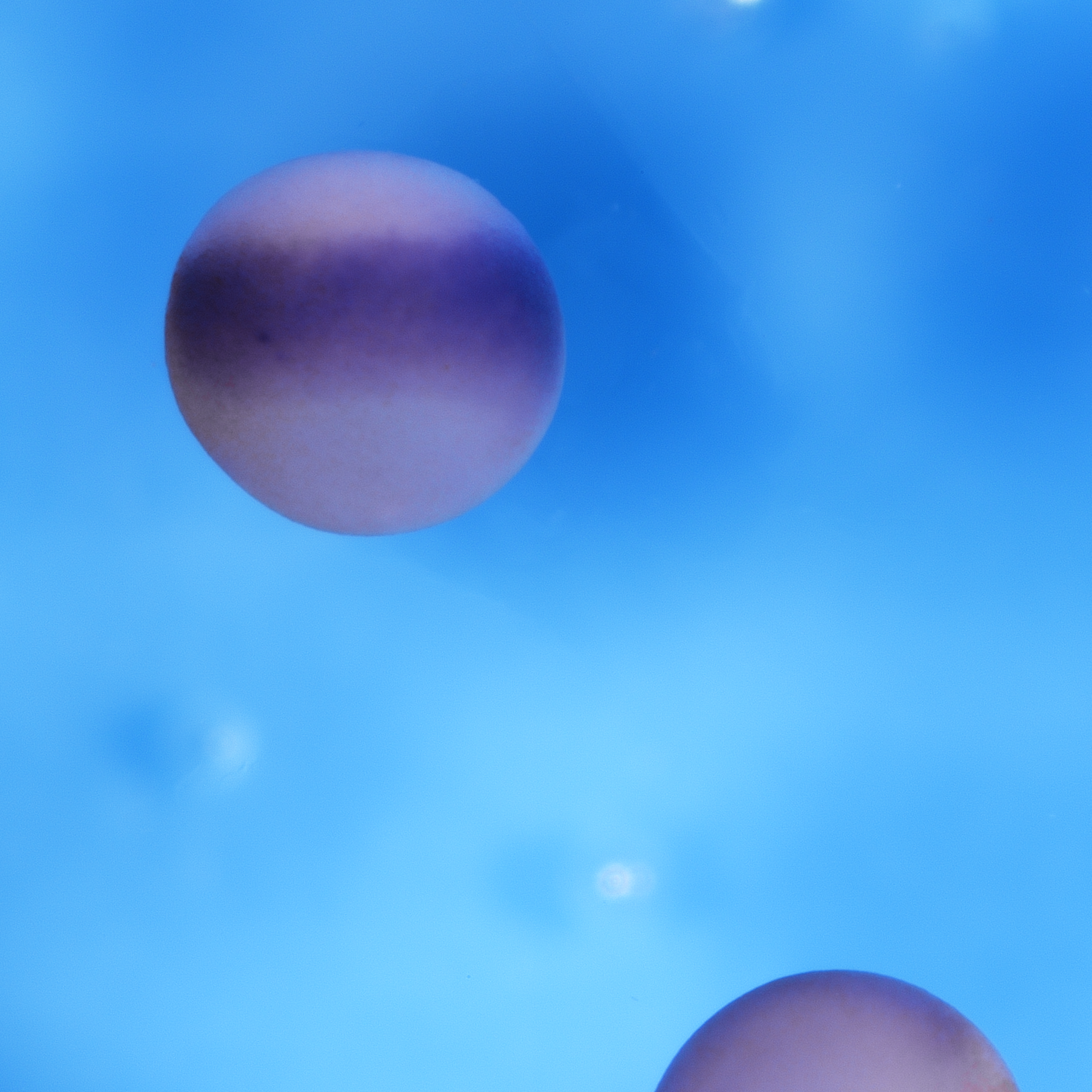

Supplement: Supplementary file 4 — Source Data Fig. 3 [file 44319_2023_46_MOESM4_ESM.zip › Figure 3/3O/image 3O mild.tif]

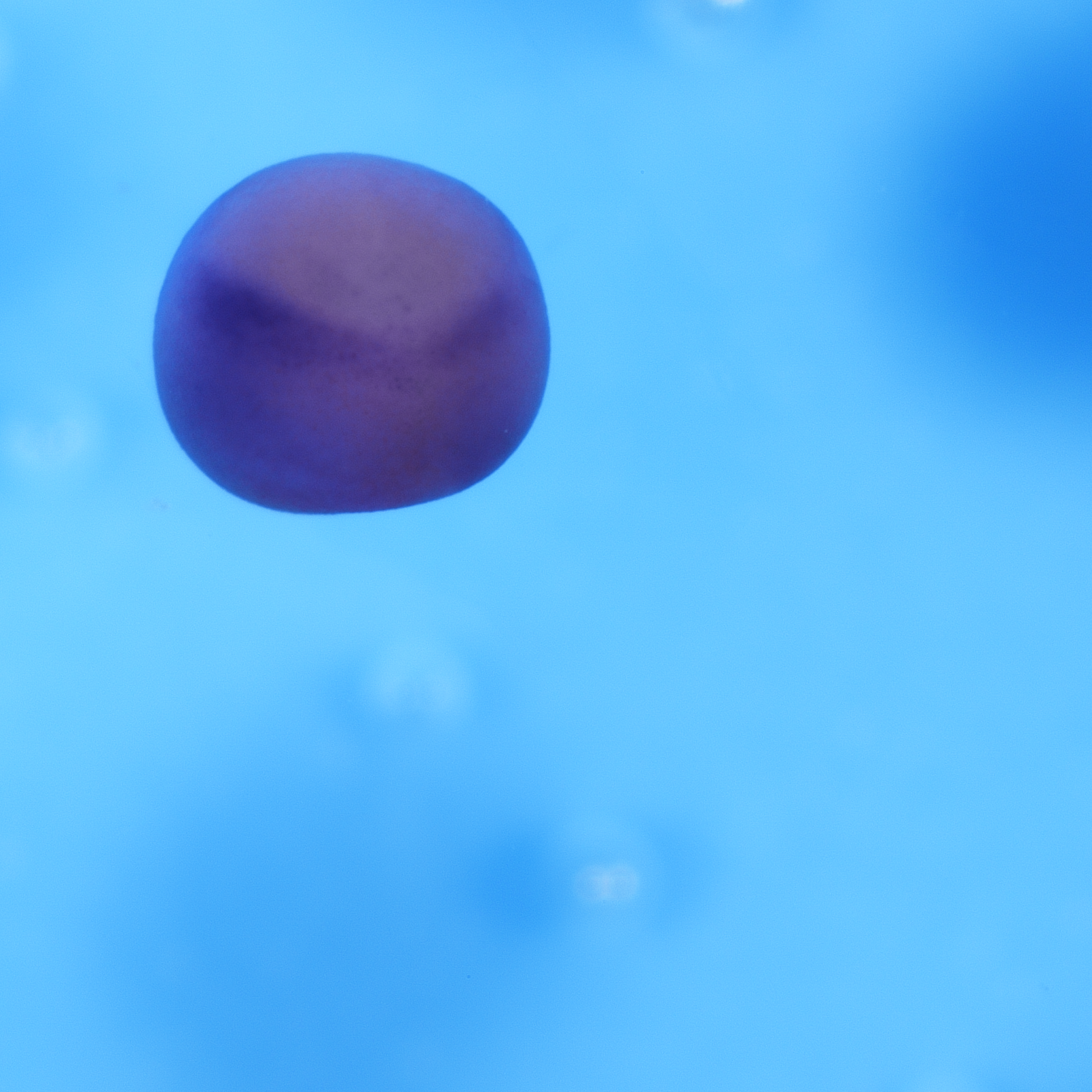

Supplement: Supplementary file 4 — Source Data Fig. 3 [file 44319_2023_46_MOESM4_ESM.zip › Figure 3/3O/image 3O normal.tif]

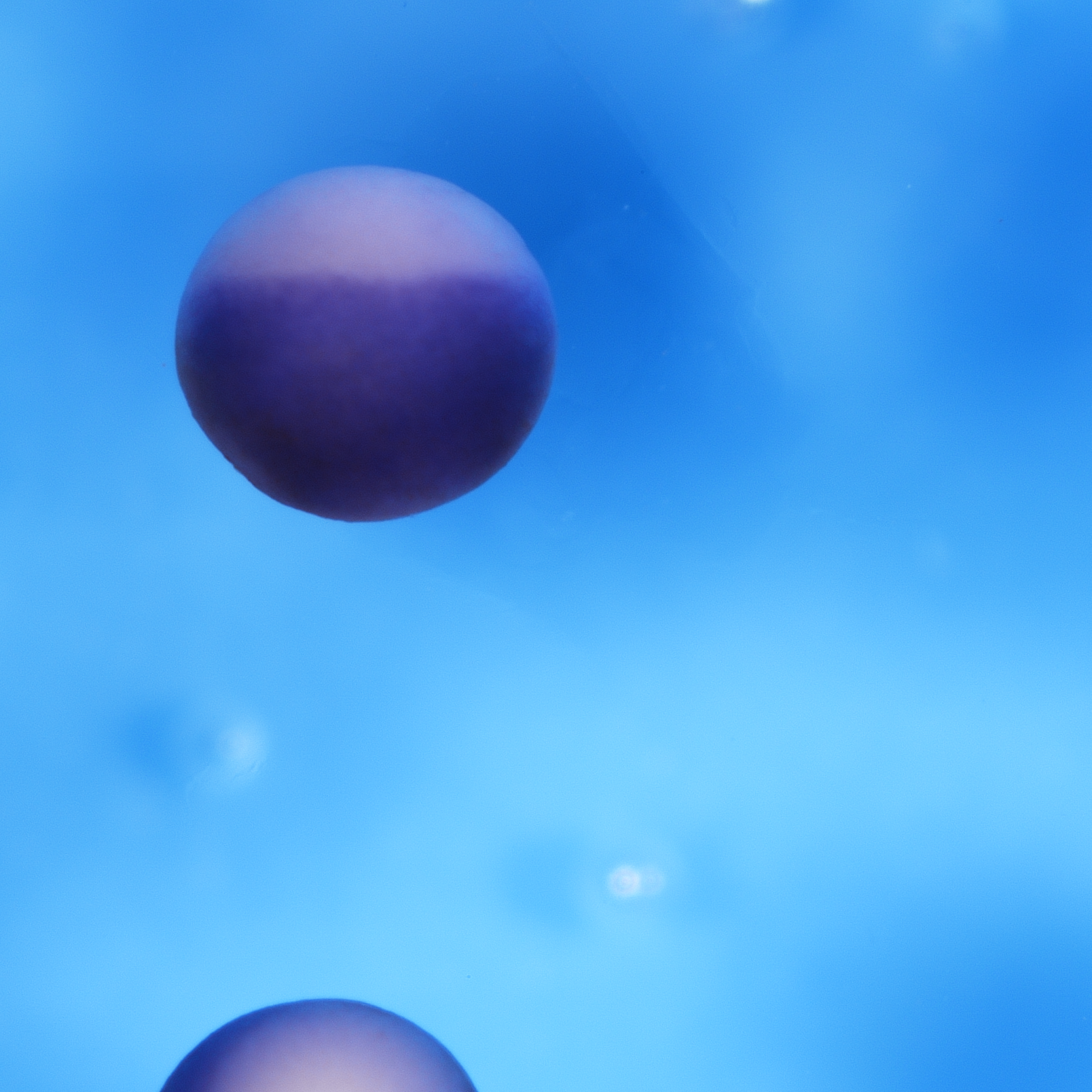

Supplement: Supplementary file 4 — Source Data Fig. 3 [file 44319_2023_46_MOESM4_ESM.zip › Figure 3/3O/image 3O severe.tif]

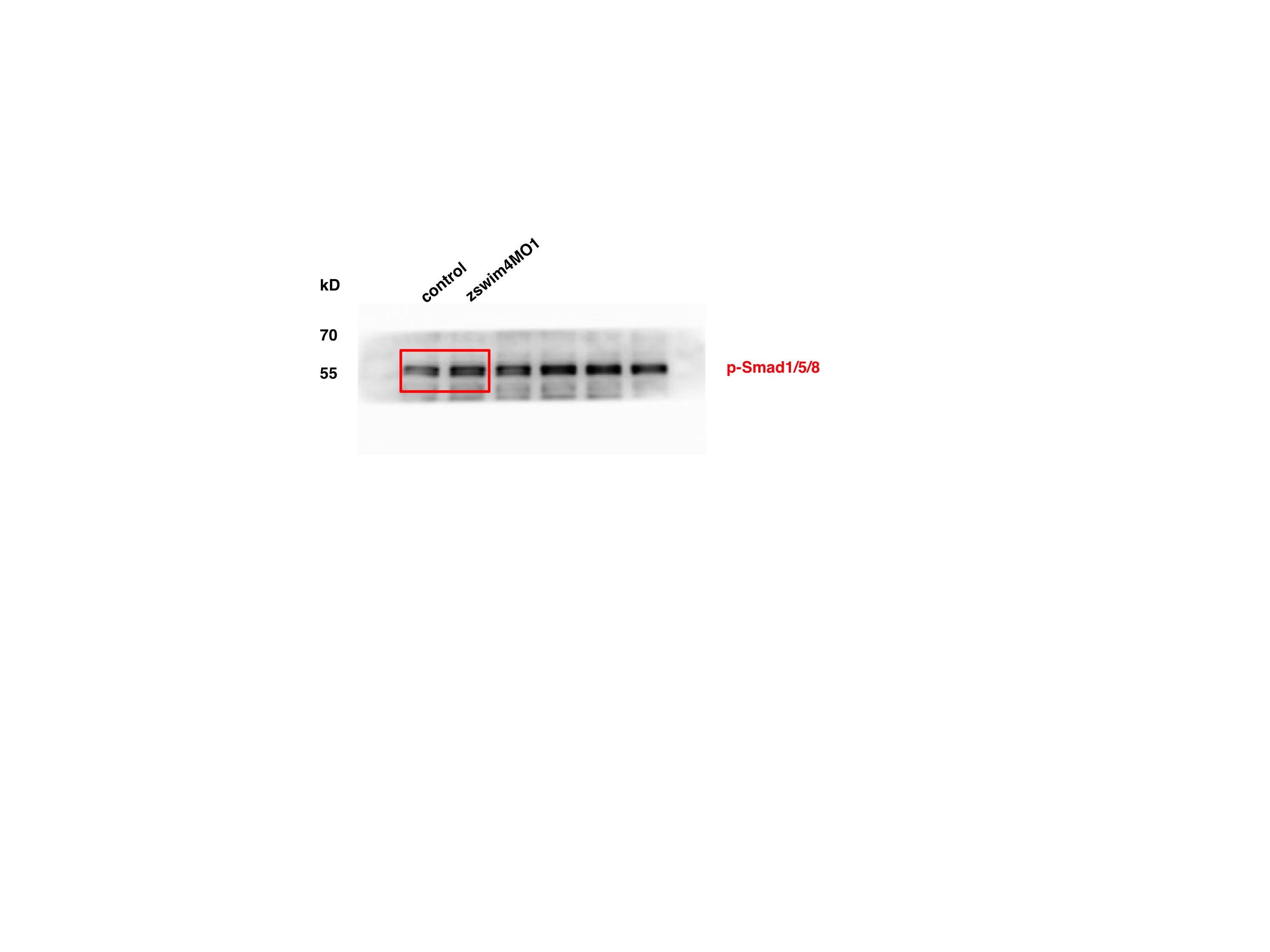

Supplement: Supplementary file 4 — Source Data Fig. 3 [file 44319_2023_46_MOESM4_ESM.zip › Figure 3/3T/replicate/western 3T p-Smad1 replicate.jpg]

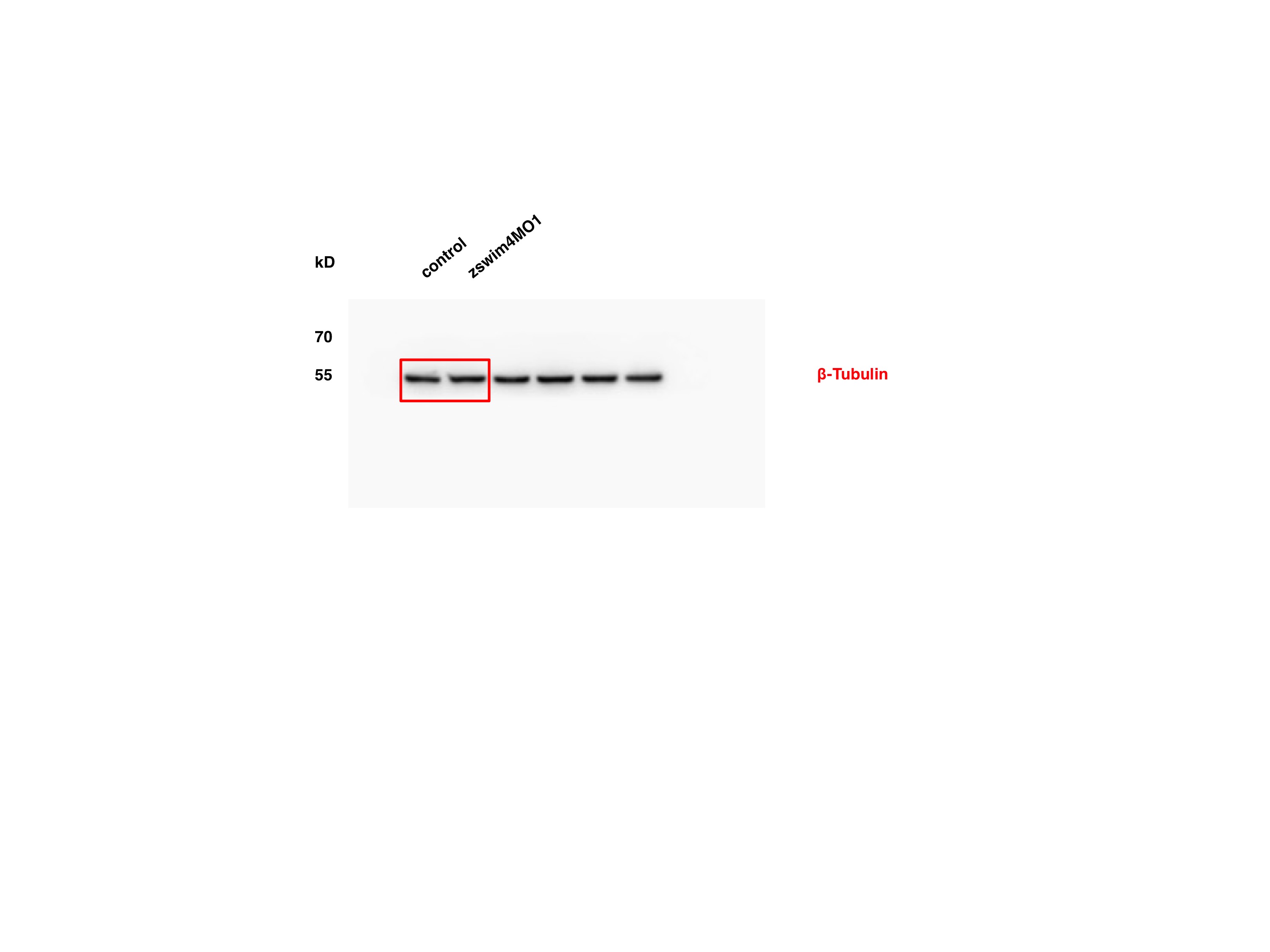

Supplement: Supplementary file 4 — Source Data Fig. 3 [file 44319_2023_46_MOESM4_ESM.zip › Figure 3/3T/replicate/western 3T tubulin replicate.jpg]

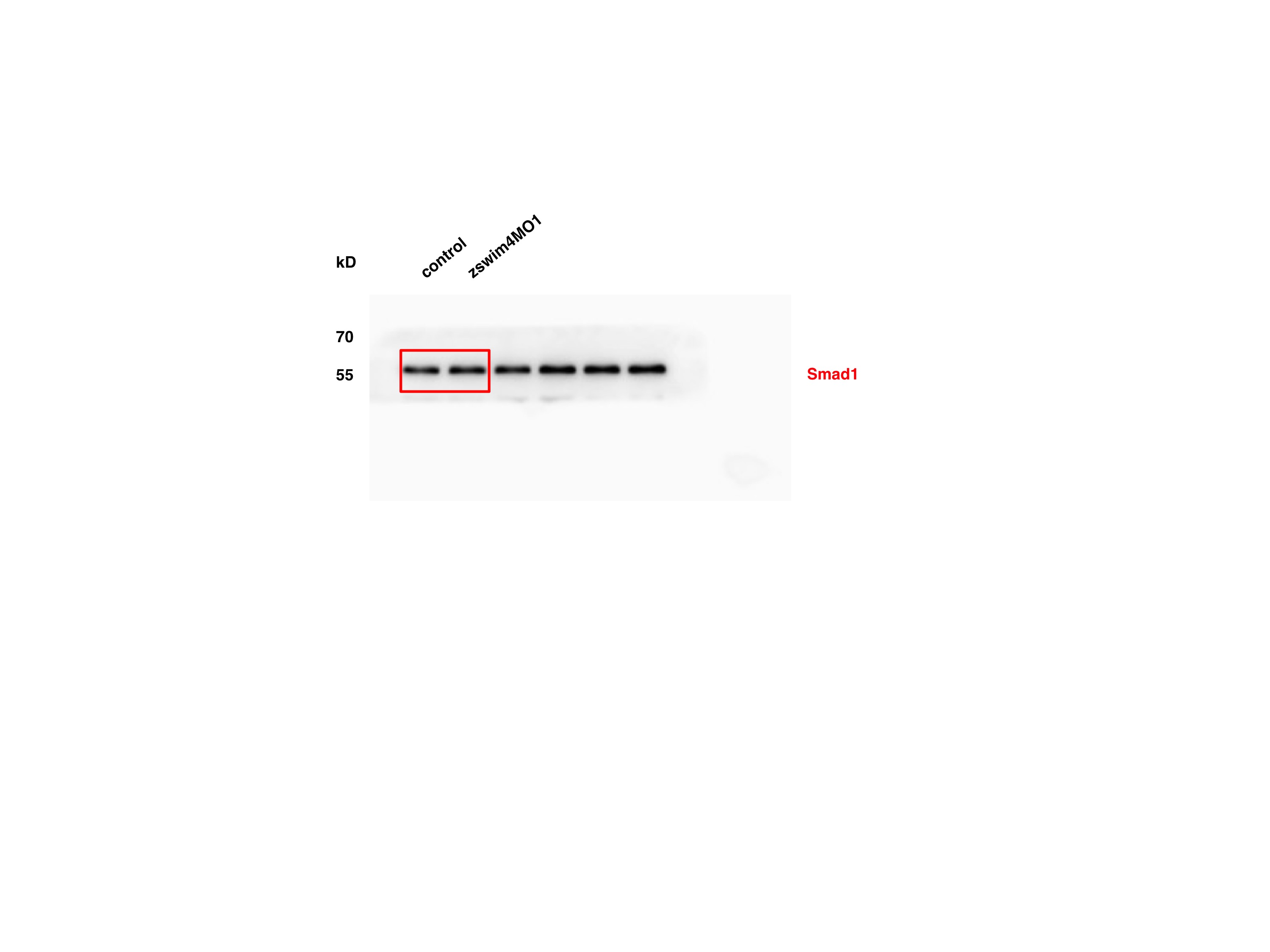

Supplement: Supplementary file 4 — Source Data Fig. 3 [file 44319_2023_46_MOESM4_ESM.zip › Figure 3/3T/replicate/western 3T Smad1 replicate.jpg]

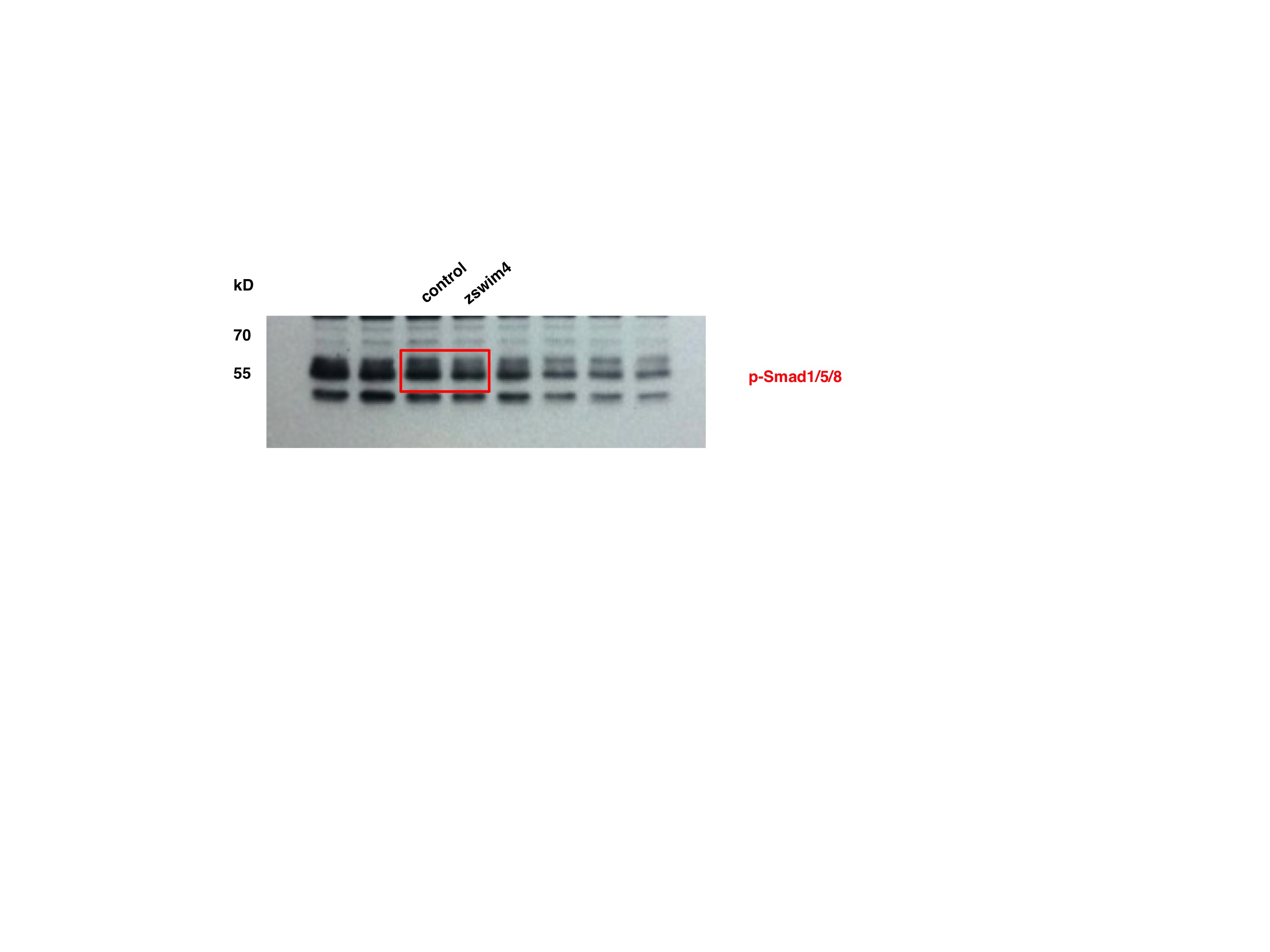

Supplement: Supplementary file 4 — Source Data Fig. 3 [file 44319_2023_46_MOESM4_ESM.zip › Figure 3/3S/replicate/western 3S p-SMAD1 replicate.jpg]

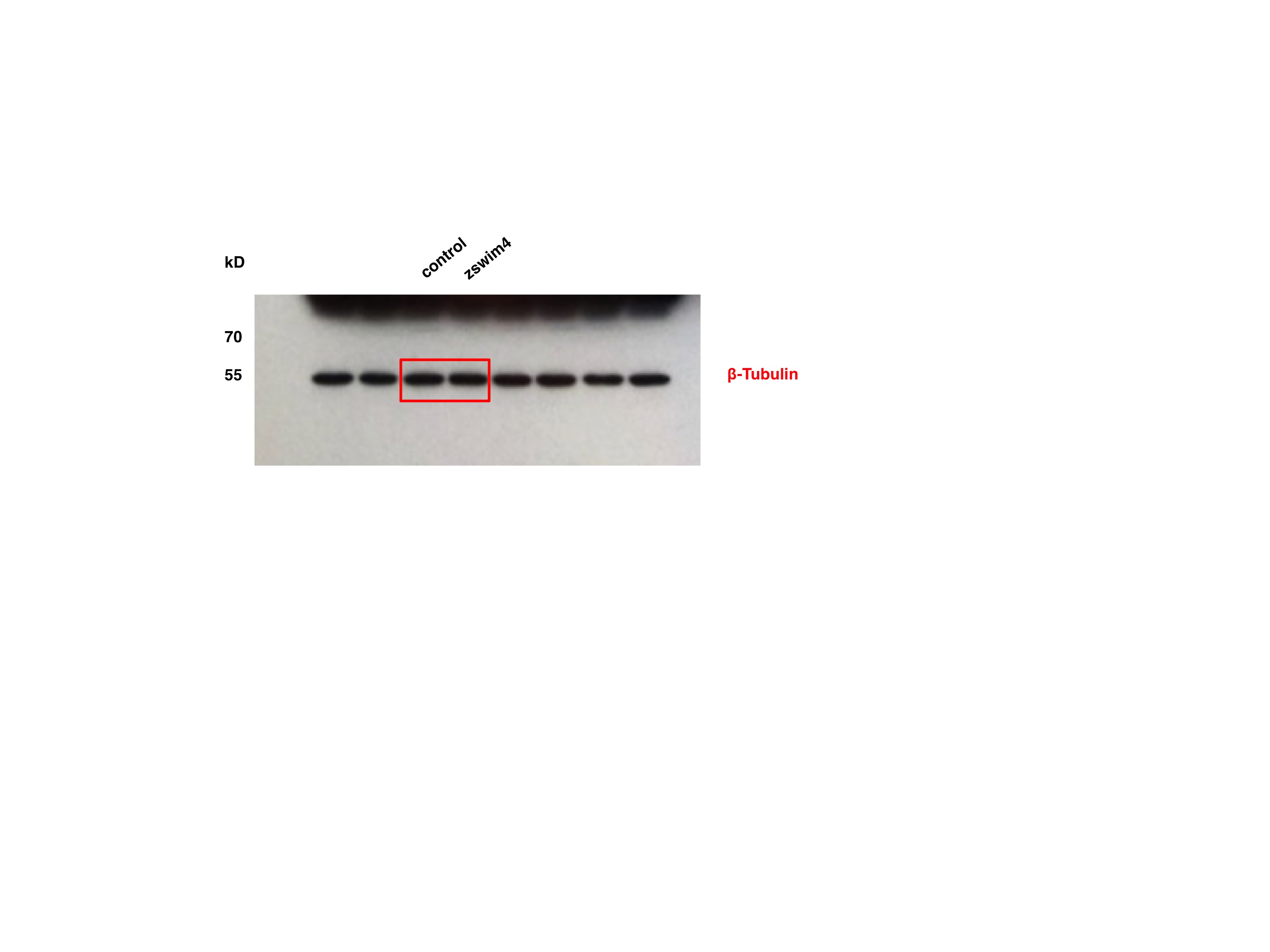

Supplement: Supplementary file 4 — Source Data Fig. 3 [file 44319_2023_46_MOESM4_ESM.zip › Figure 3/3S/replicate/western 3S tubulin replicate.jpg]

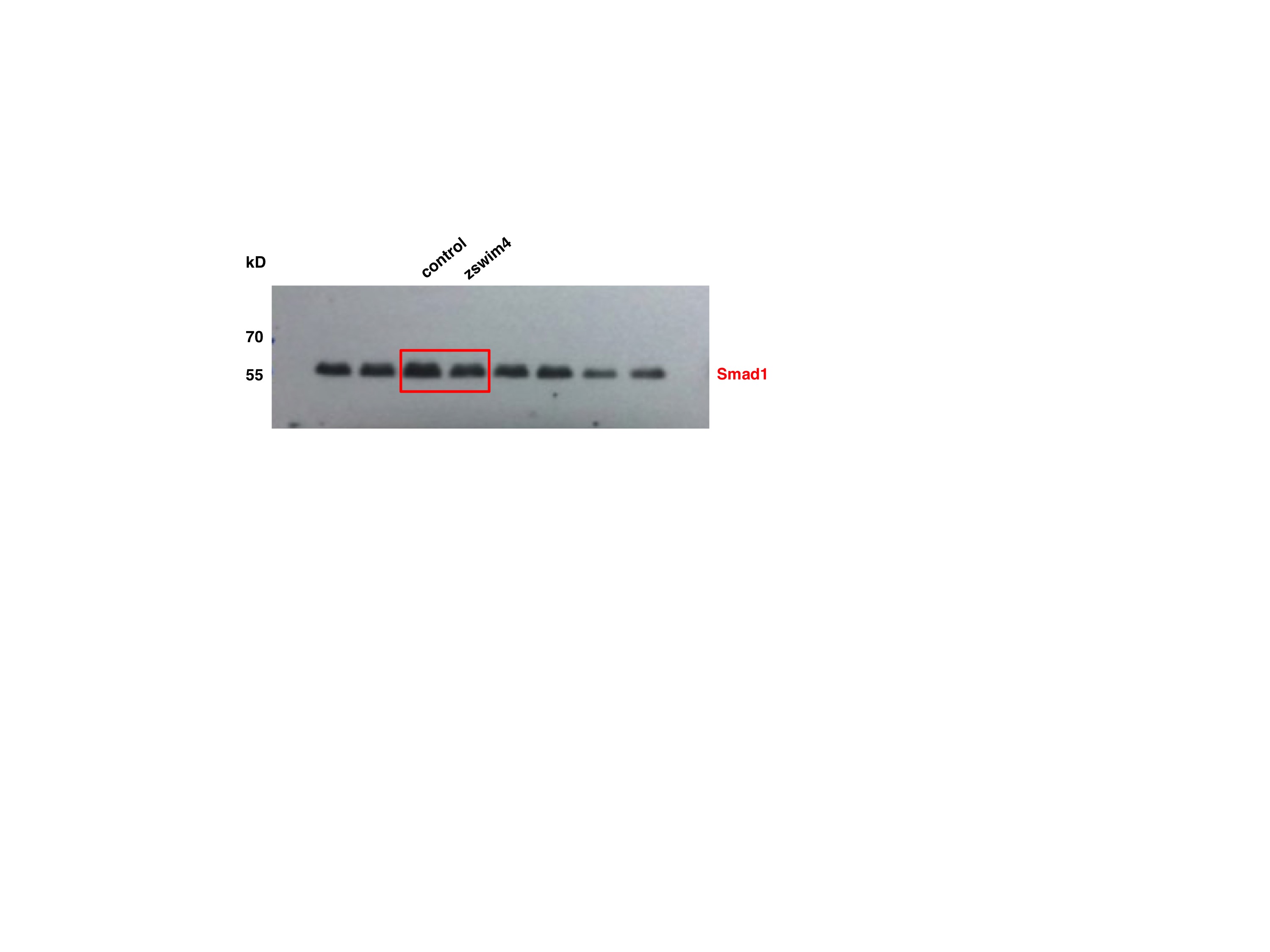

Supplement: Supplementary file 4 — Source Data Fig. 3 [file 44319_2023_46_MOESM4_ESM.zip › Figure 3/3S/replicate/western 3S Smad1 replicate.jpg]

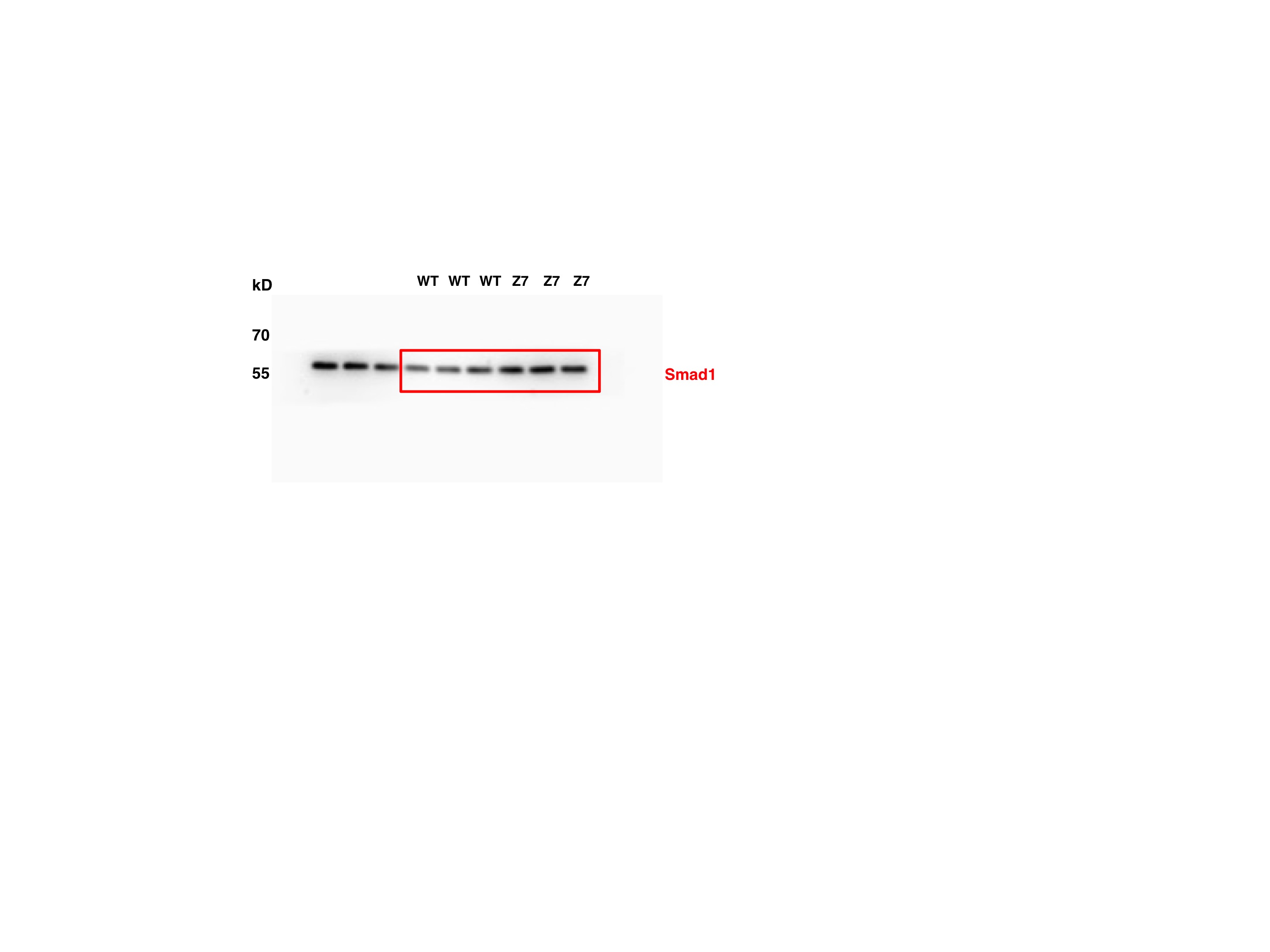

Supplement: Supplementary file 5 — Source Data Fig. 4 [file 44319_2023_46_MOESM5_ESM.zip › Figure 4/4K/western 4K Smad1 .jpg]

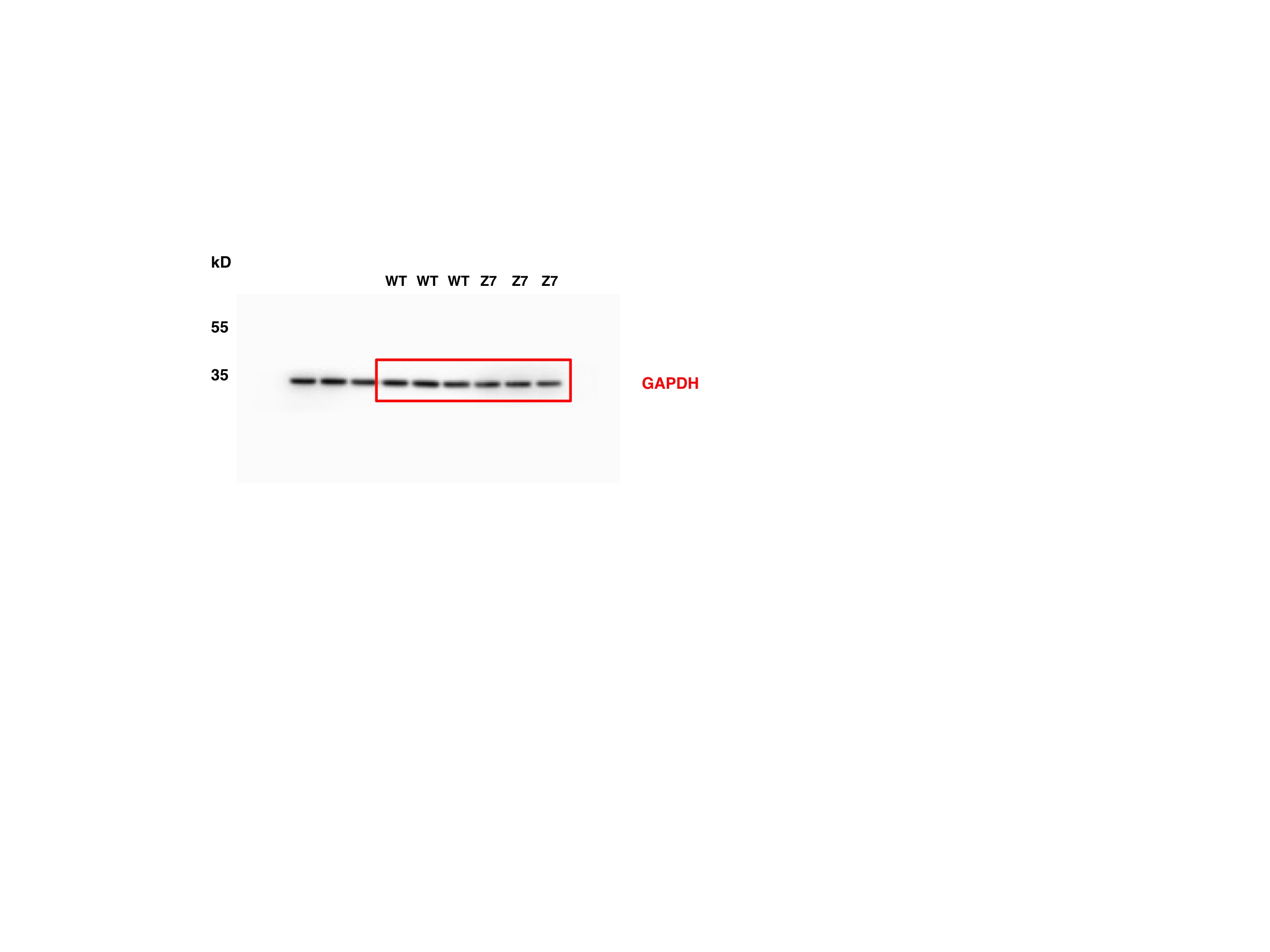

Supplement: Supplementary file 5 — Source Data Fig. 4 [file 44319_2023_46_MOESM5_ESM.zip › Figure 4/4K/western 4K GAPDH.jpg]

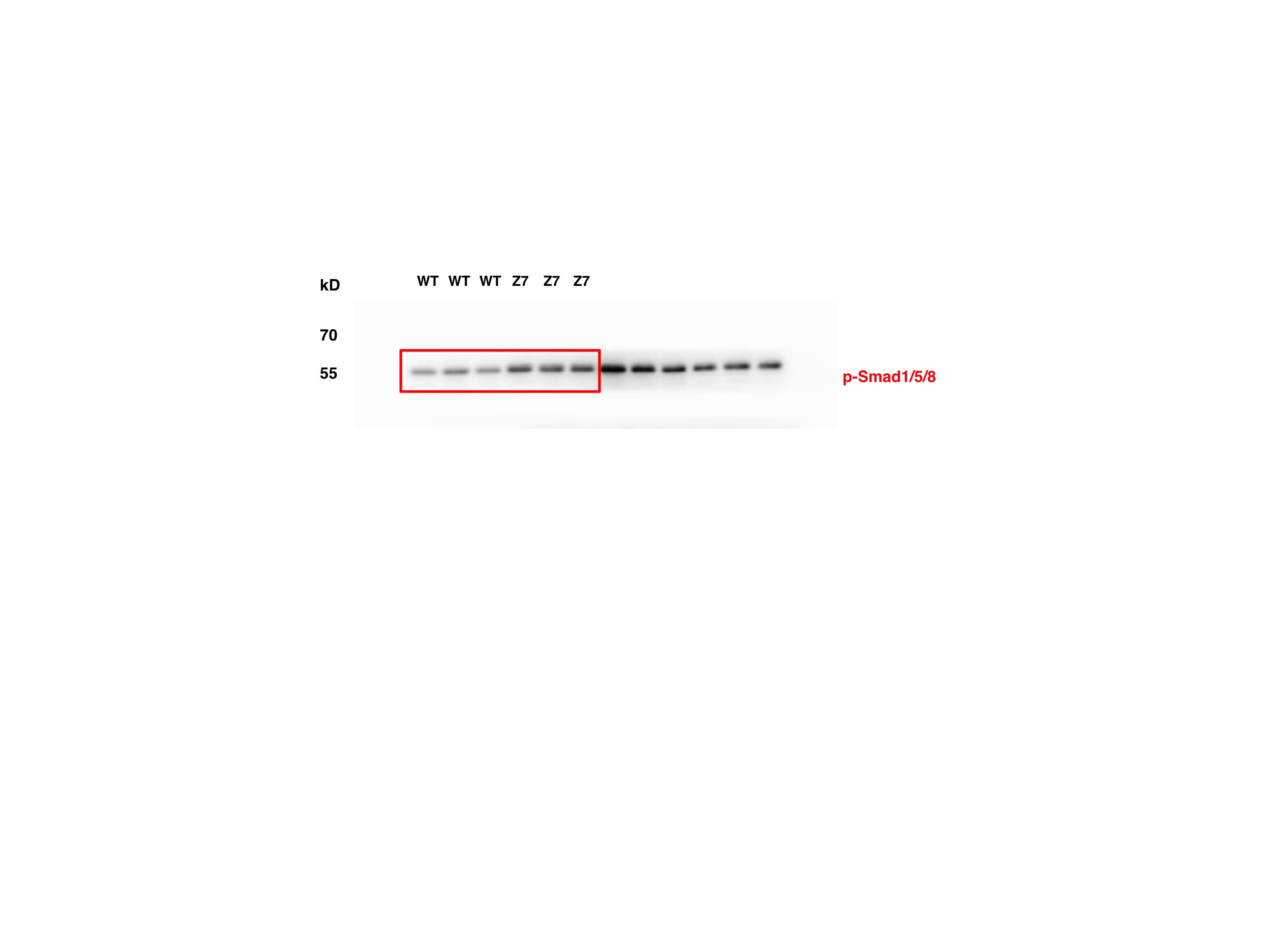

Supplement: Supplementary file 5 — Source Data Fig. 4 [file 44319_2023_46_MOESM5_ESM.zip › Figure 4/4K/western 4K p-Smad1 .jpg]

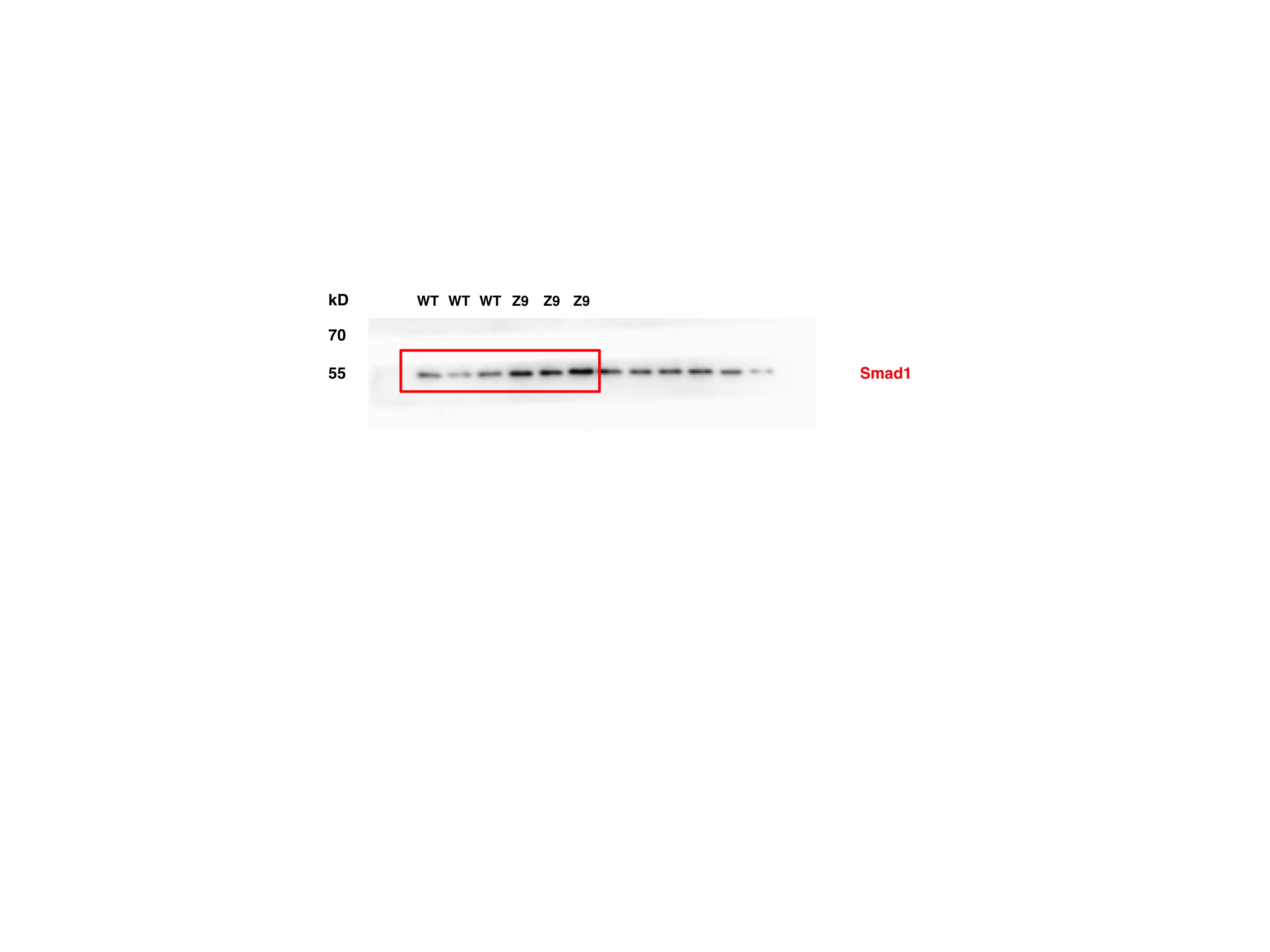

Supplement: Supplementary file 5 — Source Data Fig. 4 [file 44319_2023_46_MOESM5_ESM.zip › Figure 4/4L/western 4L Smad1 .jpg]

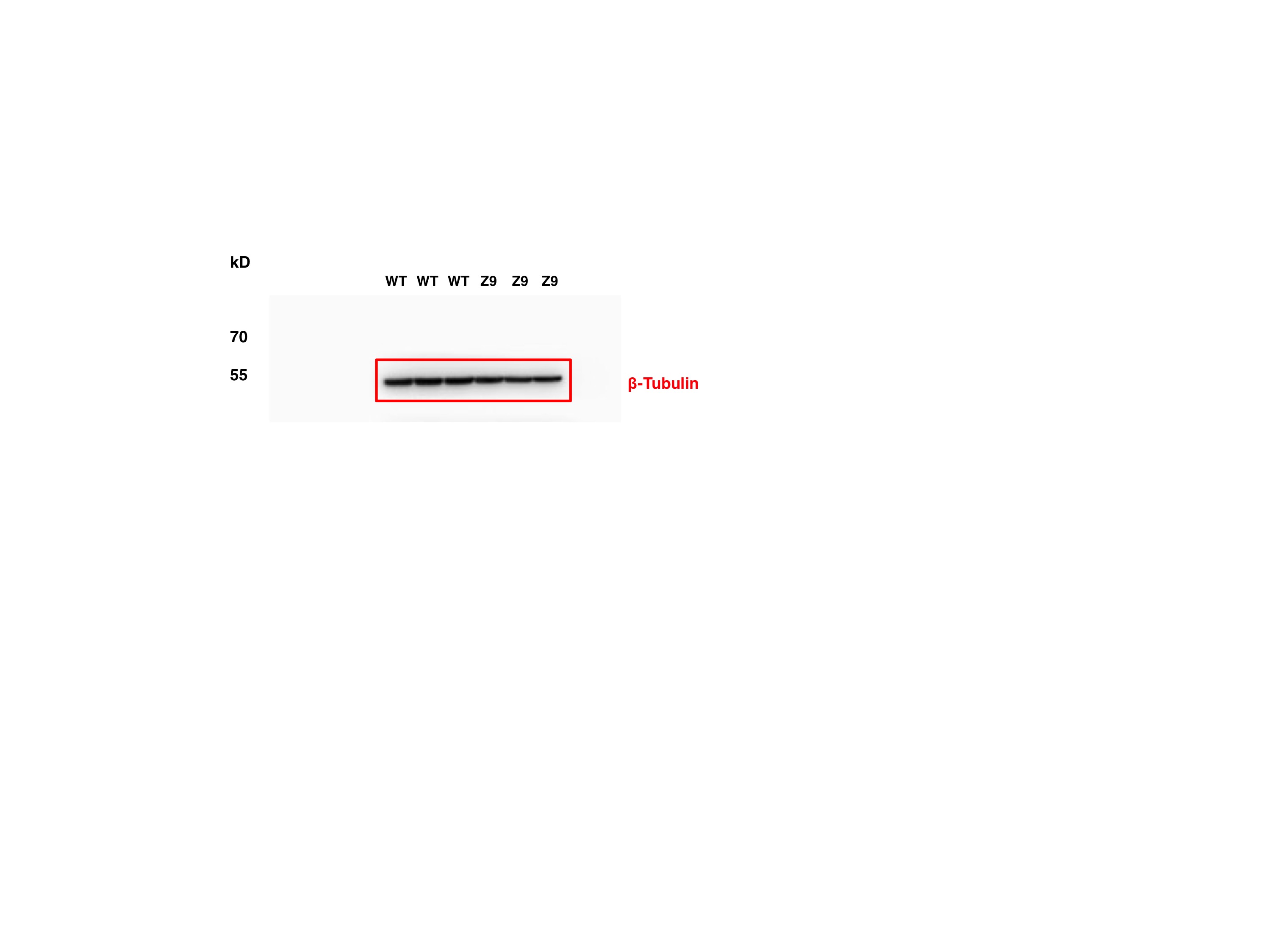

Supplement: Supplementary file 5 — Source Data Fig. 4 [file 44319_2023_46_MOESM5_ESM.zip › Figure 4/4L/western 4L tubulin.jpg]

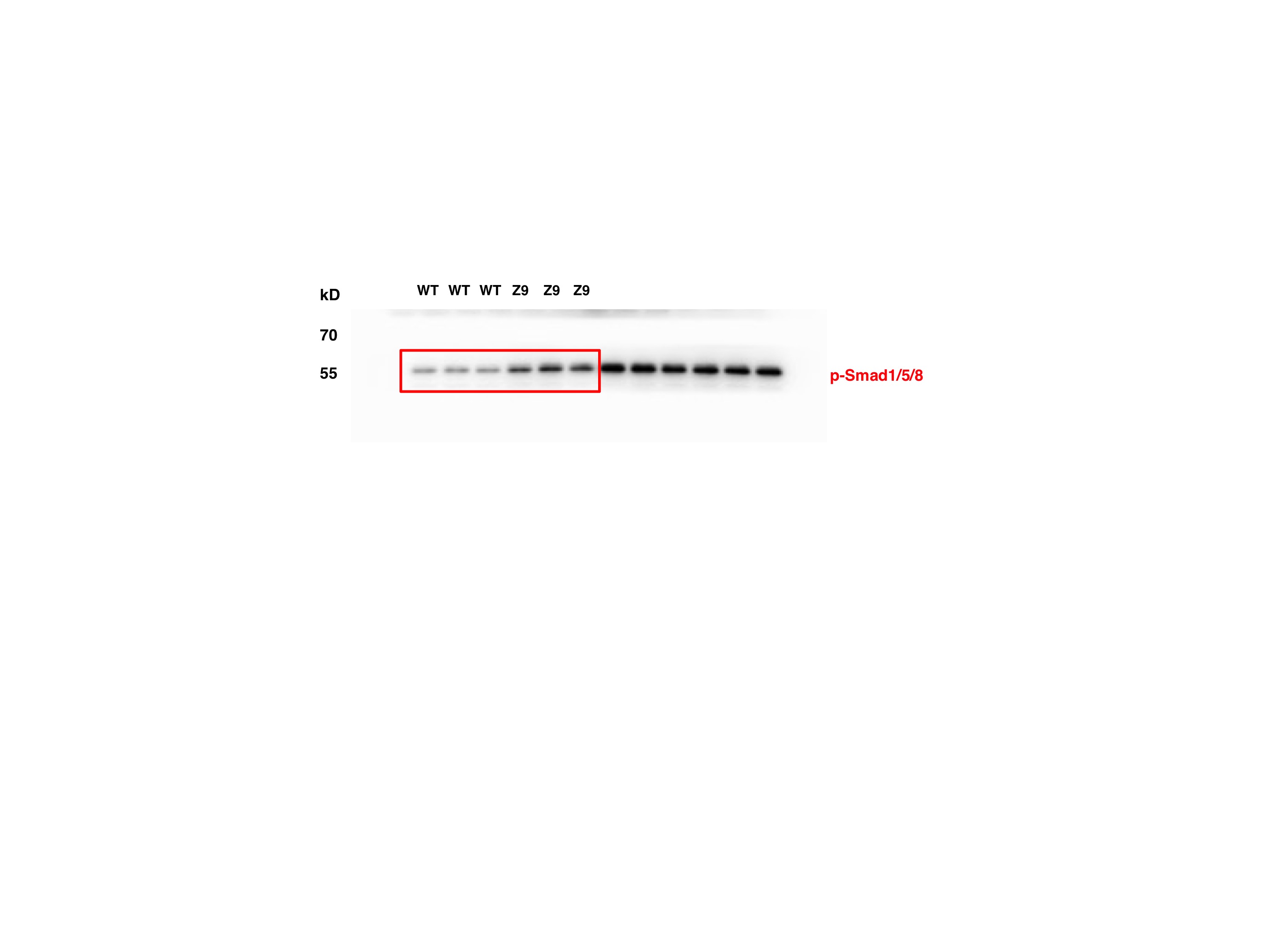

Supplement: Supplementary file 5 — Source Data Fig. 4 [file 44319_2023_46_MOESM5_ESM.zip › Figure 4/4L/western 4L p-Smad1 .jpg]

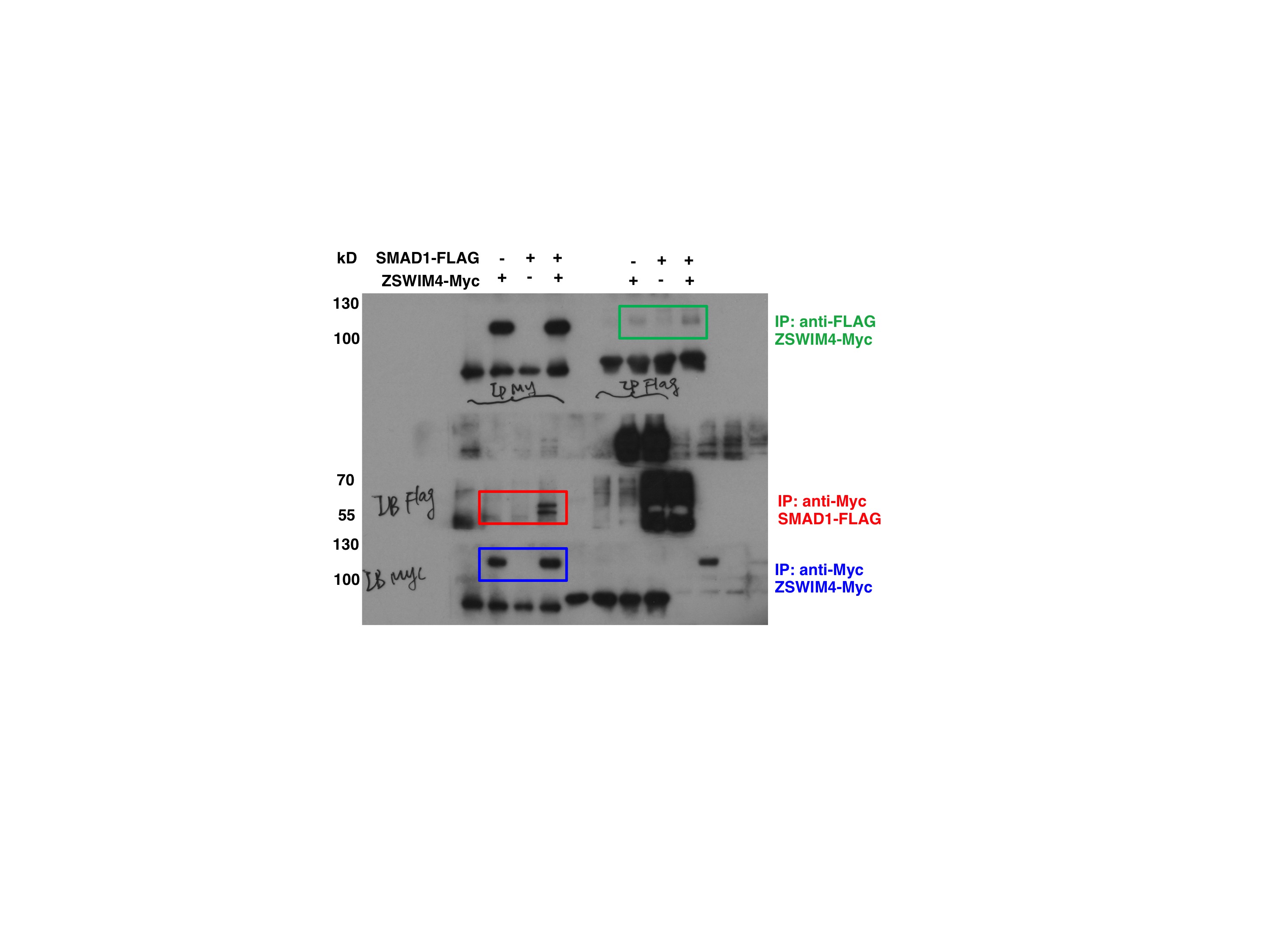

Supplement: Supplementary file 6 — Source Data Fig. 5 [file 44319_2023_46_MOESM6_ESM.zip › Figure 5/5I/western 5I Zswim4 Smad1.jpg]

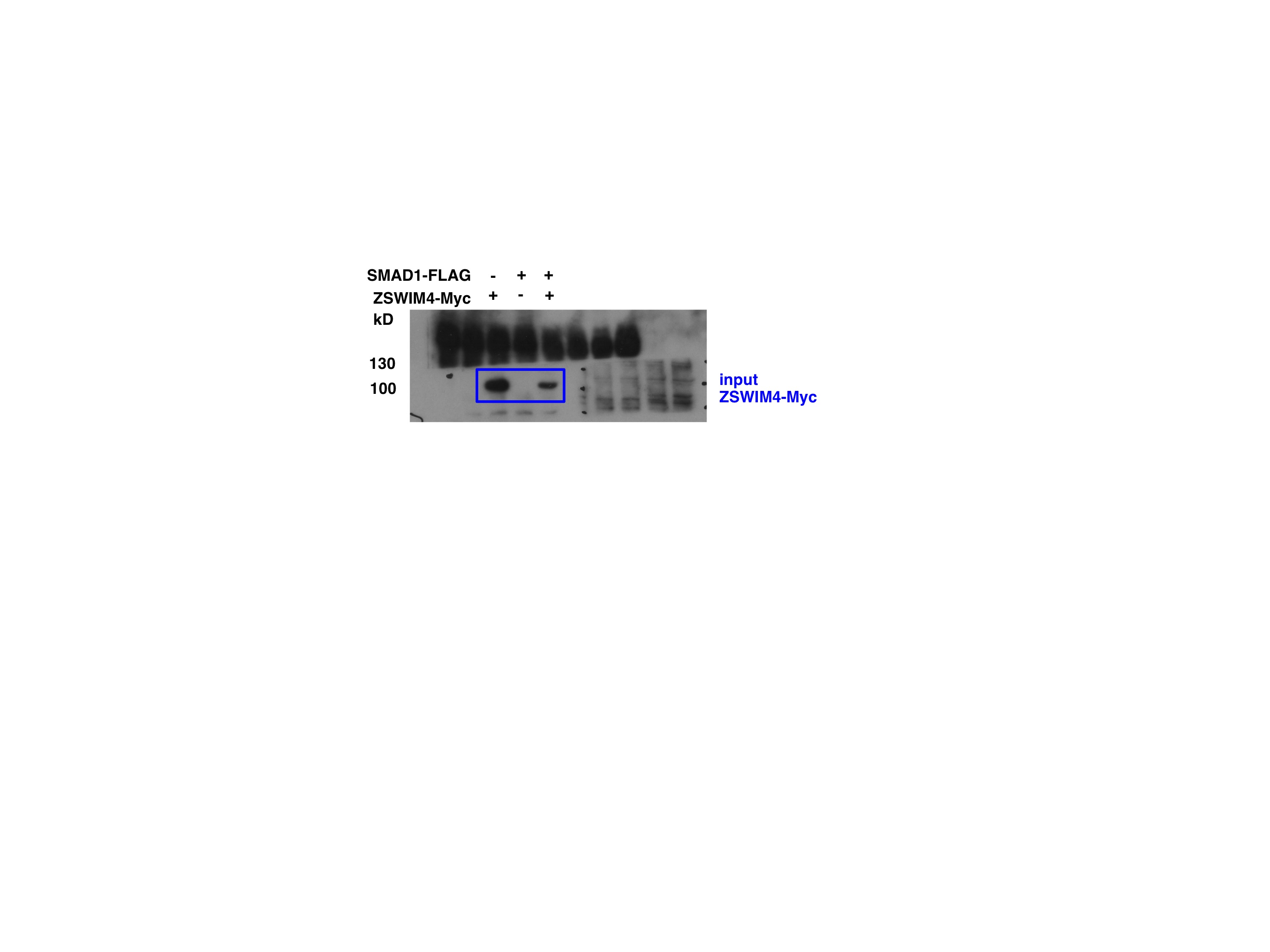

Supplement: Supplementary file 6 — Source Data Fig. 5 [file 44319_2023_46_MOESM6_ESM.zip › Figure 5/5I/western 5I zswim4-2.jpg]

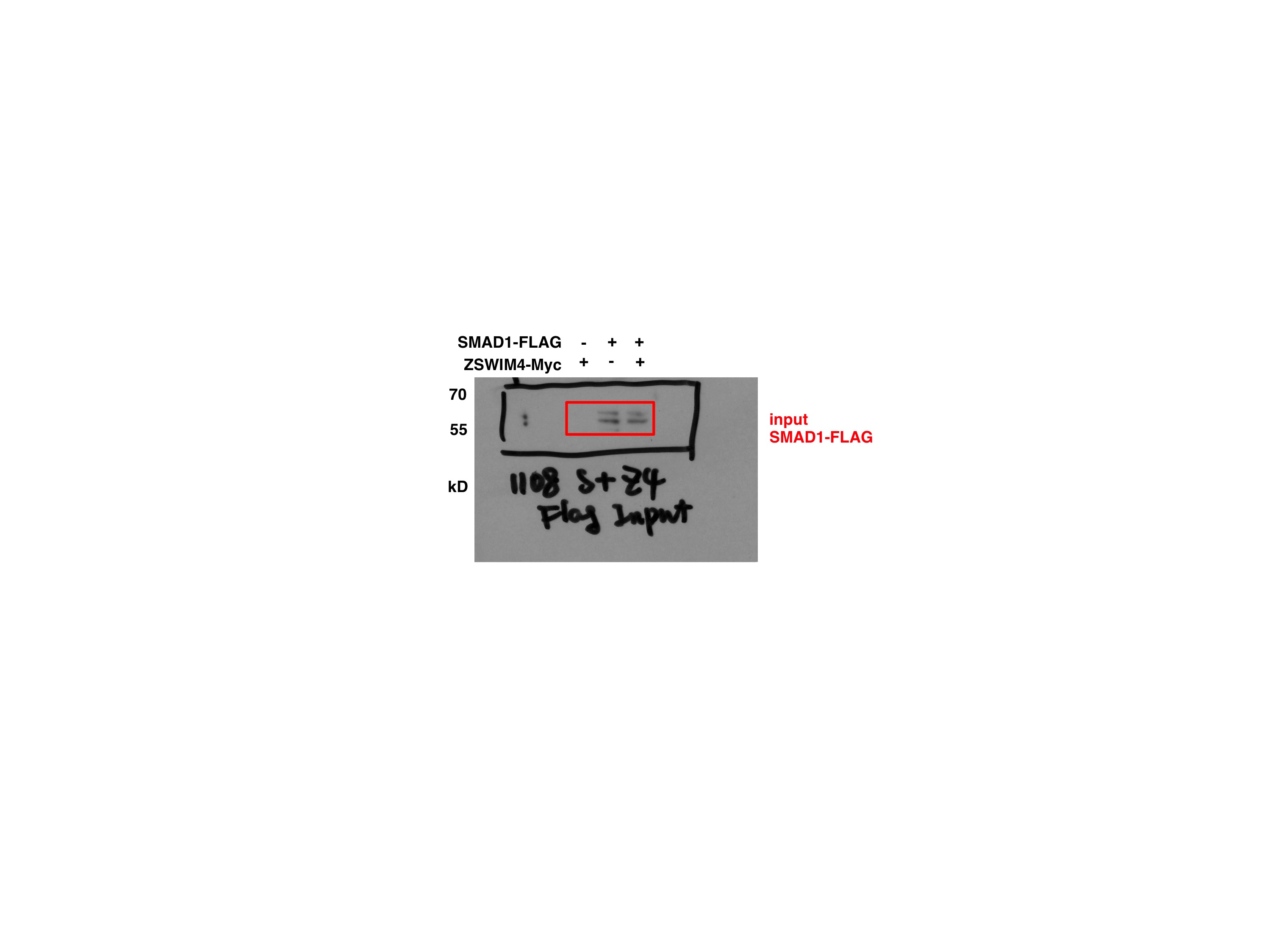

Supplement: Supplementary file 6 — Source Data Fig. 5 [file 44319_2023_46_MOESM6_ESM.zip › Figure 5/5I/western 5I Smad1-3.jpg]

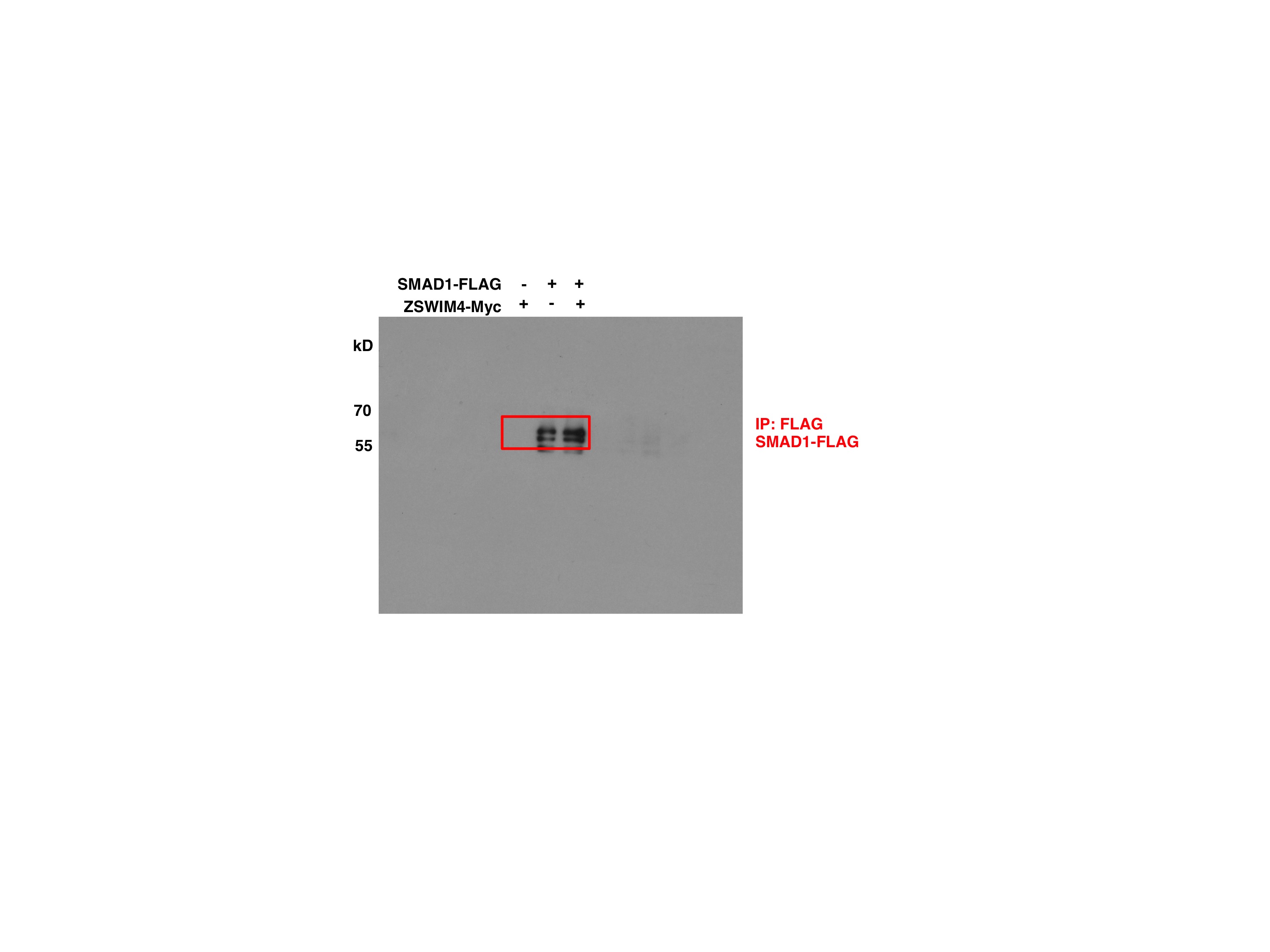

Supplement: Supplementary file 6 — Source Data Fig. 5 [file 44319_2023_46_MOESM6_ESM.zip › Figure 5/5I/western 5I Smad1-2.jpg]

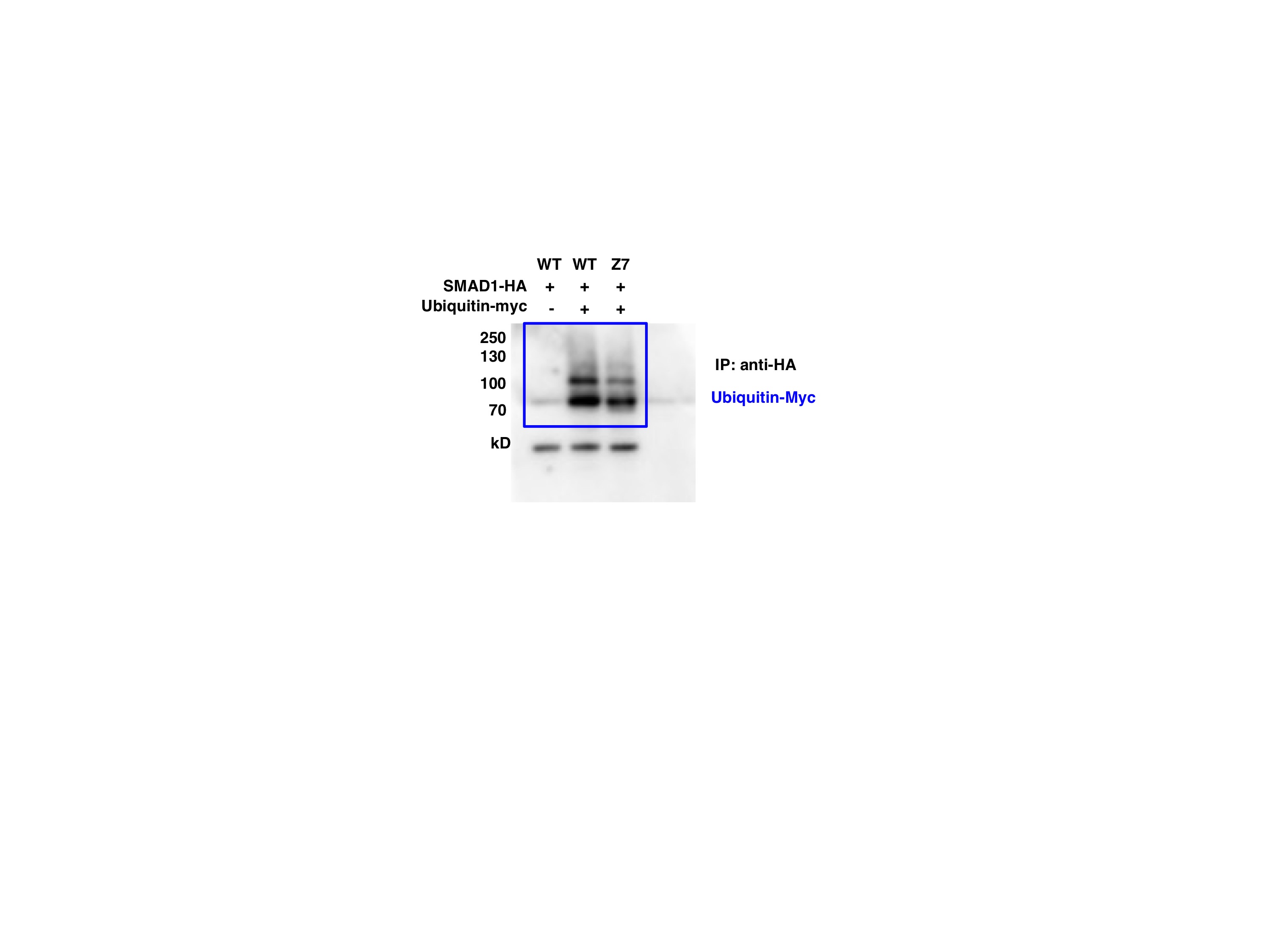

Supplement: Supplementary file 6 — Source Data Fig. 5 [file 44319_2023_46_MOESM6_ESM.zip › Figure 5/5G/western 5G Ubiqutin.jpg]

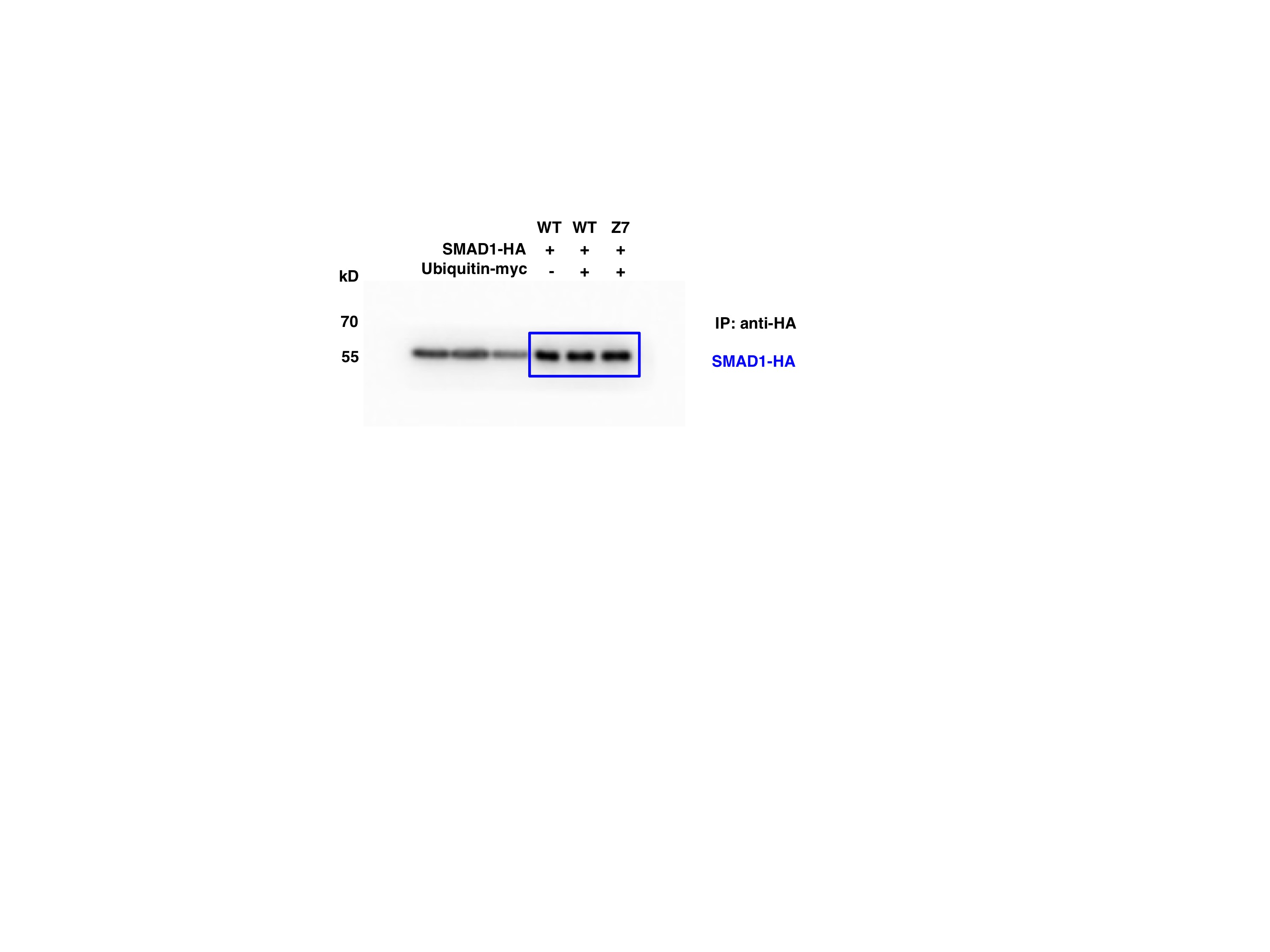

Supplement: Supplementary file 6 — Source Data Fig. 5 [file 44319_2023_46_MOESM6_ESM.zip › Figure 5/5G/western 5G Smad1.jpg]

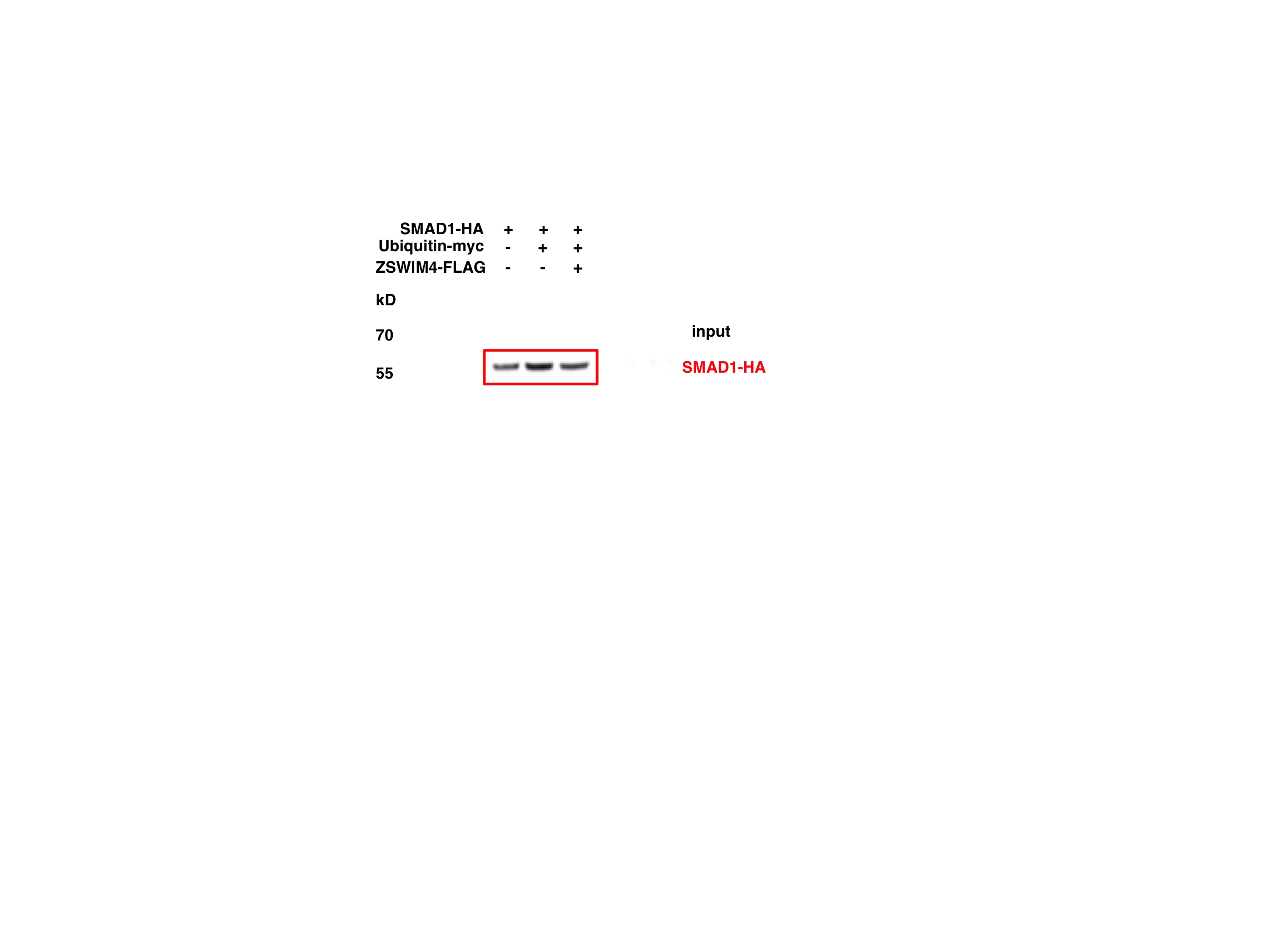

Supplement: Supplementary file 6 — Source Data Fig. 5 [file 44319_2023_46_MOESM6_ESM.zip › Figure 5/5F/western 5F Smad1-1.jpg]

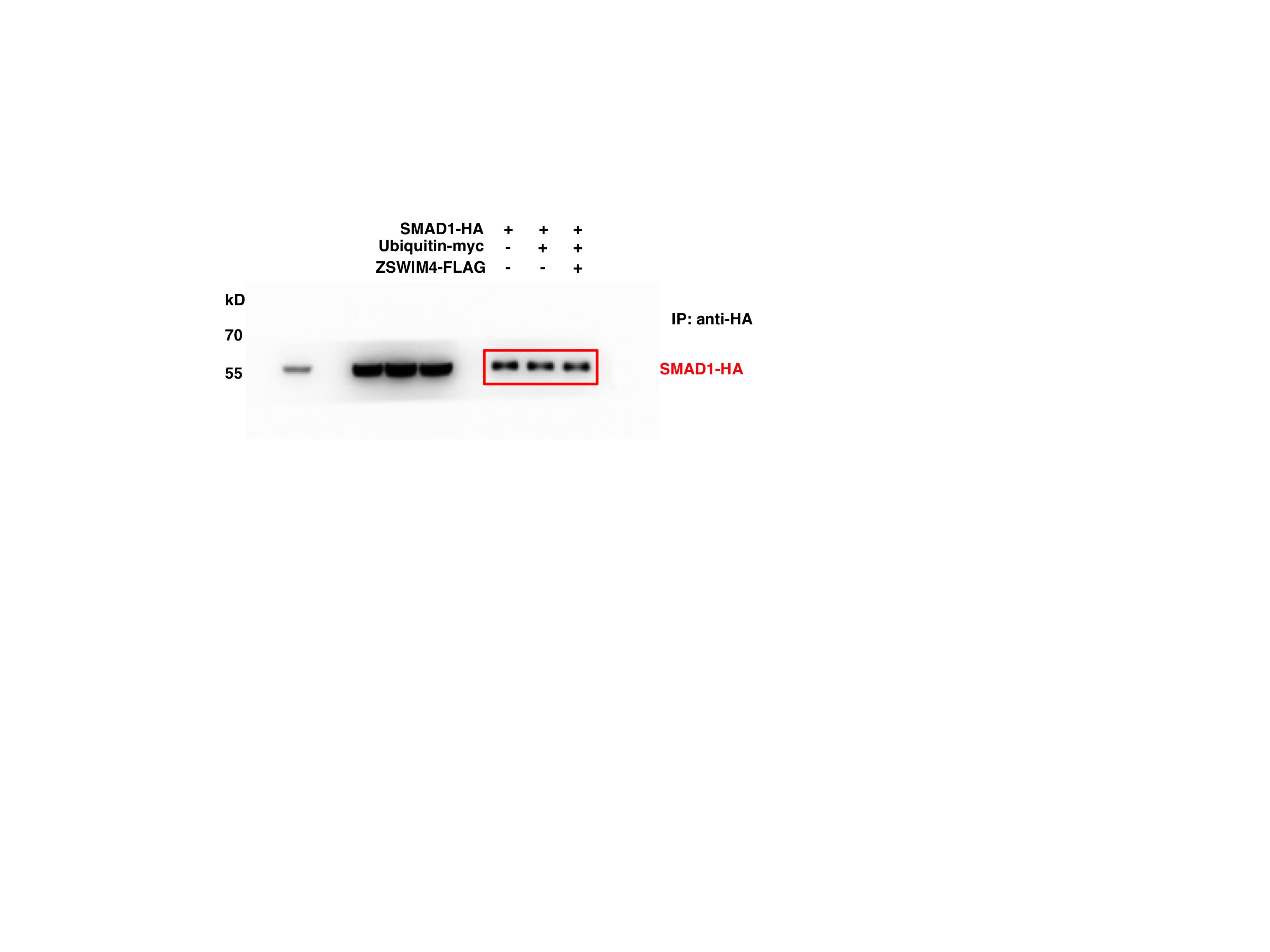

Supplement: Supplementary file 6 — Source Data Fig. 5 [file 44319_2023_46_MOESM6_ESM.zip › Figure 5/5F/western 5F Smad1-2.jpg]

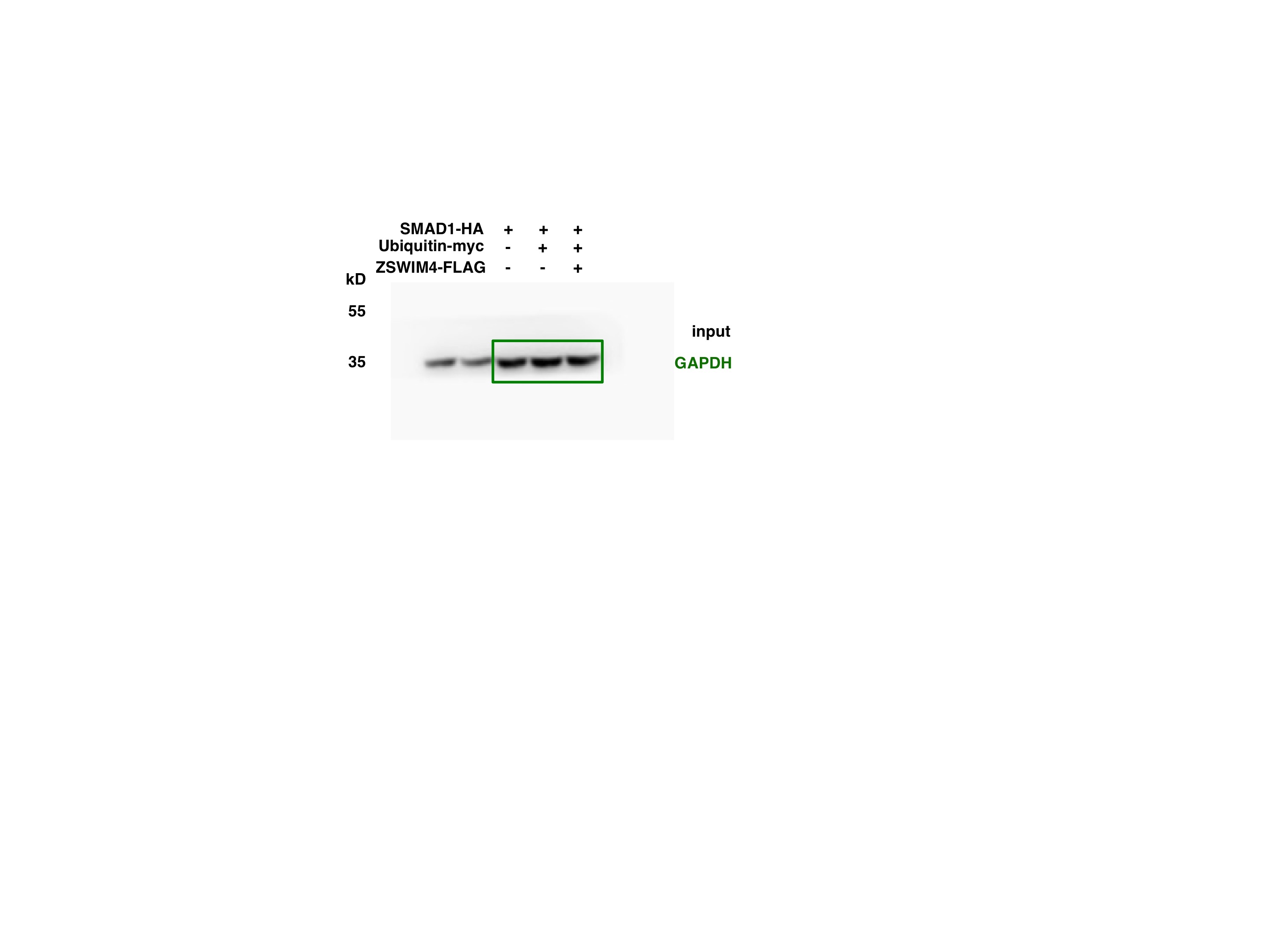

Supplement: Supplementary file 6 — Source Data Fig. 5 [file 44319_2023_46_MOESM6_ESM.zip › Figure 5/5F/western 5F GAPDH.jpg]

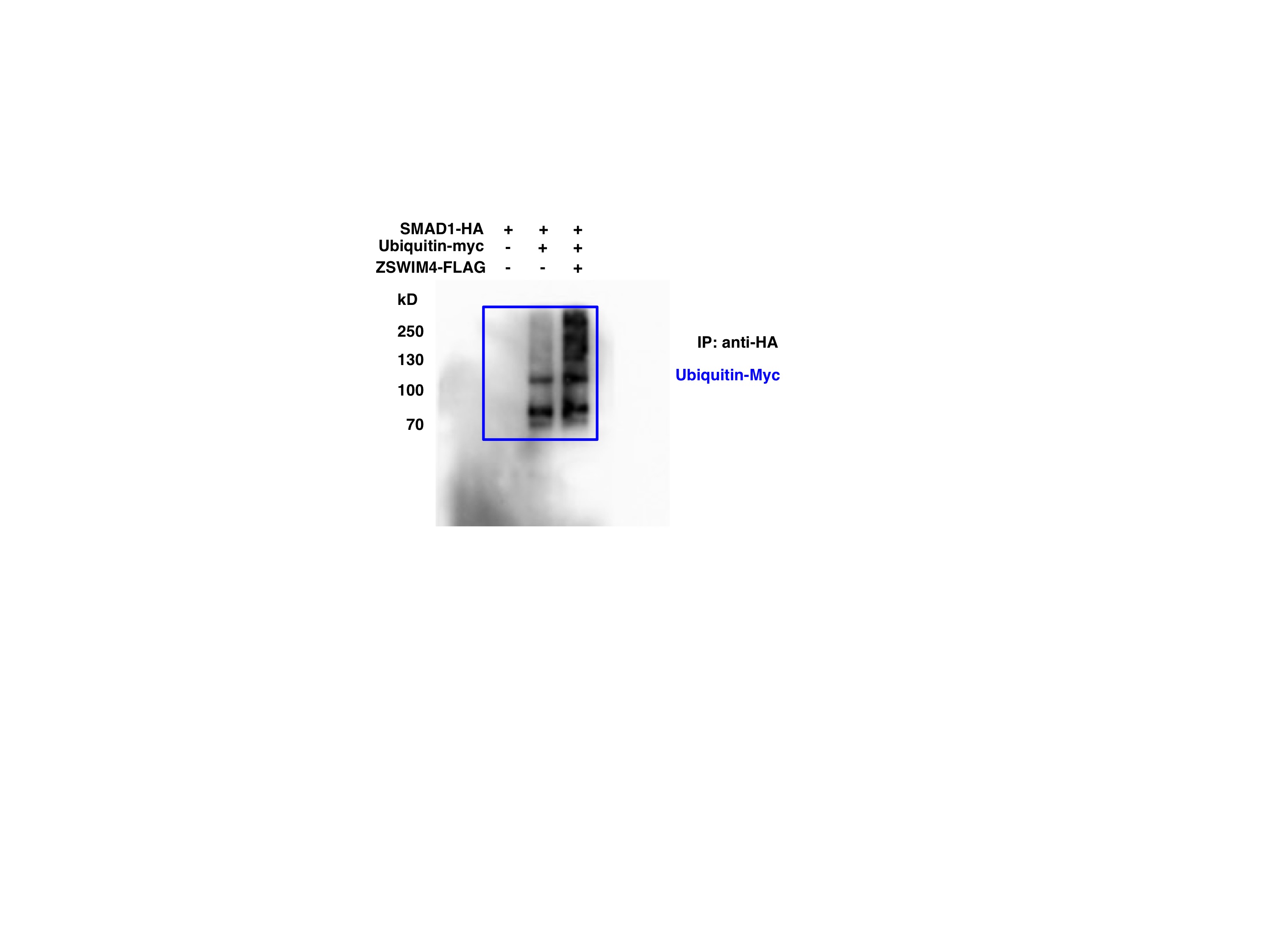

Supplement: Supplementary file 6 — Source Data Fig. 5 [file 44319_2023_46_MOESM6_ESM.zip › Figure 5/5F/western 5F Ubiquitin.jpg]

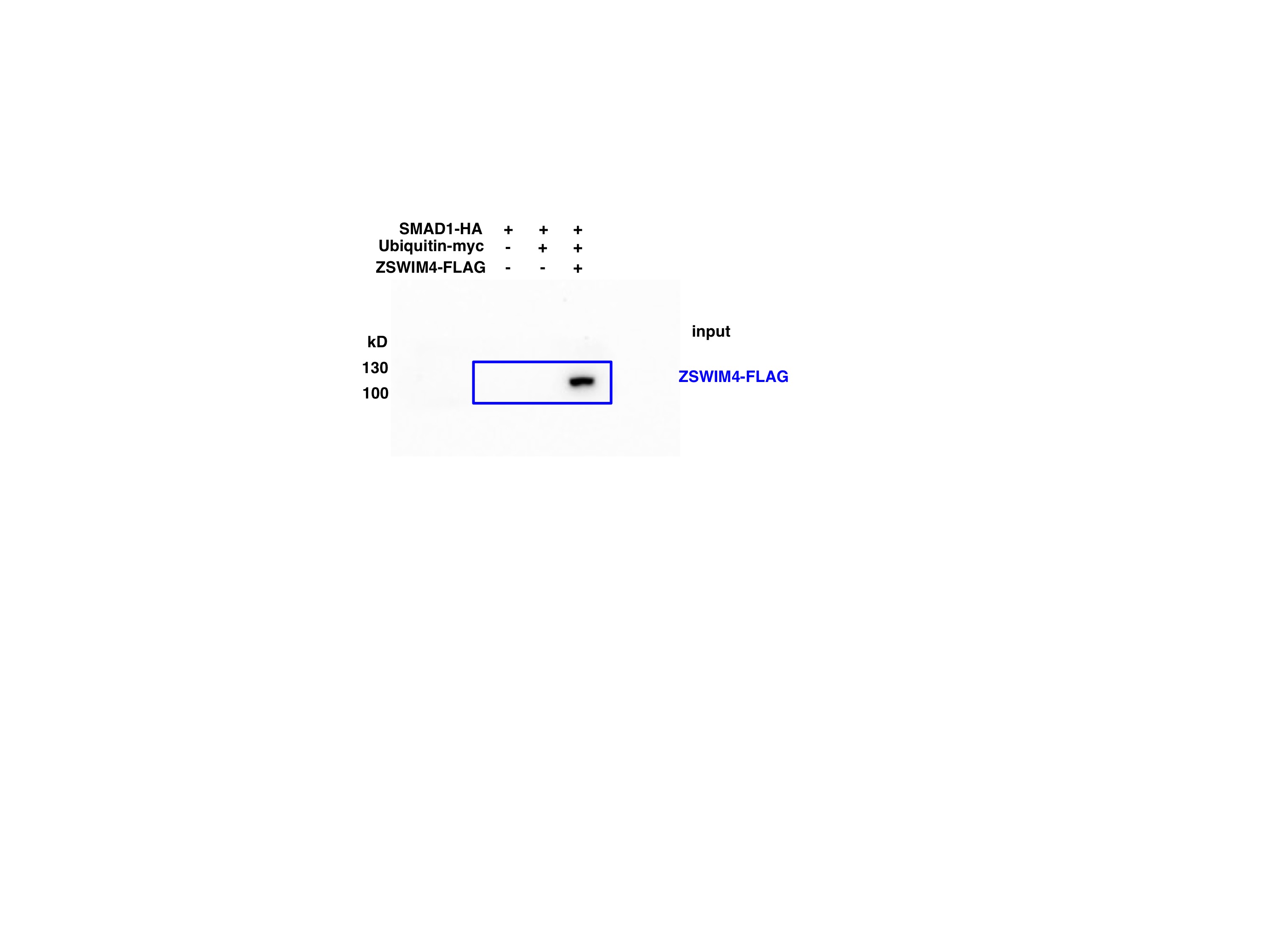

Supplement: Supplementary file 6 — Source Data Fig. 5 [file 44319_2023_46_MOESM6_ESM.zip › Figure 5/5F/western 5F Zswim4.jpg]

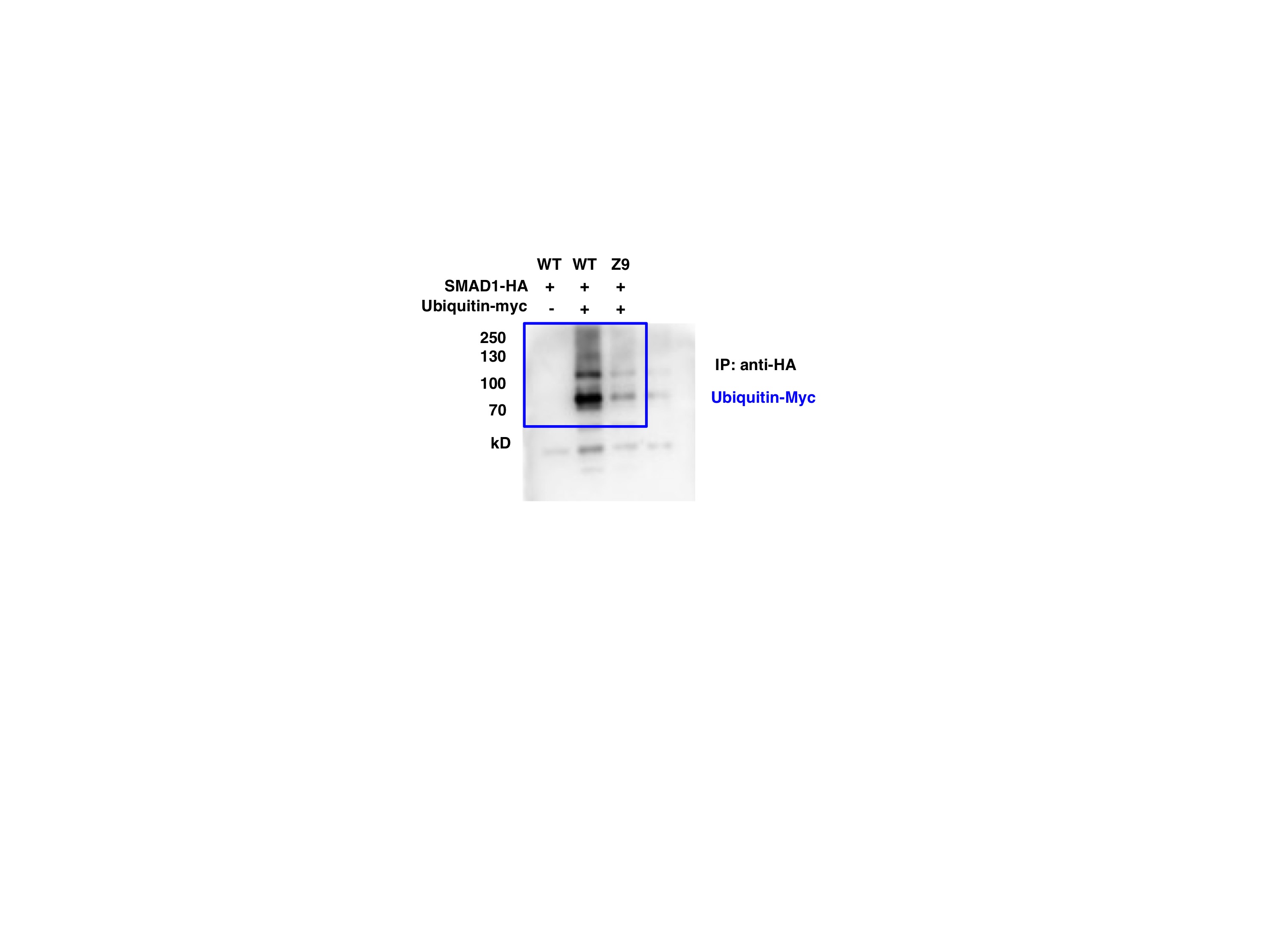

Supplement: Supplementary file 6 — Source Data Fig. 5 [file 44319_2023_46_MOESM6_ESM.zip › Figure 5/5H/western 5H Ubiqutin.jpg]

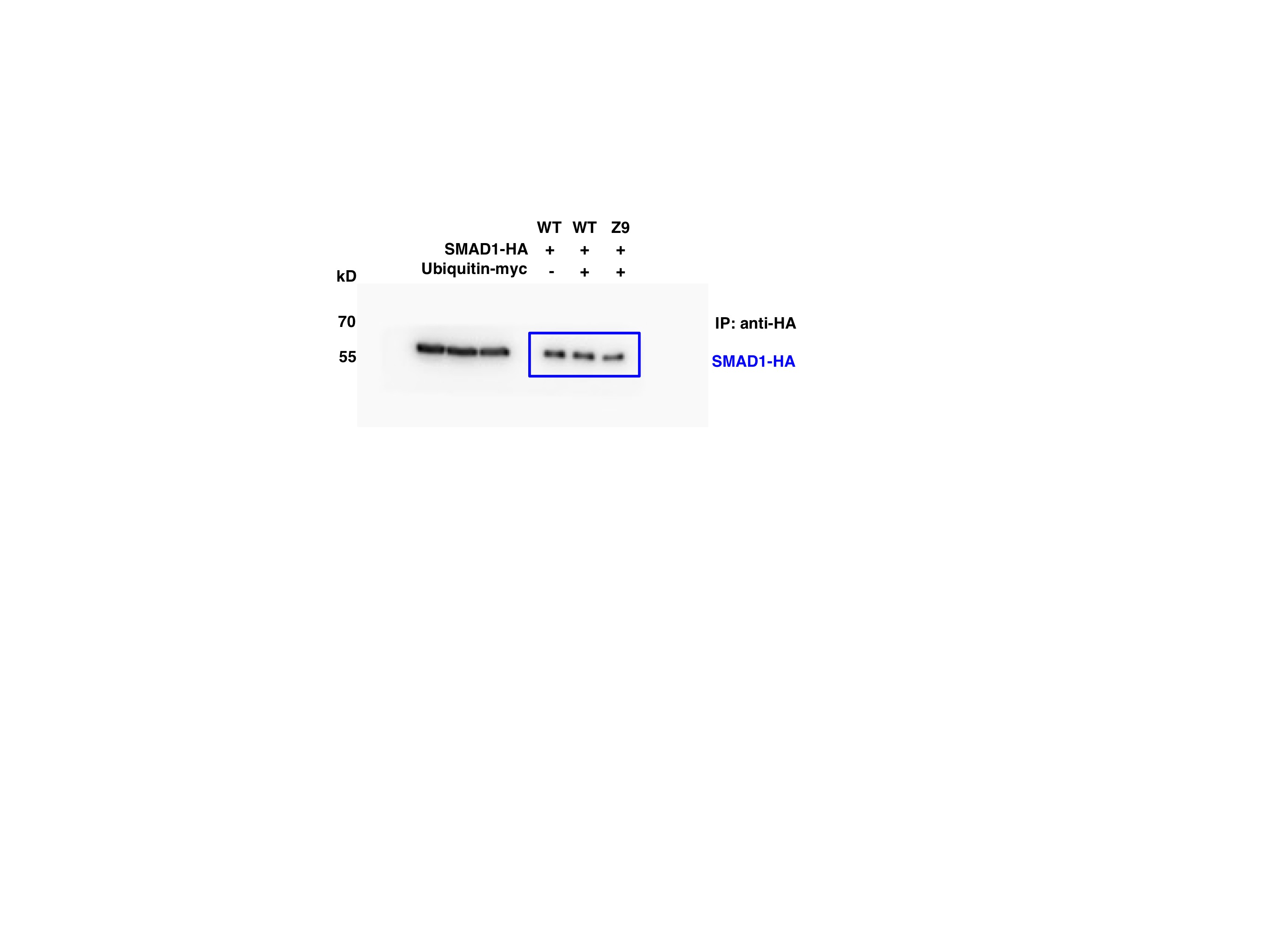

Supplement: Supplementary file 6 — Source Data Fig. 5 [file 44319_2023_46_MOESM6_ESM.zip › Figure 5/5H/western 5H Smad1.jpg]

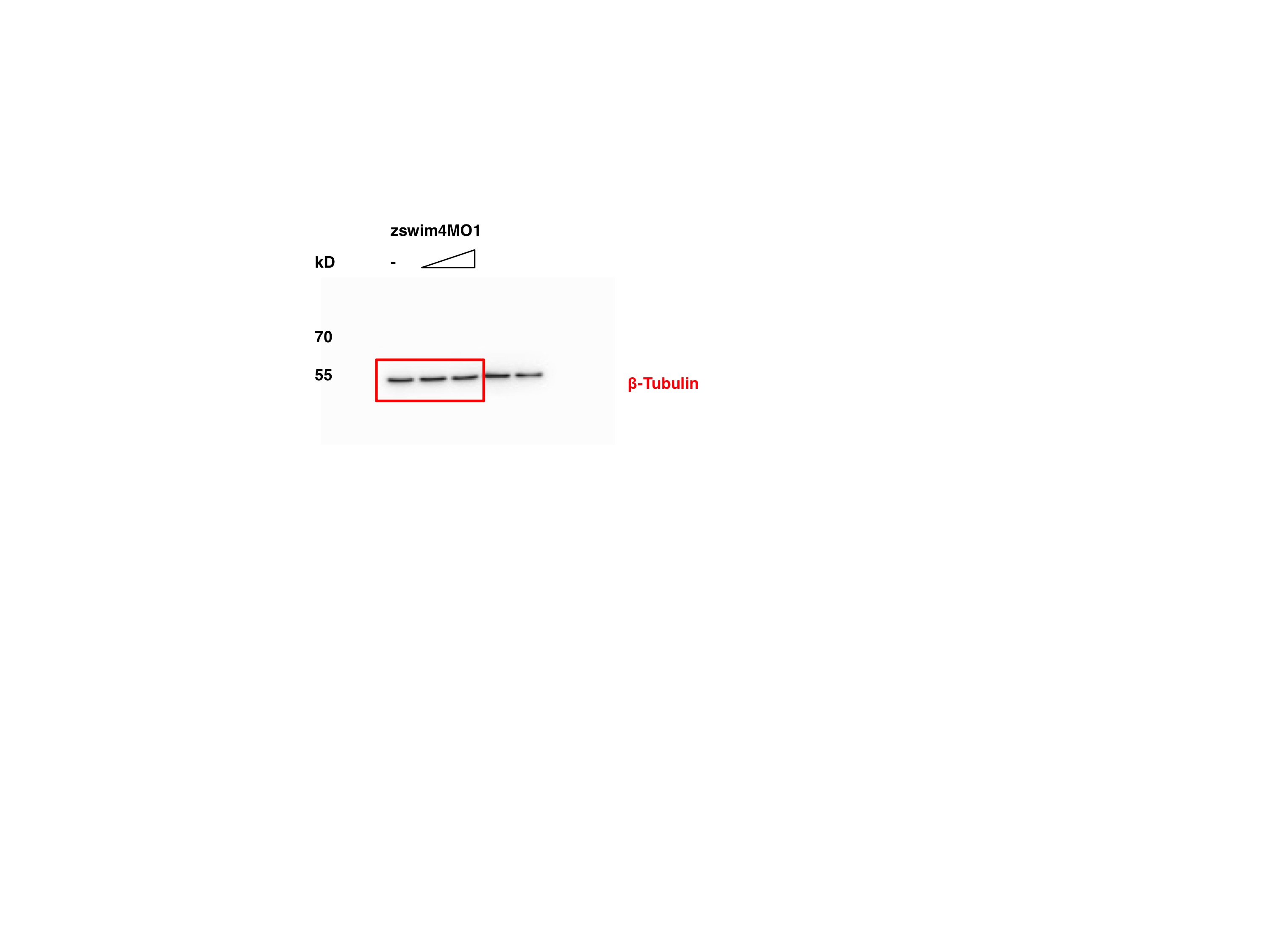

Supplement: Supplementary file 6 — Source Data Fig. 5 [file 44319_2023_46_MOESM6_ESM.zip › Figure 5/5C/western 5C Tubulin.jpg]

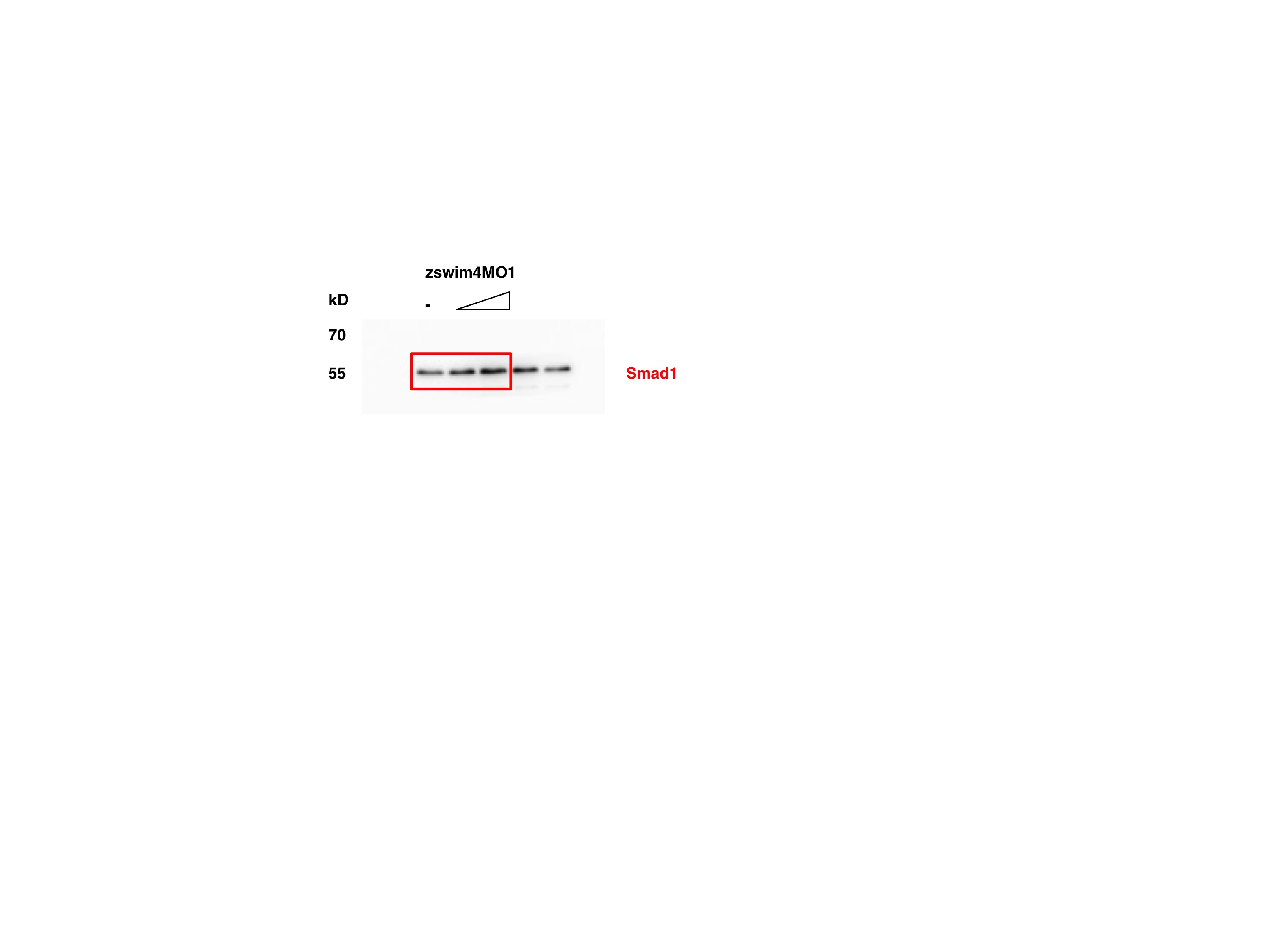

Supplement: Supplementary file 6 — Source Data Fig. 5 [file 44319_2023_46_MOESM6_ESM.zip › Figure 5/5C/western 5C Smad1.jpg]

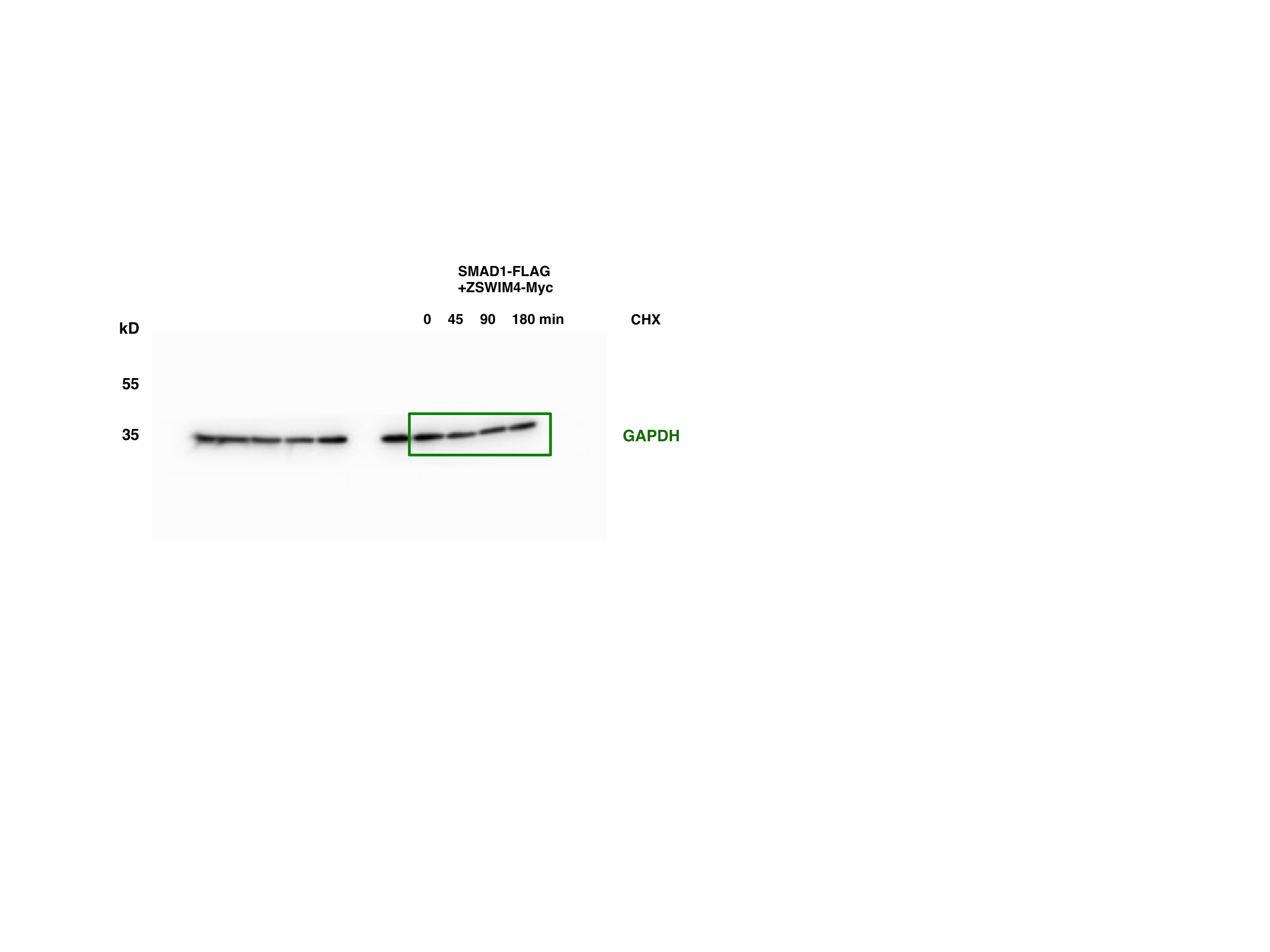

Supplement: Supplementary file 6 — Source Data Fig. 5 [file 44319_2023_46_MOESM6_ESM.zip › Figure 5/5D/western 5D GAPDH-2.jpg]

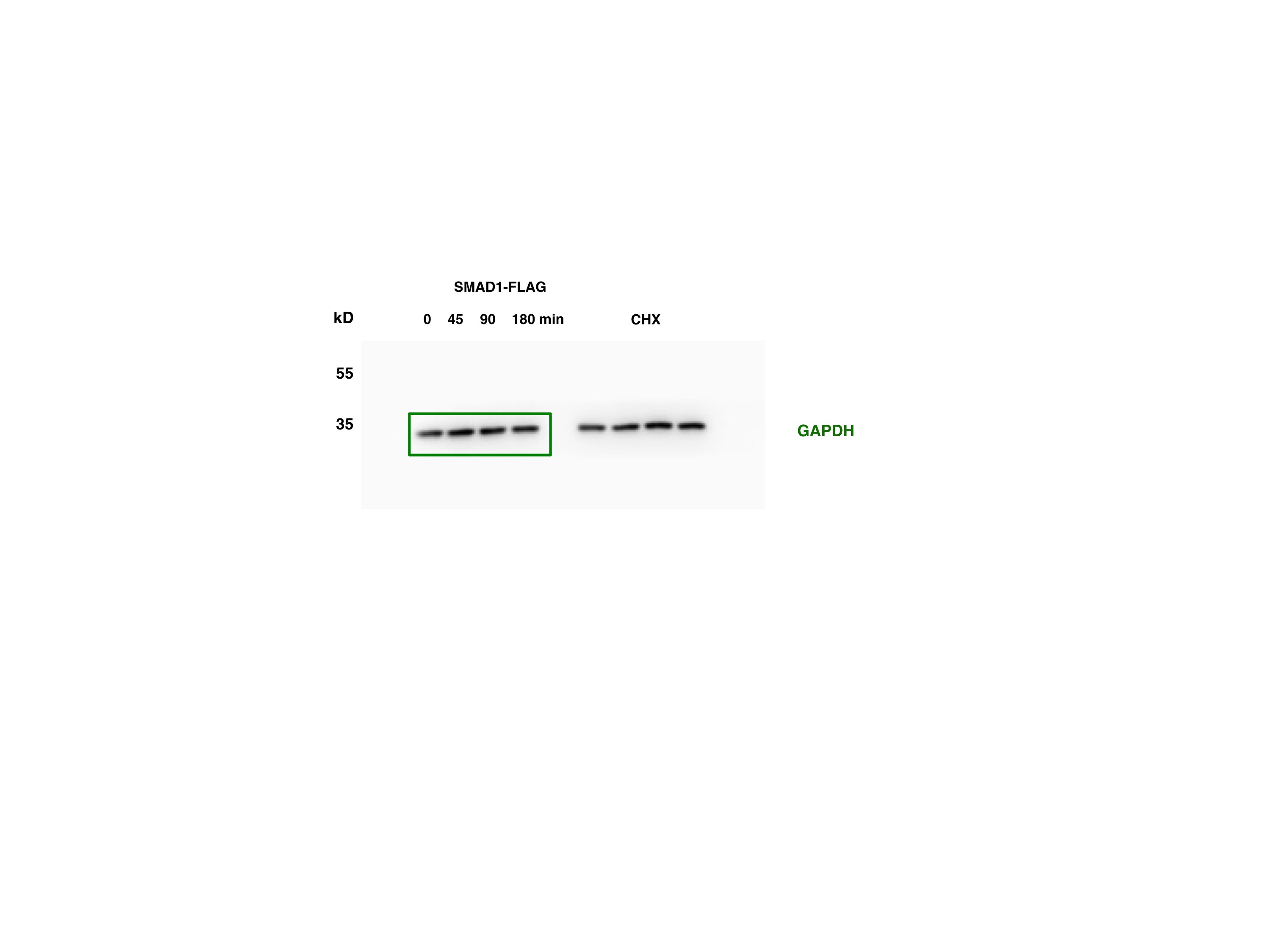

Supplement: Supplementary file 6 — Source Data Fig. 5 [file 44319_2023_46_MOESM6_ESM.zip › Figure 5/5D/western 5D GAPDH-1.jpg]

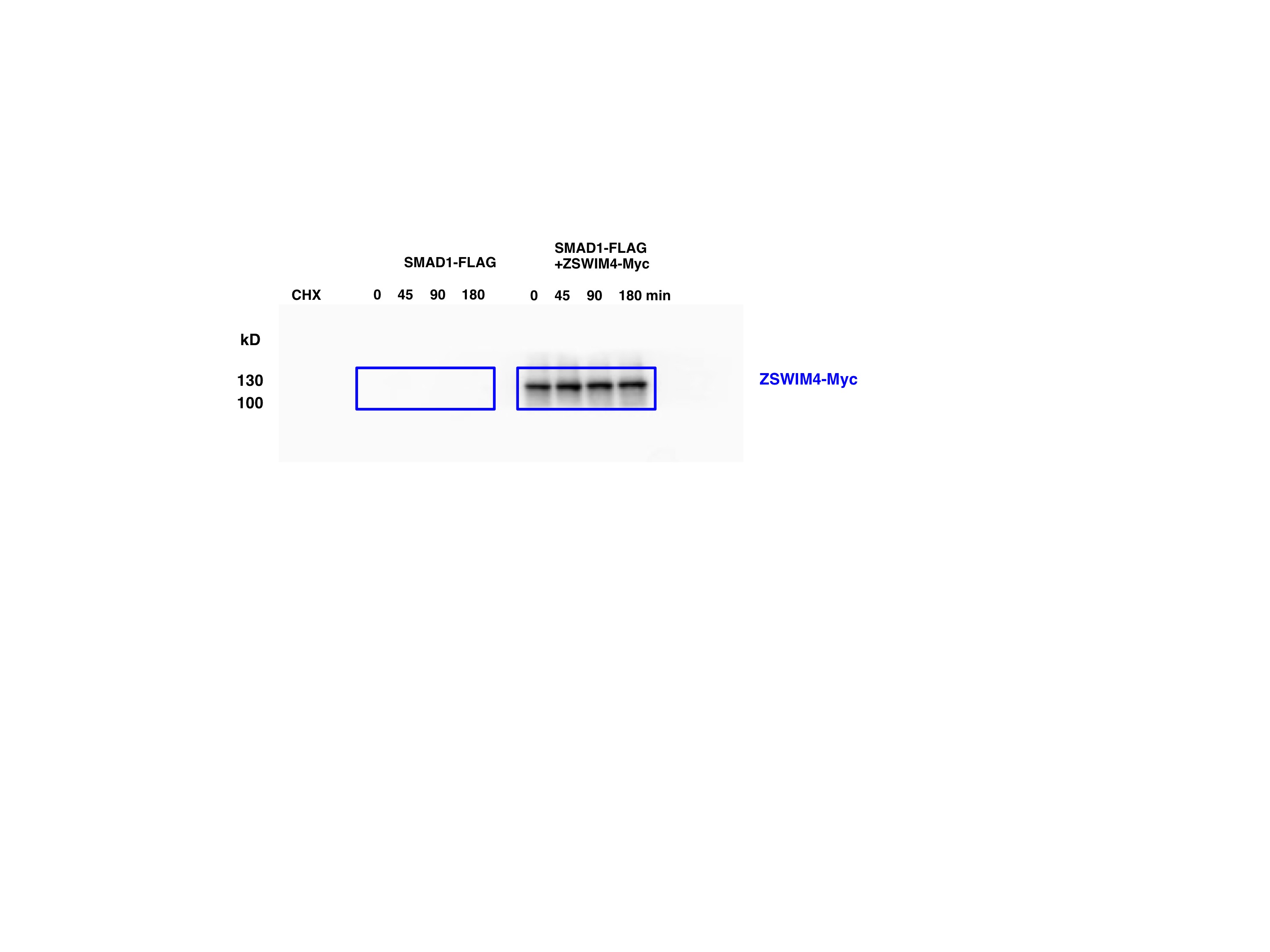

Supplement: Supplementary file 6 — Source Data Fig. 5 [file 44319_2023_46_MOESM6_ESM.zip › Figure 5/5D/western 5D Zswim4.jpg]

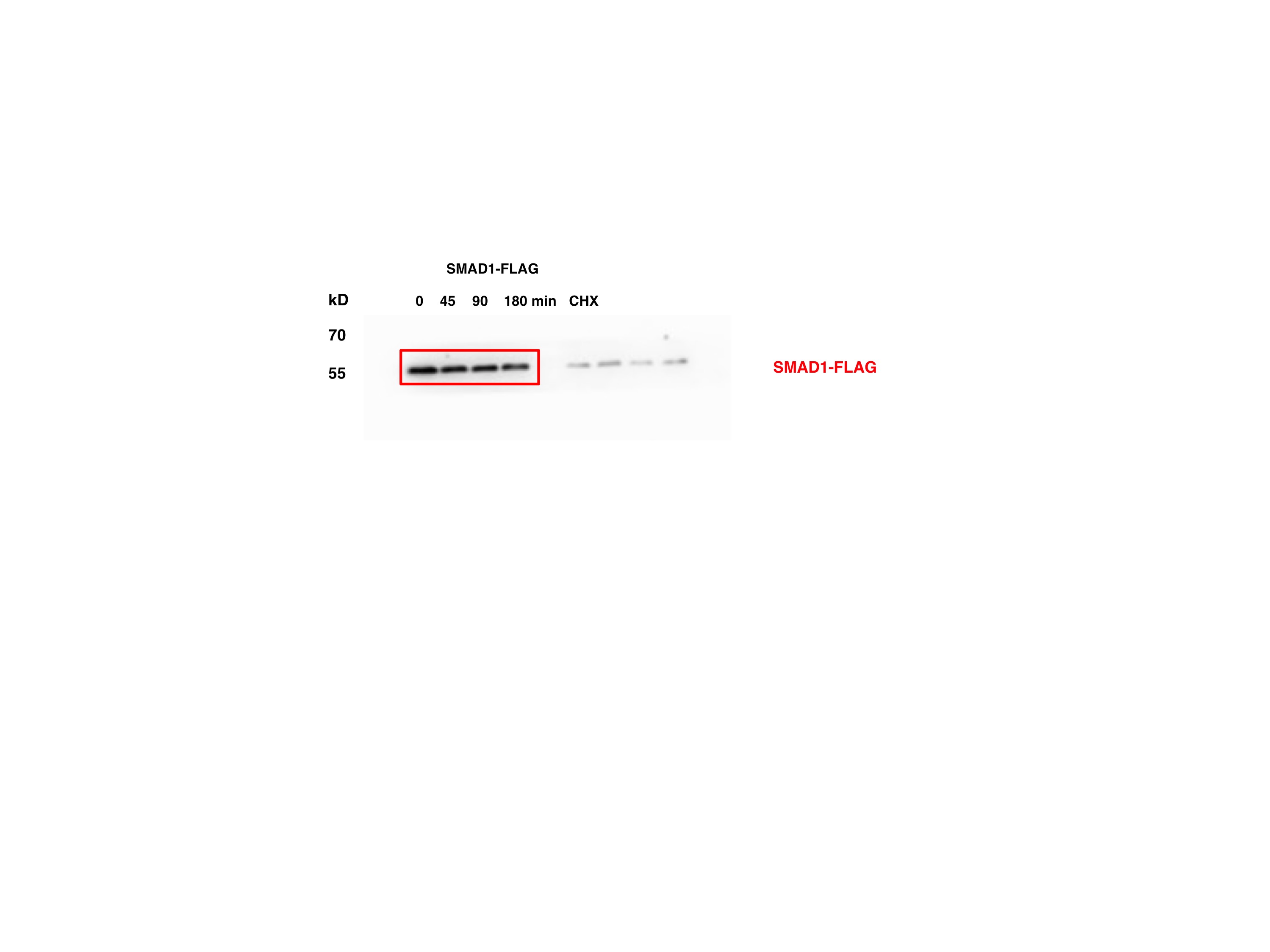

Supplement: Supplementary file 6 — Source Data Fig. 5 [file 44319_2023_46_MOESM6_ESM.zip › Figure 5/5D/western 5D Smad1-1.jpg]

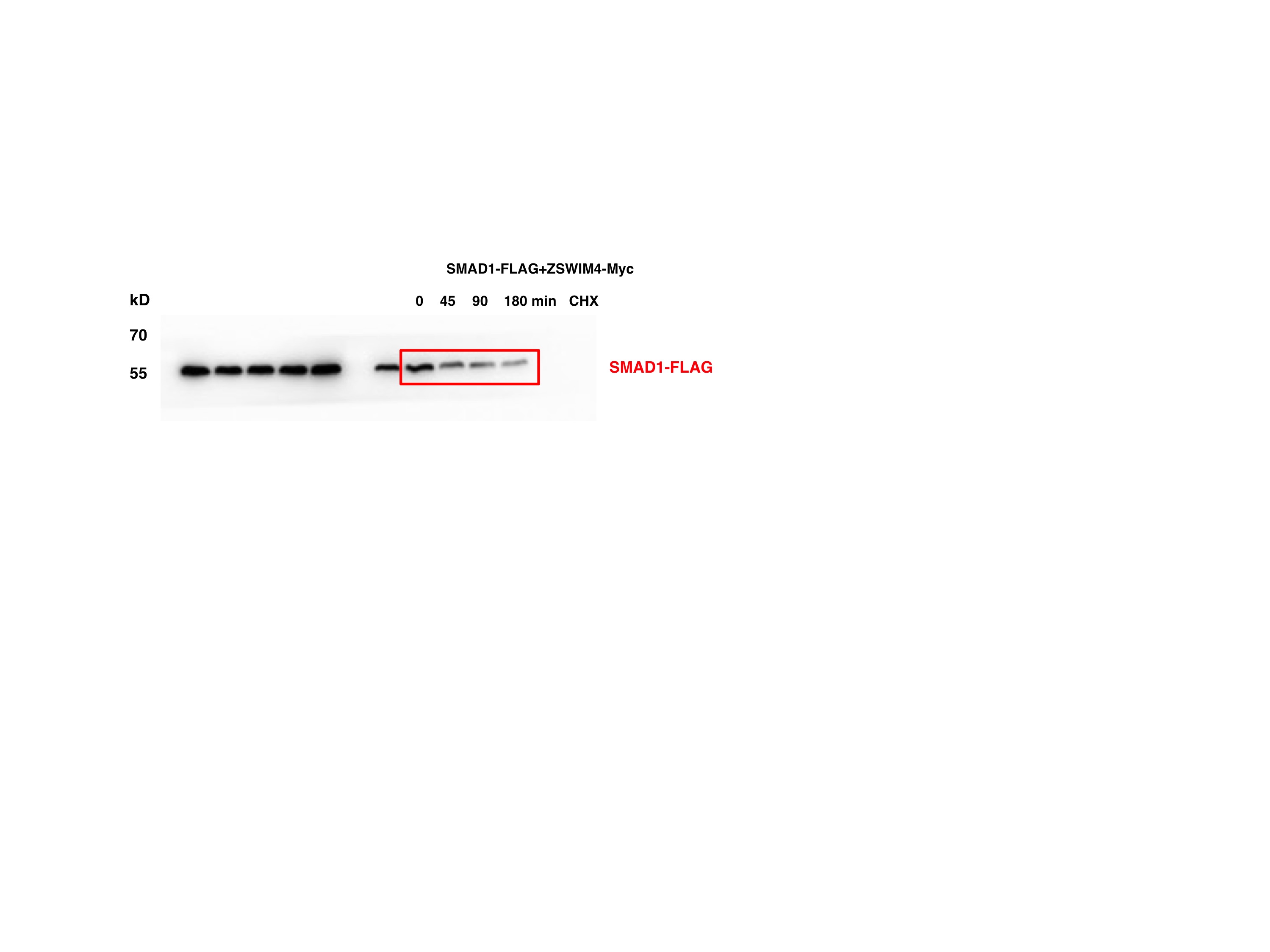

Supplement: Supplementary file 6 — Source Data Fig. 5 [file 44319_2023_46_MOESM6_ESM.zip › Figure 5/5D/western 5D Smad1-2.jpg]
