# Supplementary figures and images for: ZSWIM4 regulates embryonic patterning and BMP signaling by promoting nuclear Smad1 degradation (part 2 of 2)
Source: EMBO Rep. 2024 Jan 2;25(2):14. doi: 10.1038/s44319-023-00046-w (PMC10897318; doi:10.1038/s44319-023-00046-w)

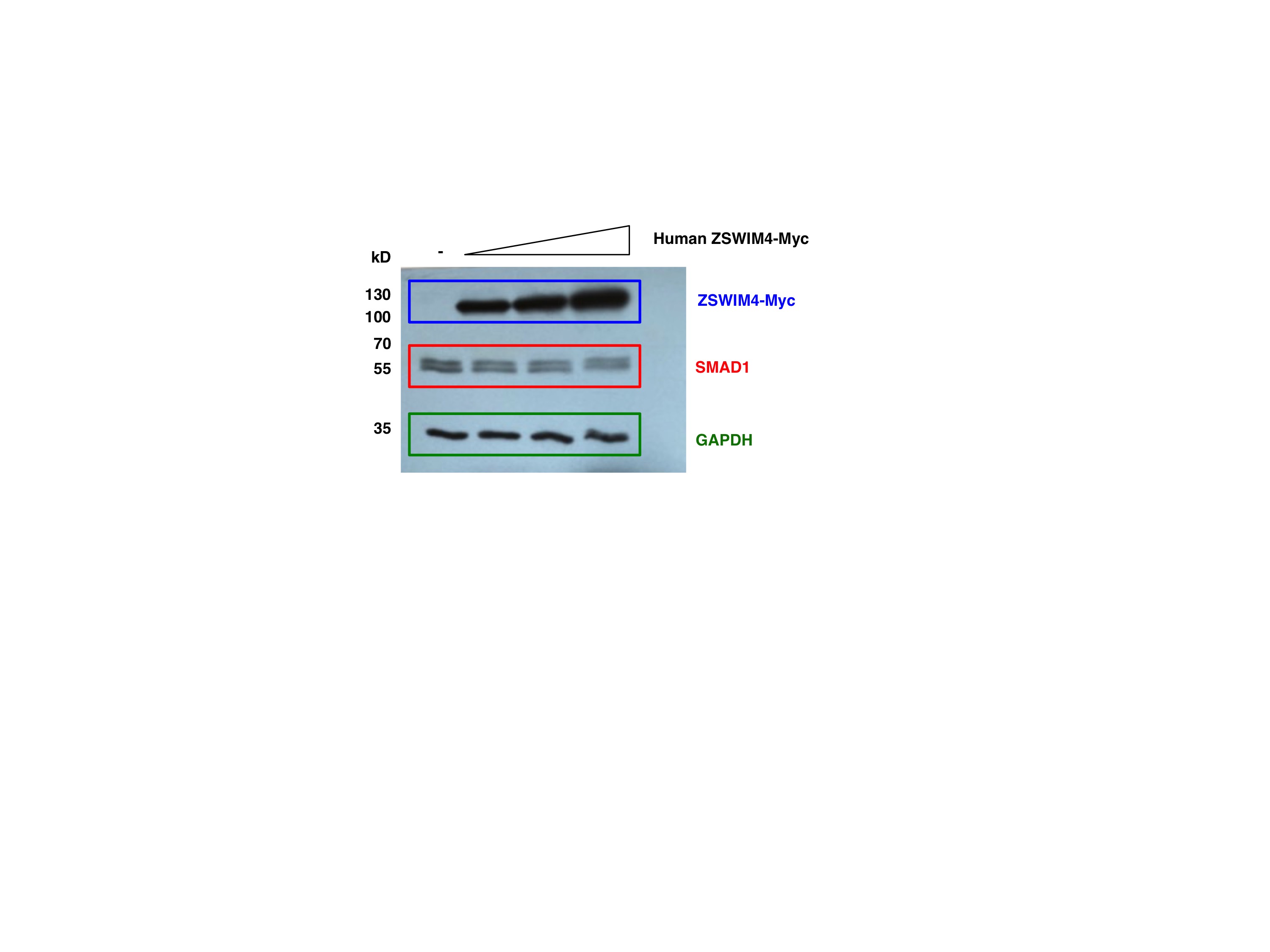

Supplement: Supplementary file 6 — Source Data Fig. 5 [file 44319_2023_46_MOESM6_ESM.zip › Figure 5/5B/western 5B Smad1 Zswim4 GAPDH.jpg]

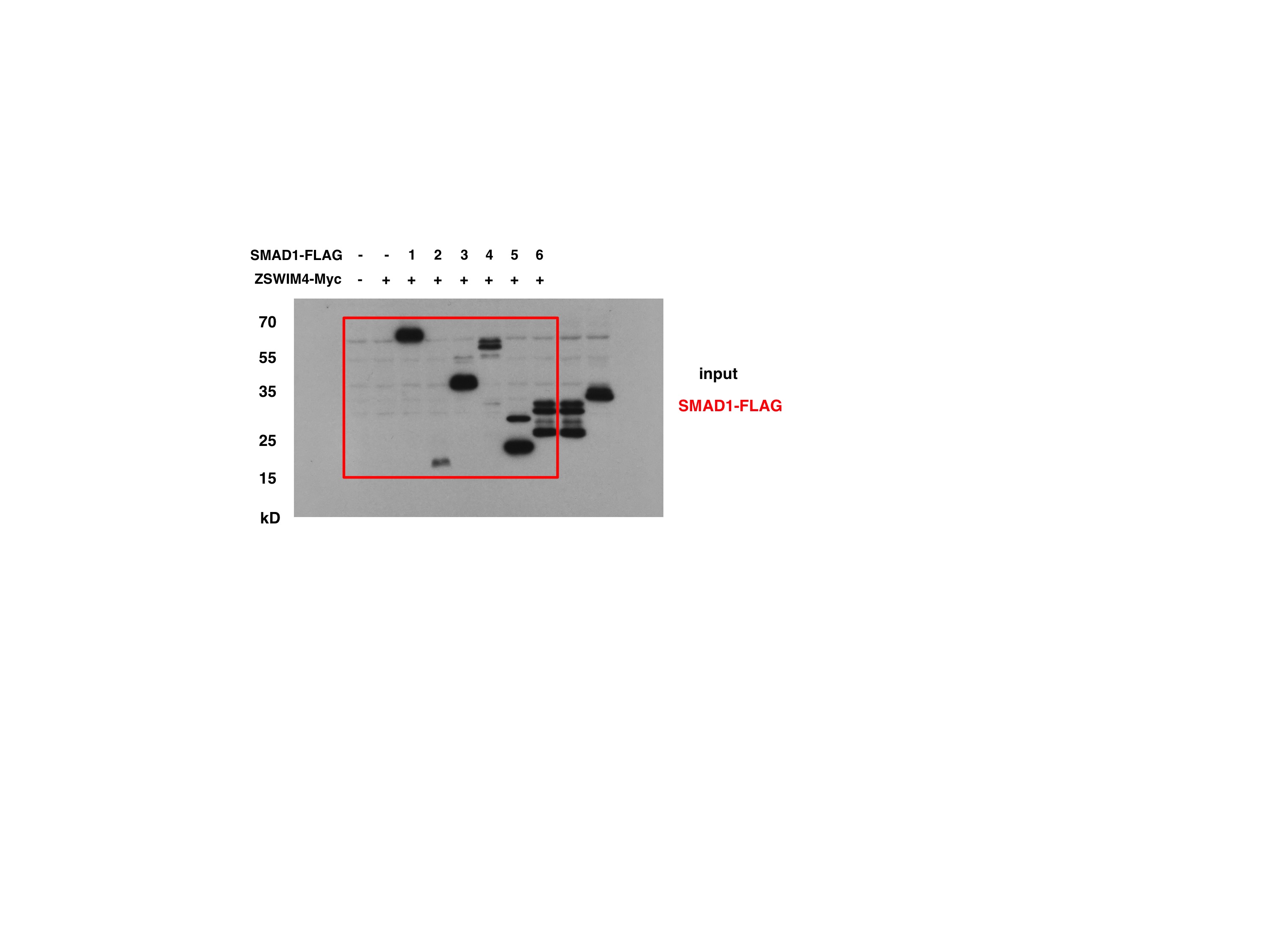

Supplement: Supplementary file 6 — Source Data Fig. 5 [file 44319_2023_46_MOESM6_ESM.zip › Figure 5/5K/western 5K smad1.jpg]

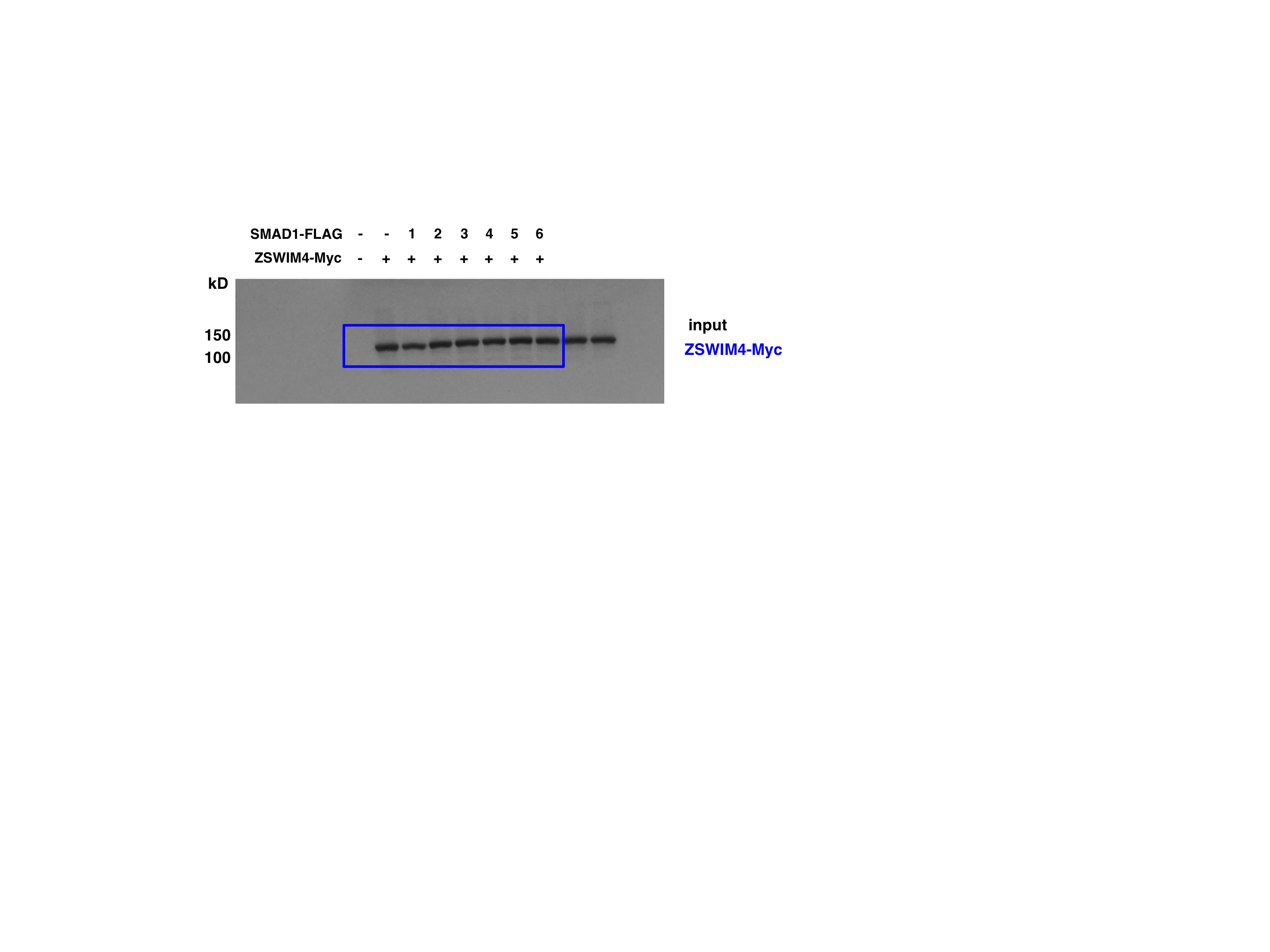

Supplement: Supplementary file 6 — Source Data Fig. 5 [file 44319_2023_46_MOESM6_ESM.zip › Figure 5/5K/western 5K zswim4-2.jpg]

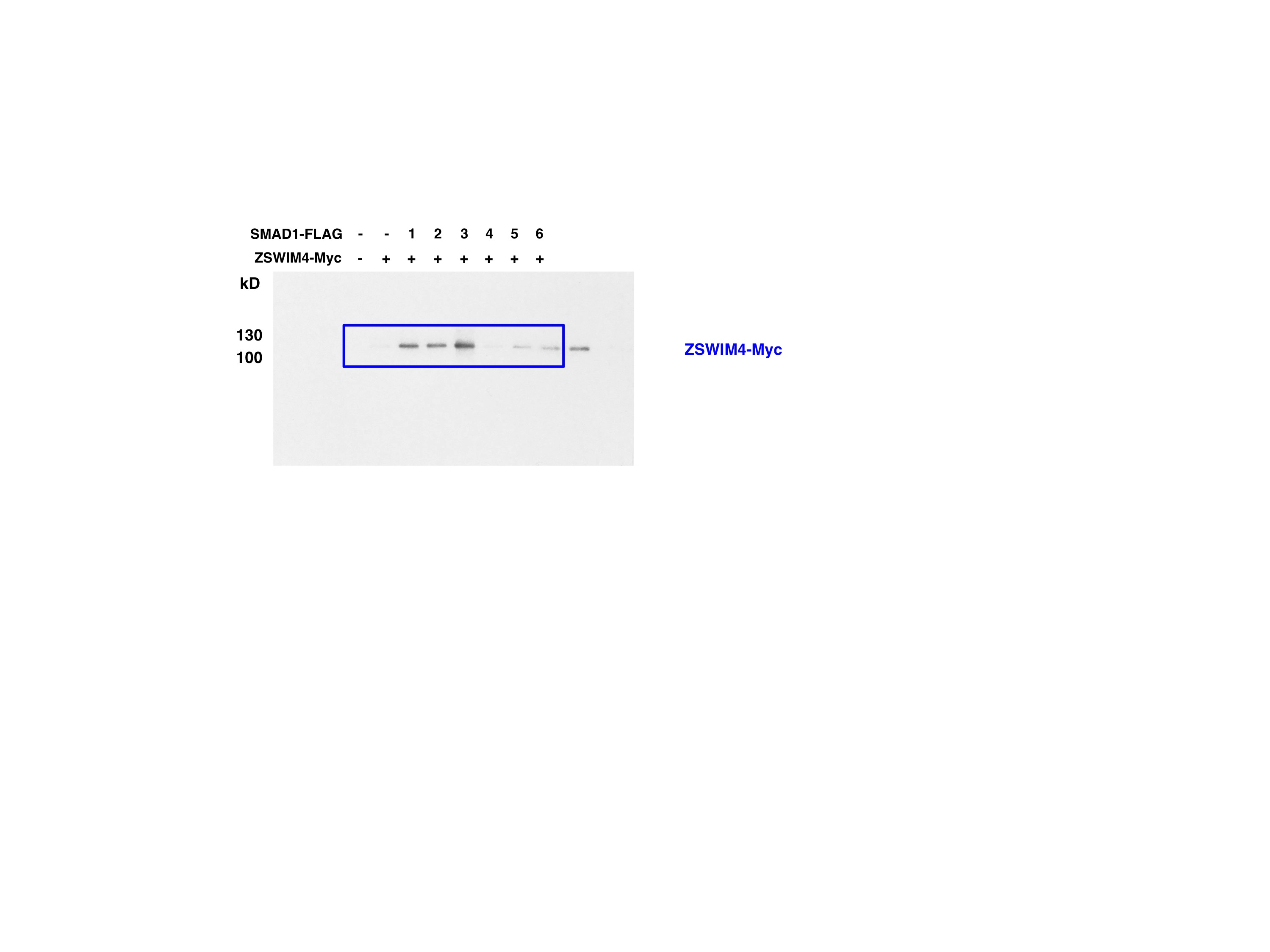

Supplement: Supplementary file 6 — Source Data Fig. 5 [file 44319_2023_46_MOESM6_ESM.zip › Figure 5/5K/western 5K zswim4.jpg]

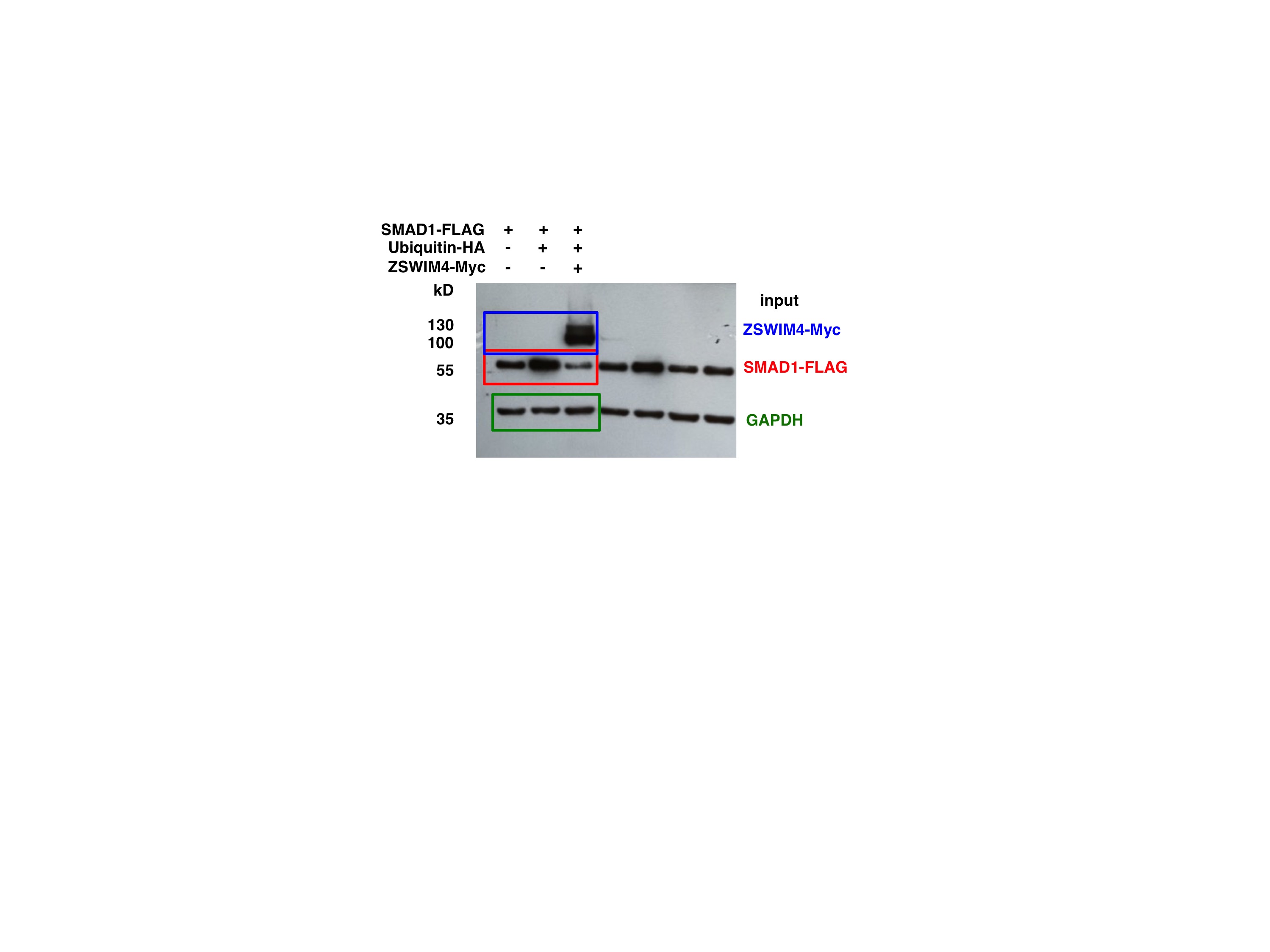

Supplement: Supplementary file 6 — Source Data Fig. 5 [file 44319_2023_46_MOESM6_ESM.zip › Figure 5/5F/replicate/western 5F GAPDH Smad1 Zswim4 replicate.jpg]

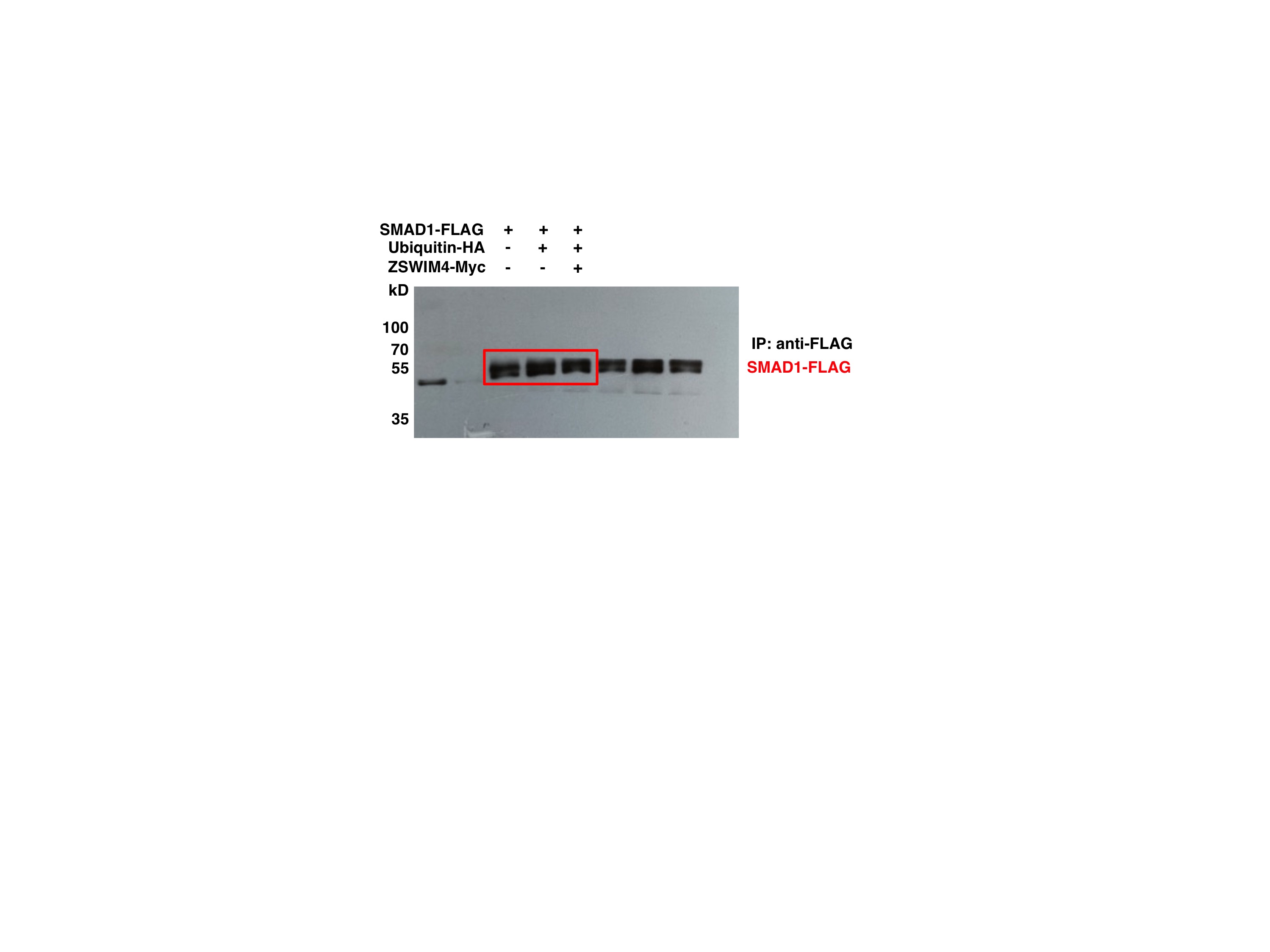

Supplement: Supplementary file 6 — Source Data Fig. 5 [file 44319_2023_46_MOESM6_ESM.zip › Figure 5/5F/replicate/western 5F Smad1-2 replicate.jpg]

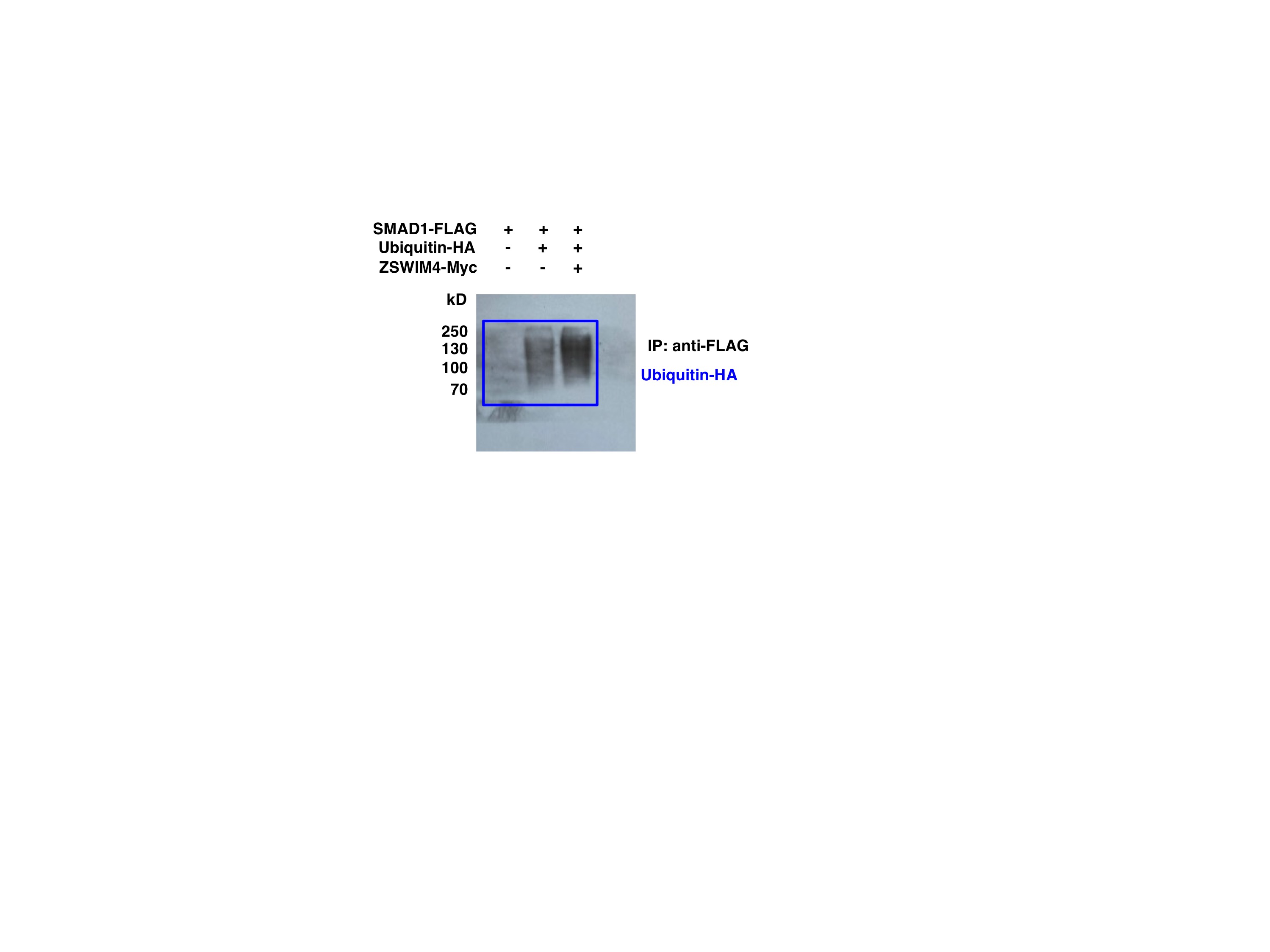

Supplement: Supplementary file 6 — Source Data Fig. 5 [file 44319_2023_46_MOESM6_ESM.zip › Figure 5/5F/replicate/western 5F Ubiquitin replicate.jpg]

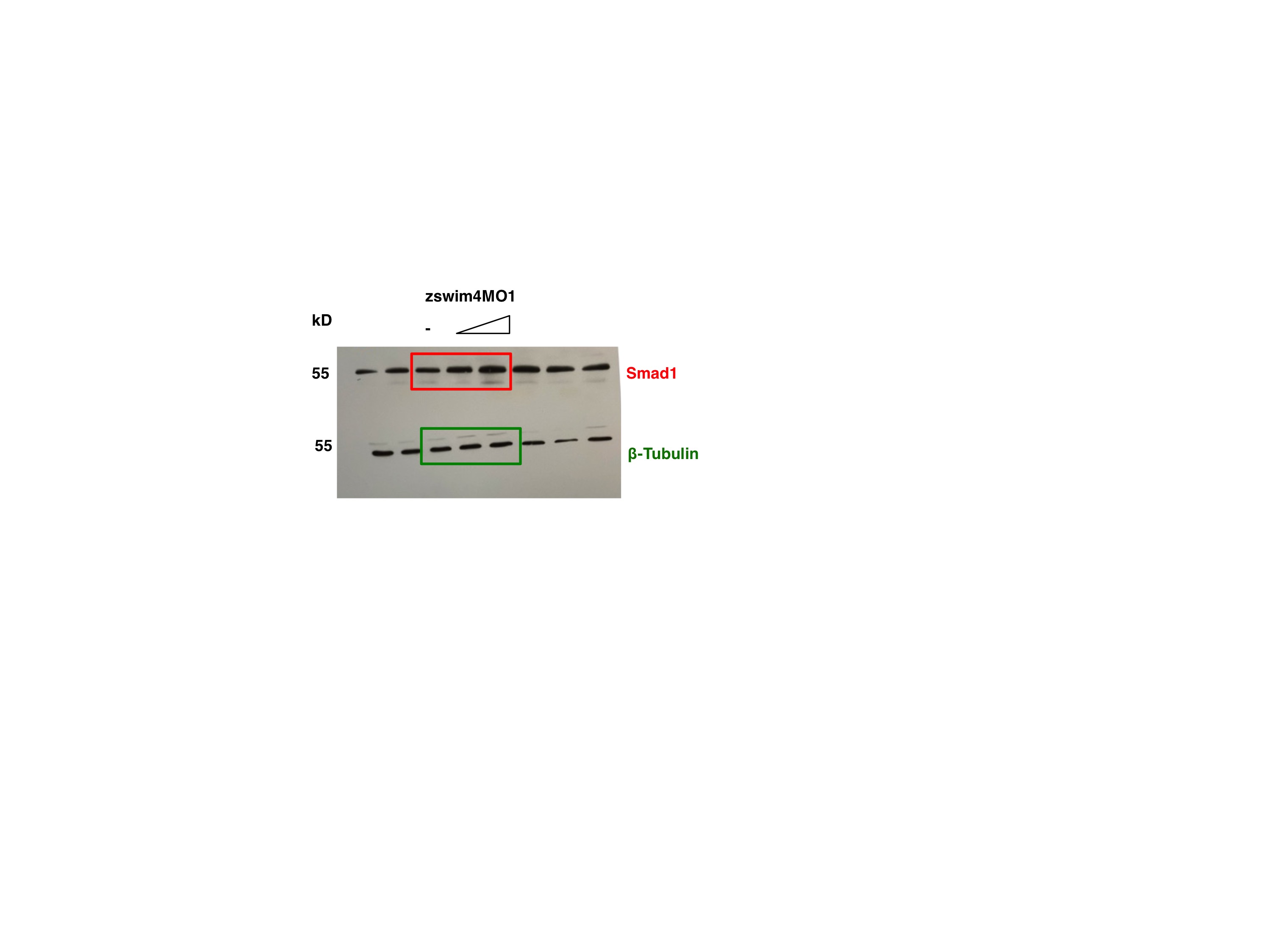

Supplement: Supplementary file 6 — Source Data Fig. 5 [file 44319_2023_46_MOESM6_ESM.zip › Figure 5/5C/replicate/western 5C Tubulin Smad1 replicate.jpg]

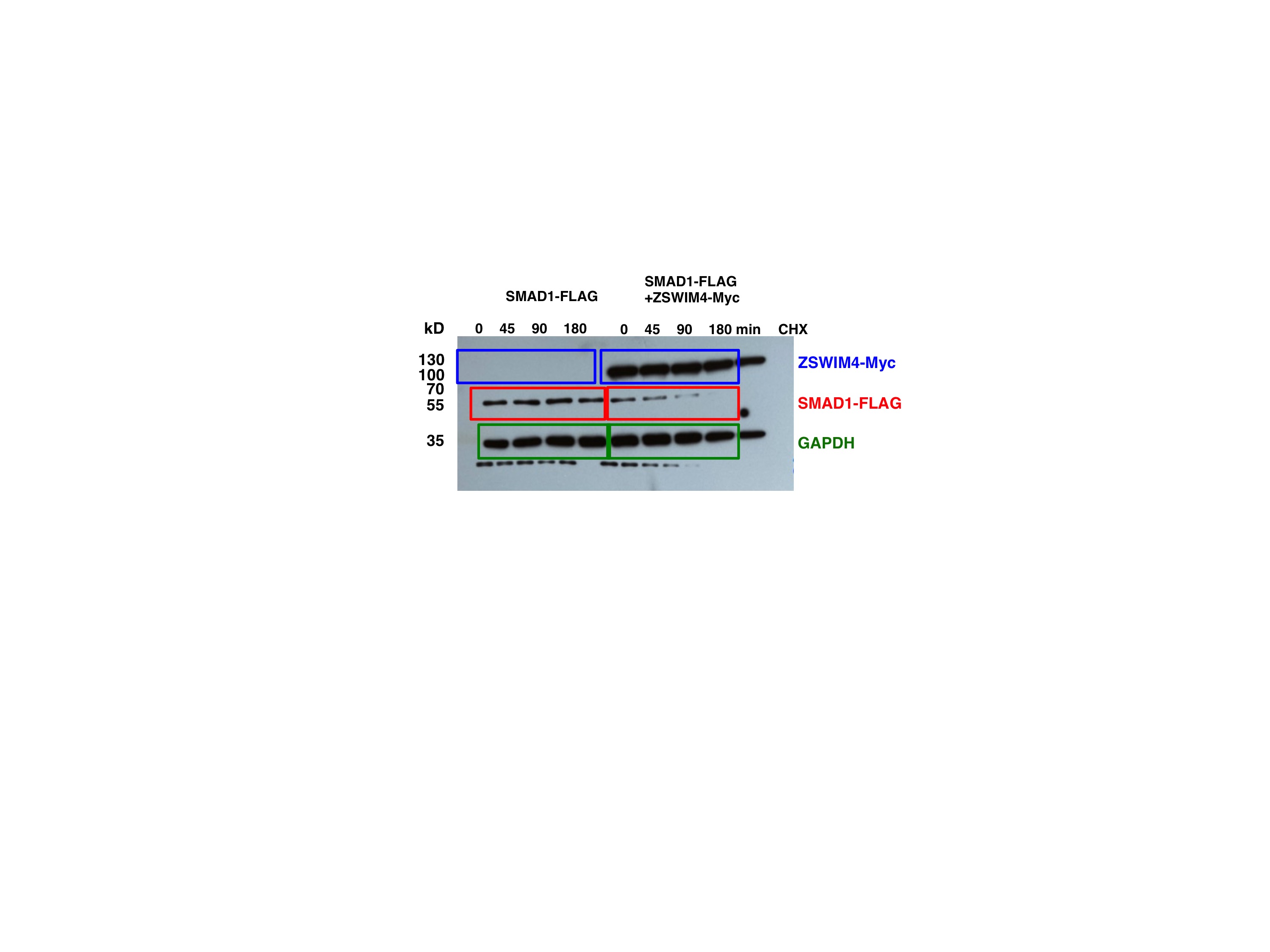

Supplement: Supplementary file 6 — Source Data Fig. 5 [file 44319_2023_46_MOESM6_ESM.zip › Figure 5/5D/replicate/western 5D GAPDH Smad1 Zswim4 replicate.jpg]

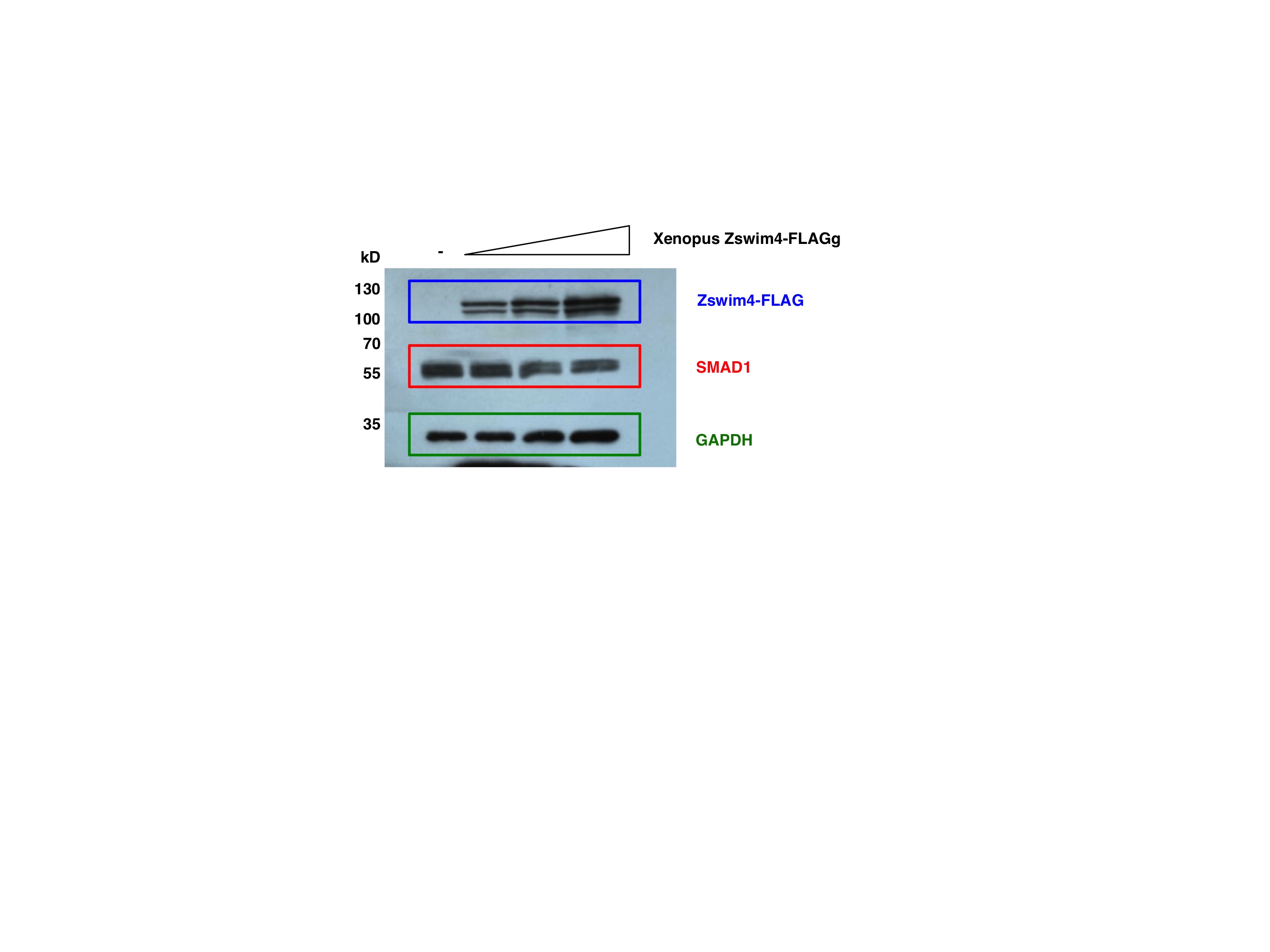

Supplement: Supplementary file 6 — Source Data Fig. 5 [file 44319_2023_46_MOESM6_ESM.zip › Figure 5/5B/replicate/western 5B Smad1 Zswim4 GAPDH replicate.jpg]

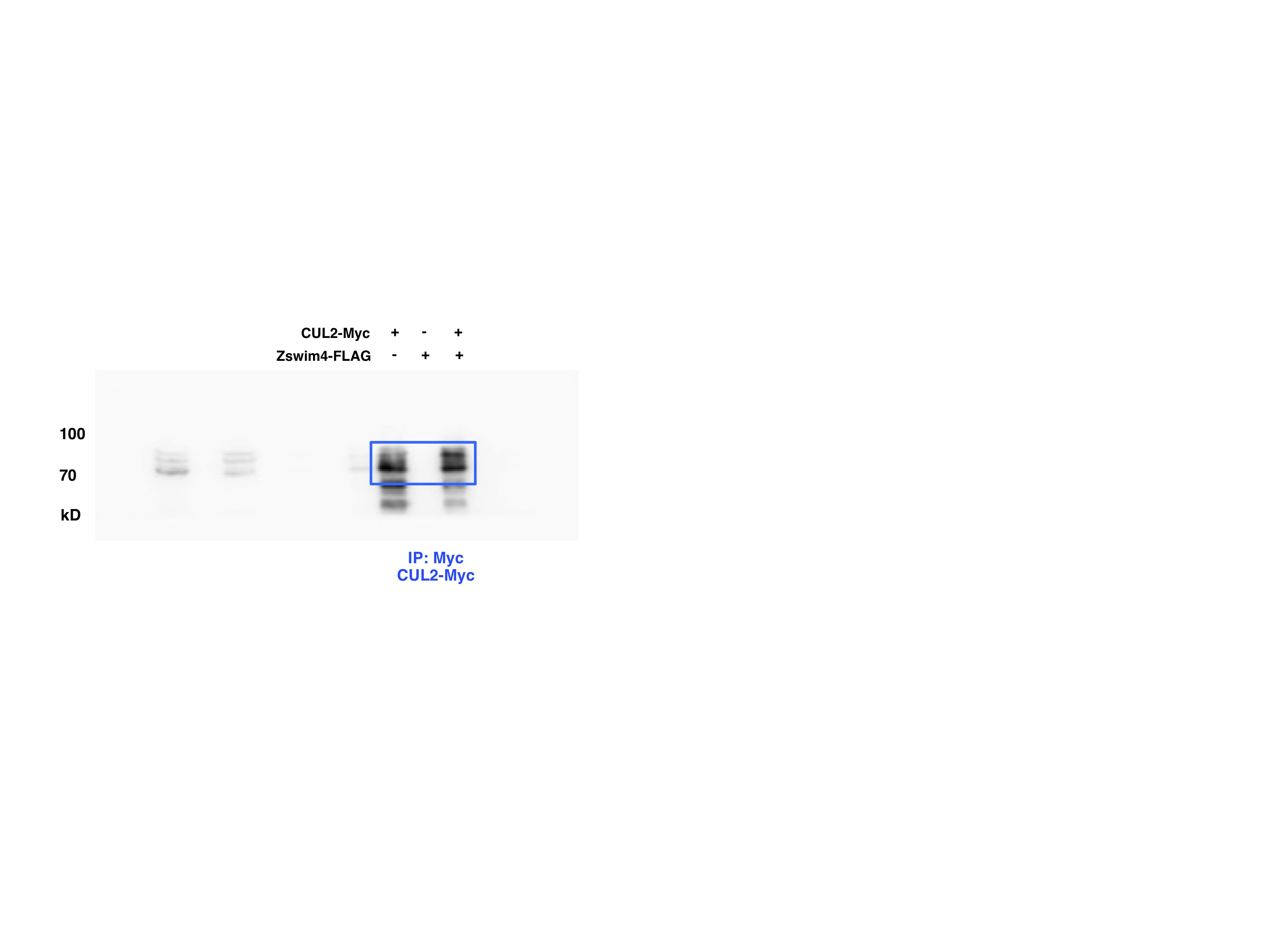

Supplement: Supplementary file 7 — Source Data Fig. 6 [file 44319_2023_46_MOESM7_ESM.zip › Figure 6/6F/western 6F CUL2-2.jpg]

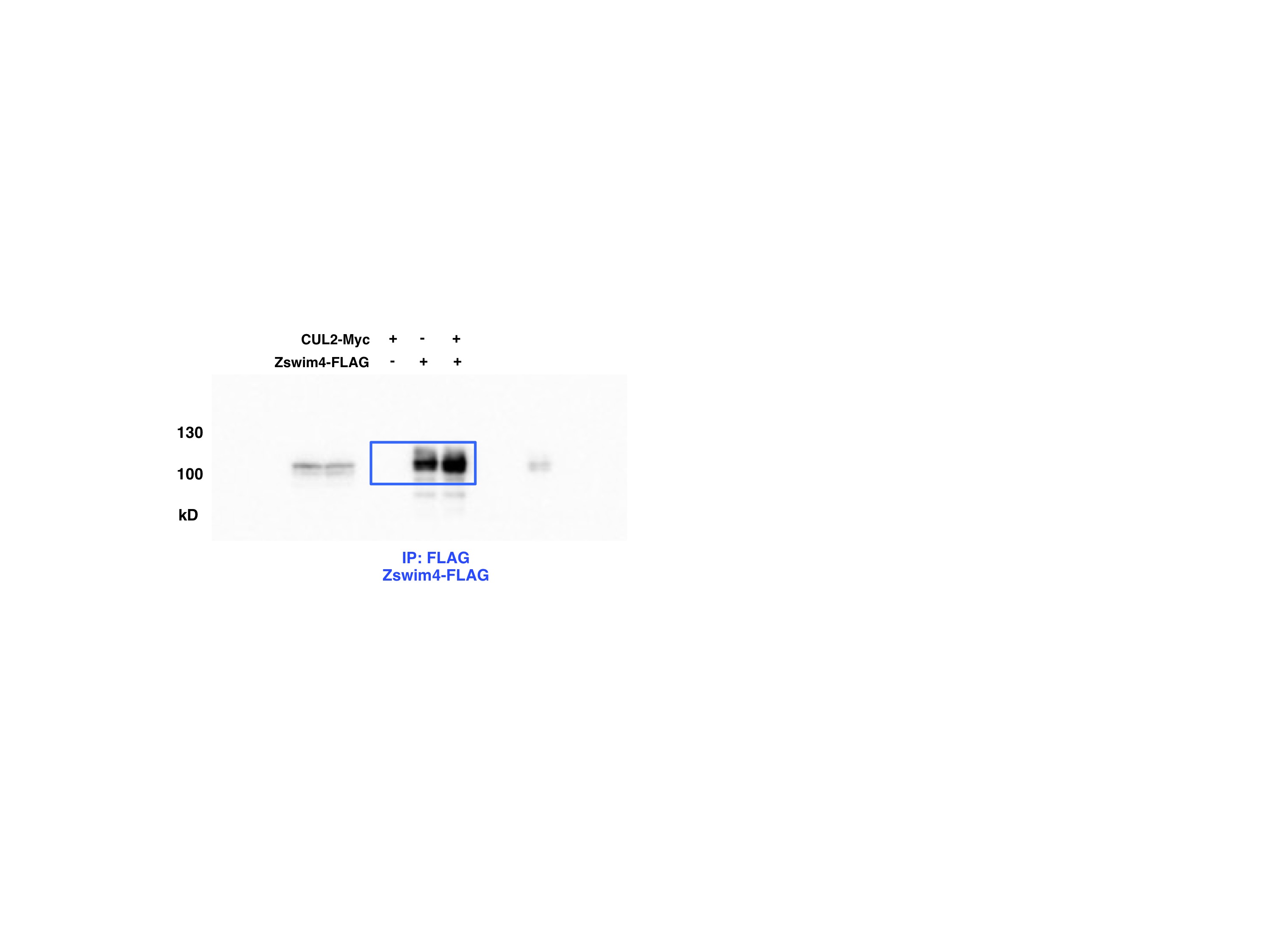

Supplement: Supplementary file 7 — Source Data Fig. 6 [file 44319_2023_46_MOESM7_ESM.zip › Figure 6/6F/western 6F Zswim4.jpg]

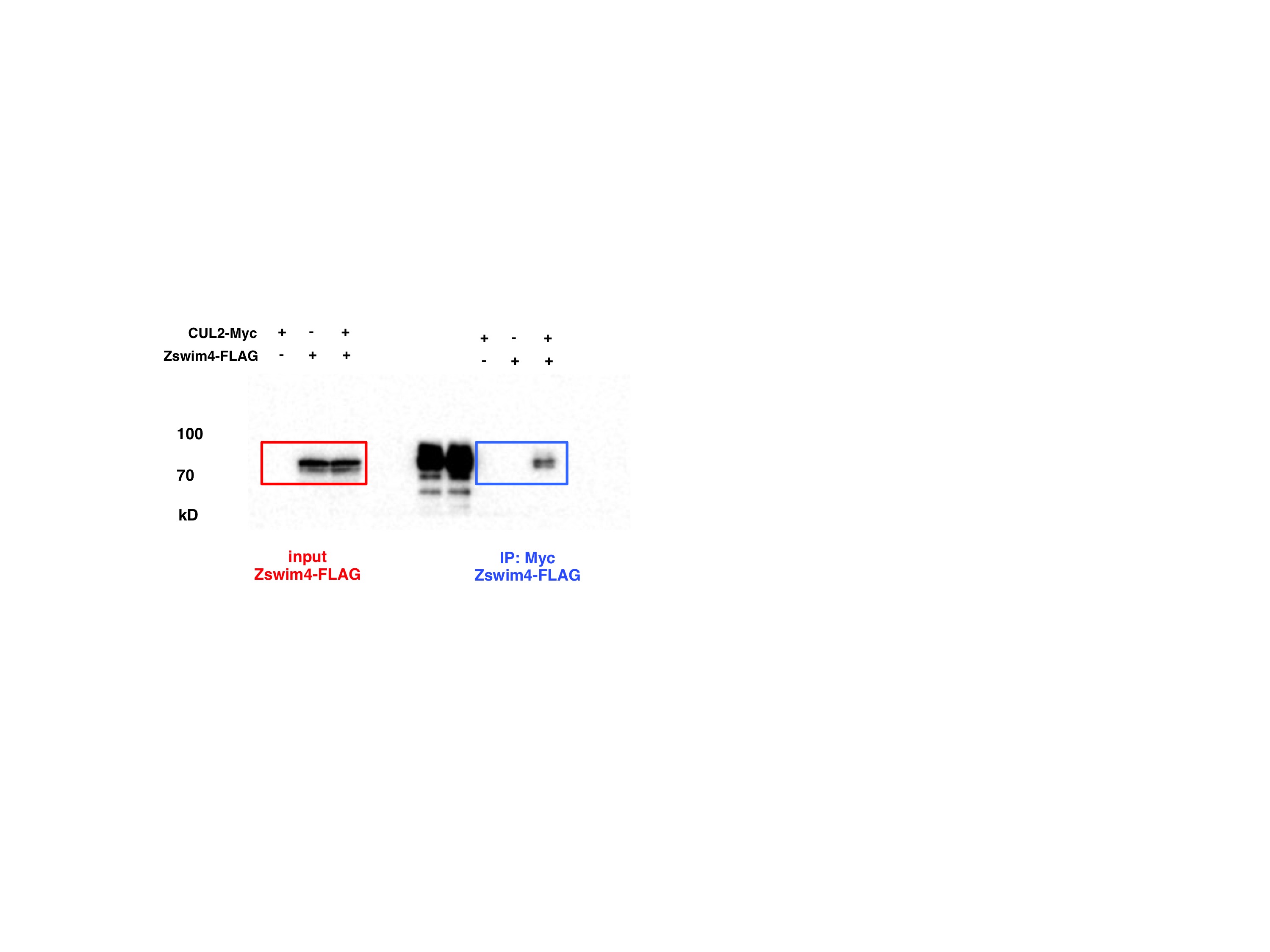

Supplement: Supplementary file 7 — Source Data Fig. 6 [file 44319_2023_46_MOESM7_ESM.zip › Figure 6/6F/western 6F Zswim4-2.jpg]

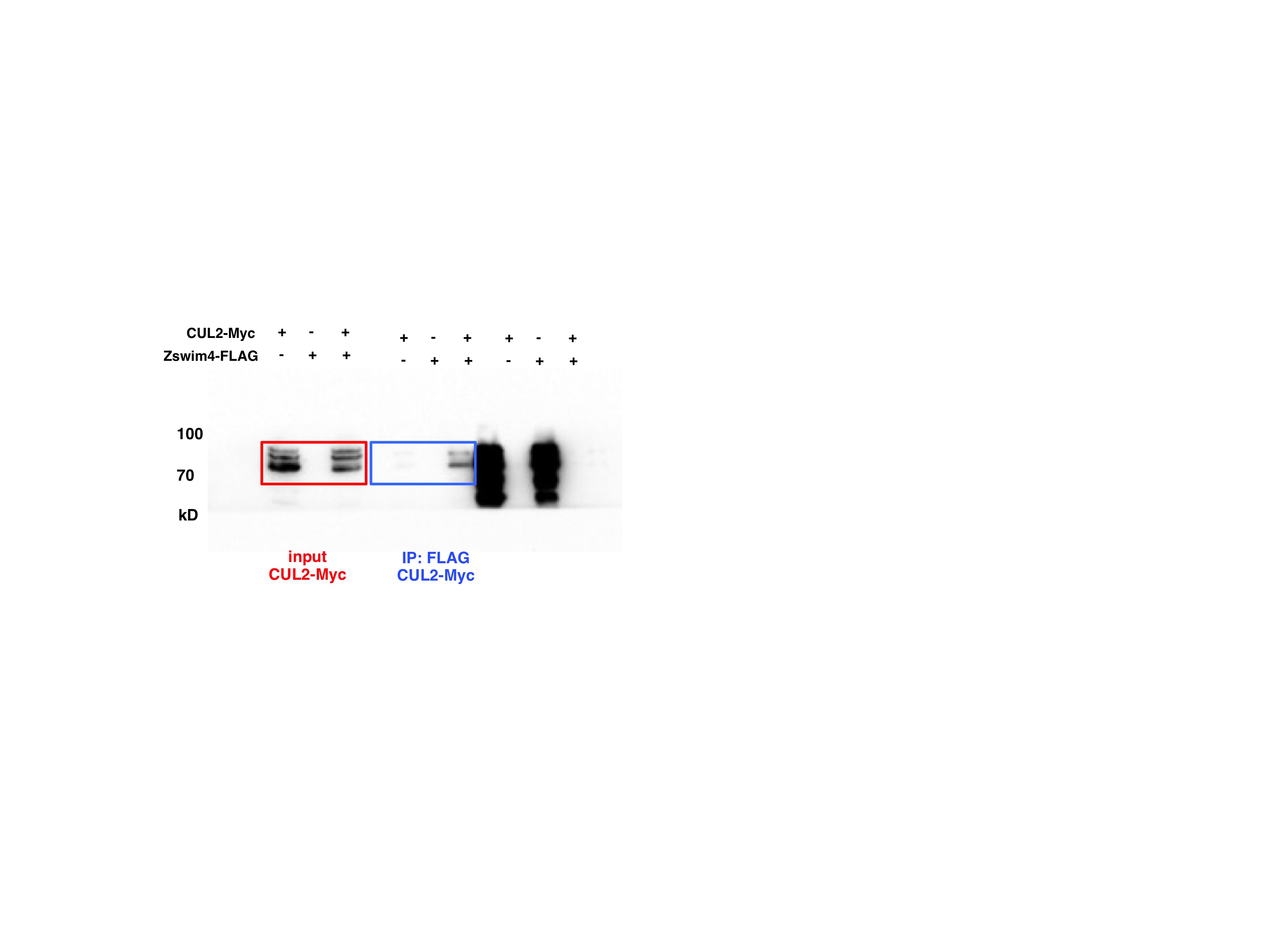

Supplement: Supplementary file 7 — Source Data Fig. 6 [file 44319_2023_46_MOESM7_ESM.zip › Figure 6/6F/western 6F CUL2.jpg]

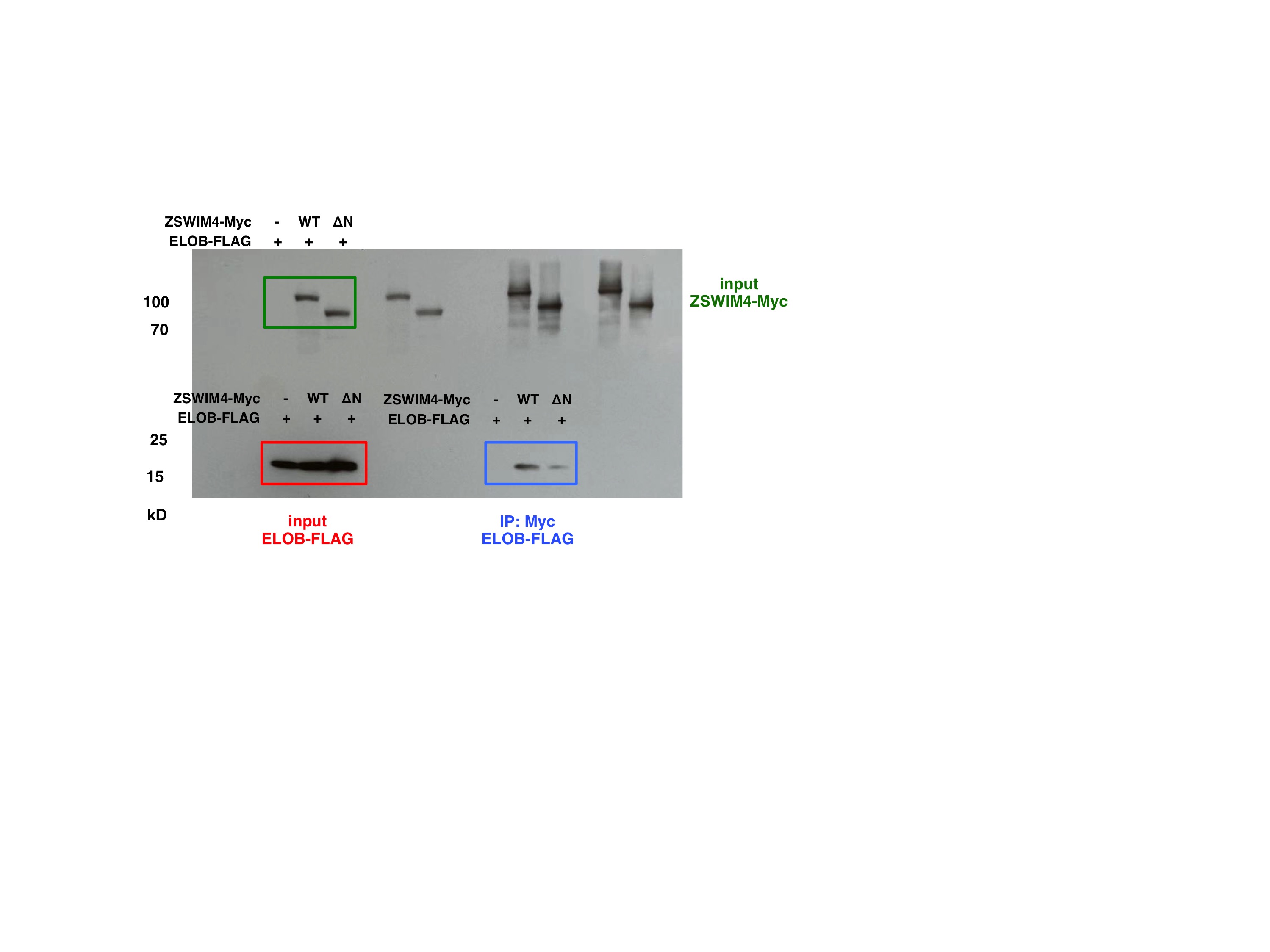

Supplement: Supplementary file 7 — Source Data Fig. 6 [file 44319_2023_46_MOESM7_ESM.zip › Figure 6/6H/western 6H ELOB zswim4.jpg]

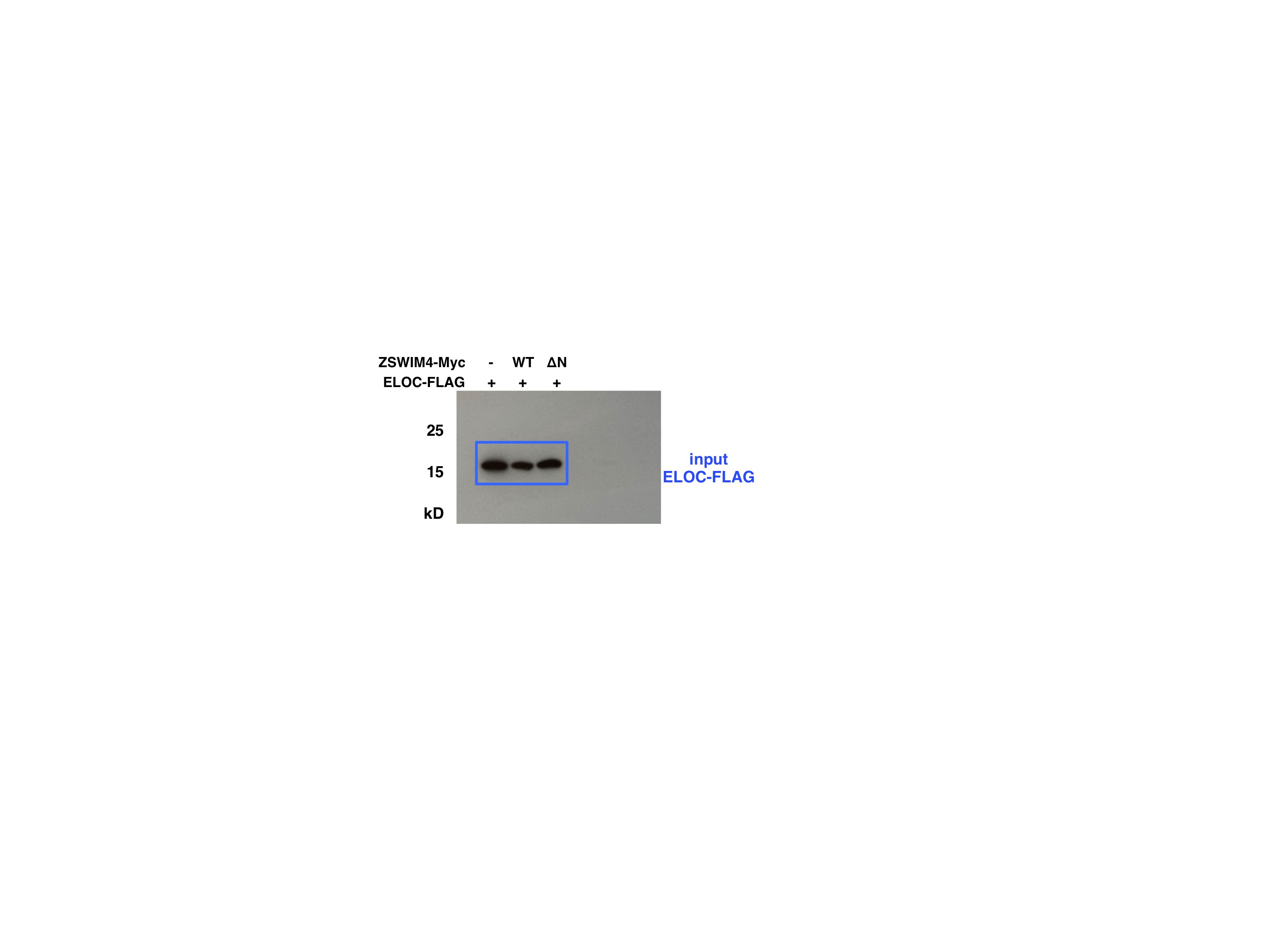

Supplement: Supplementary file 7 — Source Data Fig. 6 [file 44319_2023_46_MOESM7_ESM.zip › Figure 6/6I/western 6I ELOC-2.jpg]

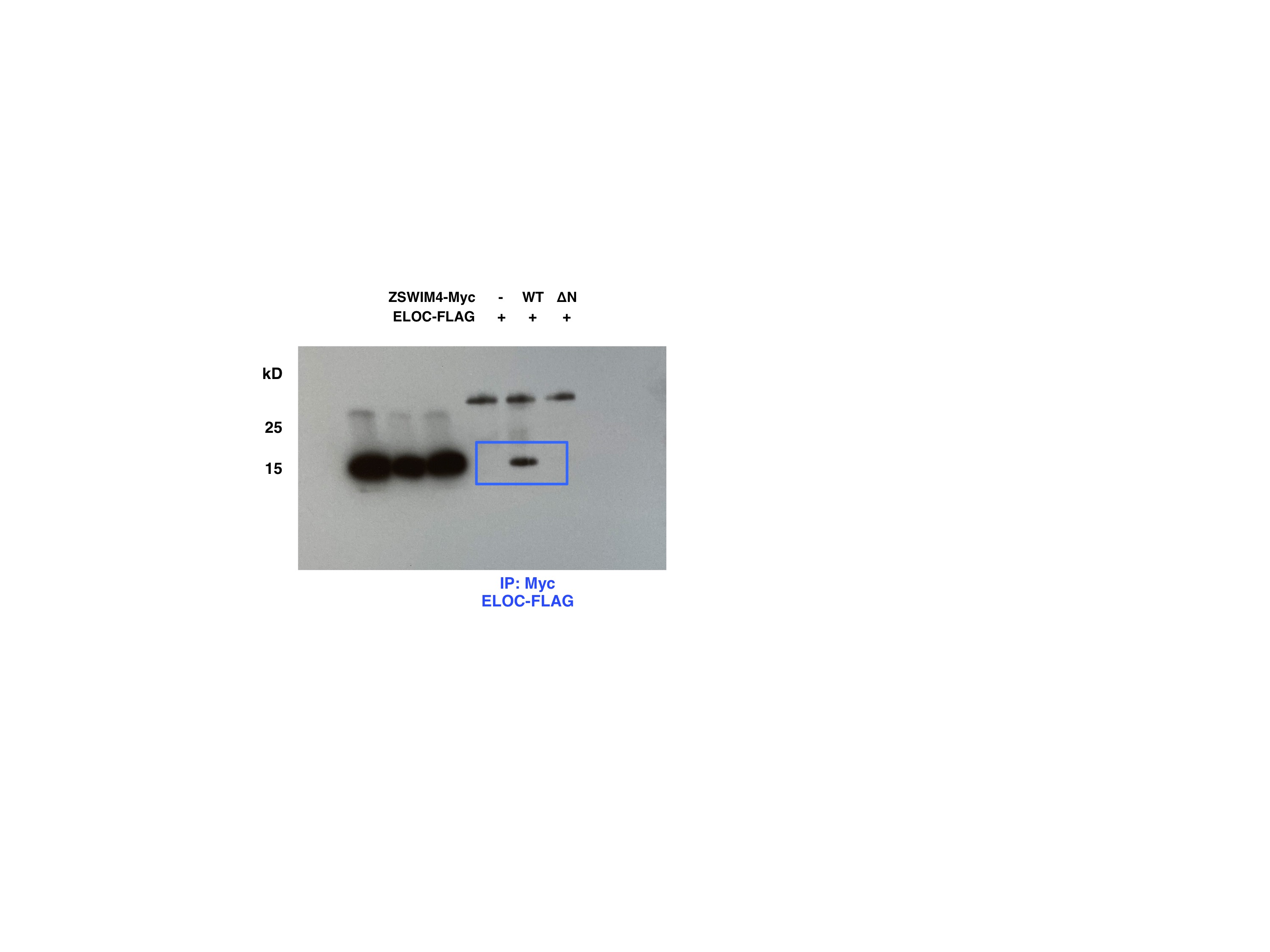

Supplement: Supplementary file 7 — Source Data Fig. 6 [file 44319_2023_46_MOESM7_ESM.zip › Figure 6/6I/western 6I ELOC.jpg]

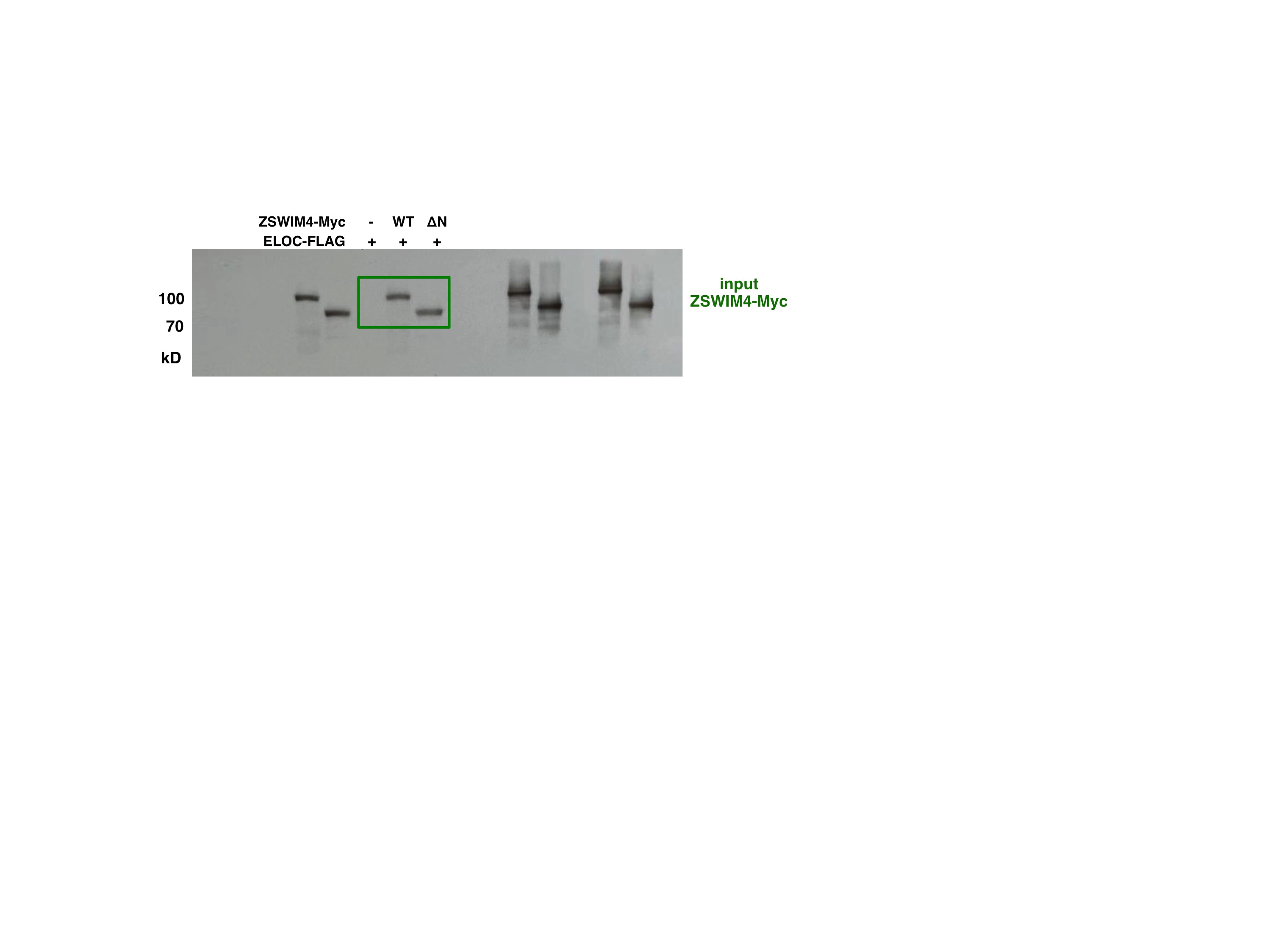

Supplement: Supplementary file 7 — Source Data Fig. 6 [file 44319_2023_46_MOESM7_ESM.zip › Figure 6/6I/western 6I zswim4.jpg]

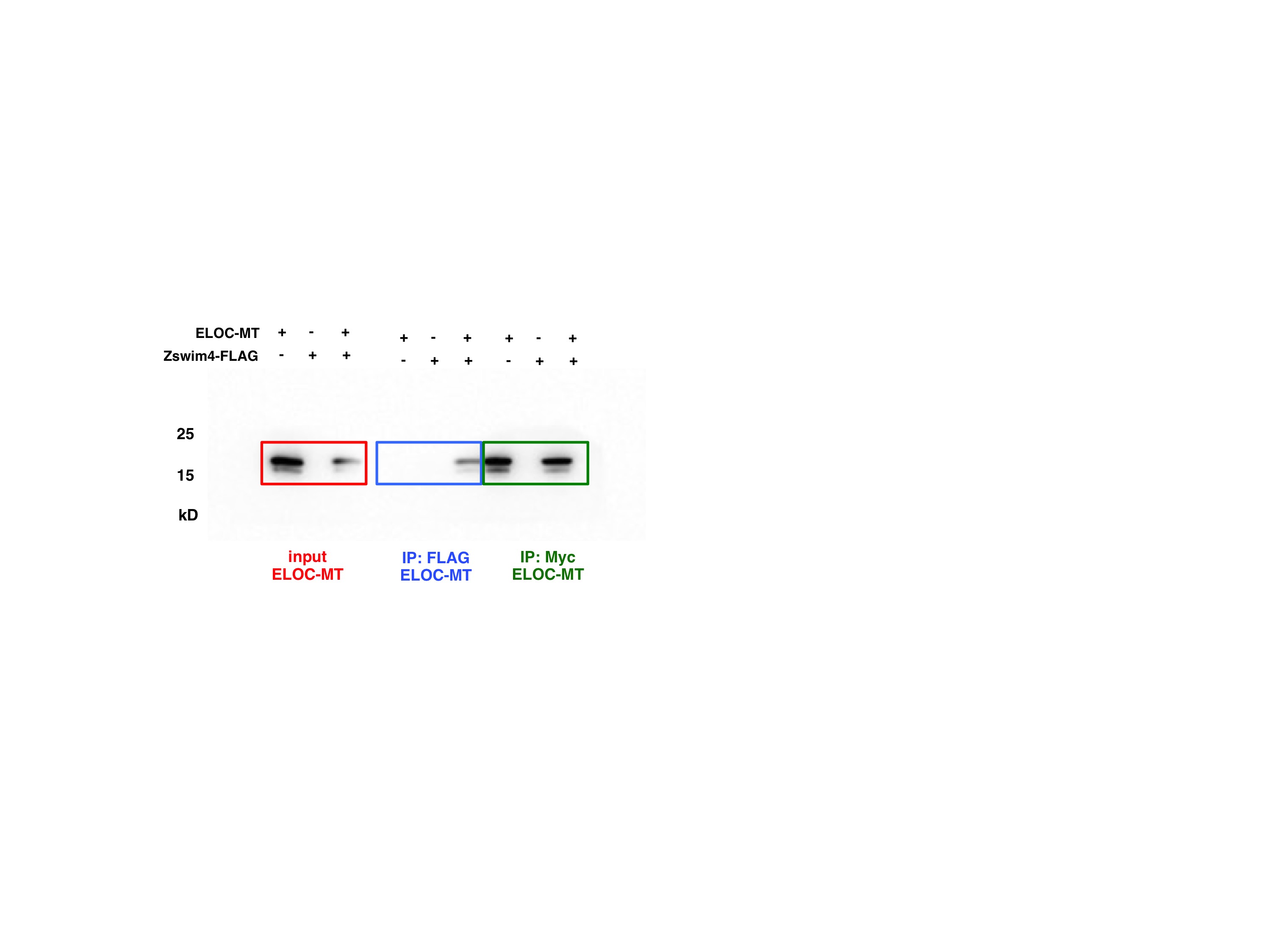

Supplement: Supplementary file 7 — Source Data Fig. 6 [file 44319_2023_46_MOESM7_ESM.zip › Figure 6/6D/western 6D ELOC.jpg]

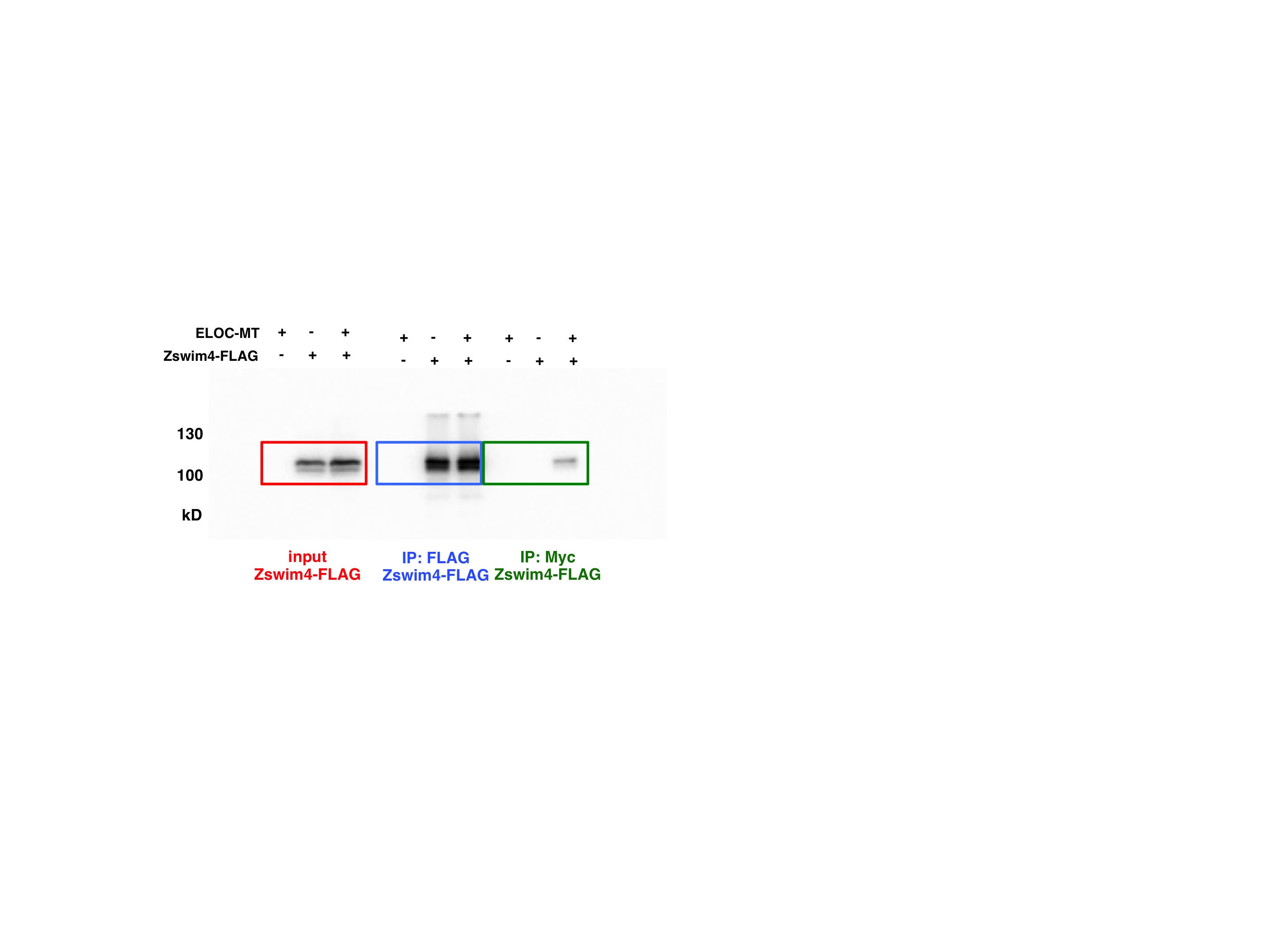

Supplement: Supplementary file 7 — Source Data Fig. 6 [file 44319_2023_46_MOESM7_ESM.zip › Figure 6/6D/western 6D Zswim4.jpg]

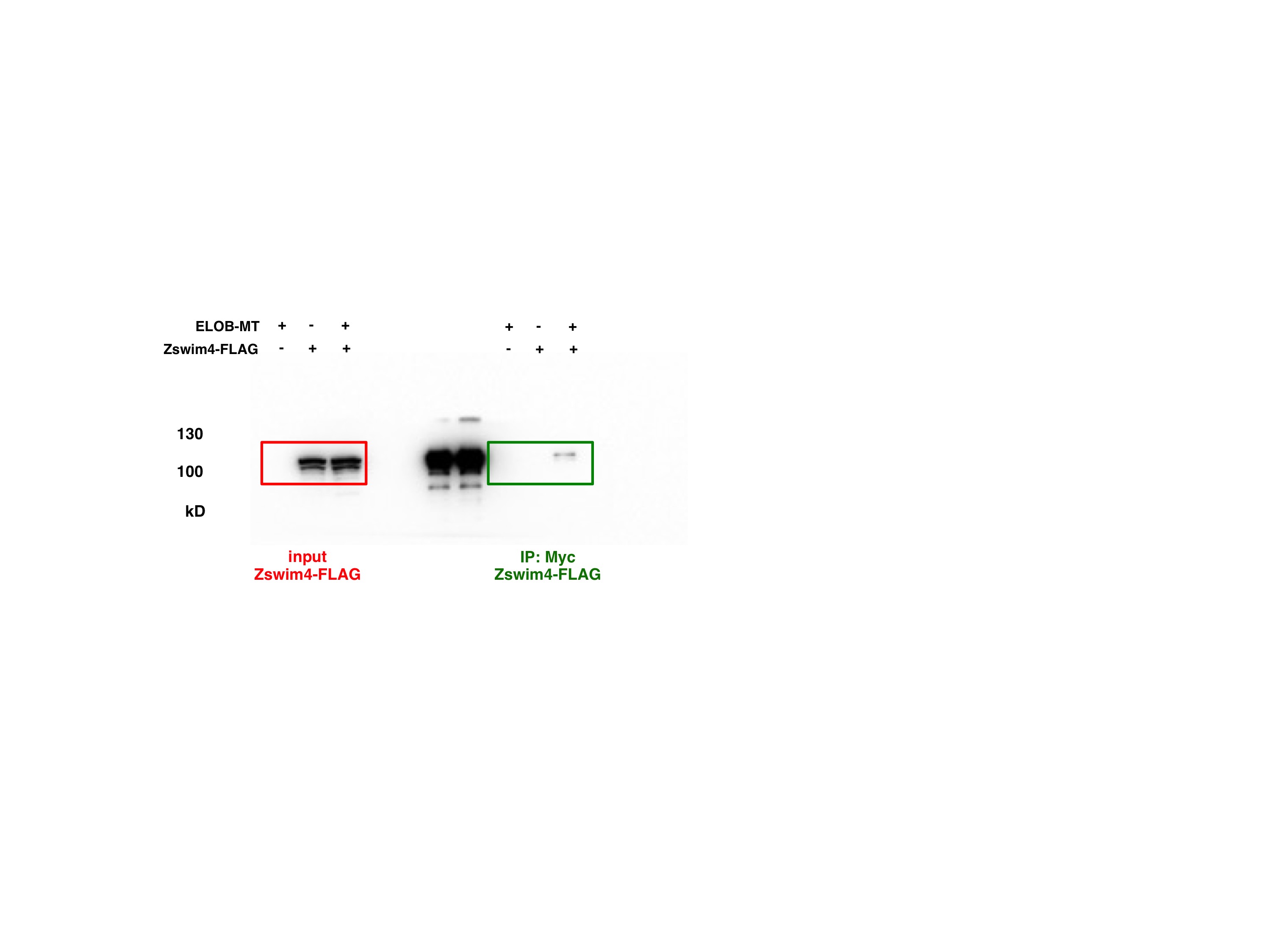

Supplement: Supplementary file 7 — Source Data Fig. 6 [file 44319_2023_46_MOESM7_ESM.zip › Figure 6/6C/western 6C Zswim4-2.jpg]

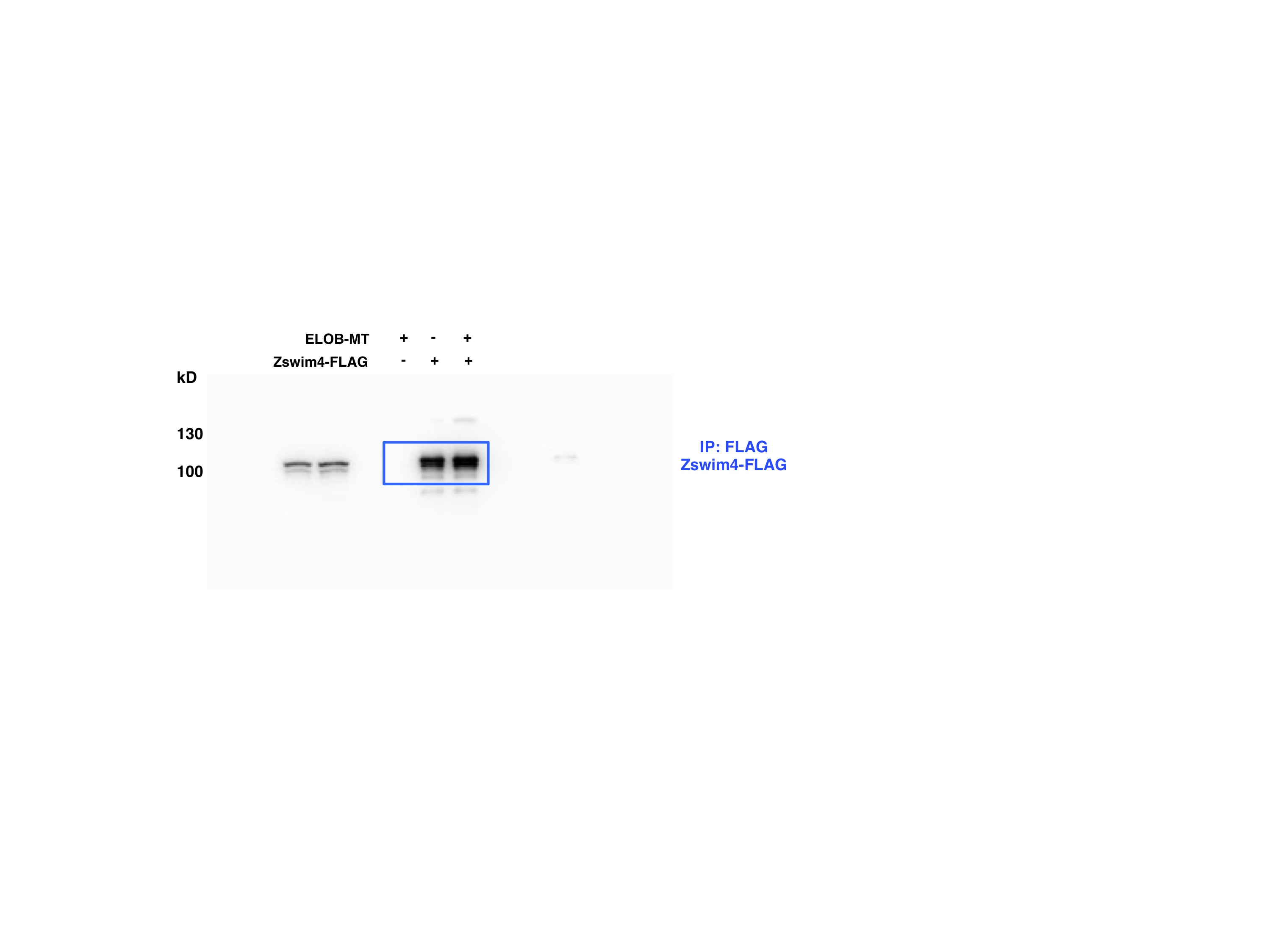

Supplement: Supplementary file 7 — Source Data Fig. 6 [file 44319_2023_46_MOESM7_ESM.zip › Figure 6/6C/western 6C Zswim4.jpg]

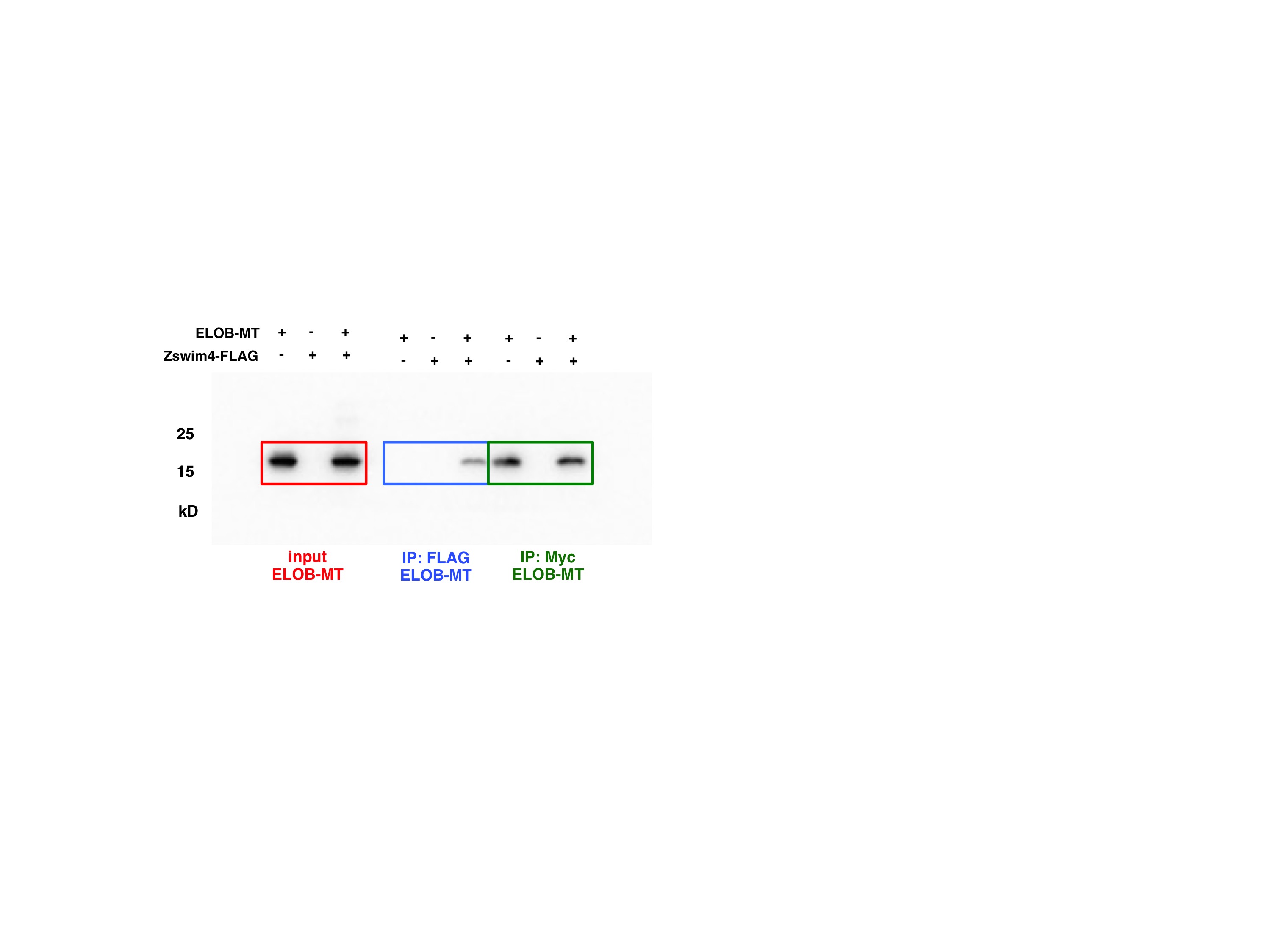

Supplement: Supplementary file 7 — Source Data Fig. 6 [file 44319_2023_46_MOESM7_ESM.zip › Figure 6/6C/western 6C ELOB.jpg]

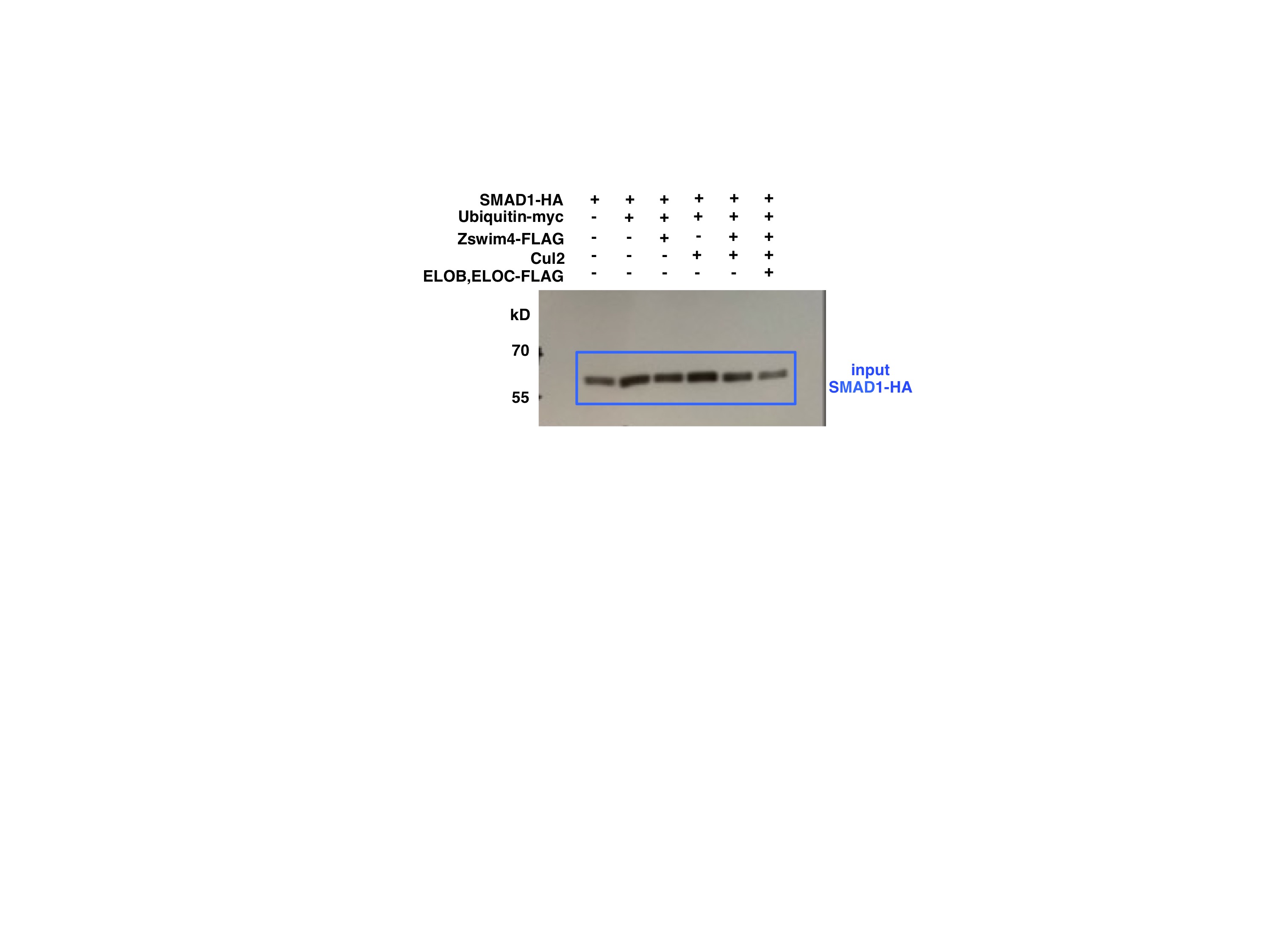

Supplement: Supplementary file 8 — Source Data Fig. 7 [file 44319_2023_46_MOESM8_ESM.zip › Figure 7/7B/western 7B smad1-2.jpg]

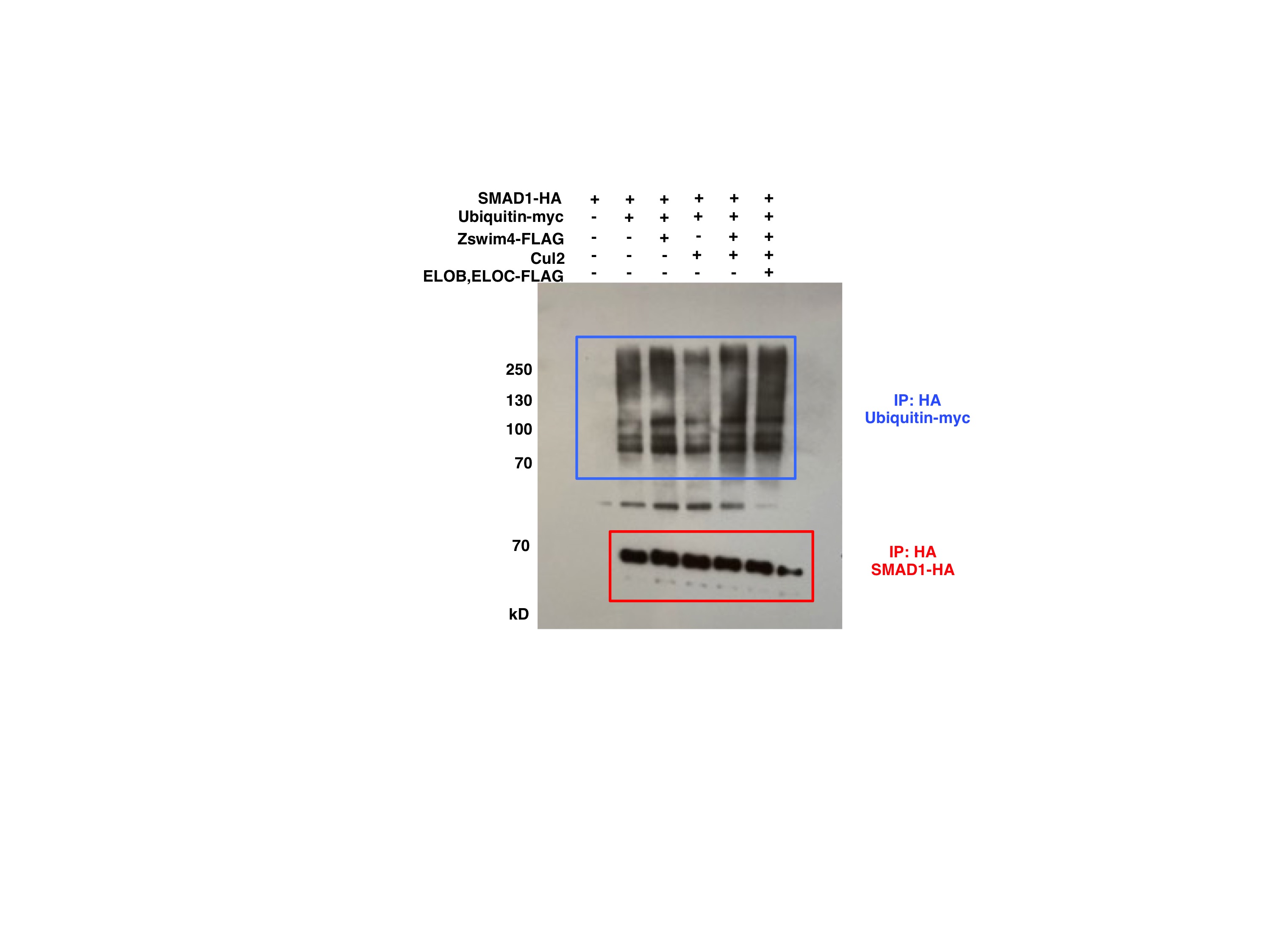

Supplement: Supplementary file 8 — Source Data Fig. 7 [file 44319_2023_46_MOESM8_ESM.zip › Figure 7/7B/western 7B ubiquitin smad1.jpg]

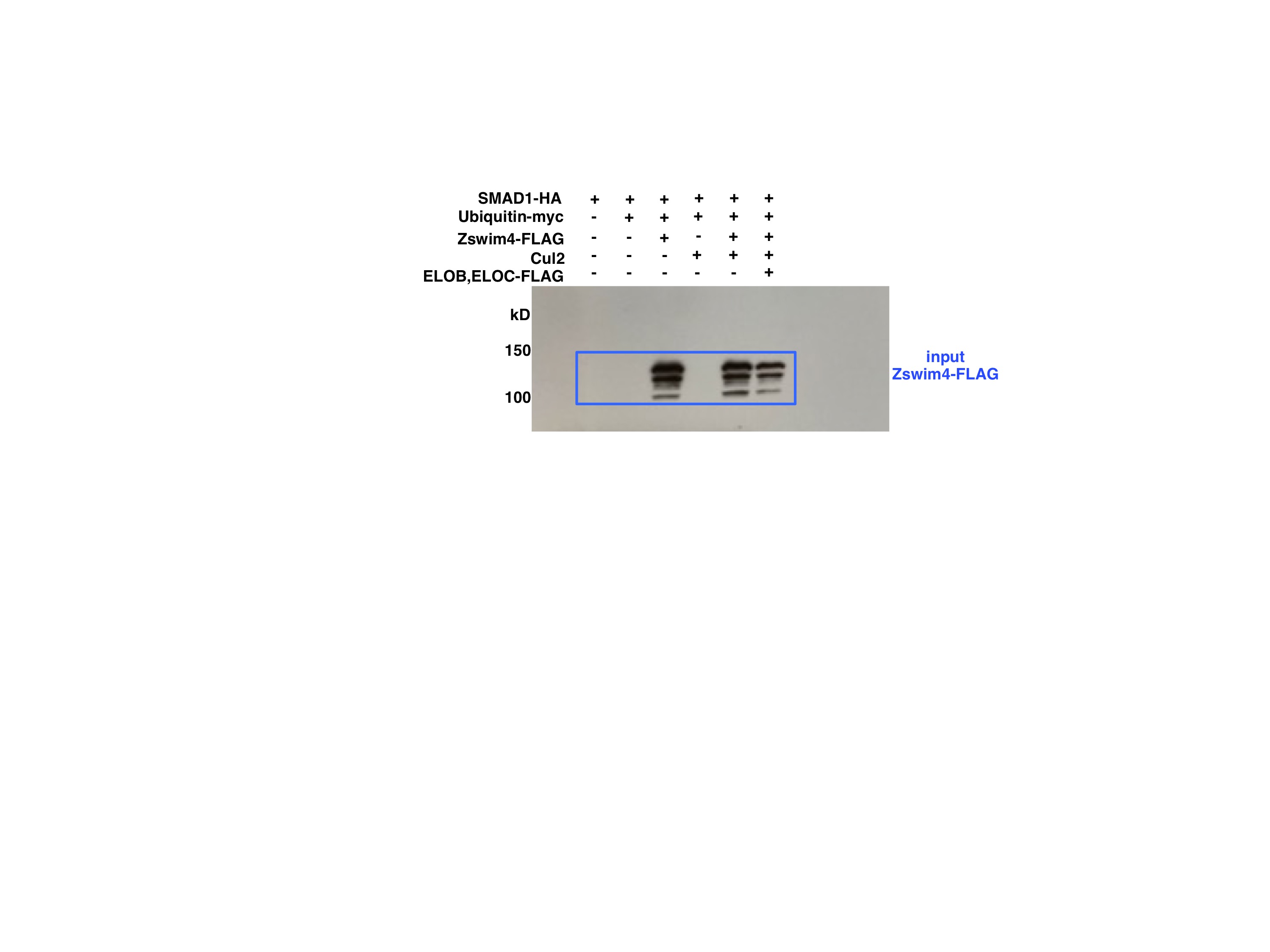

Supplement: Supplementary file 8 — Source Data Fig. 7 [file 44319_2023_46_MOESM8_ESM.zip › Figure 7/7B/western 7B zswim4.jpg]

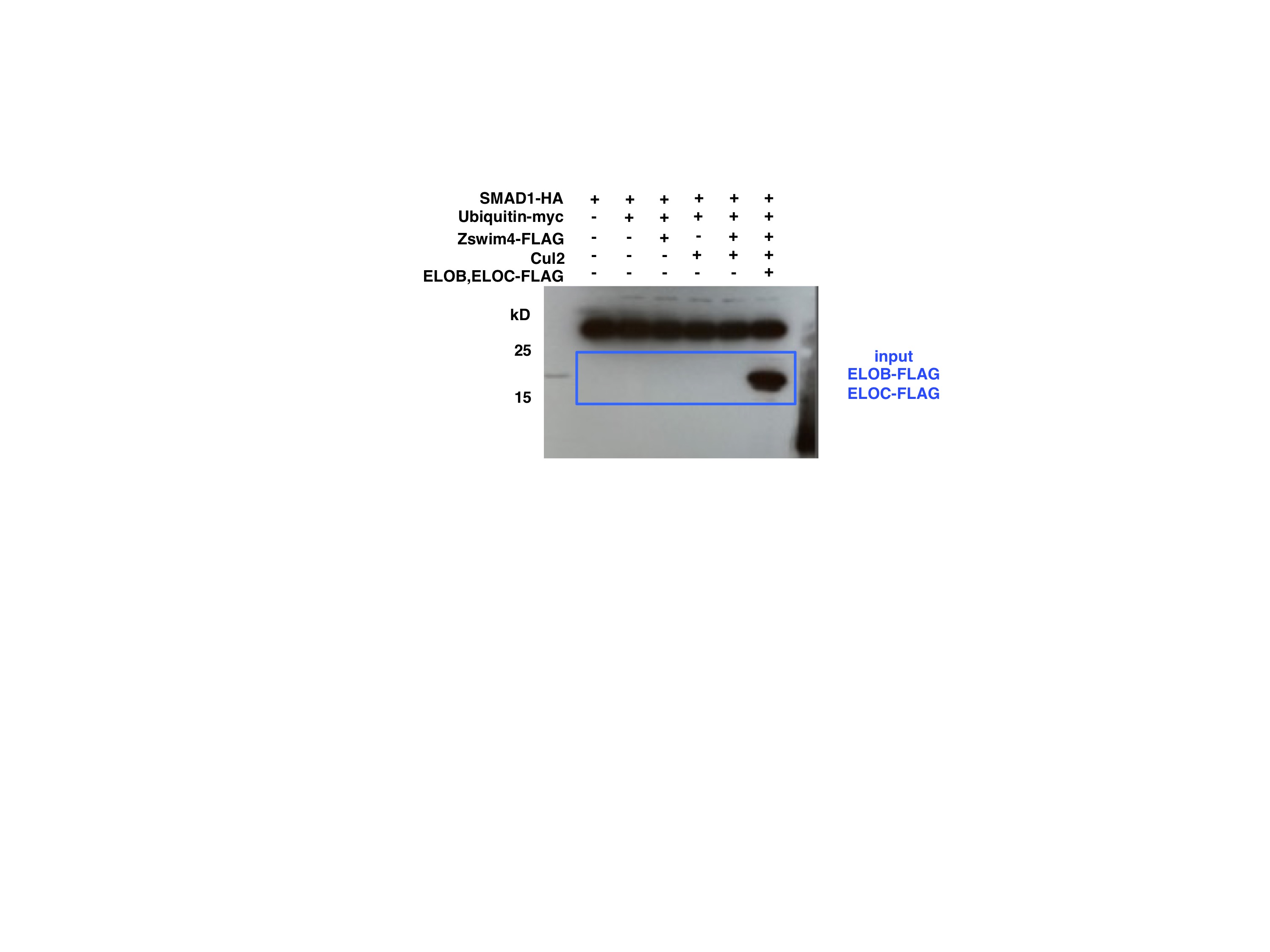

Supplement: Supplementary file 8 — Source Data Fig. 7 [file 44319_2023_46_MOESM8_ESM.zip › Figure 7/7B/western 7B elob eloc.jpg]

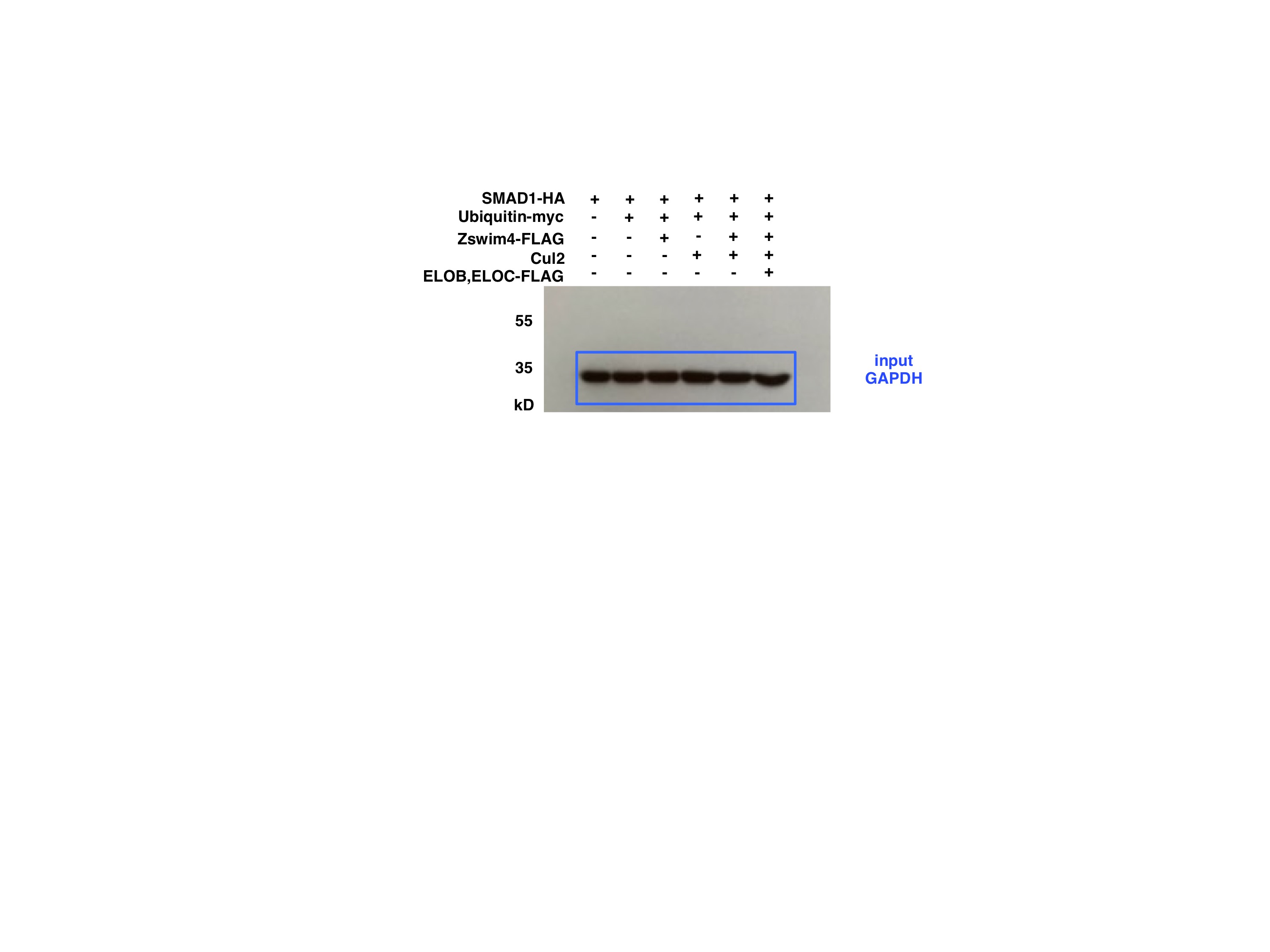

Supplement: Supplementary file 8 — Source Data Fig. 7 [file 44319_2023_46_MOESM8_ESM.zip › Figure 7/7B/western 7B GAPDH.jpg]

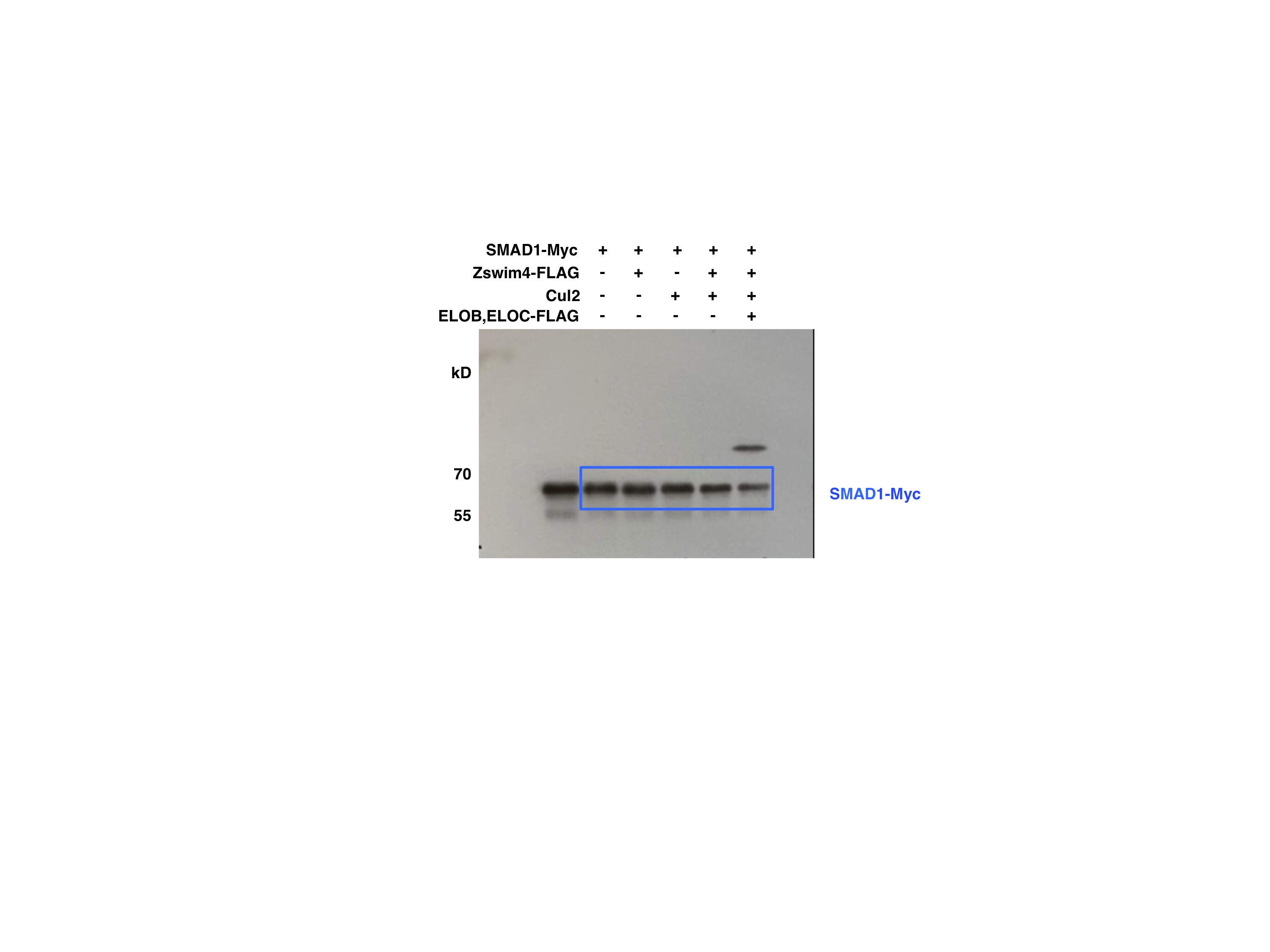

Supplement: Supplementary file 8 — Source Data Fig. 7 [file 44319_2023_46_MOESM8_ESM.zip › Figure 7/7A/western 7A smad1.jpg]

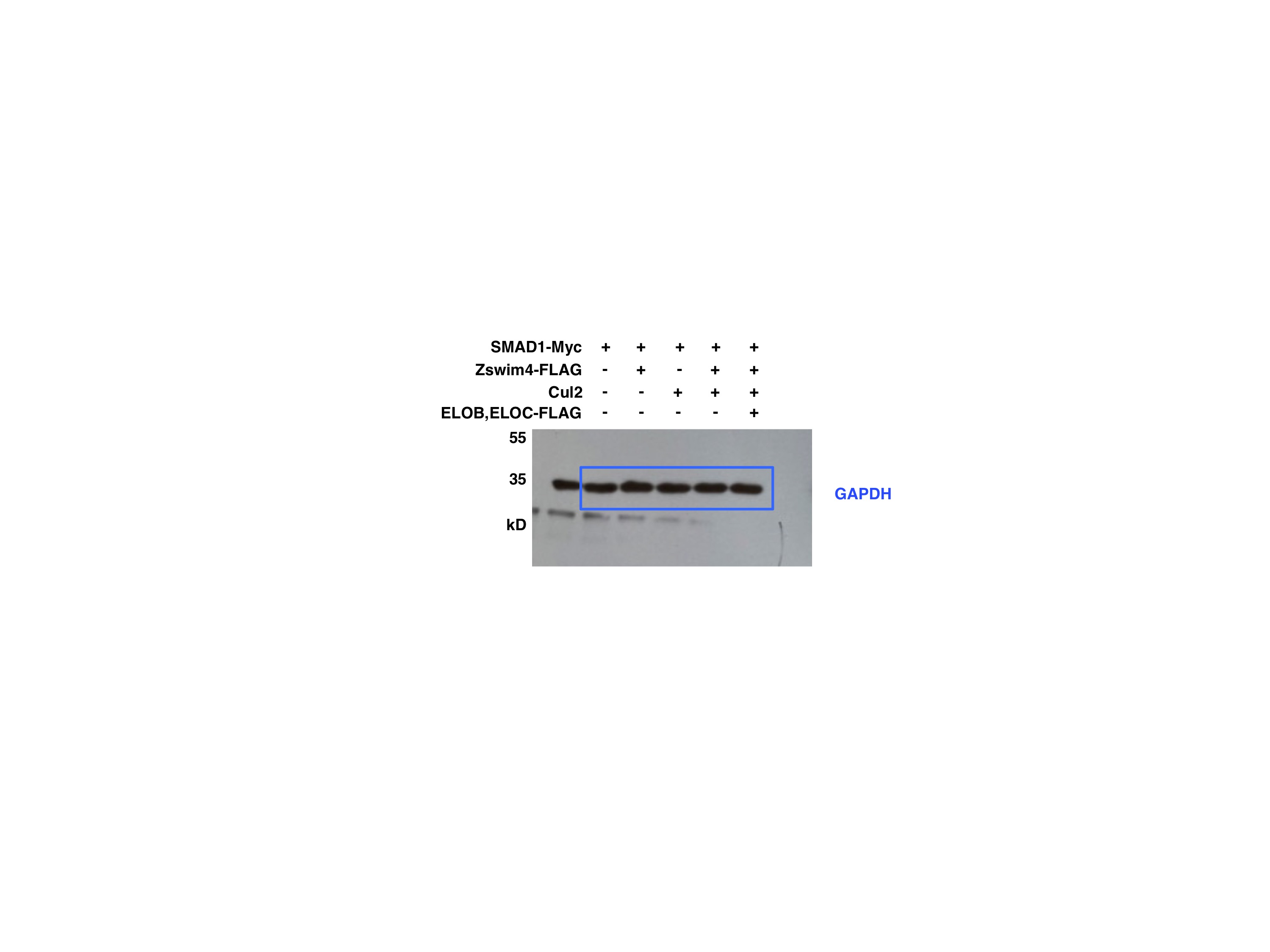

Supplement: Supplementary file 8 — Source Data Fig. 7 [file 44319_2023_46_MOESM8_ESM.zip › Figure 7/7A/western 7A GAPDH.jpg]

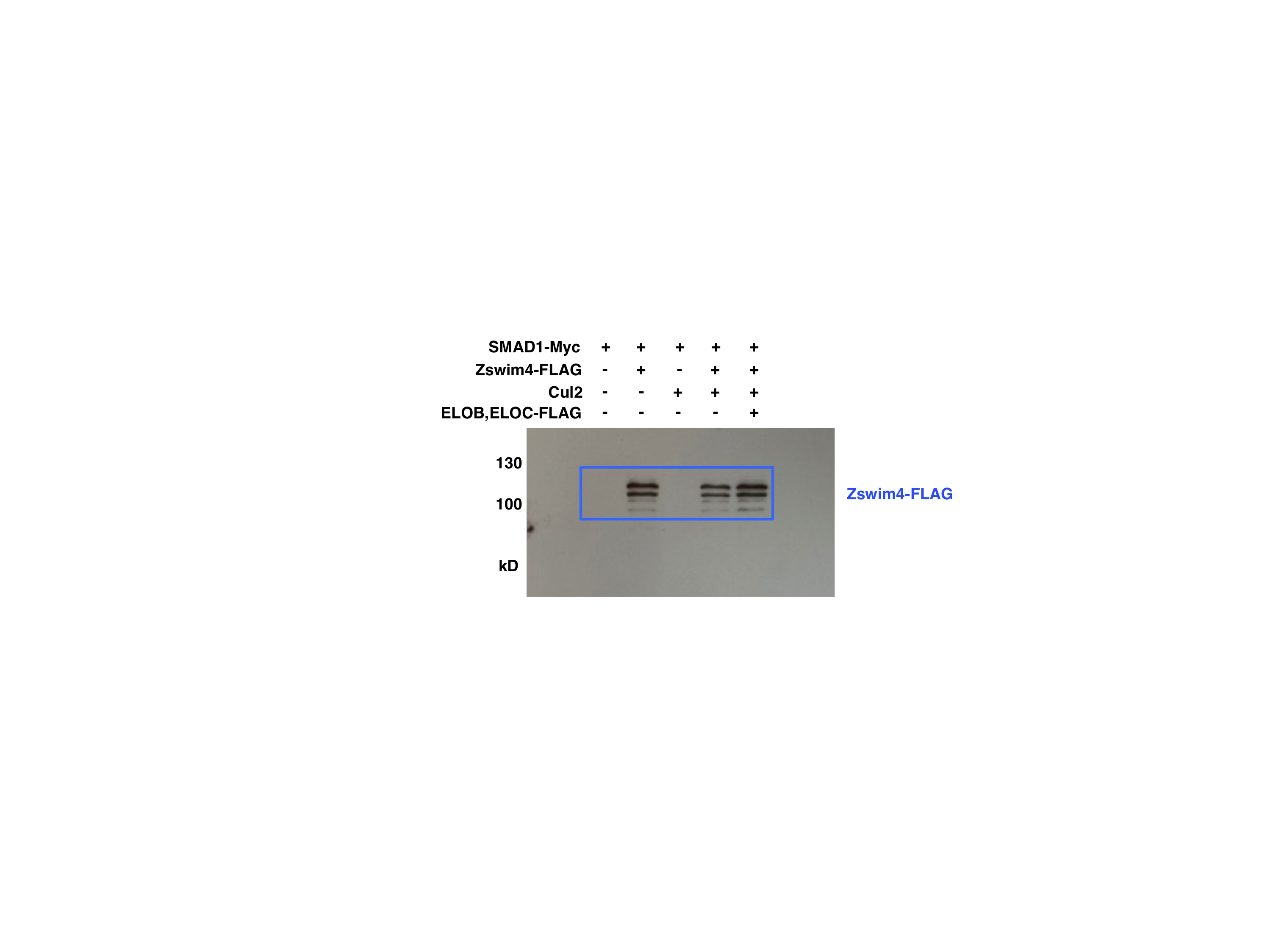

Supplement: Supplementary file 8 — Source Data Fig. 7 [file 44319_2023_46_MOESM8_ESM.zip › Figure 7/7A/western 7A zswim4.jpg]

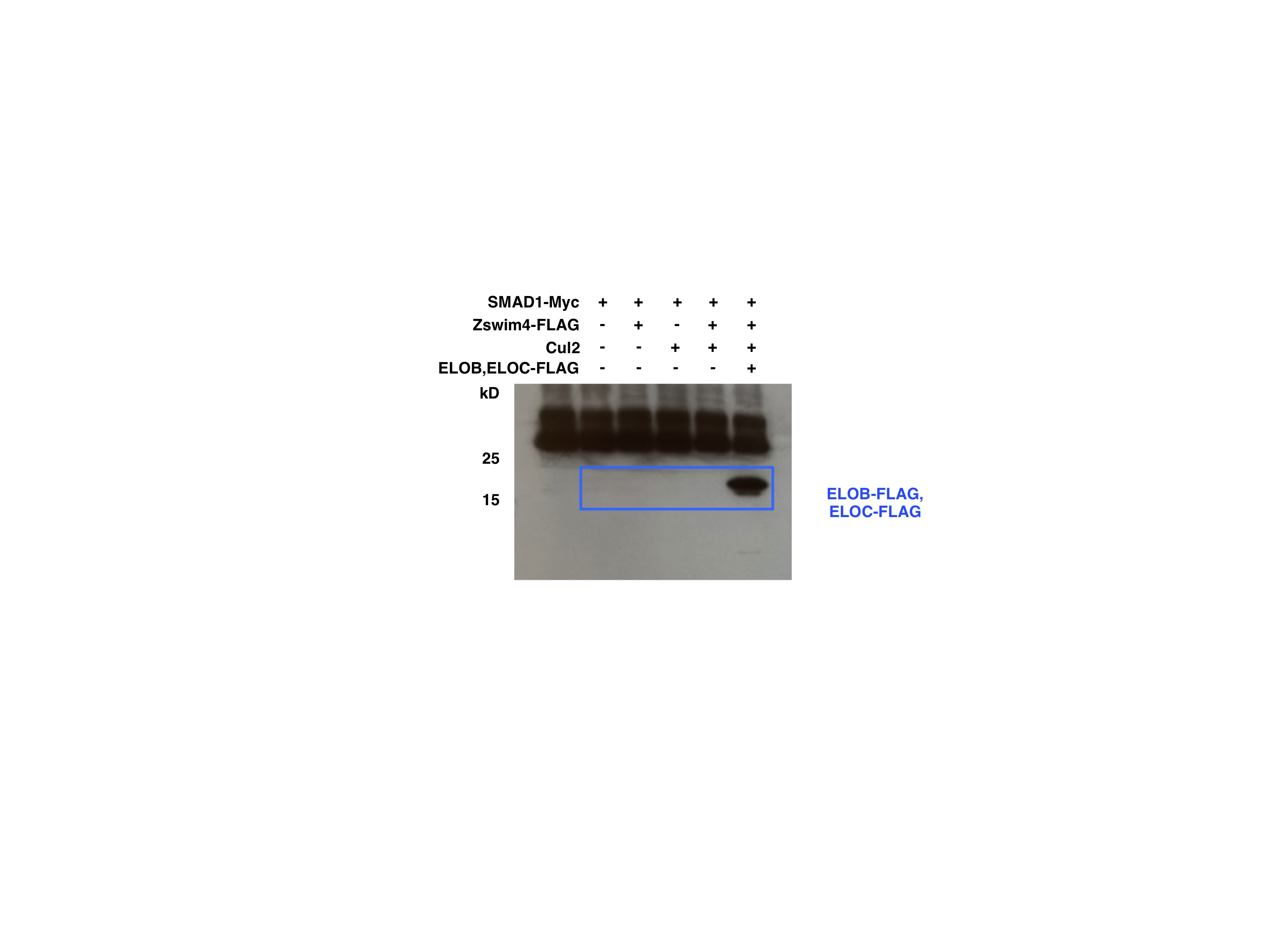

Supplement: Supplementary file 8 — Source Data Fig. 7 [file 44319_2023_46_MOESM8_ESM.zip › Figure 7/7A/western 7A elob eloc.jpg]

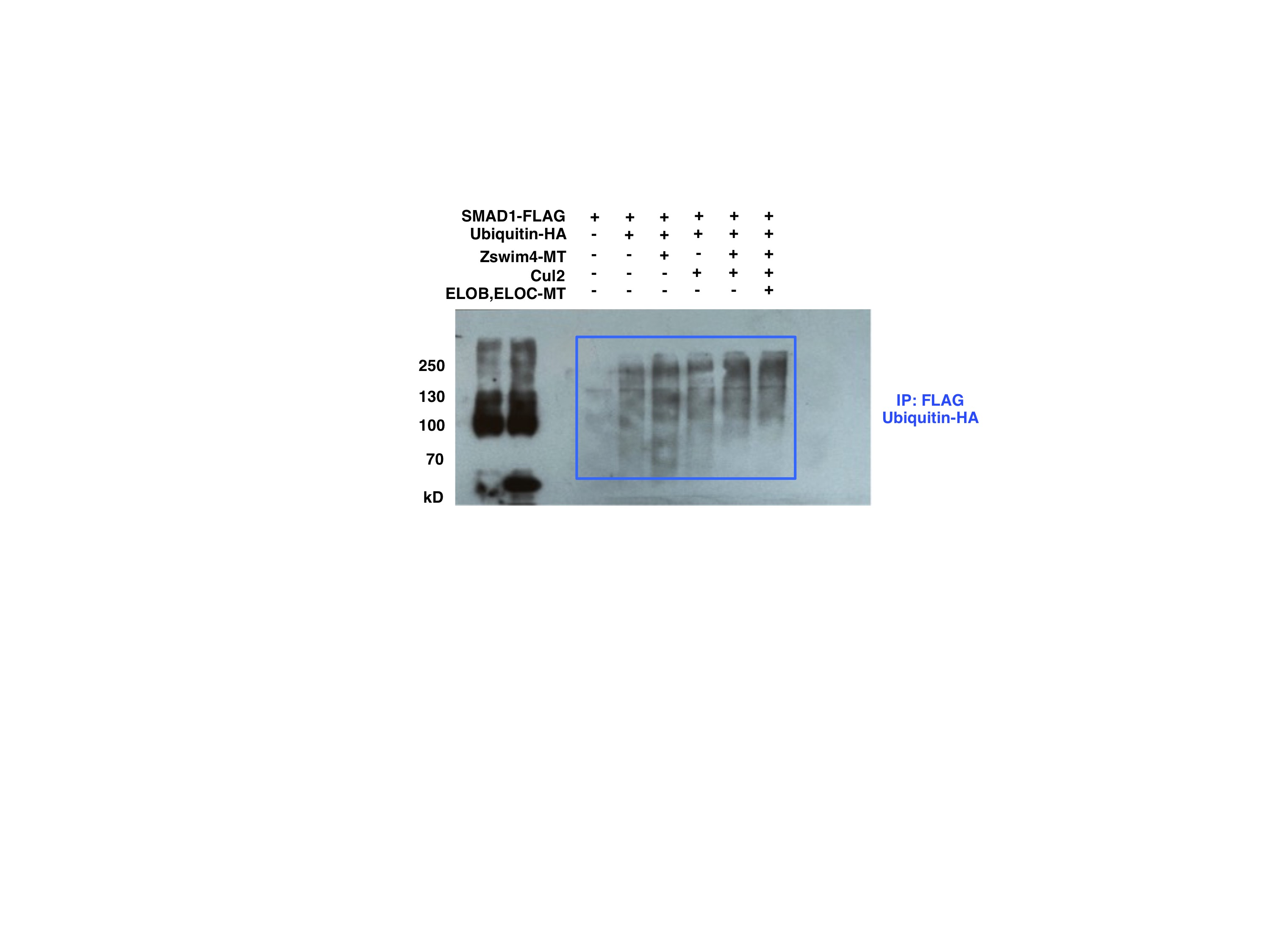

Supplement: Supplementary file 8 — Source Data Fig. 7 [file 44319_2023_46_MOESM8_ESM.zip › Figure 7/7B/replicate/western 7B ubiquitin replicate.jpg]

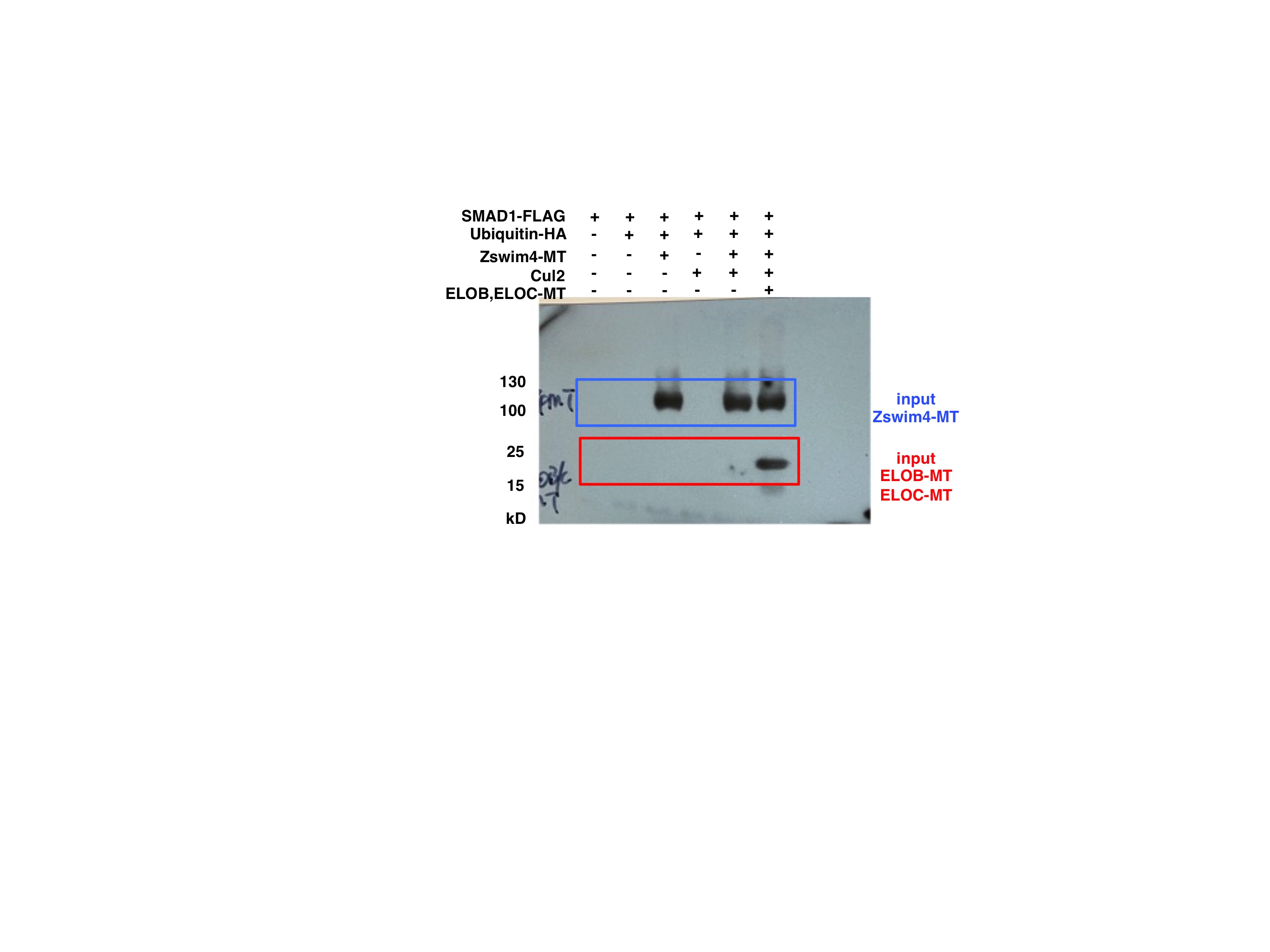

Supplement: Supplementary file 8 — Source Data Fig. 7 [file 44319_2023_46_MOESM8_ESM.zip › Figure 7/7B/replicate/western 7B elob eloc zswim4 replicate.jpg]

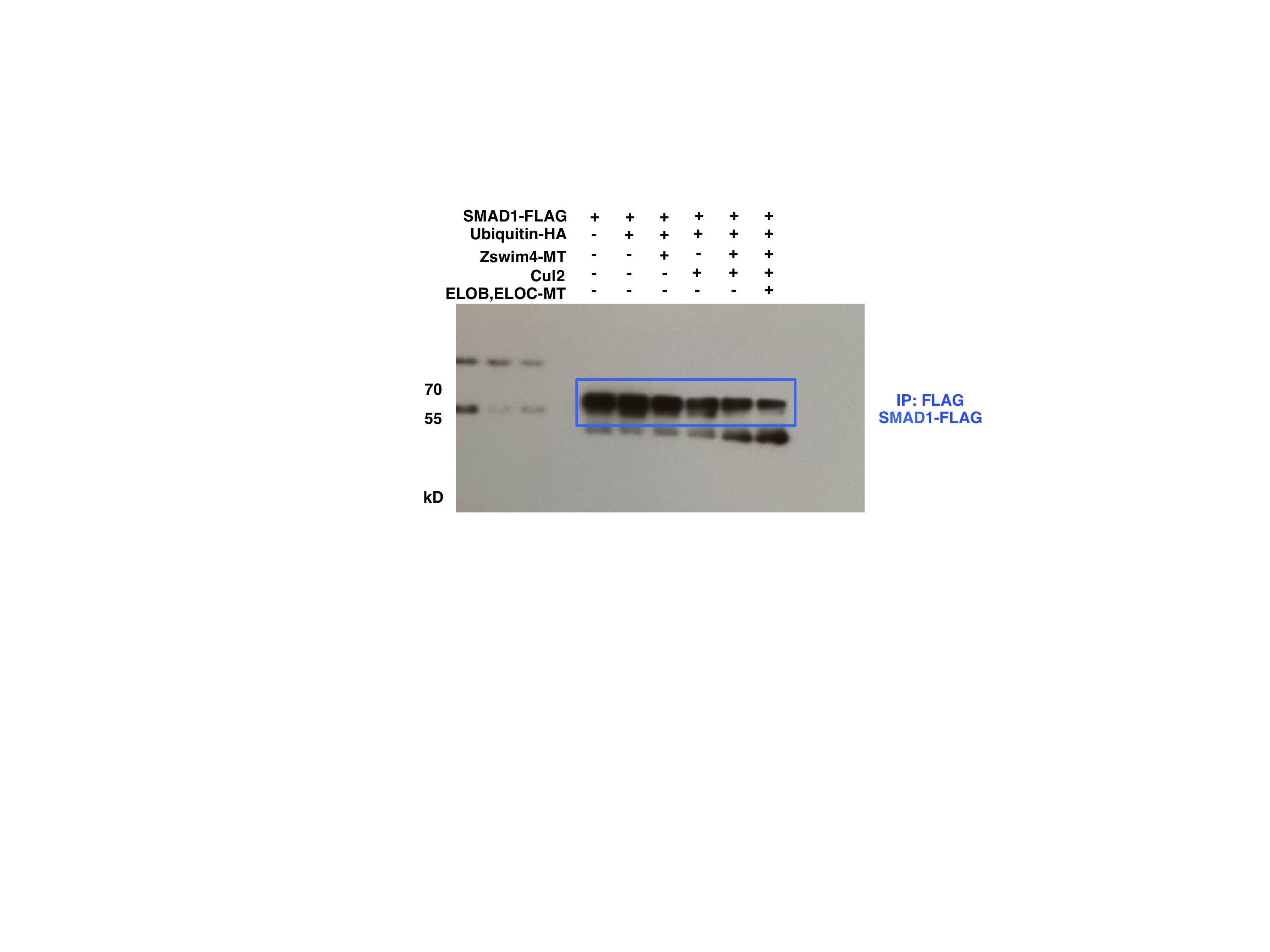

Supplement: Supplementary file 8 — Source Data Fig. 7 [file 44319_2023_46_MOESM8_ESM.zip › Figure 7/7B/replicate/western 7B smad1 replicate.jpg]

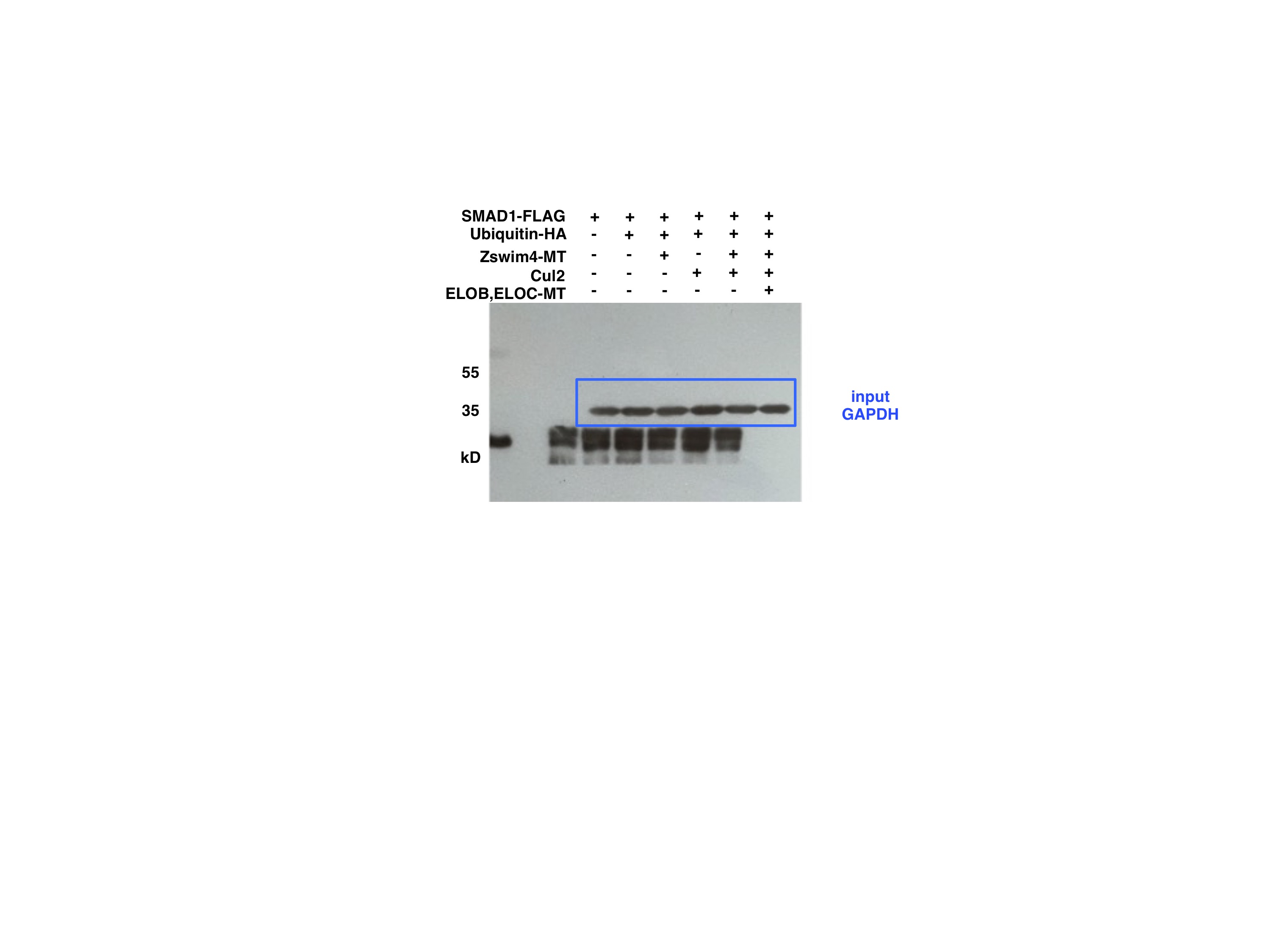

Supplement: Supplementary file 8 — Source Data Fig. 7 [file 44319_2023_46_MOESM8_ESM.zip › Figure 7/7B/replicate/western 7B GAPDH replicate.jpg]

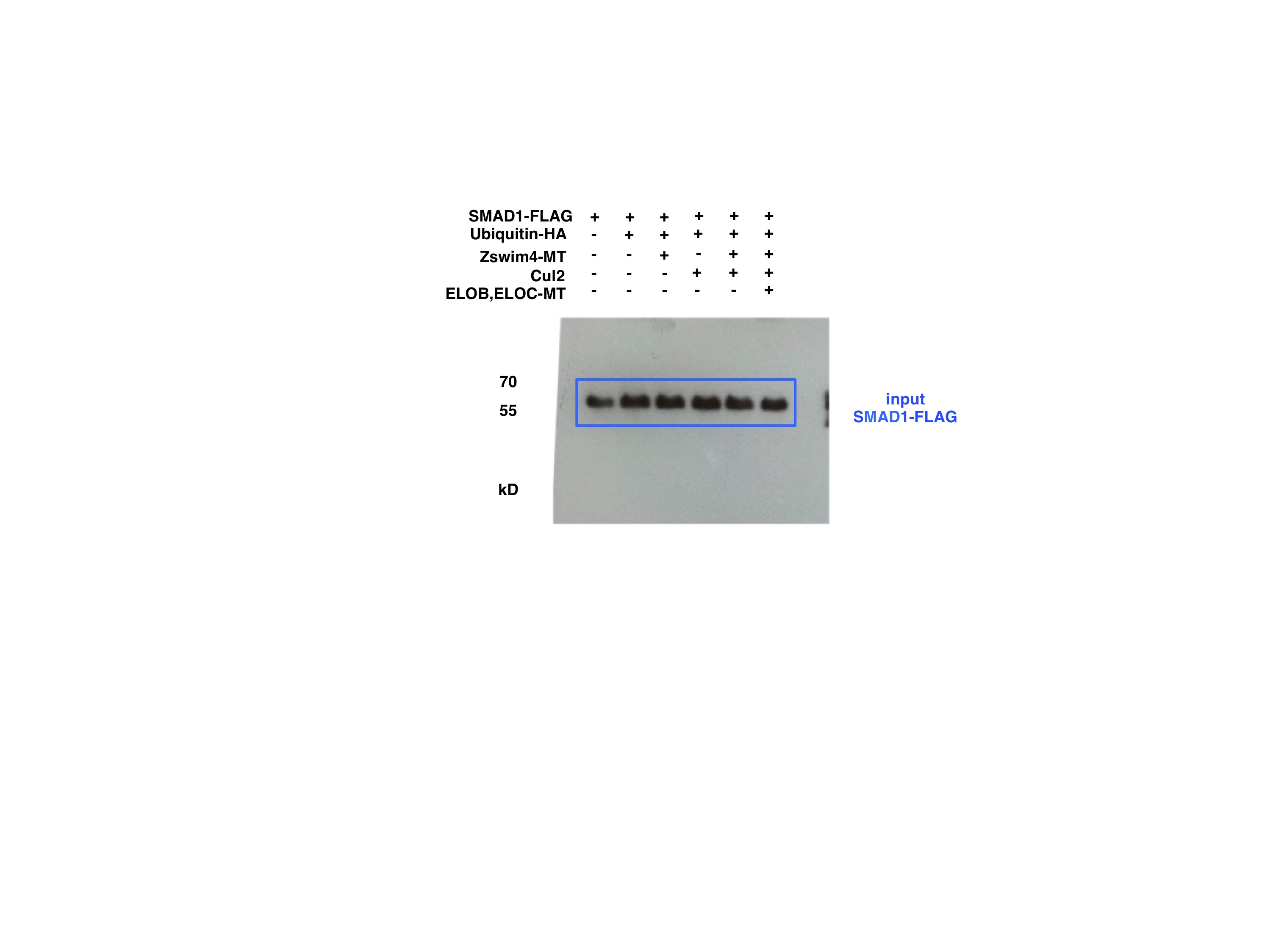

Supplement: Supplementary file 8 — Source Data Fig. 7 [file 44319_2023_46_MOESM8_ESM.zip › Figure 7/7B/replicate/western 7B smad1-2 replicate.jpg]

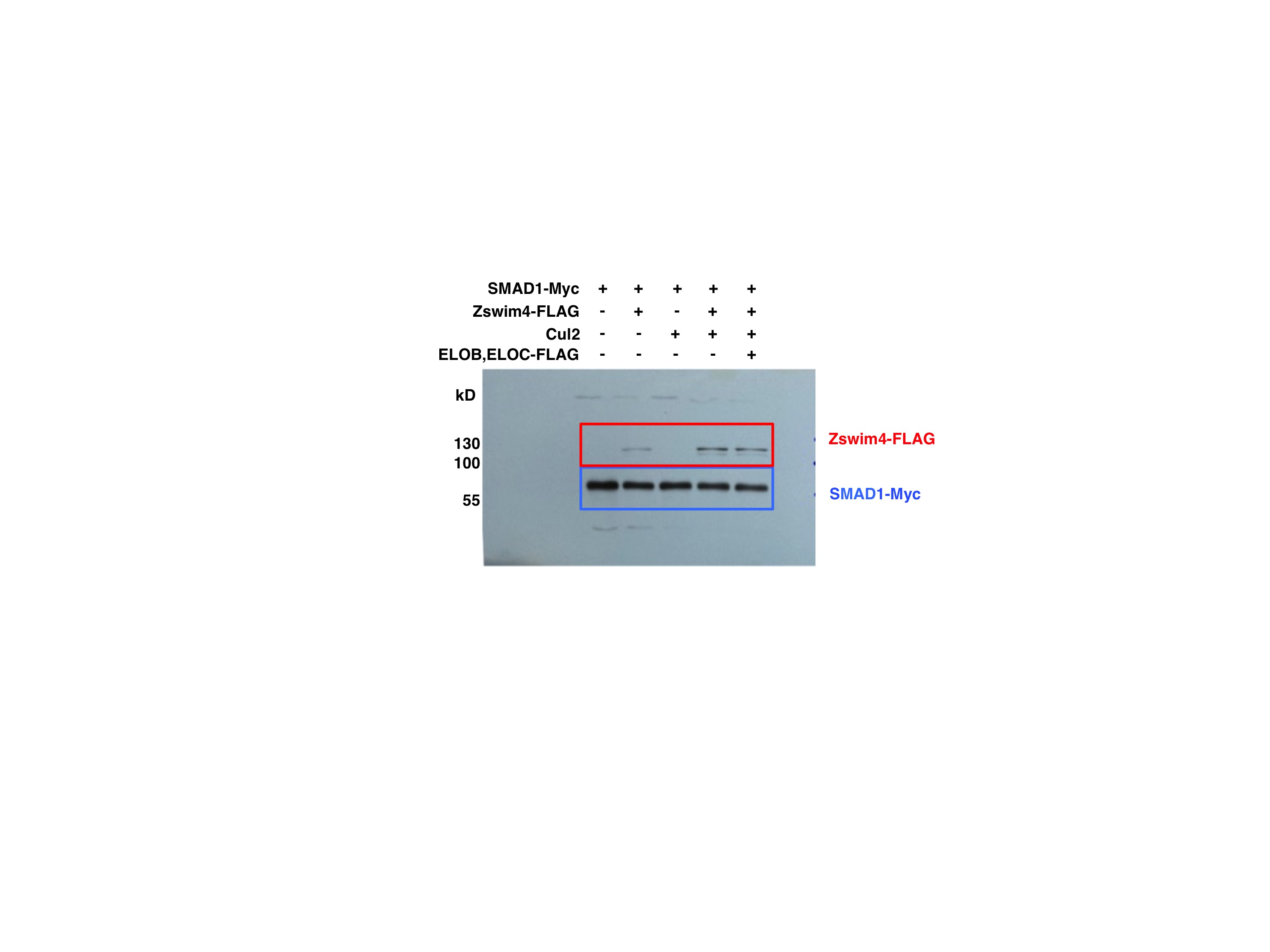

Supplement: Supplementary file 8 — Source Data Fig. 7 [file 44319_2023_46_MOESM8_ESM.zip › Figure 7/7A/replicate/western 7A zswim4 smad1 replicate.jpg]

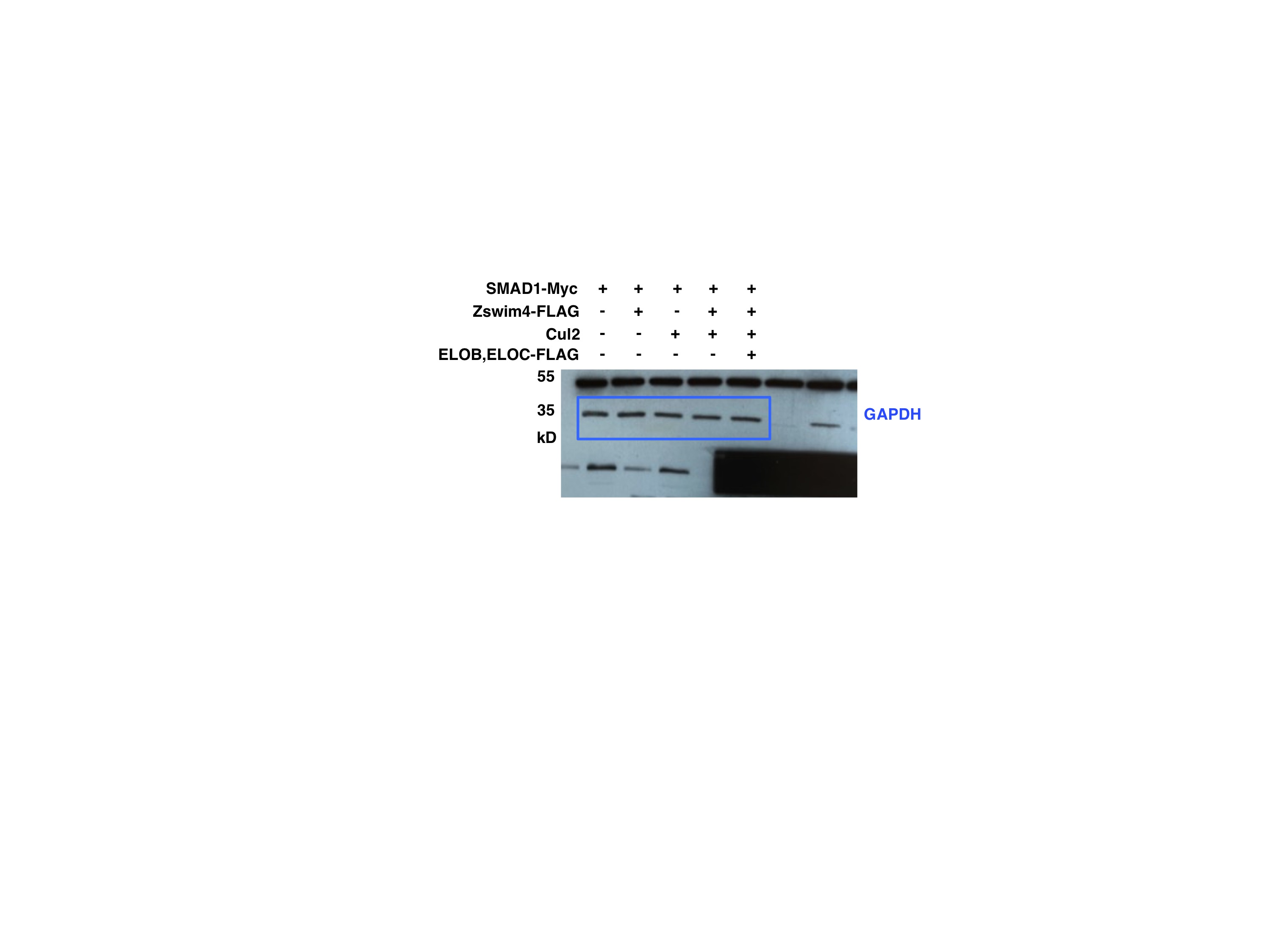

Supplement: Supplementary file 8 — Source Data Fig. 7 [file 44319_2023_46_MOESM8_ESM.zip › Figure 7/7A/replicate/western 7A GAPDH replicate.jpg]

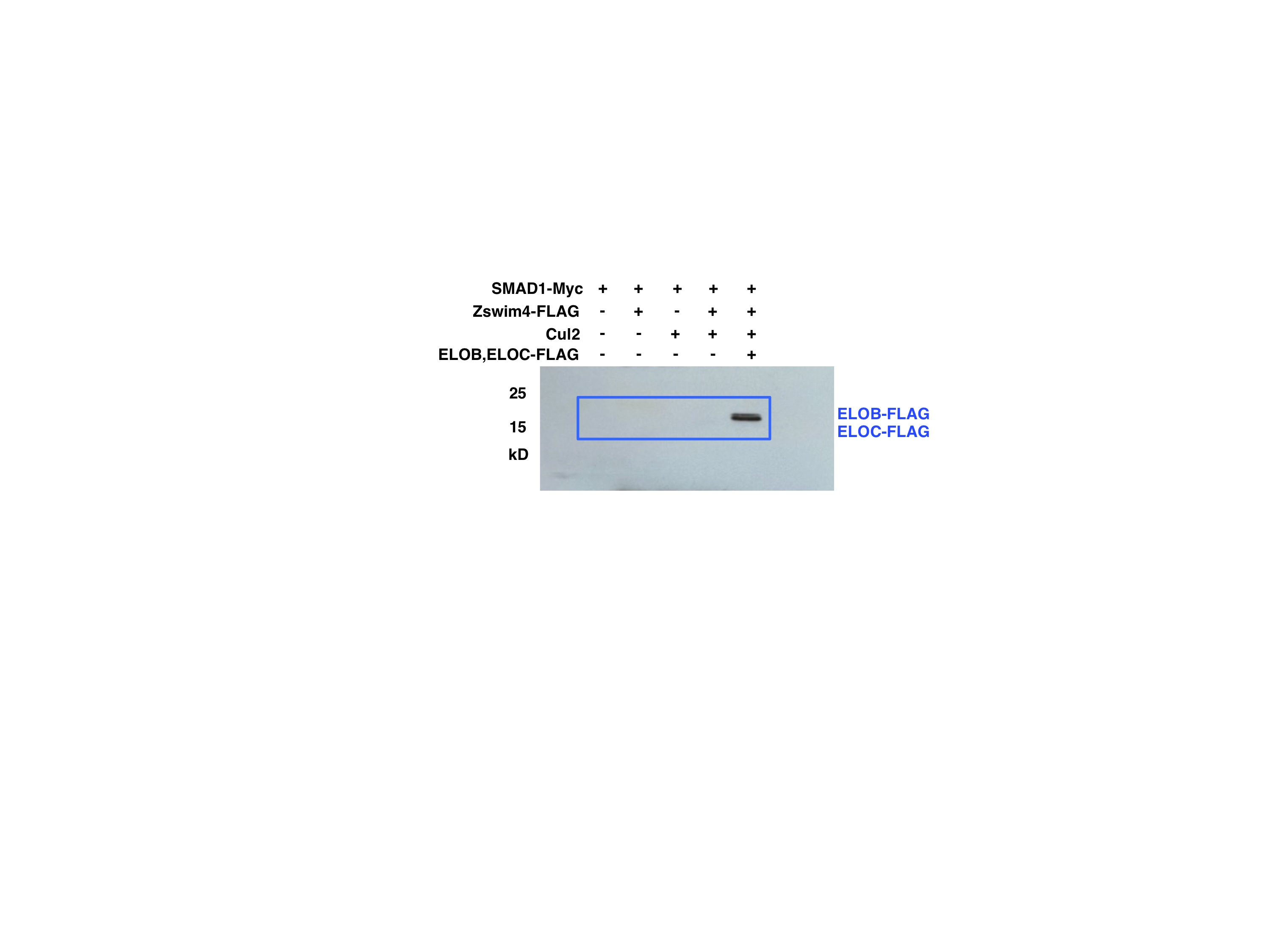

Supplement: Supplementary file 8 — Source Data Fig. 7 [file 44319_2023_46_MOESM8_ESM.zip › Figure 7/7A/replicate/western 7A elob eloc replicate.jpg]

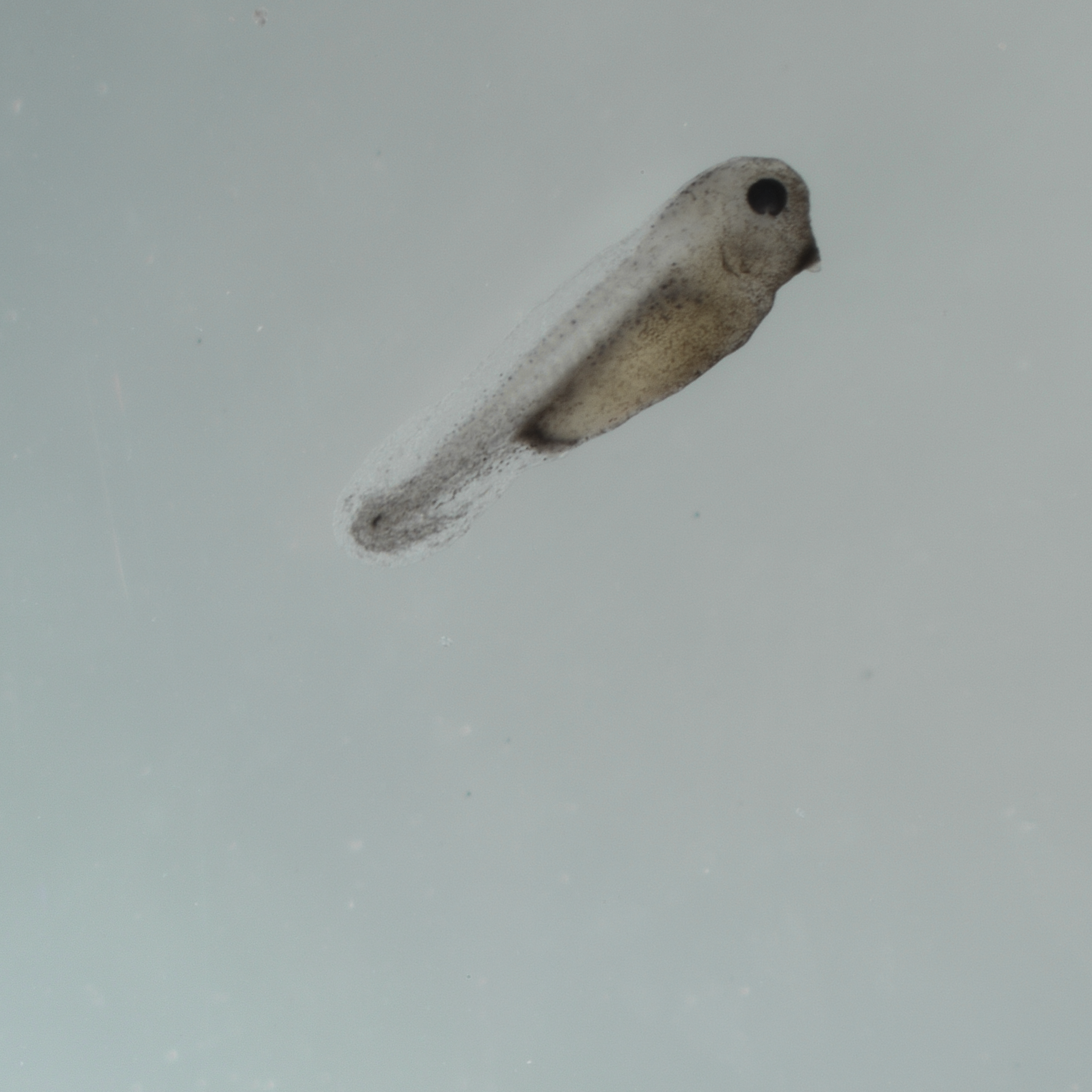

Supplement: Supplementary file 9 — Source Data EV Figures [file 44319_2023_46_MOESM9_ESM.zip › EV Figures/Figure EV4/EV4F/image EV4D normal.tif]

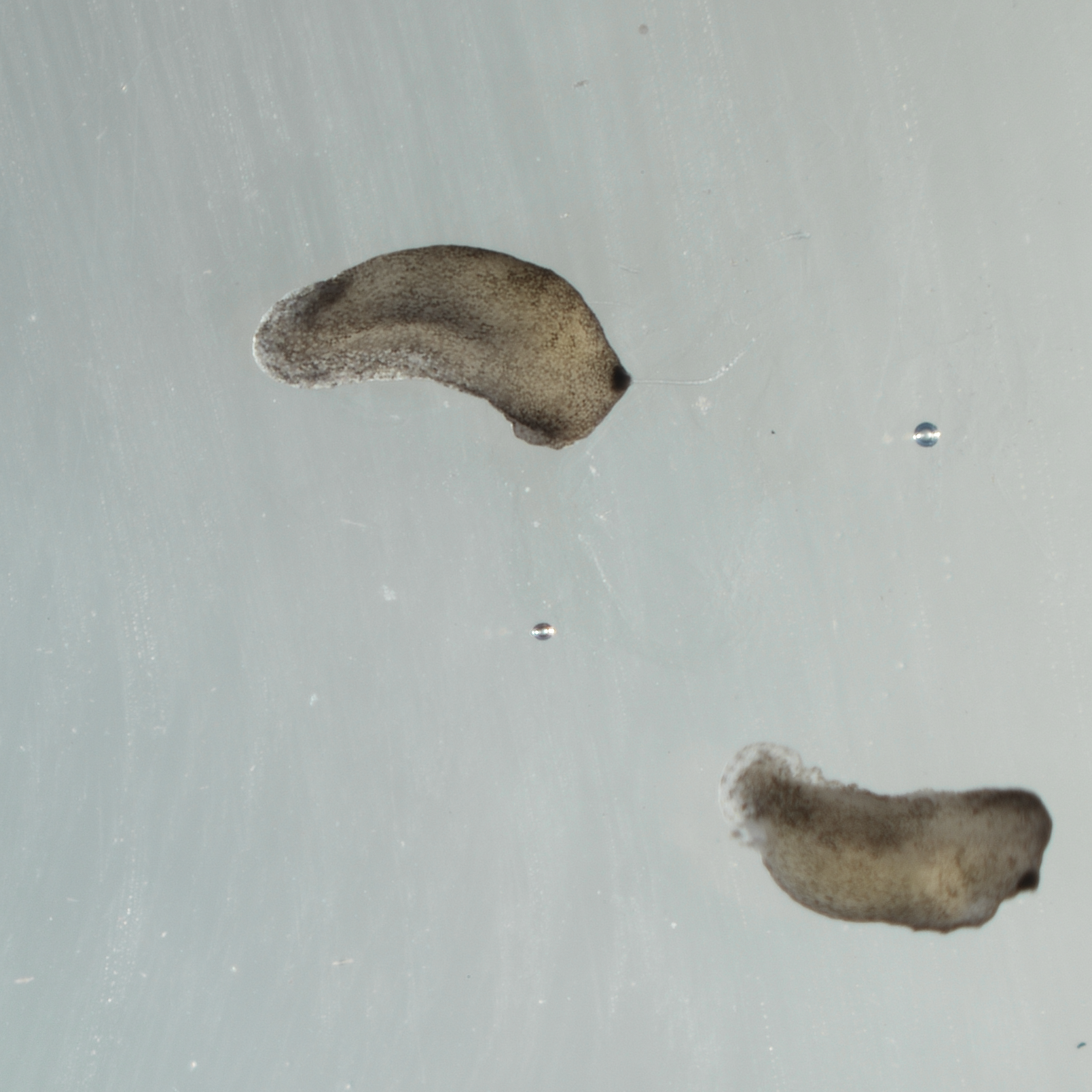

Supplement: Supplementary file 9 — Source Data EV Figures [file 44319_2023_46_MOESM9_ESM.zip › EV Figures/Figure EV4/EV4F/image EV4D moderate.tif]

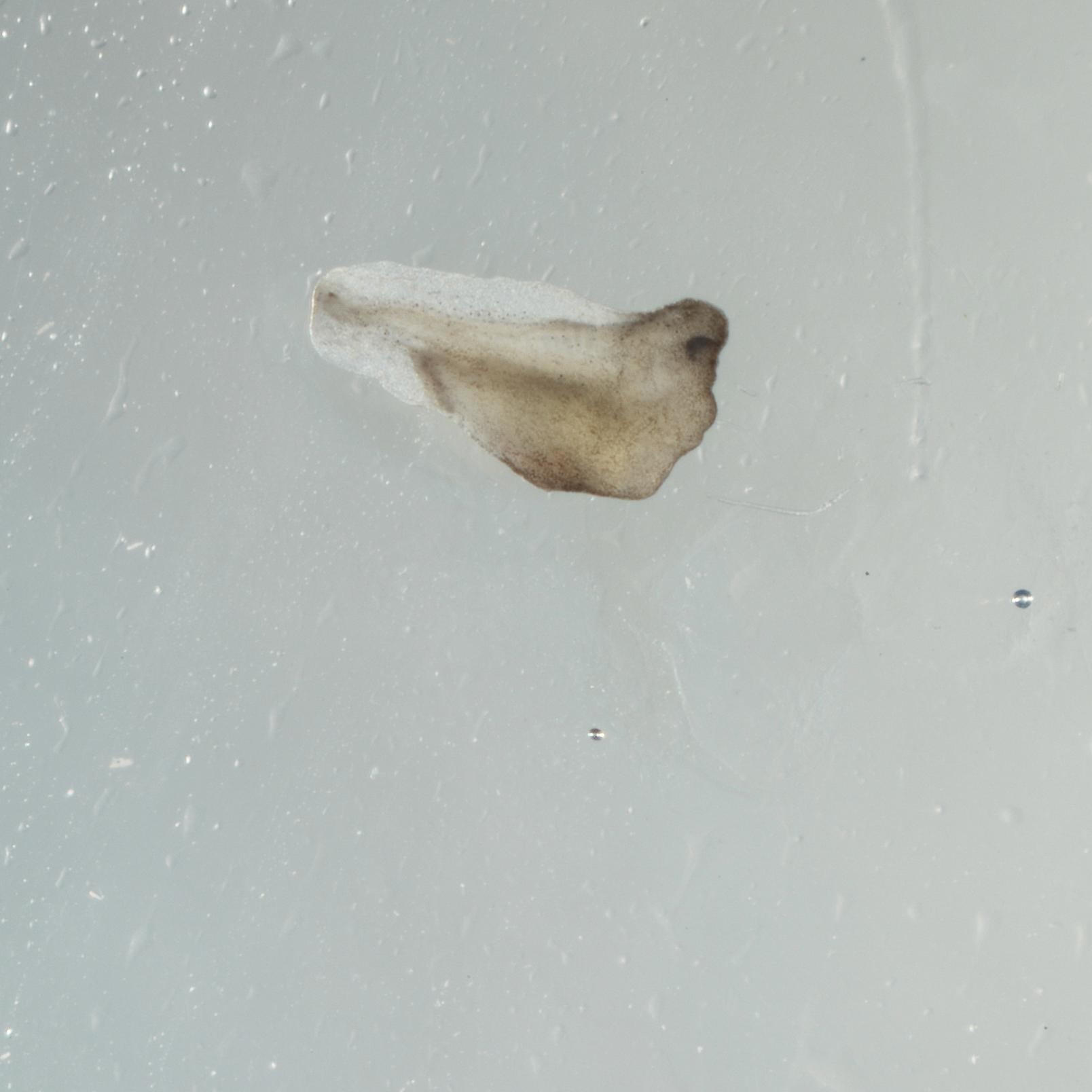

Supplement: Supplementary file 9 — Source Data EV Figures [file 44319_2023_46_MOESM9_ESM.zip › EV Figures/Figure EV4/EV4F/image EV4D severe.tif]

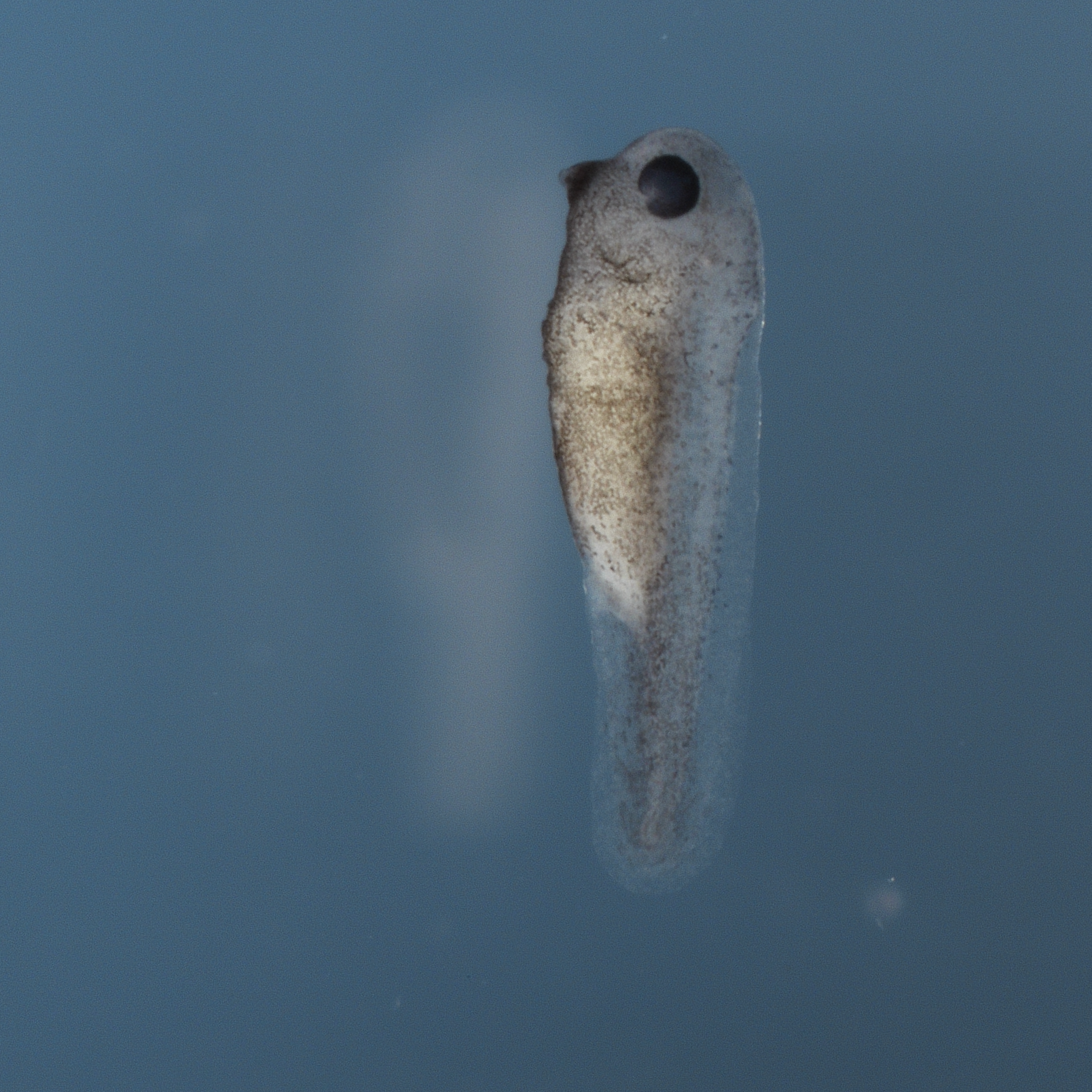

Supplement: Supplementary file 9 — Source Data EV Figures [file 44319_2023_46_MOESM9_ESM.zip › EV Figures/Figure EV4/EV4D/image F2-uninjected.tif]

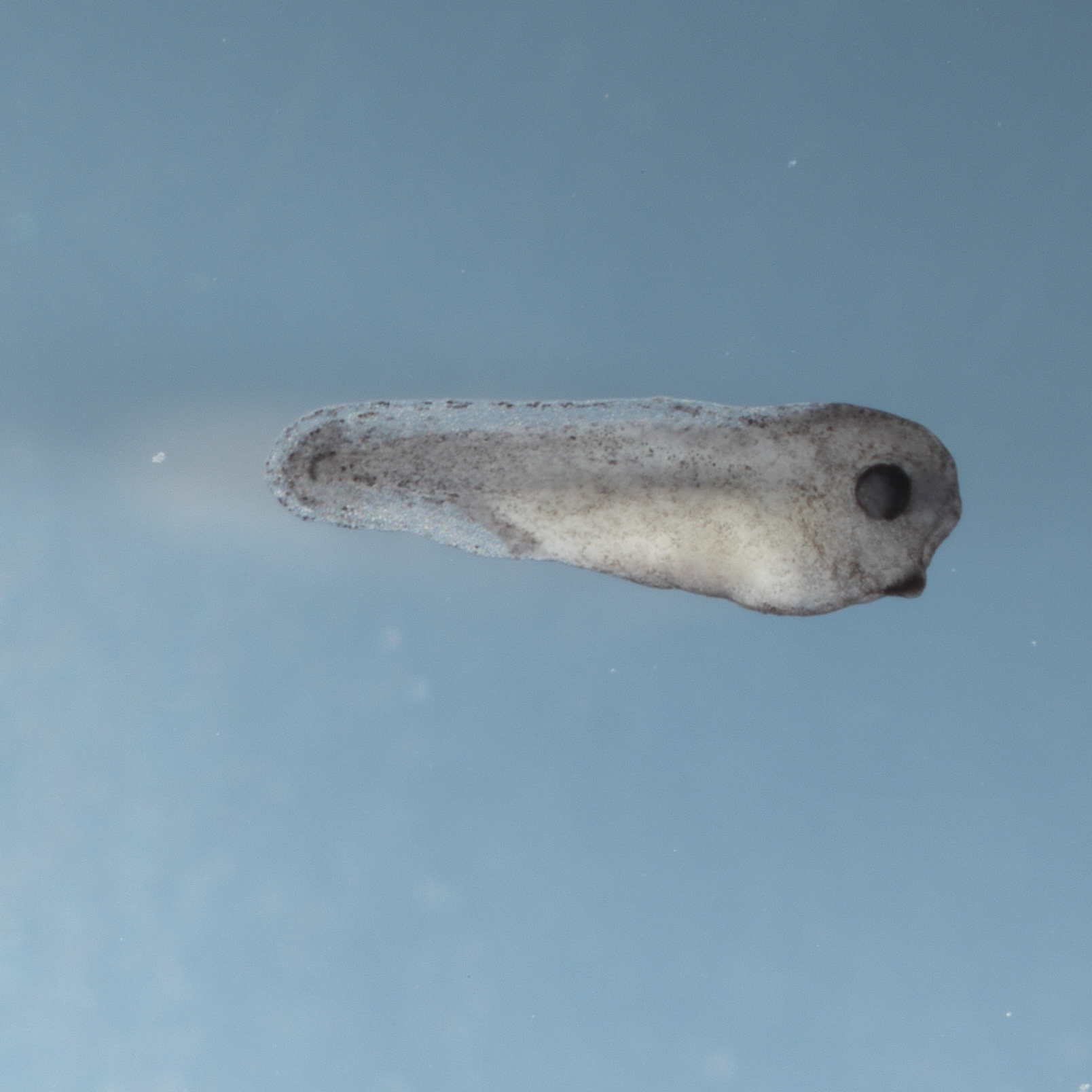

Supplement: Supplementary file 9 — Source Data EV Figures [file 44319_2023_46_MOESM9_ESM.zip › EV Figures/Figure EV4/EV4D/image WT-Z7MO.tif]

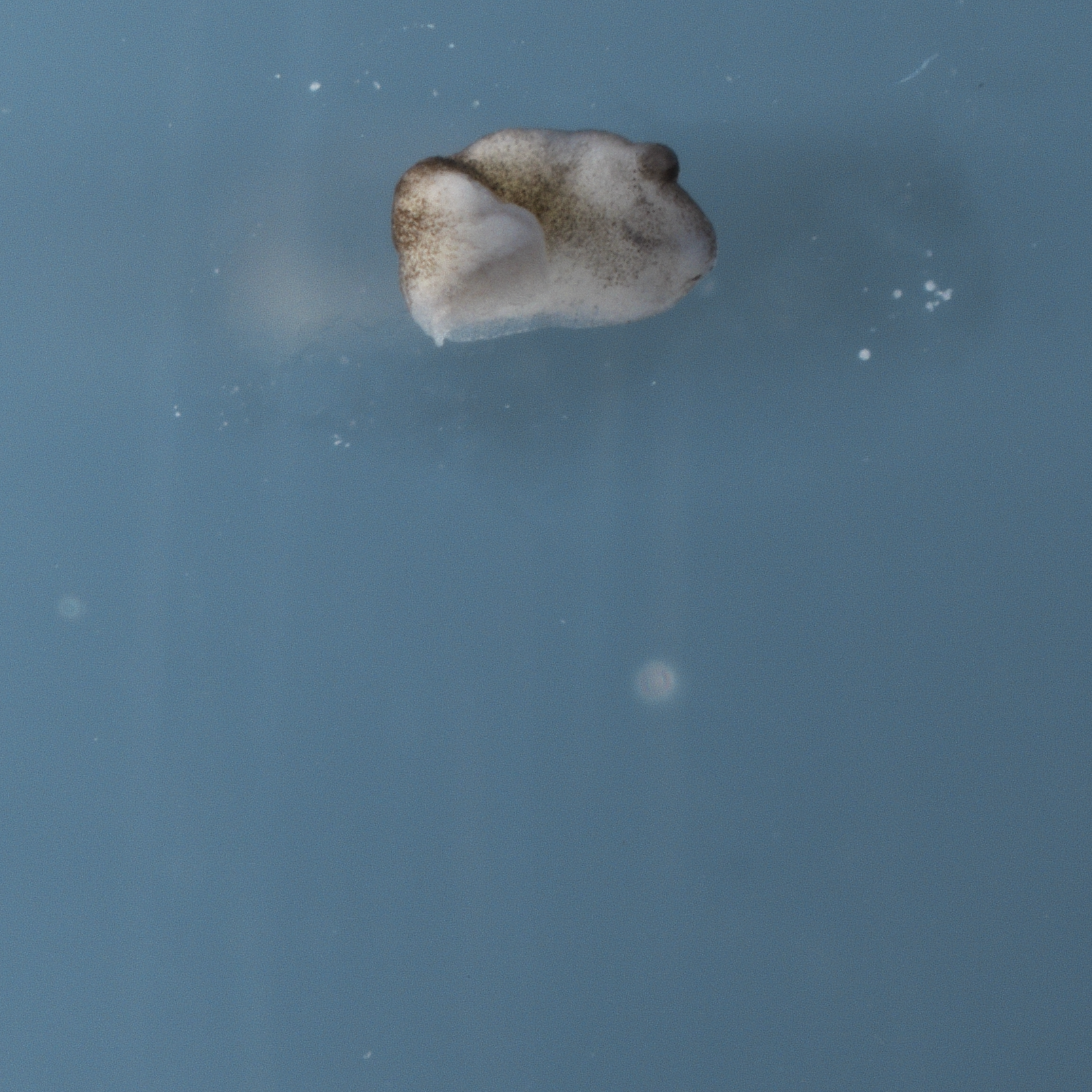

Supplement: Supplementary file 9 — Source Data EV Figures [file 44319_2023_46_MOESM9_ESM.zip › EV Figures/Figure EV4/EV4D/image F2-Z7MO-1.tif]

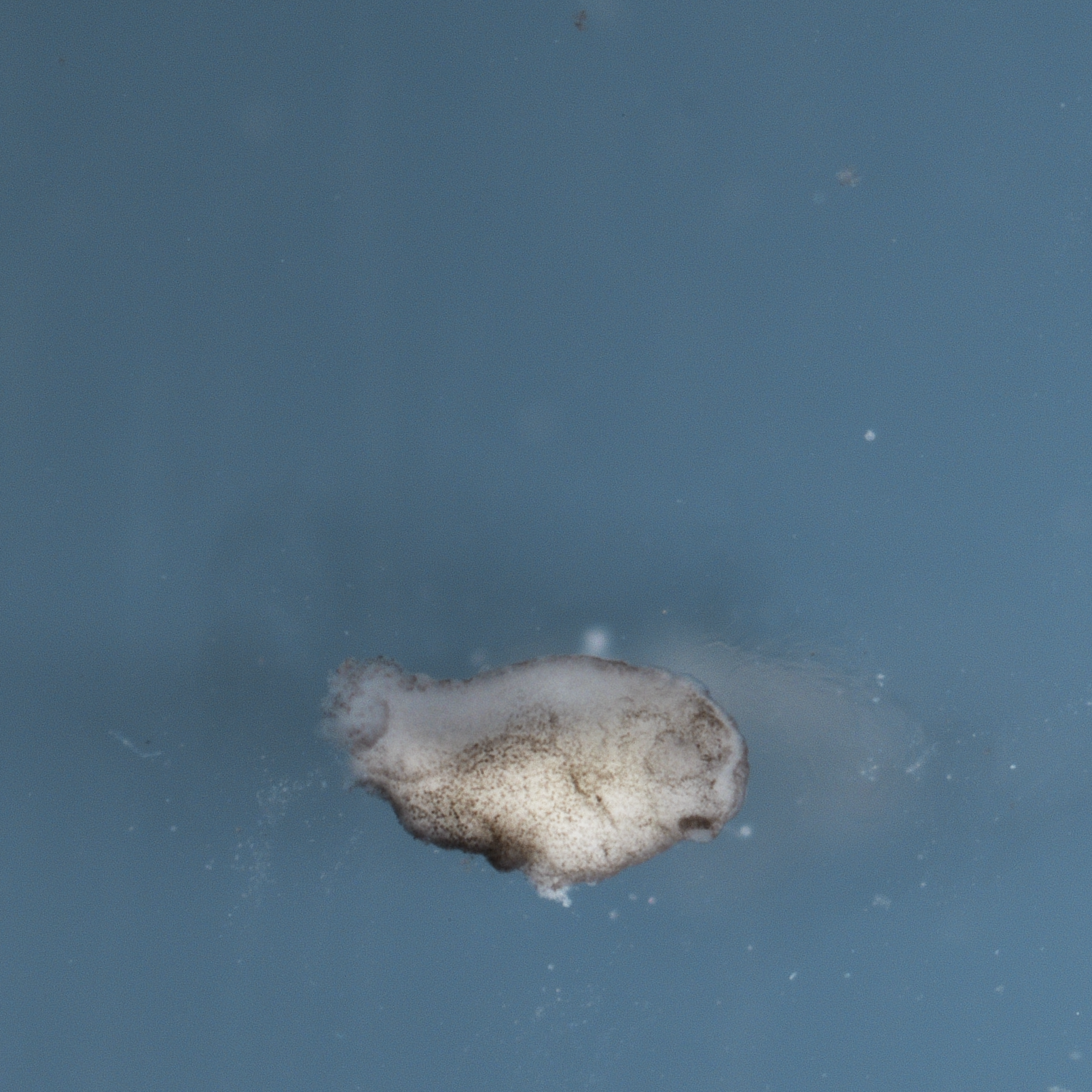

Supplement: Supplementary file 9 — Source Data EV Figures [file 44319_2023_46_MOESM9_ESM.zip › EV Figures/Figure EV4/EV4D/image F2-Z7MO-2.tif]

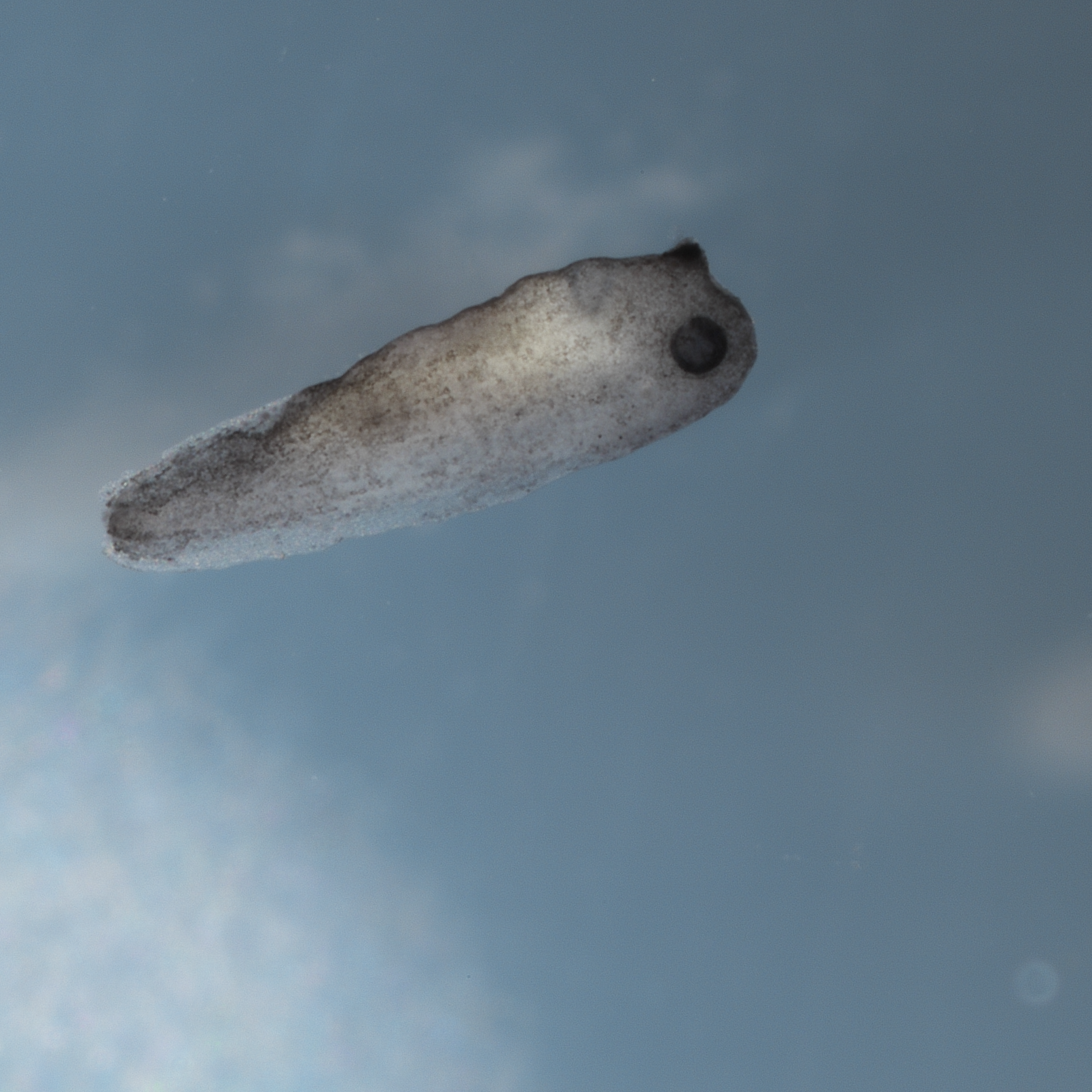

Supplement: Supplementary file 9 — Source Data EV Figures [file 44319_2023_46_MOESM9_ESM.zip › EV Figures/Figure EV4/EV4D/image WT-uninj.tif]

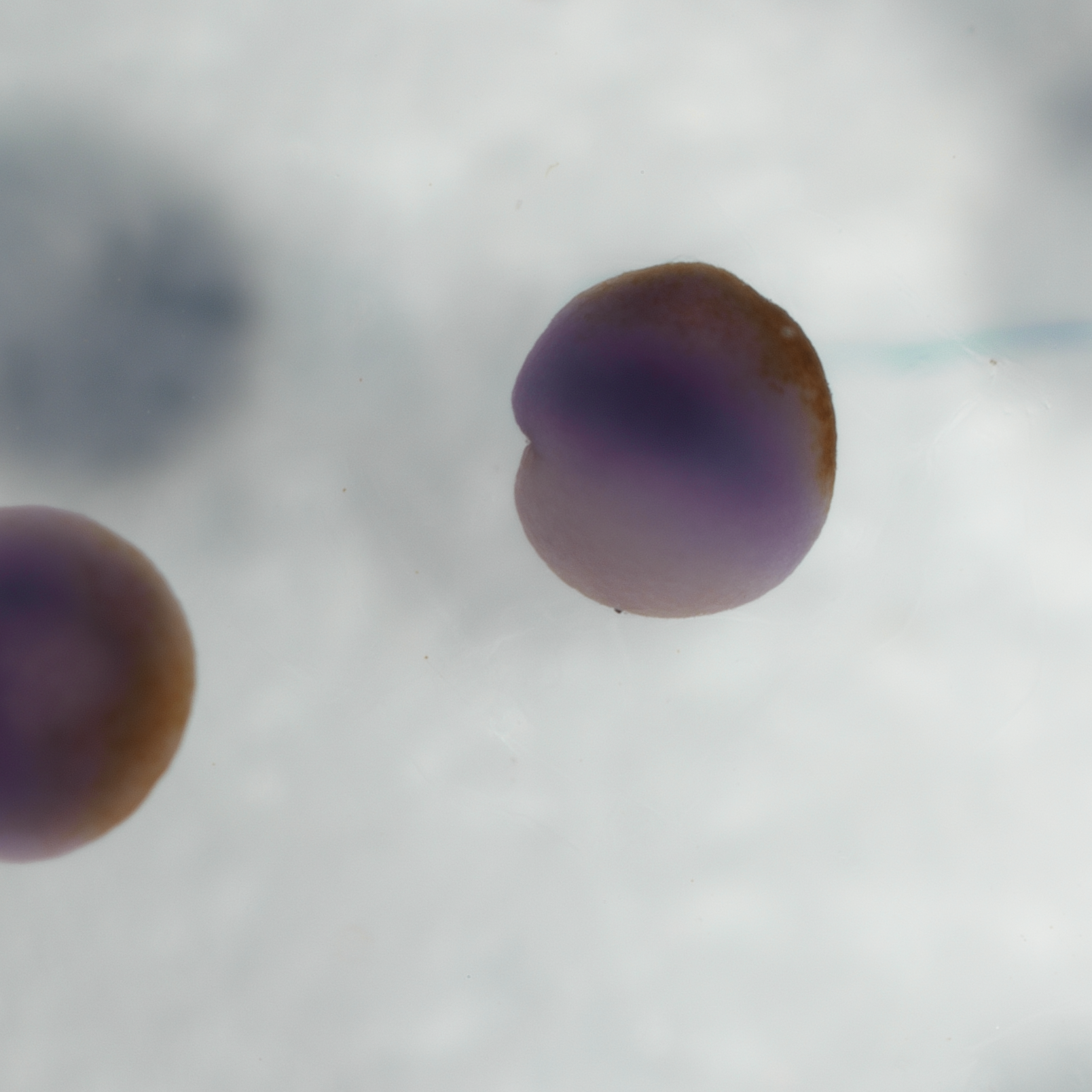

Supplement: Supplementary file 9 — Source Data EV Figures [file 44319_2023_46_MOESM9_ESM.zip › EV Figures/Figure EV4/EV4B/image zswim4F2 lateral view.tif]

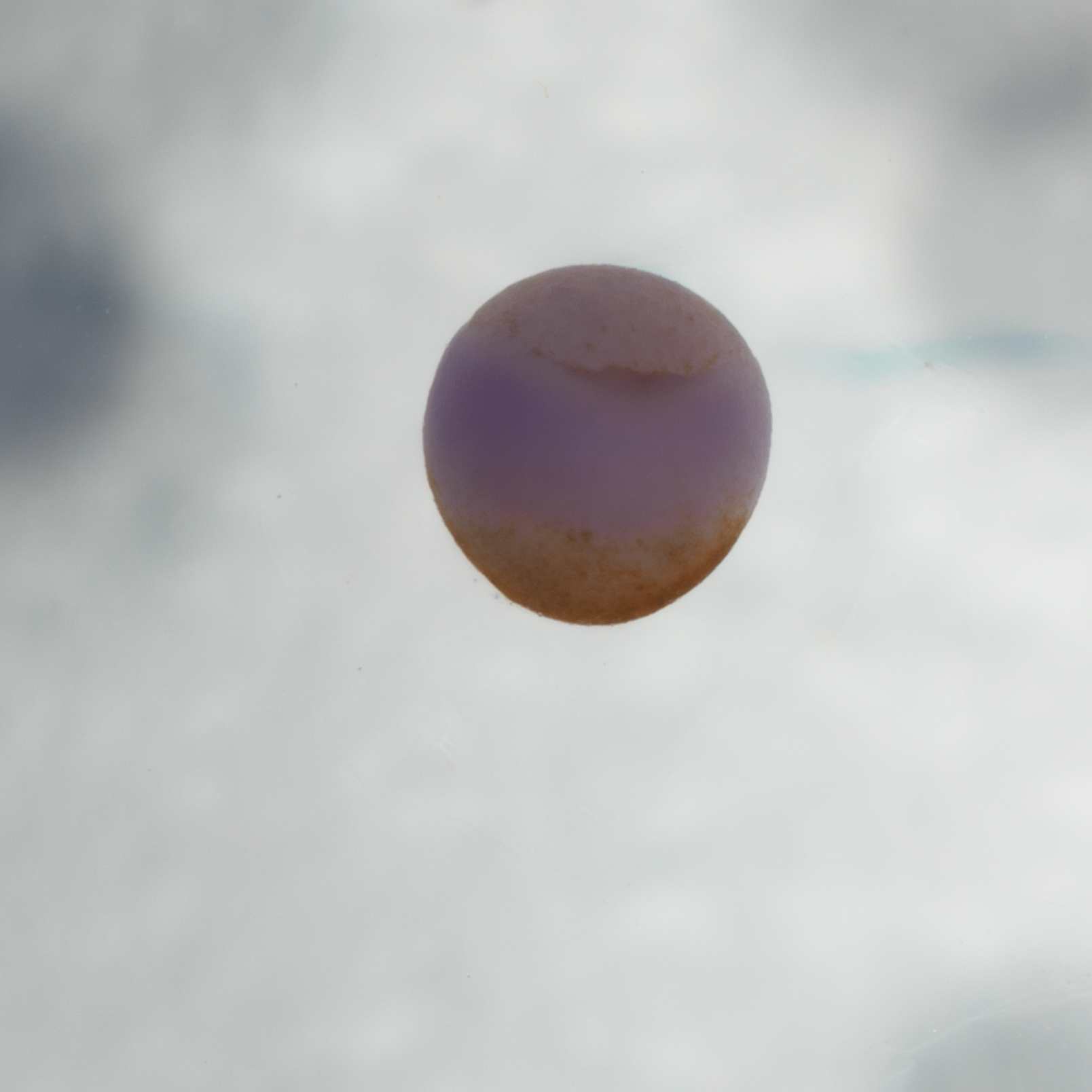

Supplement: Supplementary file 9 — Source Data EV Figures [file 44319_2023_46_MOESM9_ESM.zip › EV Figures/Figure EV4/EV4B/image WT dorsal view.tif]

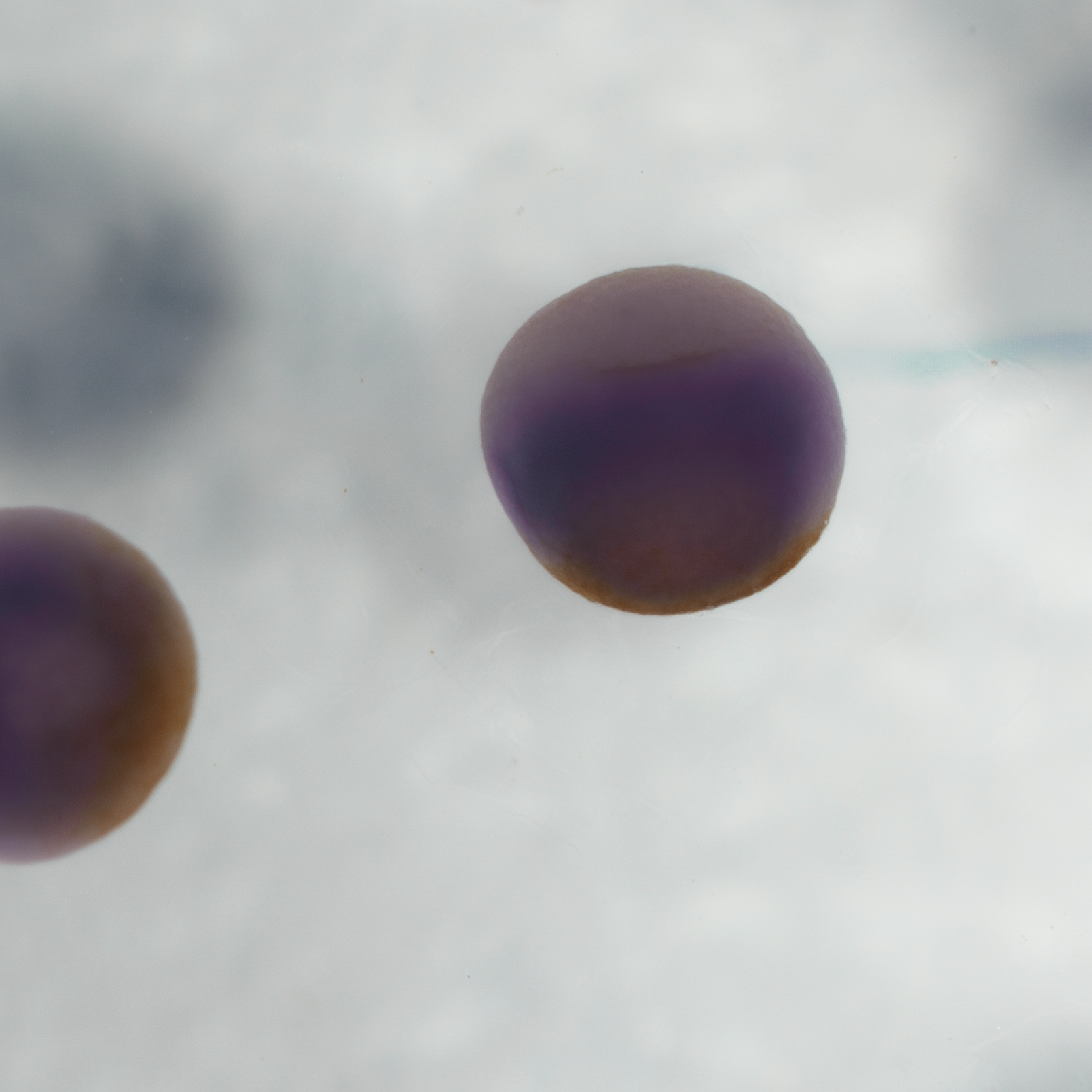

Supplement: Supplementary file 9 — Source Data EV Figures [file 44319_2023_46_MOESM9_ESM.zip › EV Figures/Figure EV4/EV4B/image zswim4F2 dorsal view.tif]

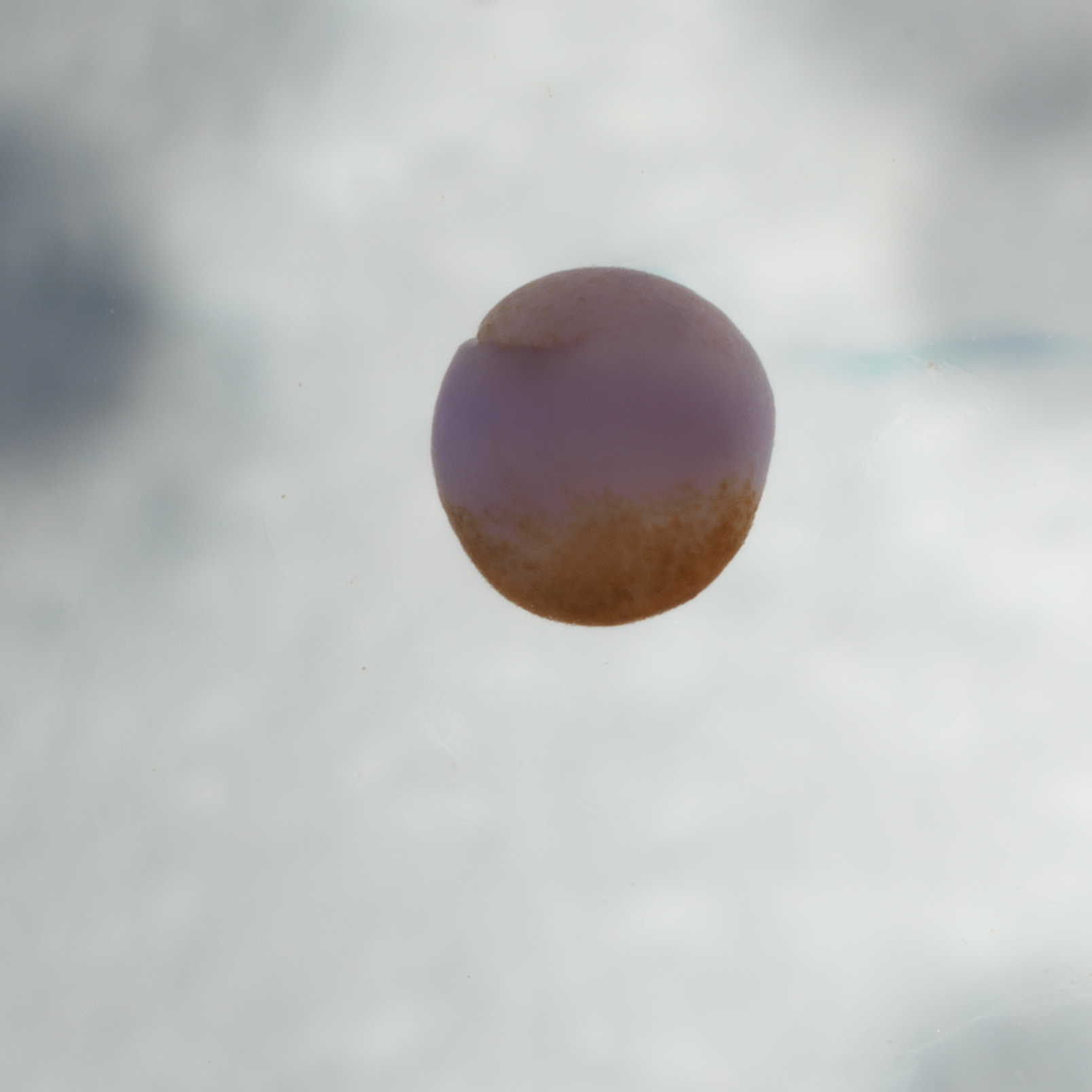

Supplement: Supplementary file 9 — Source Data EV Figures [file 44319_2023_46_MOESM9_ESM.zip › EV Figures/Figure EV4/EV4B/image WT lateral view.tif]

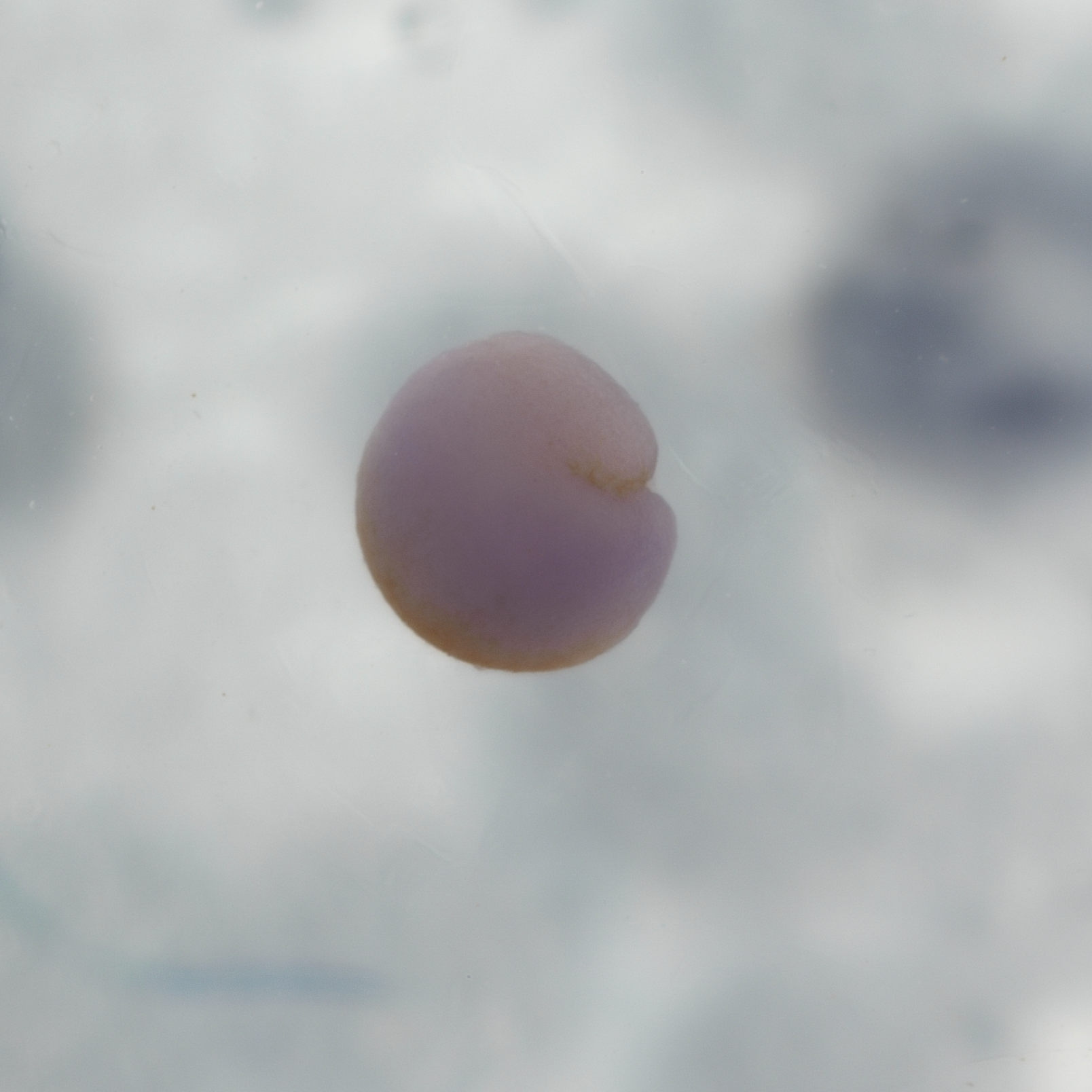

Supplement: Supplementary file 9 — Source Data EV Figures [file 44319_2023_46_MOESM9_ESM.zip › EV Figures/Figure EV4/EV4B/image zswim4-crispant lateral view.tif]

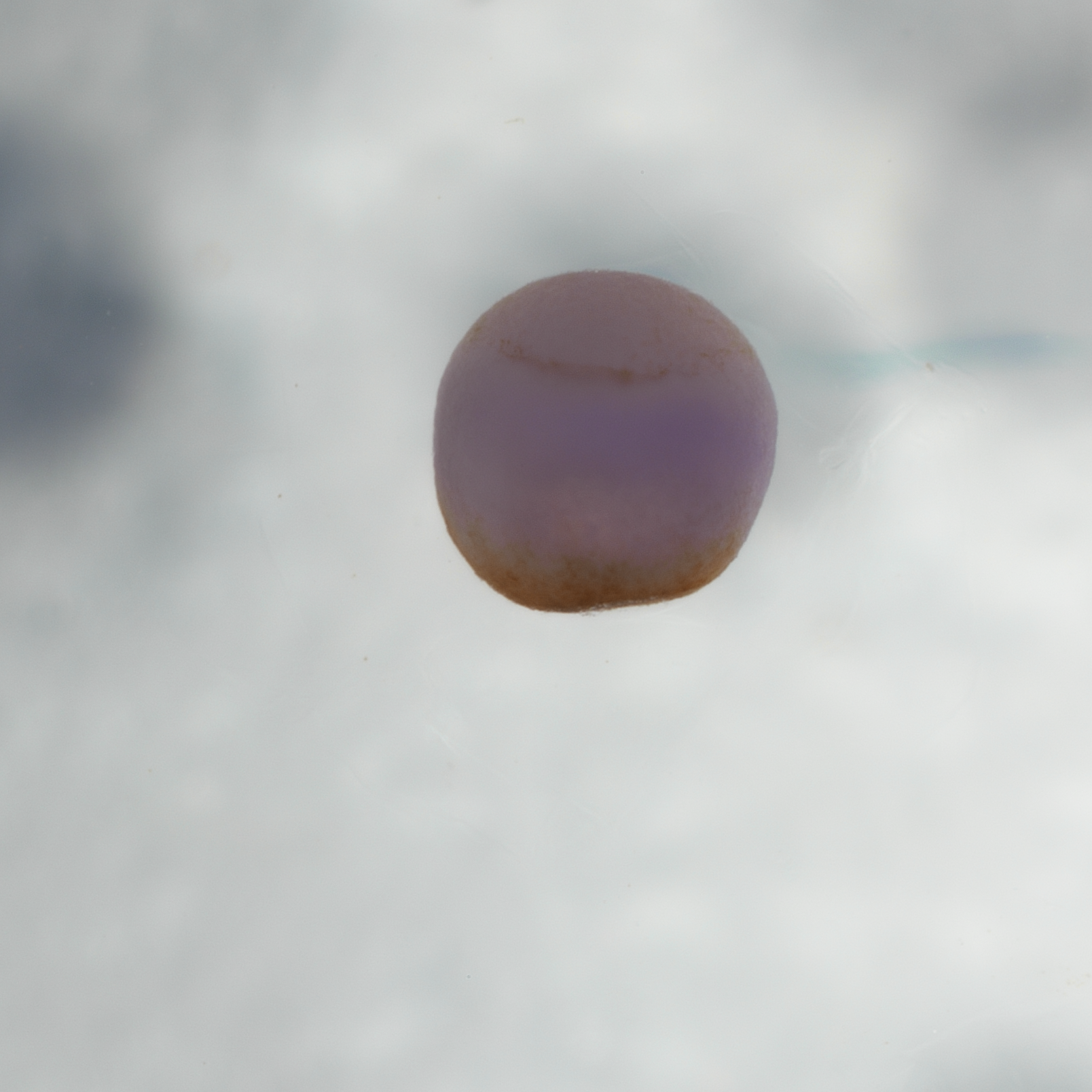

Supplement: Supplementary file 9 — Source Data EV Figures [file 44319_2023_46_MOESM9_ESM.zip › EV Figures/Figure EV4/EV4B/image zswim4-crispant dorsal view.tif]

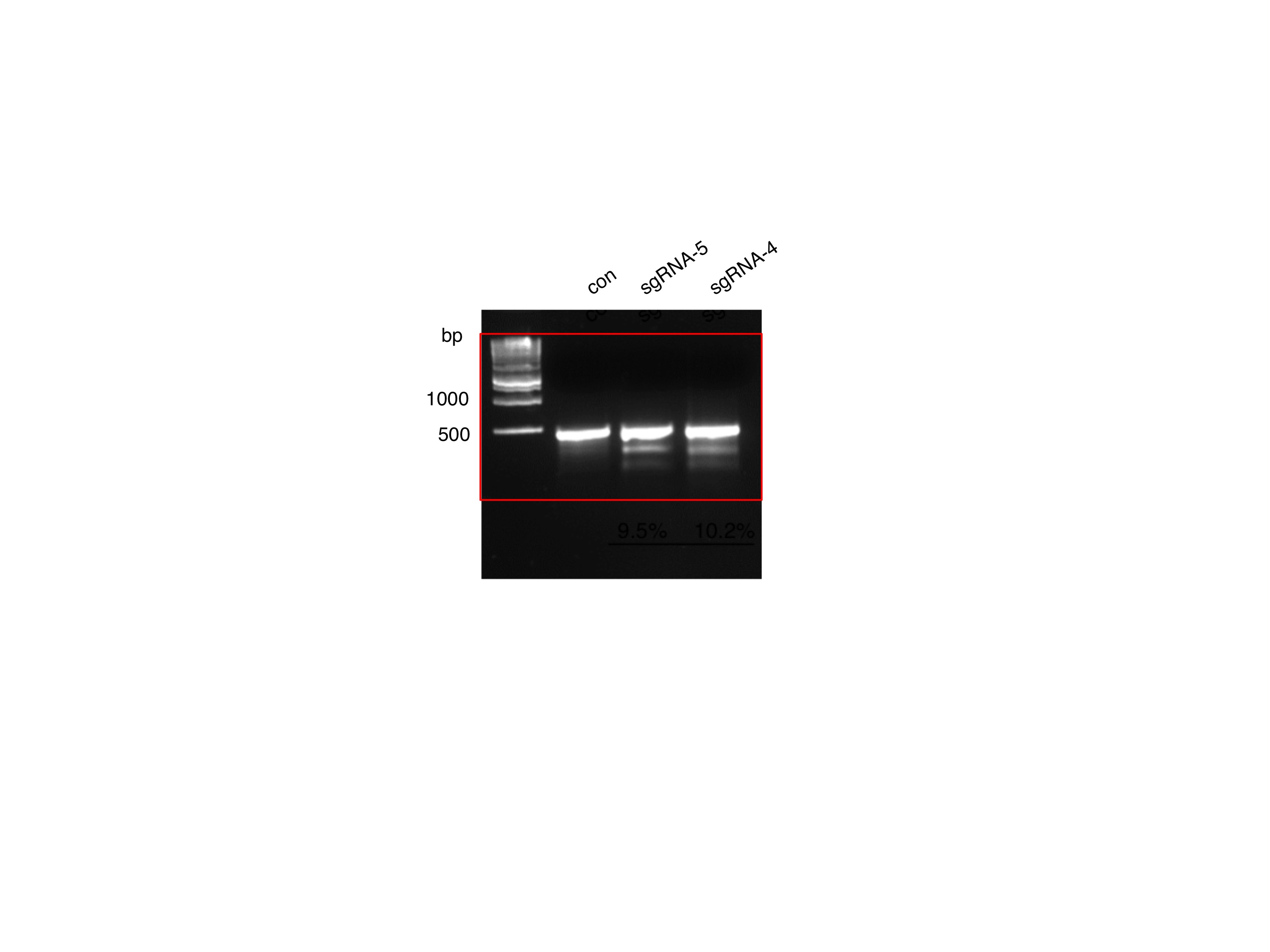

Supplement: Supplementary file 9 — Source Data EV Figures [file 44319_2023_46_MOESM9_ESM.zip › EV Figures/Figure EV3/EV3C/Presentation1.jpg]

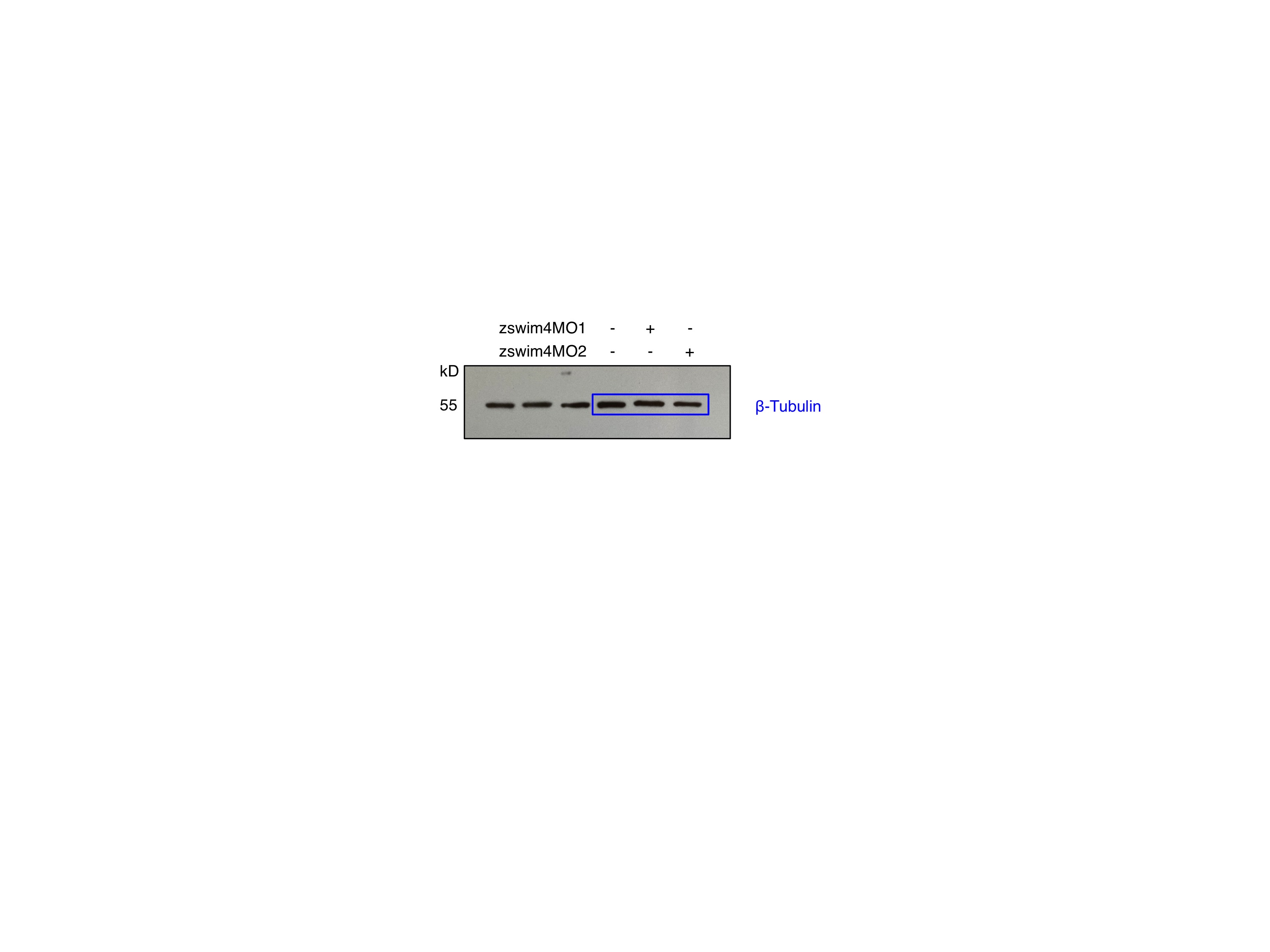

Supplement: Supplementary file 9 — Source Data EV Figures [file 44319_2023_46_MOESM9_ESM.zip › EV Figures/Figure EV2/EV2F/western EV2F b-tubulin.jpg]

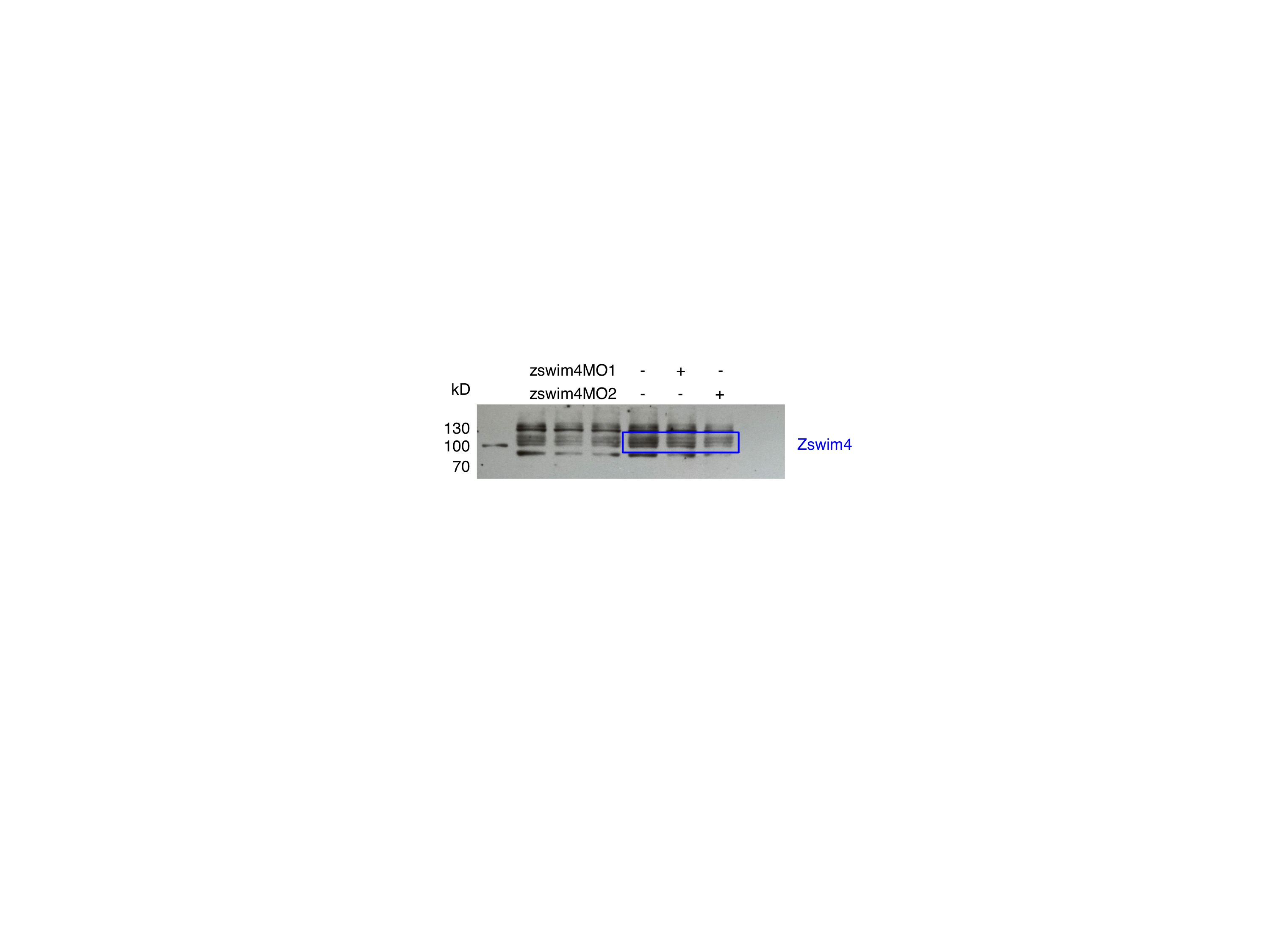

Supplement: Supplementary file 9 — Source Data EV Figures [file 44319_2023_46_MOESM9_ESM.zip › EV Figures/Figure EV2/EV2F/western EV2F Zswim4.jpg]

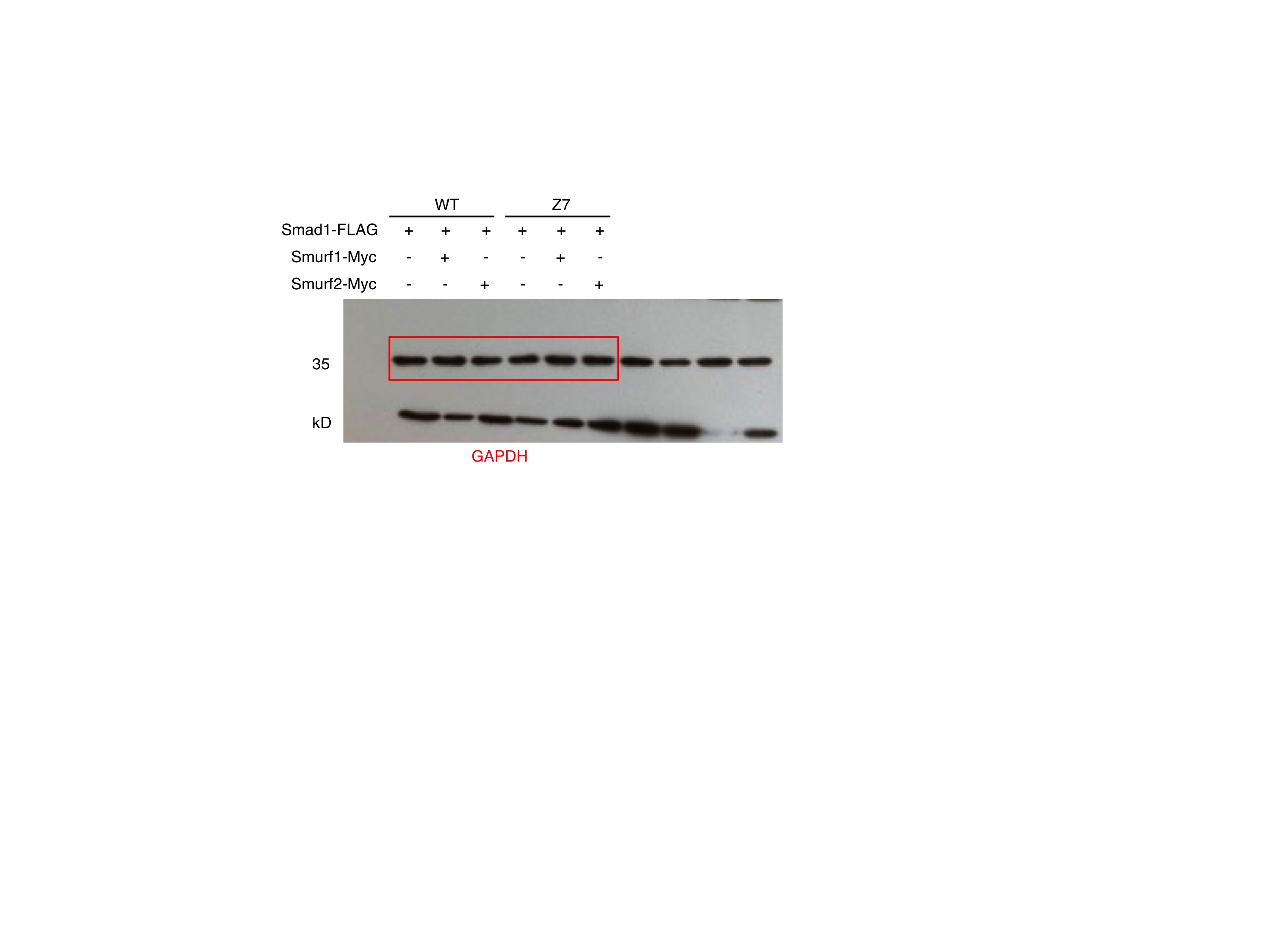

Supplement: Supplementary file 9 — Source Data EV Figures [file 44319_2023_46_MOESM9_ESM.zip › EV Figures/Figure EV5/EV5J/western GAPDH.jpg]

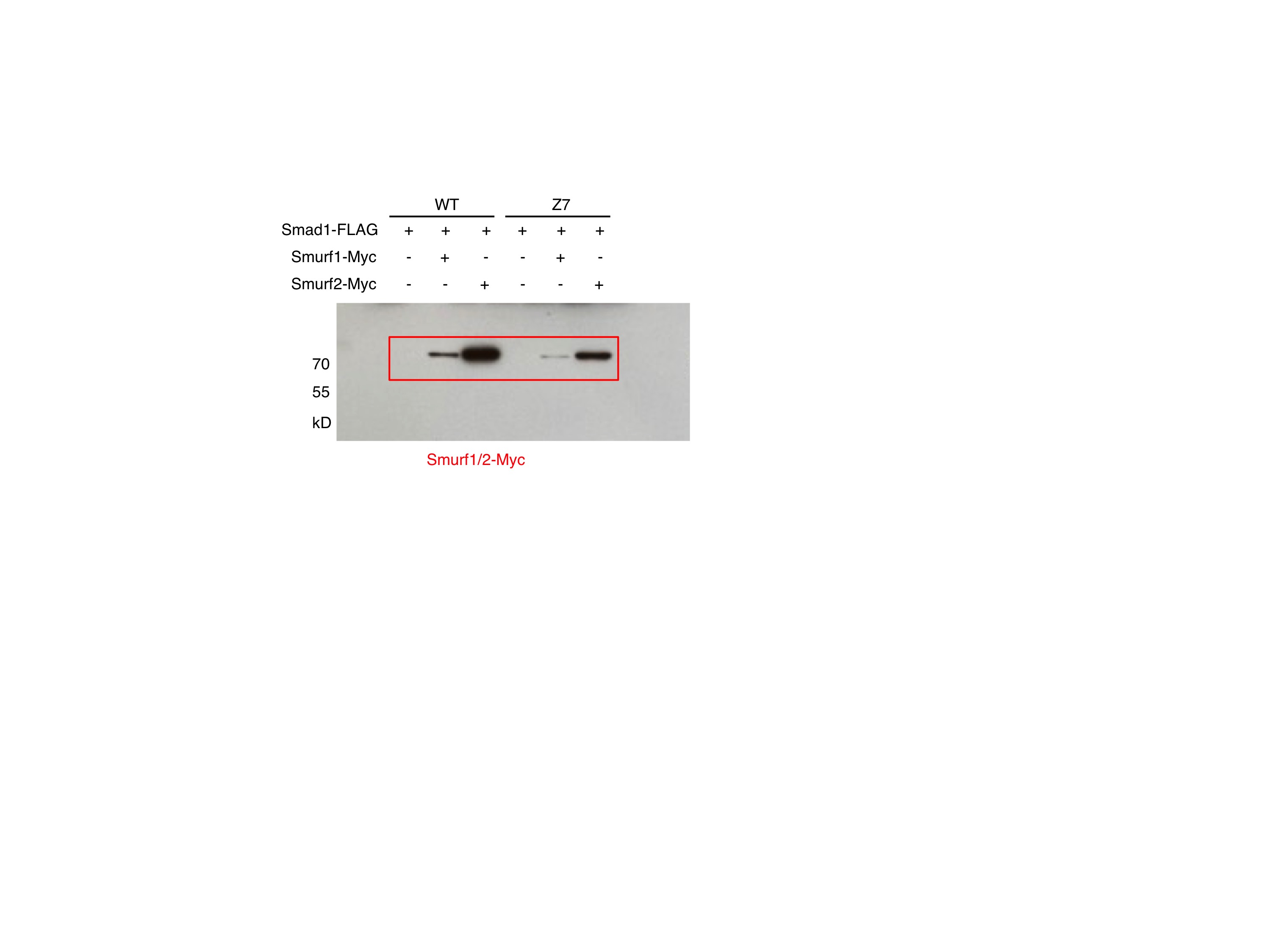

Supplement: Supplementary file 9 — Source Data EV Figures [file 44319_2023_46_MOESM9_ESM.zip › EV Figures/Figure EV5/EV5J/western Smurf1-2-Myc.jpg]

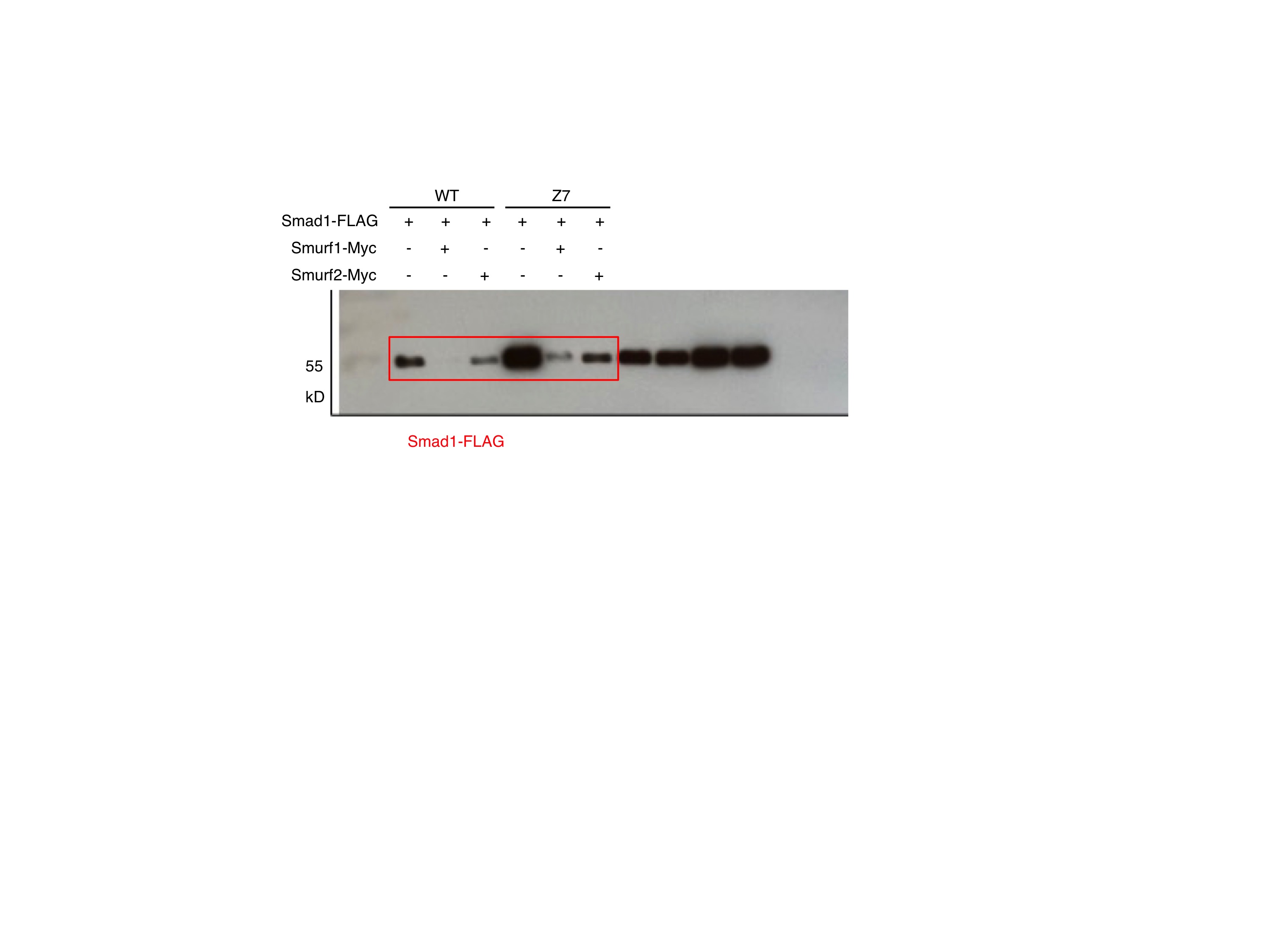

Supplement: Supplementary file 9 — Source Data EV Figures [file 44319_2023_46_MOESM9_ESM.zip › EV Figures/Figure EV5/EV5J/western Smad1-FLAG.jpg]

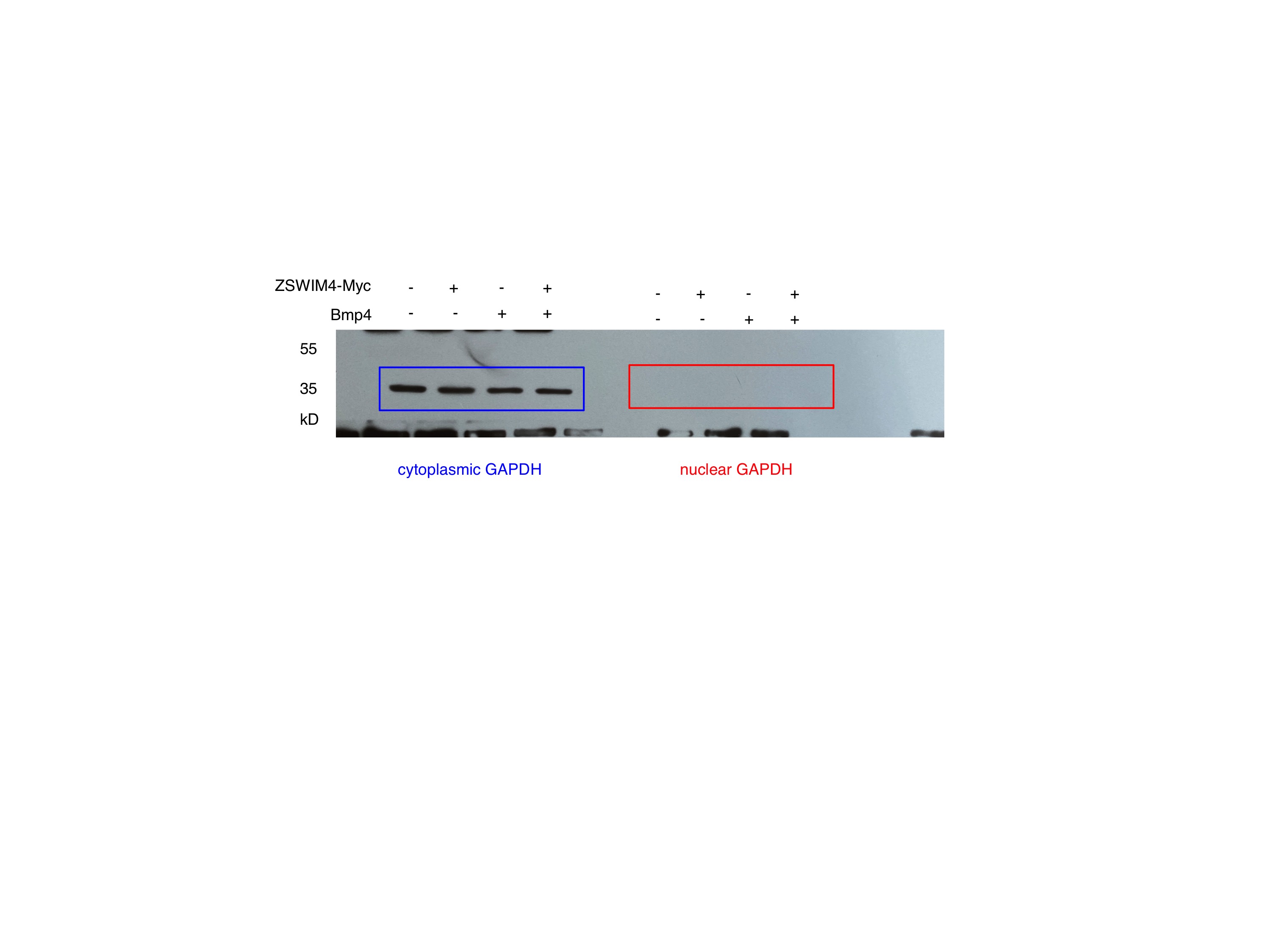

Supplement: Supplementary file 9 — Source Data EV Figures [file 44319_2023_46_MOESM9_ESM.zip › EV Figures/Figure EV5/EV5G/western GAPDH.jpg]

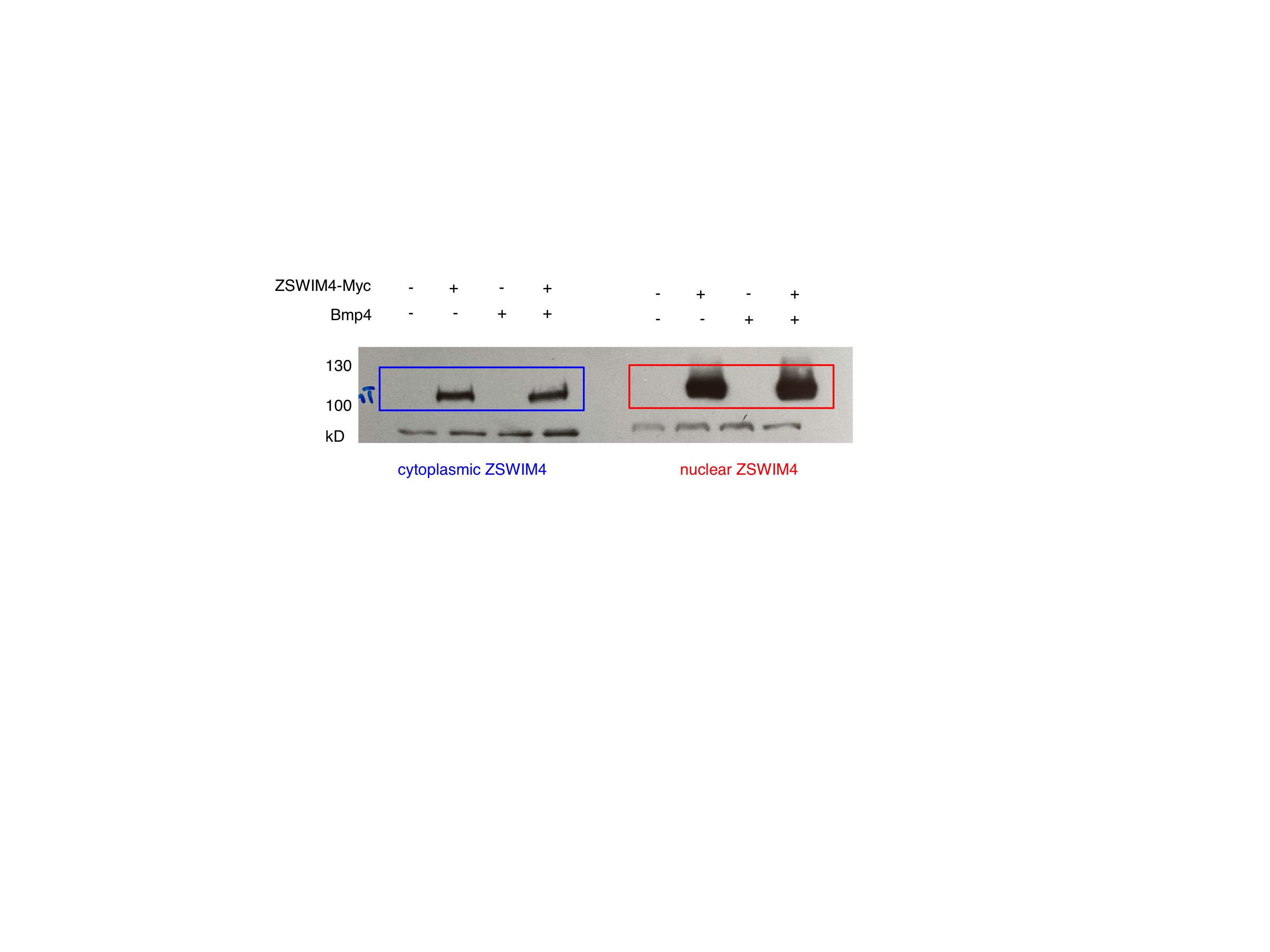

Supplement: Supplementary file 9 — Source Data EV Figures [file 44319_2023_46_MOESM9_ESM.zip › EV Figures/Figure EV5/EV5G/western ZSWIM4Myc.jpg]

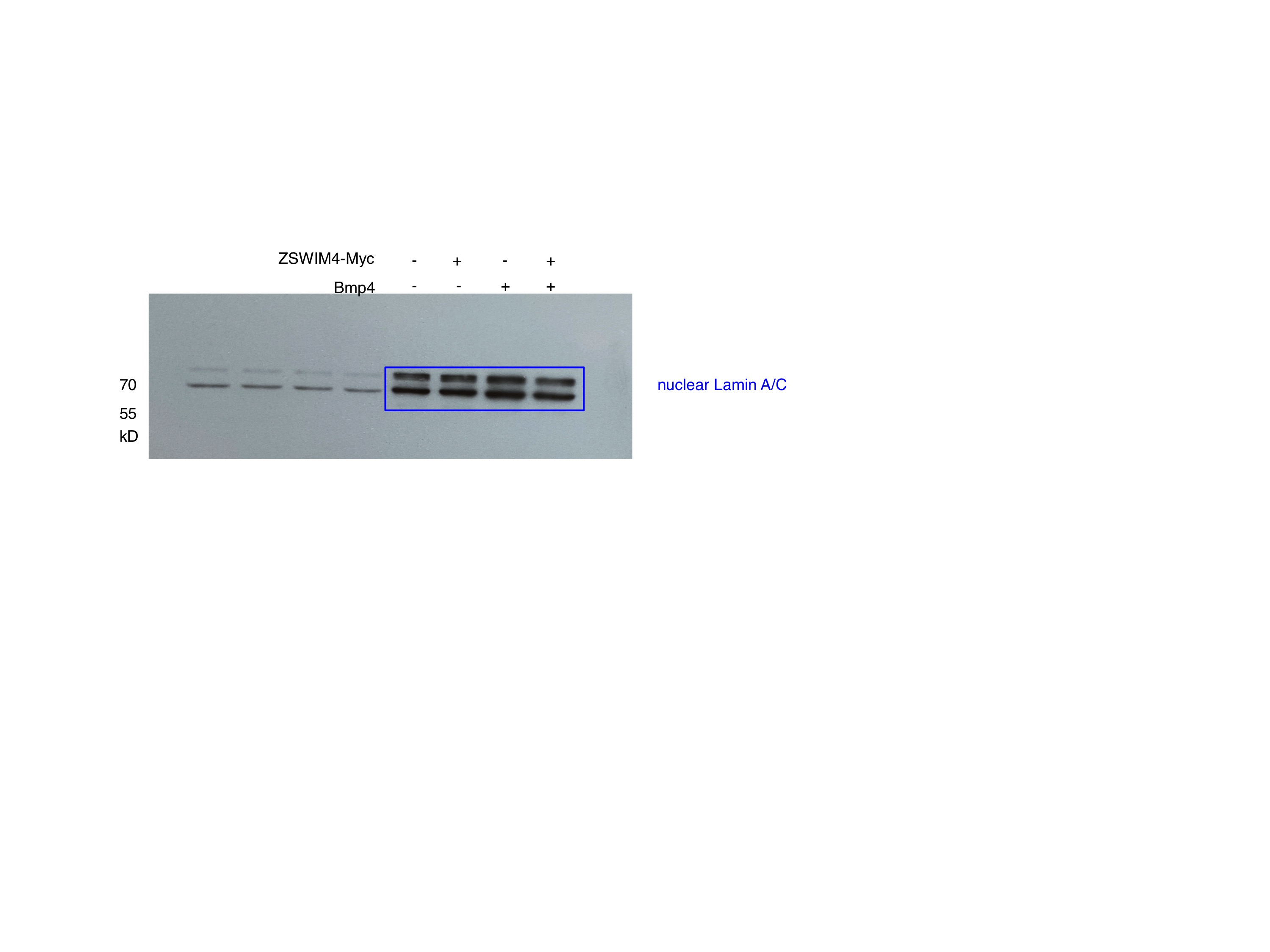

Supplement: Supplementary file 9 — Source Data EV Figures [file 44319_2023_46_MOESM9_ESM.zip › EV Figures/Figure EV5/EV5G/western nuclear Lamin AC.jpg]

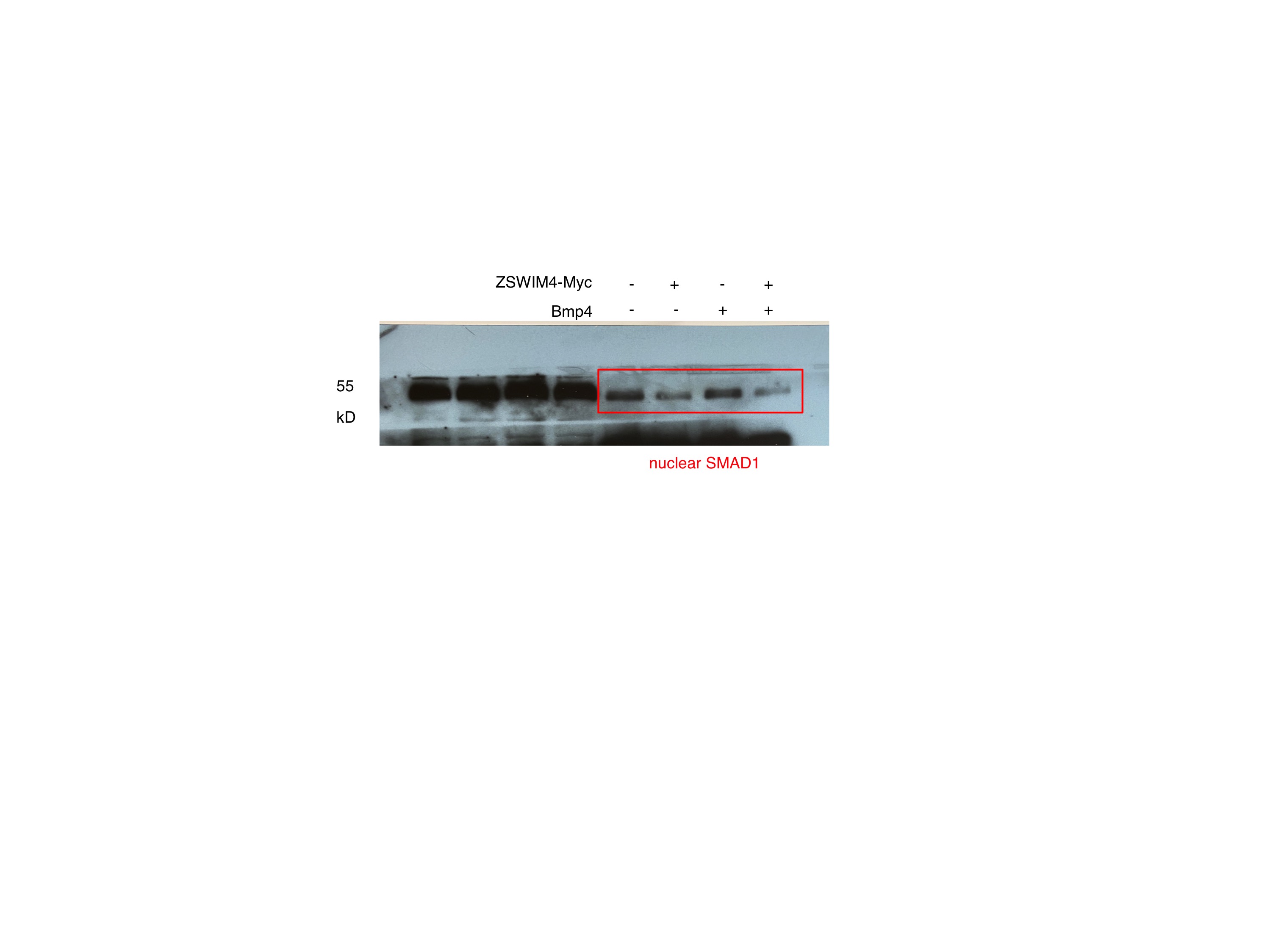

Supplement: Supplementary file 9 — Source Data EV Figures [file 44319_2023_46_MOESM9_ESM.zip › EV Figures/Figure EV5/EV5G/western nuclear SMAD1.jpg]

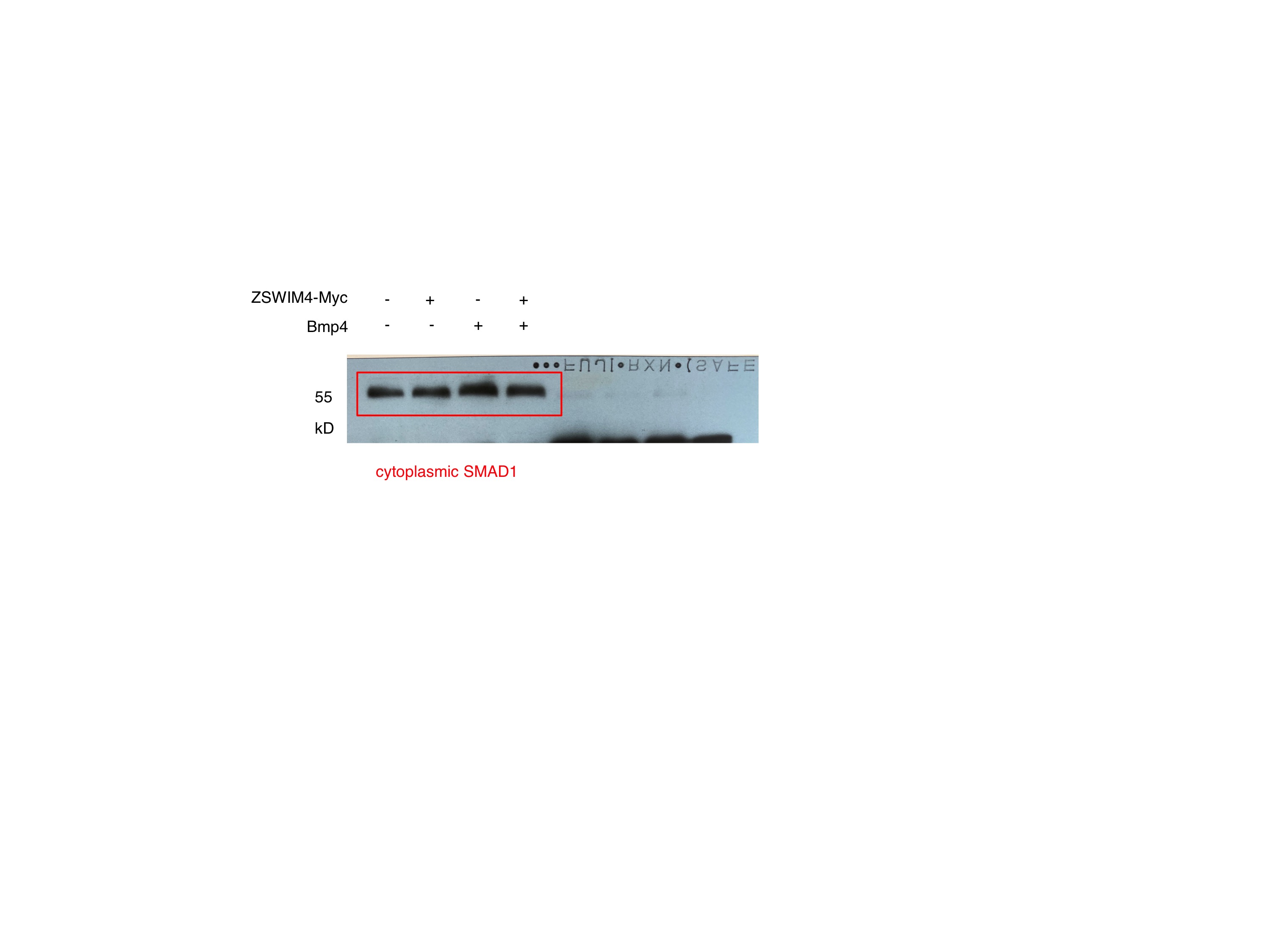

Supplement: Supplementary file 9 — Source Data EV Figures [file 44319_2023_46_MOESM9_ESM.zip › EV Figures/Figure EV5/EV5G/western cytoplasmic SMAD1.jpg]

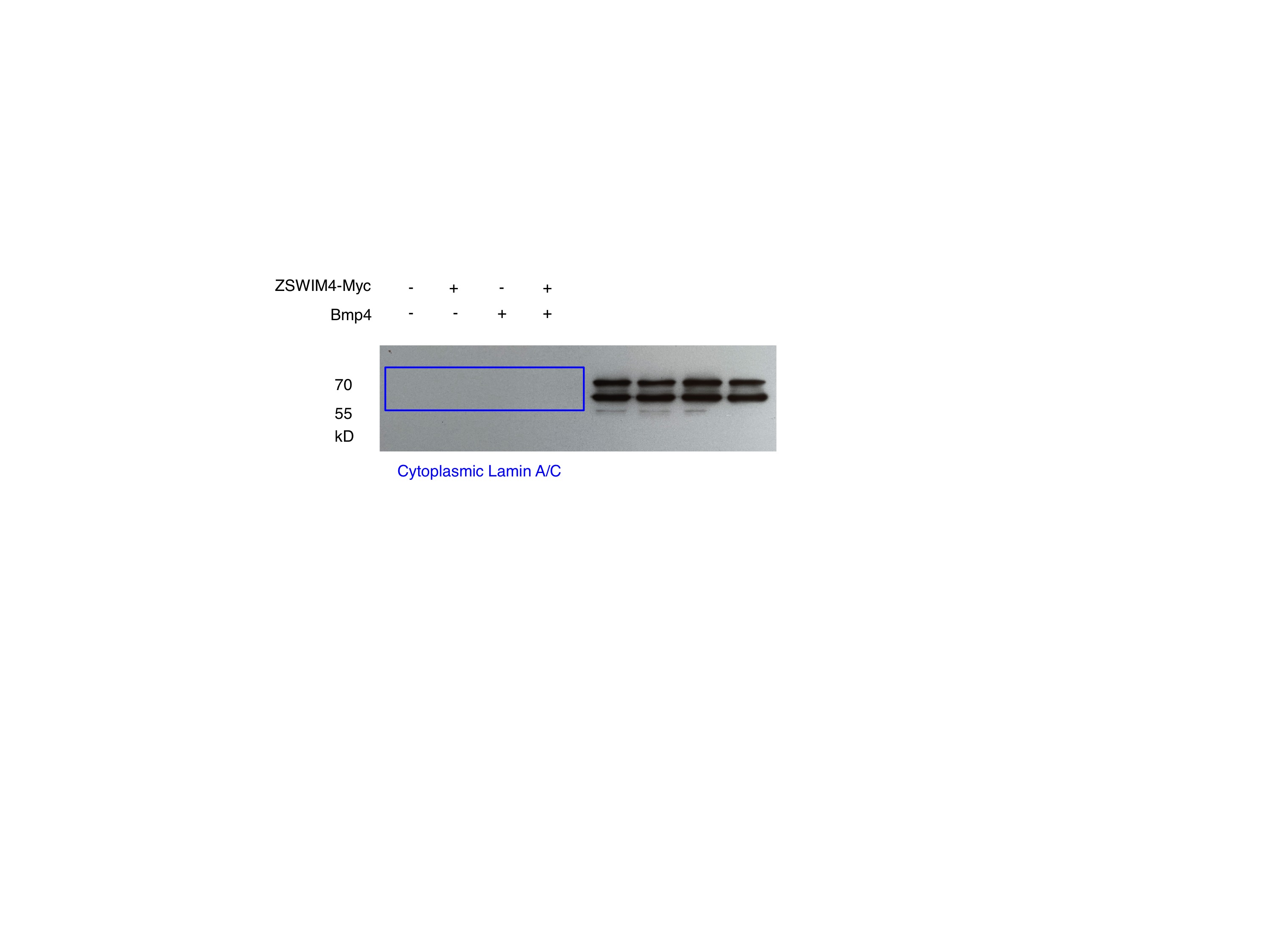

Supplement: Supplementary file 9 — Source Data EV Figures [file 44319_2023_46_MOESM9_ESM.zip › EV Figures/Figure EV5/EV5G/western cytoplasmic Lamin AC.jpg]

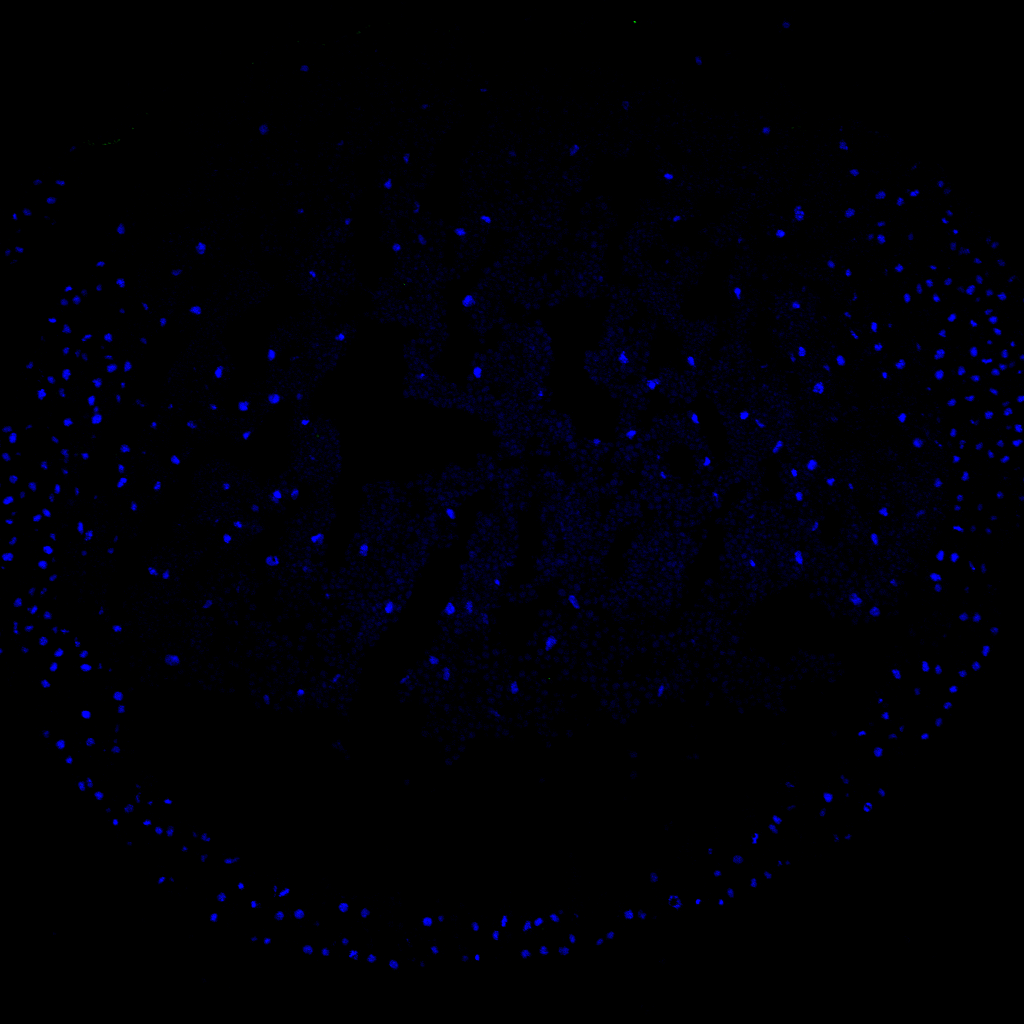

Supplement: Supplementary file 9 — Source Data EV Figures [file 44319_2023_46_MOESM9_ESM.zip › EV Figures/Figure EV1/EV1M/image EV1M-IgG-merge.tif]

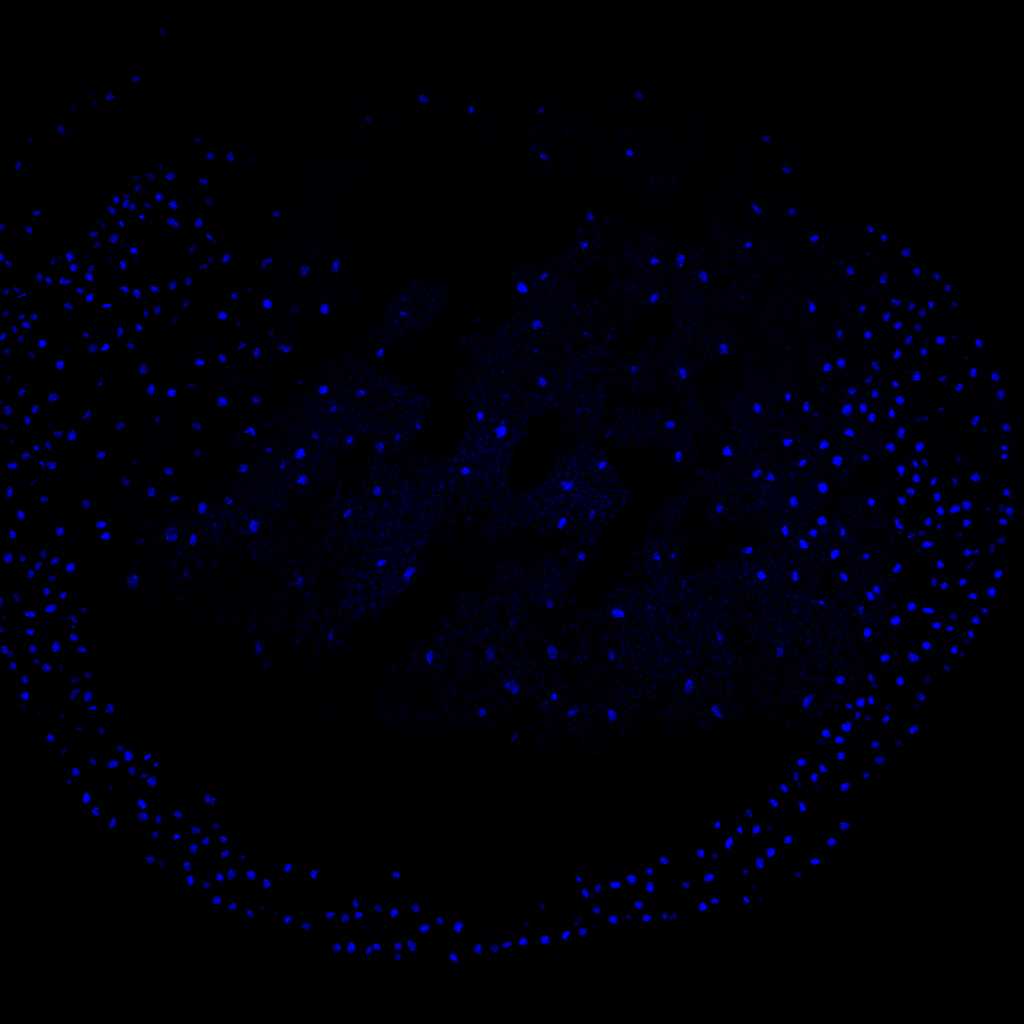

Supplement: Supplementary file 9 — Source Data EV Figures [file 44319_2023_46_MOESM9_ESM.zip › EV Figures/Figure EV1/EV1M/image EV1M-antizswim4-DAPI.tif]

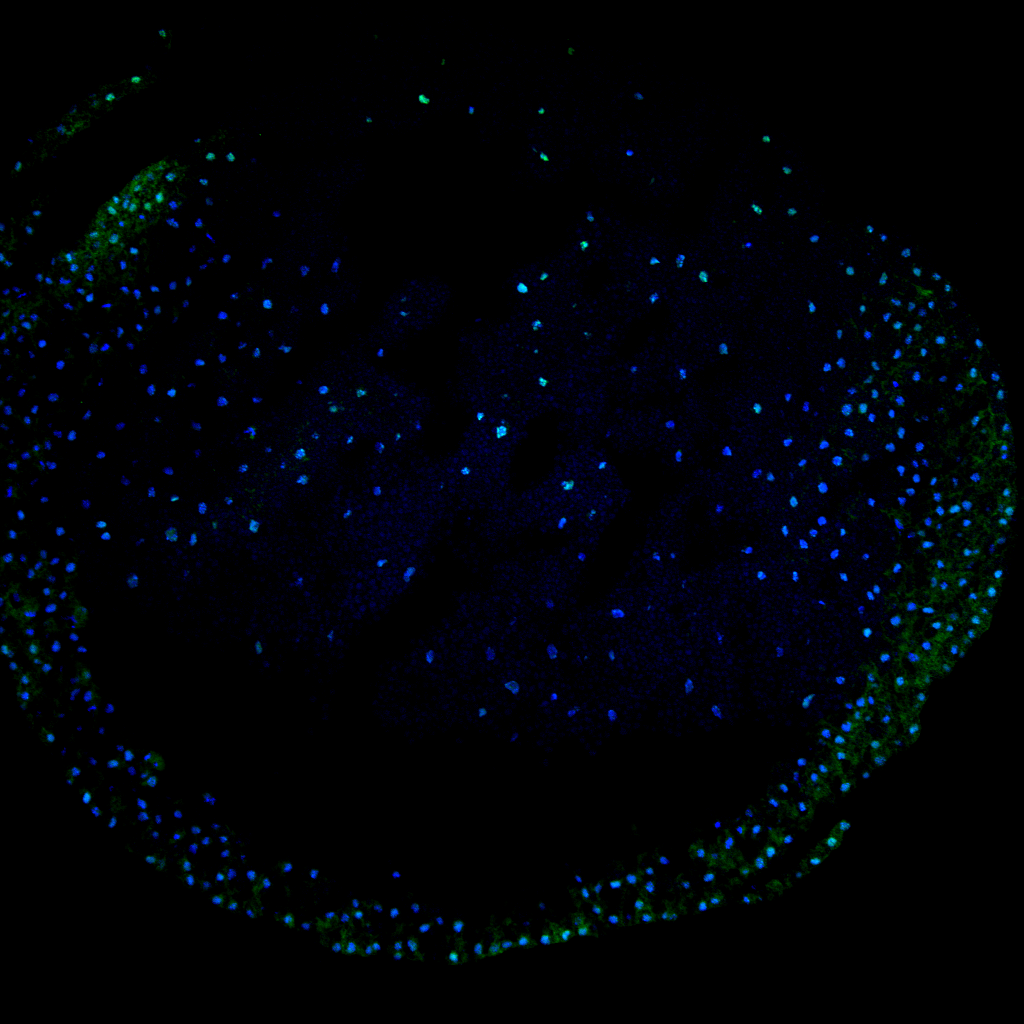

Supplement: Supplementary file 9 — Source Data EV Figures [file 44319_2023_46_MOESM9_ESM.zip › EV Figures/Figure EV1/EV1M/image EV1M-antizswim4-merge.tif]

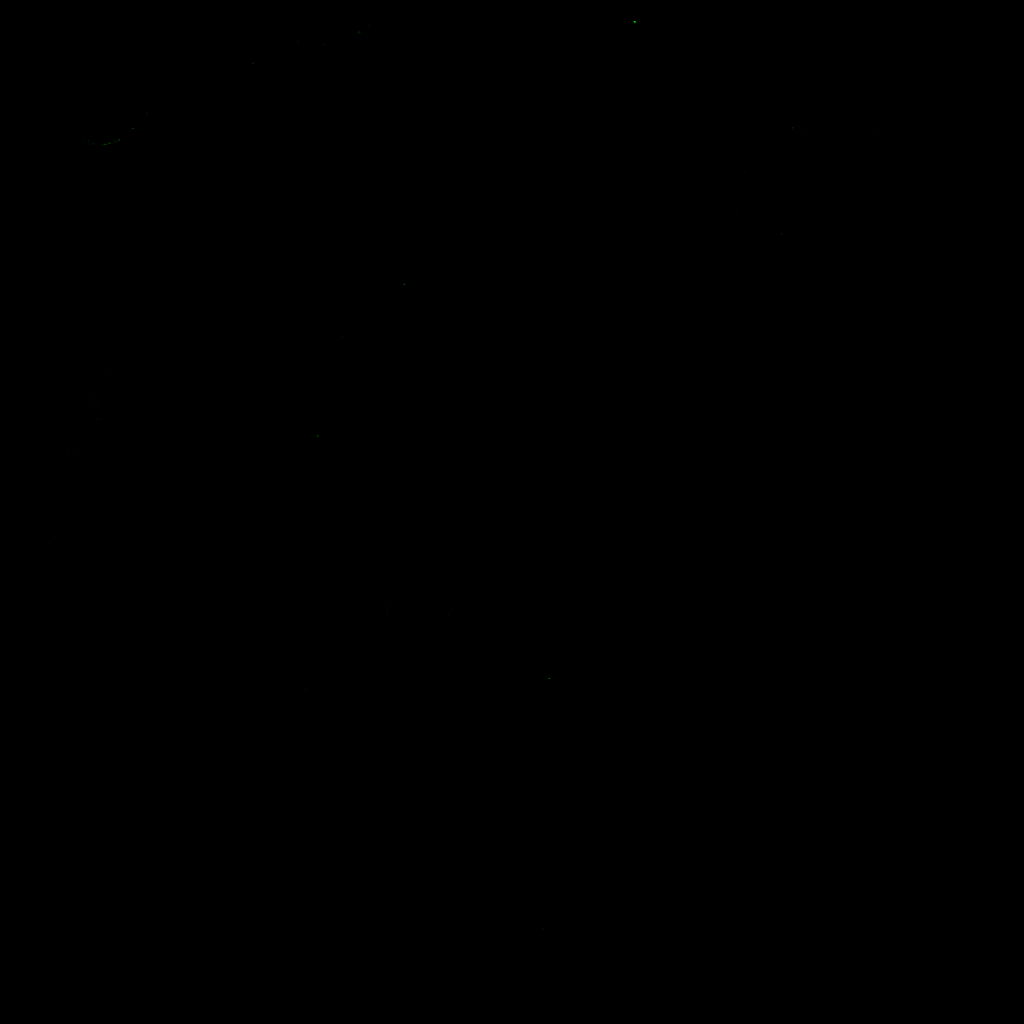

Supplement: Supplementary file 9 — Source Data EV Figures [file 44319_2023_46_MOESM9_ESM.zip › EV Figures/Figure EV1/EV1M/image EV1M-IgG-zswim4.tif]

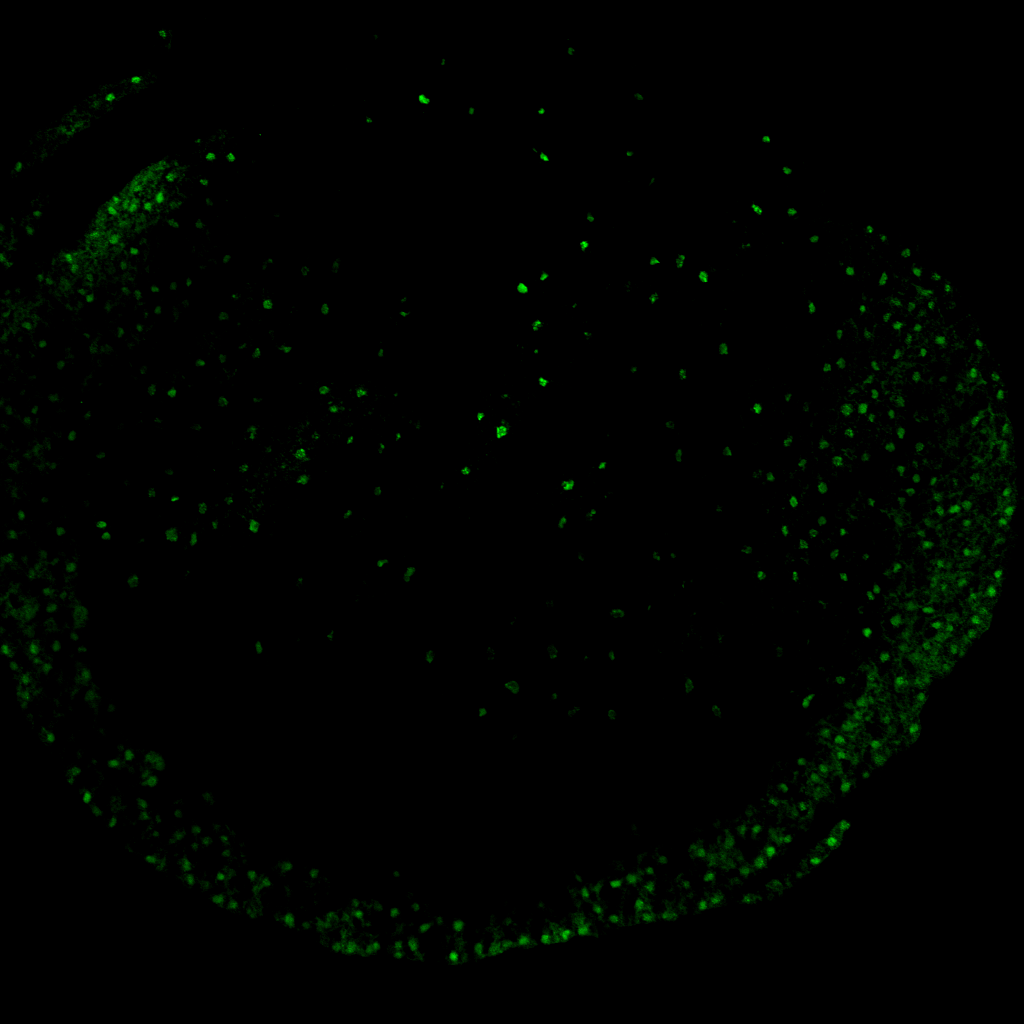

Supplement: Supplementary file 9 — Source Data EV Figures [file 44319_2023_46_MOESM9_ESM.zip › EV Figures/Figure EV1/EV1M/image EV1M-antizswim4-zswim4.tif]

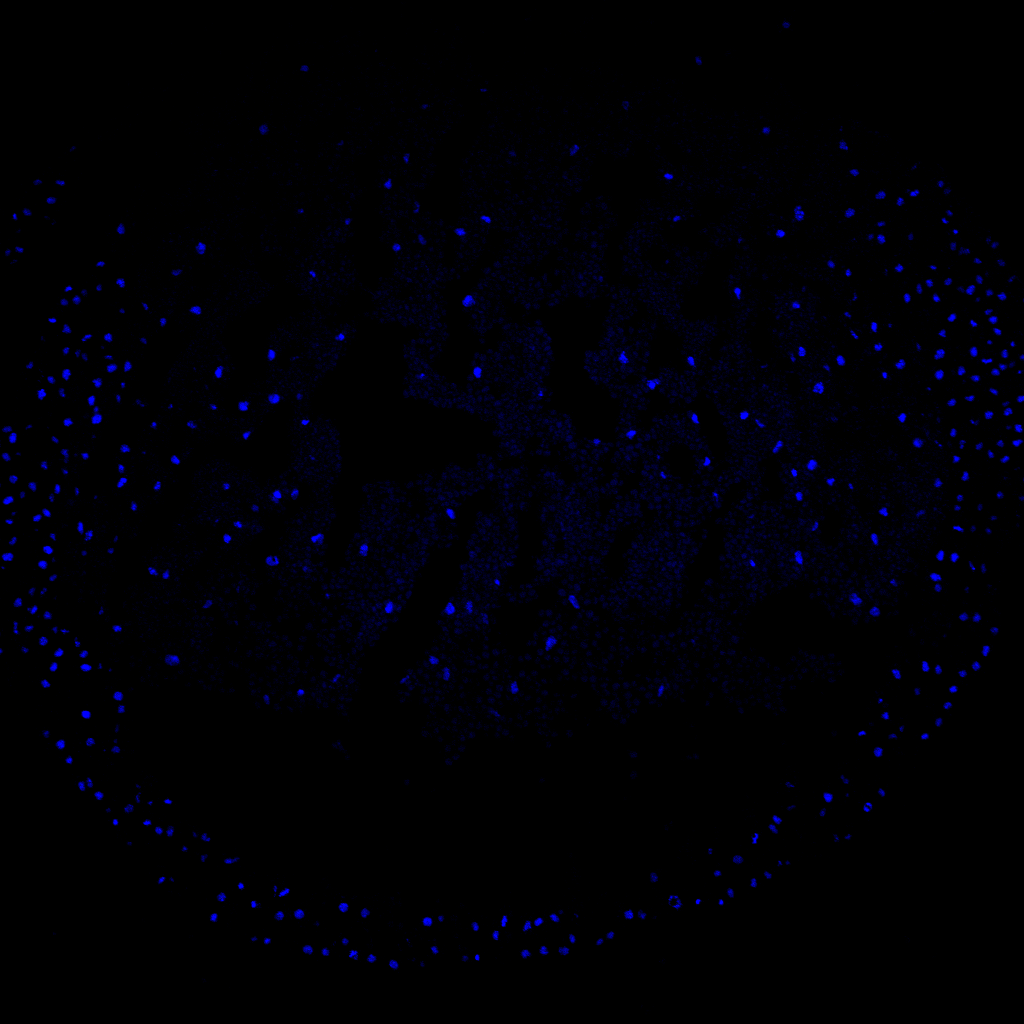

Supplement: Supplementary file 9 — Source Data EV Figures [file 44319_2023_46_MOESM9_ESM.zip › EV Figures/Figure EV1/EV1M/image EV1M-IgG-DAPI.tif]
