# Supplementary material for: ZSWIM4 regulates embryonic patterning and BMP signaling by promoting nuclear Smad1 degradation
Source: EMBO Rep. 2024 Jan 2;25(2):14. doi: 10.1038/s44319-023-00046-w (PMC10897318; doi:10.1038/s44319-023-00046-w)
Supplement: Supplementary file 11 — Expanded View Figures [file 44319_2023_46_MOESM11_ESM.pdf]

Expanded View Figures

Figure EV1. Characterization of *Xenopus zswim4*.

(**A**, **B**) Zswim4 is highly conservative among different species. Sequence alignment (**A**) and sequence identities (**B**) among *X. laevis*, *X. tropicalis*, mouse, and human ZSWIM4. (**C–L**) Spatial expression pattern of *zswim4* in *Xenopus* embryos revealed by whole mount in situ hybridization. (**C**, **D**) animal pole view; (**E**) dorsal vegetal view; (**F**) lateral view with dorsal towards right; (**G**, **H**) dorsal view; (**I**, **J**) lateral view; (**K**, **L**) Transverse section of a stage 28 embryo at the levels illustrated by black lines in (**I**). nt neural tube, e eye, fg foregut, nc notochord. Scale bar = 250 μm. (**M**) Immunofluorescence staining of *X. laevis* embryos at stage 10 using anti-Rabbit IgG or anti-Zswim4 antibody, with dorsal side towards right. Scale bar = 200 μm. Source data are available online for this figure.

A

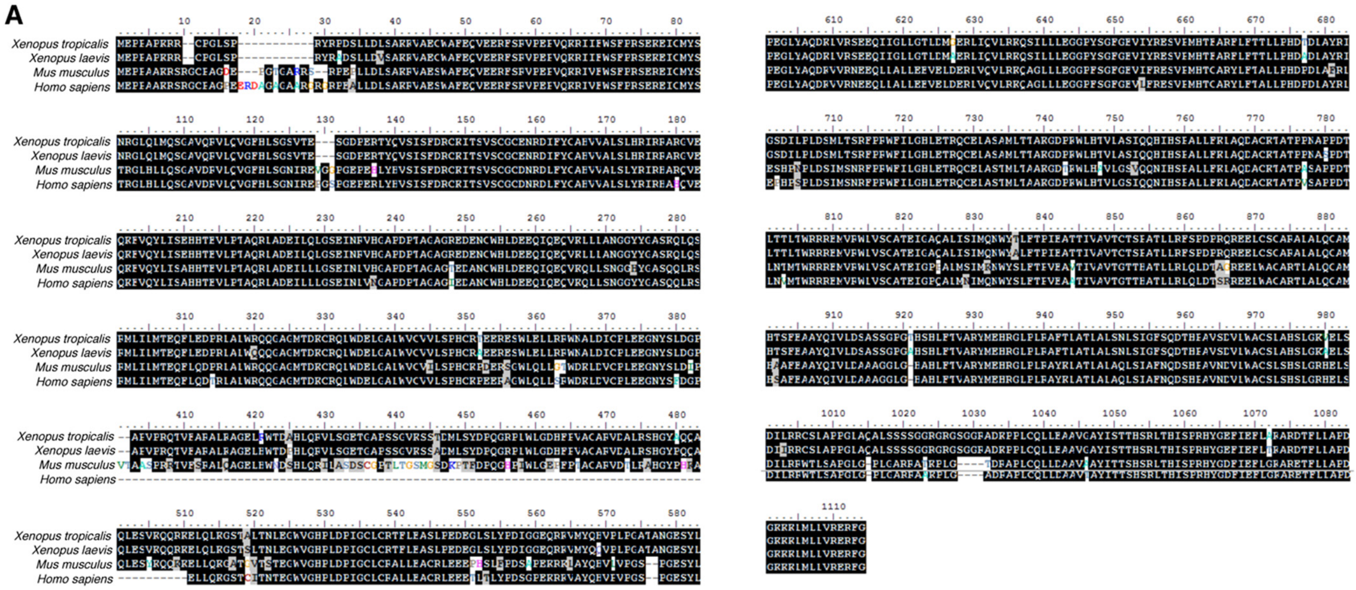

B

Species	Identity
Xenupus laevis	---
Xenopus tropicalis	98.1%
Mus musculus	74.3%
Homo sapiens	69.9%

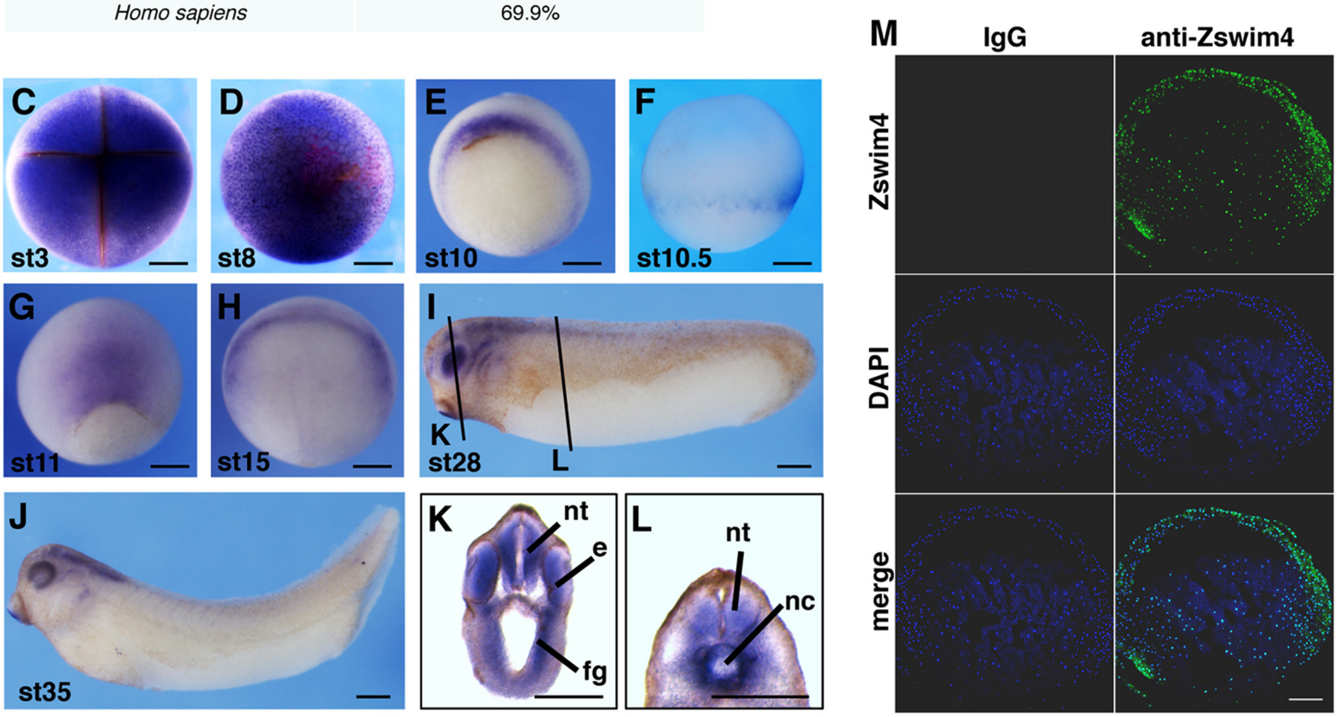

 © The Author(s)

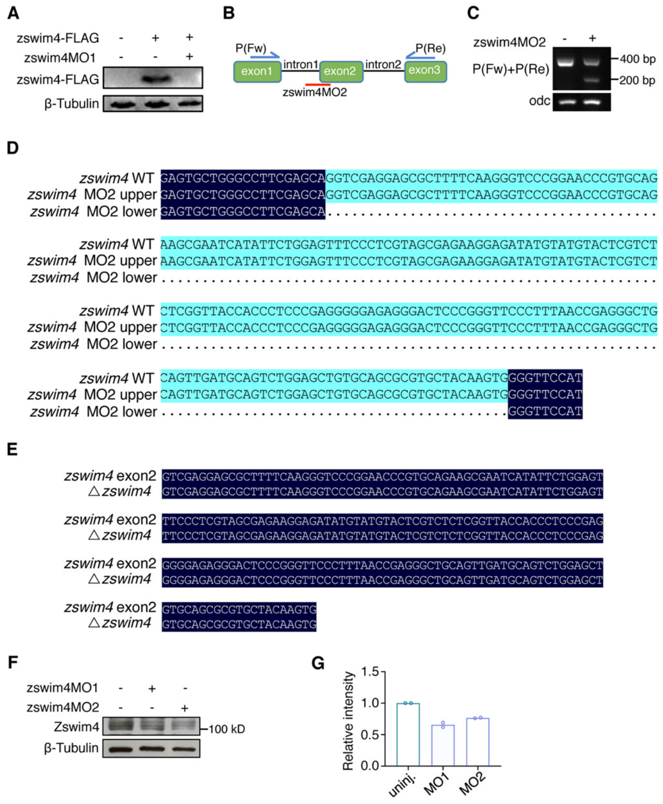

Figure EV2. Validation of zswim4MO1 and zswim4MO2.

(**A**) Western blot analysis of *zswim4-FLAG* protein in *X. tropicalis* embryos injected with *zswim4-FLAG* mRNA (60 pg/embryo) and zswim4MO1 (6 ng/embryo). β-Tubulin was used as a loading control. $n = 2$. One representative blot is shown. (**B–E**) Schematic diagram showing the zswim4MO2 binding site and the primer pair used to amplify the exon2 (**B**). RT-PCR was performed using the primer pair covering the *zswim4* exon2, and two bands were amplified from the *X. tropicalis* embryos injected with zswim4MO2 (**C**). The two bands were recovered for Sanger sequencing. The sequencing results were aligned with wild-type *zswim4* (**D**). The lost sequence in the lower band has 100% identity with *zswim4* exon2 (**E**). For western blot in (**A**) and RT-PCR in (**C**), $n = 2$. One representative blot is shown. (**F**, **G**) Western blot analysis of endogenous Zswim4 protein in *X. tropicalis* embryos injected with 6 ng of zswim4MO1 or zswim4MO2. β-Tubulin was used as a loading control. Quantification of Zswim4 bands is shown in (**G**). $n = 2$. Data information: *n* indicates biological replicates. Source data are available online for this figure.

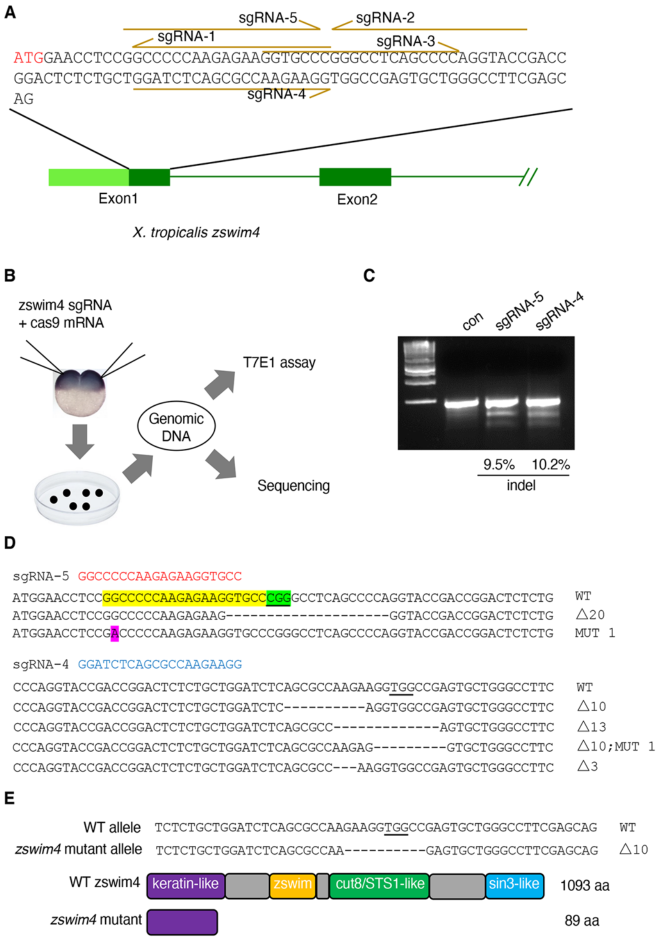

Figure EV3. Generation of the *zswim4* mutant frog line using CRISPR/Cas9.

(**A**) Five sgRNAs were designed to target *X. tropicalis zswim4* exon1. The start codon is marked in red. (**B**) Schematic diagram indicating the generation of *zswim4* mutant embryos using CRISPR/Cas9. (**C**) T7E1 assay was performed using genomic DNA extracts from the injected or uninjected embryos. $n = 2$. One representative blot is shown. (**D**) Sanger sequencing results confirmed the mutations in the *zswim4* CRISPR target region. (**E**) The *zswim4* mutant allele with a 10-bp deletion and the putative peptide encoded by the *zswim4* Δ10 mutant. Data information: *n* indicates biological replicates. Source data are available online for this figure.

 © The Author(s)

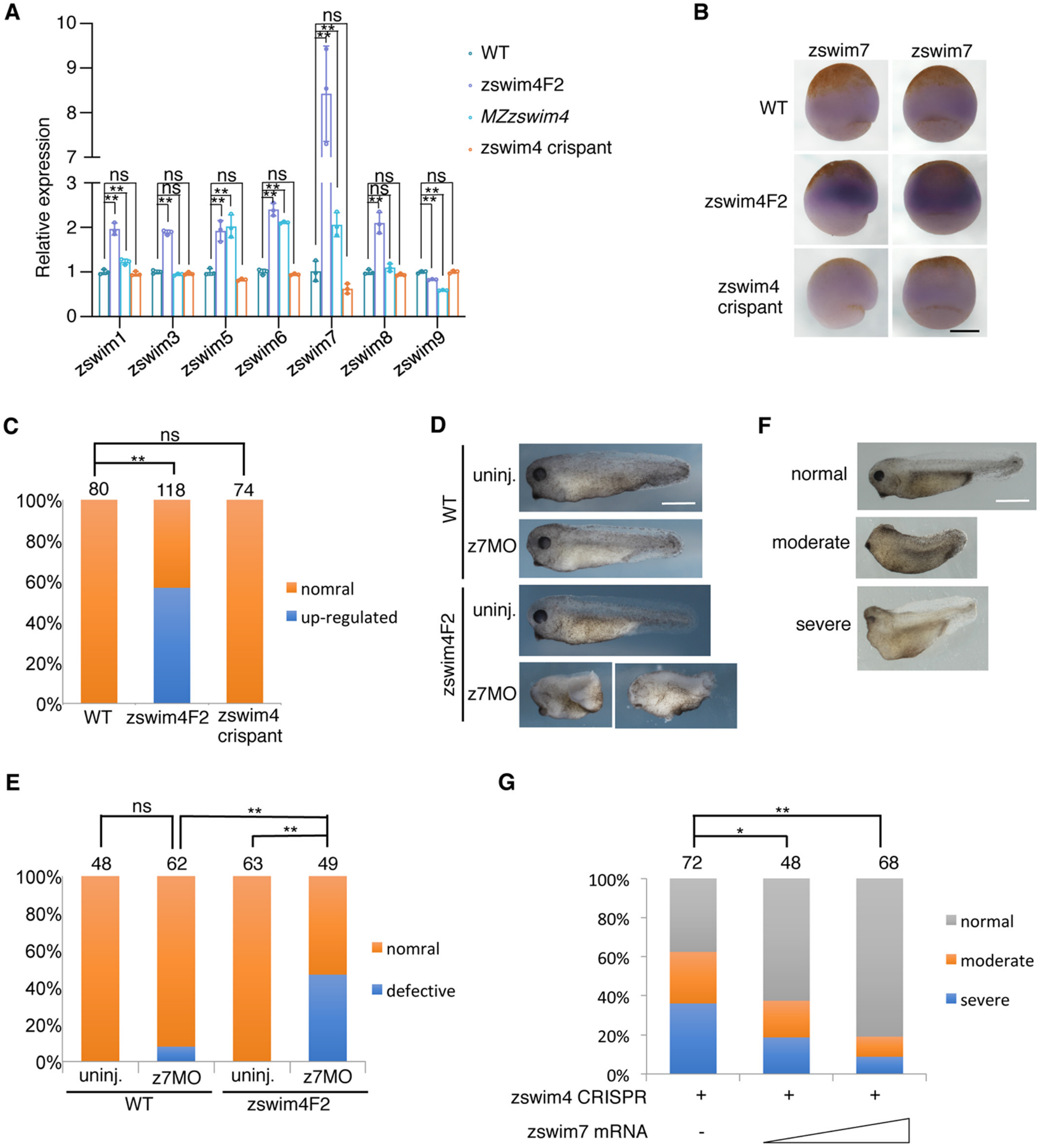
A
Relative expression
WT
zswim4F2
MZzswim4
zswim4 crispant
zswim1
zswim3
zswim5
zswim6
zswim7
zswim8
zswim9
B
zswim7
zswim7
WT
zswim4F2
zswim4 crispant
C
nomral
up-regulated
WT
zswim4F2
zswim4 crispant
D
WT
zswim4F2
uninj.
z7MO
F
normal
moderate
severe
E
nomral
defective
uninj.
z7MO
G
normal
moderate
severe
zswim4 CRISPR
zswim7 mRNA

Figure EV4. Up-regulated *zswim7* contributes to the genetic compensation in *zswim4* mutant frog lines.

(**A**) Genetic compensation was observed in *zswim4F2* embryos or *MZzswim4*$^{-/-}$ embryos. After crossing two *zswim4*$^{+/-}$ or two *zswim4*$^{-/-}$, the offsprings of stage 10 were collected for RNA extraction. The F0 embryos injected with Cas9 protein and four sgRNAs were also collected. Quantitative RT-PCR was performed to determine the gene expression of the indicated *zswim* family members. *odc* was used as the internal standard. $n = 3$. (**B**, **C**) WMISH showing the expression of *zswim7* in *X. tropicalis* embryos, *zswim4F2* embryos or zswim4 crispants (injected with 200 pg sgRNA plus 0.5 ng Cas9 protein/embryo). Embryos in the left column, lateral view with the dorsal side towards the right. Embryos in the right column, dorsal view. Scale bar = 1000 µm. The ratio of embryos with up-regulated expression of *zswim7* is shown in (**C**). The numbers on the top indicate the total number of embryos. $n = 3$. (**D**, **E**) *zswim4F2* embryos were injected with 6 ng of morpholino targeting *zswim7* (z7MO) at the one- or two-cell stage, and the phenotype was examined at the later tail-bud stage (**D**) Scale bar = 500 µm. The ratio of defective embryos is shown in (**E**). The numbers on the top indicate the total number of embryos. $n = 3$. (**F**, **G**) *X. tropicalis* embryos were injected with *zswim4* CRISPR/Cas9 (200 pg sgRNA plus 0.5 ng Cas9 protein/embryo) or increasing doses of *zswim7* mRNA (50 pg/embryo, 100 pg/embryos). Scale bar = 500 µm. The phenotype was examined at later tail-bud stage. The numbers on the top indicate the total number of embryos. $n = 3$. Data information: *n* indicates biological replicates. Error bars show mean ± standard deviation (SD). Statistical analysis was performed using an unpaired Student's *t* test for (**A**) and chi-squared test for (**C**, **E**, **G**). $^*p < 0.05$, $^{**}p < 0.01$, and ns indicates "not significant" ($p > 0.05$). Source data are available online for this figure.

 © The Author(s)

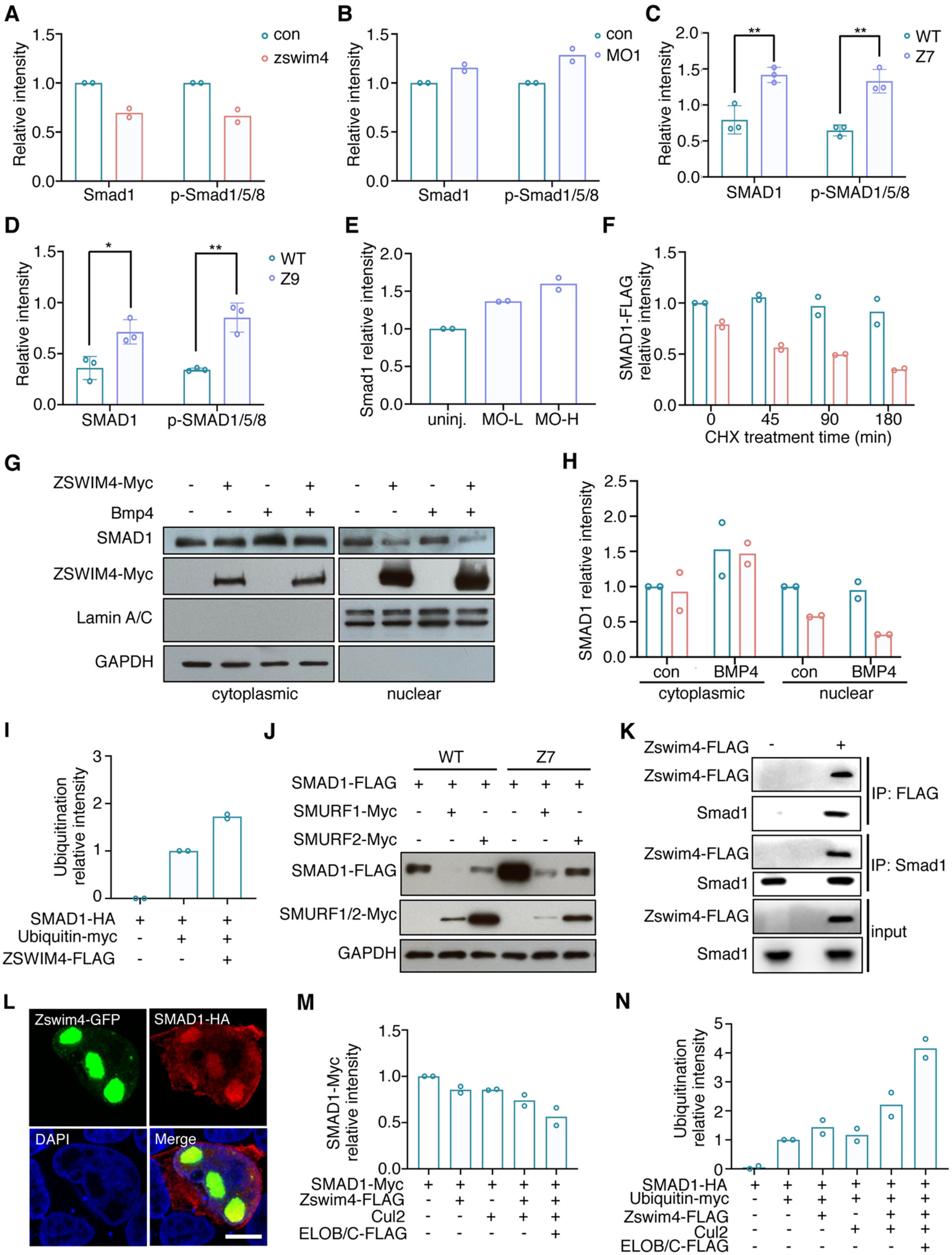
A
Relative intensity
con
zswim4
Smad1
p-Smad1/5/8
B
Relative intensity
con
MO1
Smad1
p-Smad1/5/8
C
Relative intensity
WT
Z7
SMAD1
p-SMAD1/5/8
D
Relative intensity
WT
Z9
SMAD1
p-SMAD1/5/8
E
Smad1 relative intensity
uninj.
MO-L
MO-H
F
SMAD1-FLAG relative intensity
CHX treatment time (min)
G
ZSWIM4-Myc
Bmp4
SMAD1
ZSWIM4-Myc
Lamin A/C
GAPDH
cytoplasmic
nuclear
H
SMAD1 relative intensity
con
BMP4
con
BMP4
cytoplasmic
nuclear
I
Ubiquitination relative intensity
SMAD1-HA
Ubiquitin-myc
ZSWIM4-FLAG
J
WT
Z7
SMAD1-FLAG
SMURF1-Myc
SMURF2-Myc
SMAD1-FLAG
SMURF1/2-Myc
GAPDH
K
Zswim4-FLAG
Zswim4-FLAG
Smad1
IP: FLAG
Zswim4-FLAG
Smad1
IP: Smad1
Zswim4-FLAG
Smad1
input
L
Zswim4-GFP
SMAD1-HA
DAPI
Merge
M
SMAD1-Myc relative intensity
SMAD1-Myc
Zswim4-FLAG
Cul2
ELOB/C-FLAG
N
Ubiquitination relative intensity
SMAD1-HA
Ubiquitin-myc
Zswim4-FLAG
Cul2
ELOB/C-FLAG

Figure EV5. ZSWIM4 reduces SMAD1 protein levels in the nucleus, but not in the cytosol.

(**A**, **B**) Quantifications of Smad1 and p-Smad1/5/8 bands in western blot shown in Fig. 3S (**A**) and Fig. 3T (**B**). $n = 2$. (**C**, **D**) Quantifications of SMAD1 and p-SMAD1/5/8 bands in western blot shown in Fig. 4K (**C**) and Fig. 4L (**D**). $n = 3$. (**E**) Quantifications of endogenous Smad1 bands in western blot shown in Fig. 5C. $n = 2$. (**F**) Quantifications of SMAD1-FLAG bands in western blot shown in Fig. 5D. $n = 2$. (**G**, **H**) Cell fractionation was performed on HEK293T cells transfected with *ZSWIM4-Myc* (500 ng/ml medium) and treated with BMP4 (100 ng/μl). The cytosolic and nuclear levels of SMAD1 were assayed by western blot. GAPDH and Lamin A/C were used as the loading control for the cytosolic and nuclear proteins, respectively. Quantification of SMAD1 bands is shown in (**H**). $n = 2$. (**I**) Quantifications of SMAD1-HA ubiquitination bands in western blot shown in Fig. 5F. $n = 2$. (**J**) *SMAD1-FLAG* (400 ng/ml medium) was co-transfected with *SMURF1-Myc* (200 ng/ml medium) or *SMURF2-Myc* (200 ng/ml medium) into the *ZSWIM4* mutant cell line, Z7, or wild-type HEK293T cells. The protein level of SMAD1-FLAG was examined by western blot. $n = 2$. One representative blot is shown. (**K**) Co-IP to detect the interaction between Zswim4-FLAG and endogenous Smad1 in *X. laevis* embryos. $n = 2$. One representative blot is shown. (**L**) Confocal imaging illustrates the co-localization of Zswim4-GFP and SMAD1-HA in the nucleus of HeLa cells treated with BMP2 (100 ng/μl) for 5 h. Scale bar = 10 μm. $n = 2$. One representative blot is shown. (**M**) Quantifications of SMAD1-Myc bands in western blot shown in Fig. 7A. $n = 2$. (**N**) Quantifications of SMAD1-HA ubiquitination bands in western blot shown in Fig. 7B. $n = 2$. Data information: *n* indicates biological replicates. Error bars show mean ± standard deviation (SD). Statistical analysis was performed using an unpaired Student's *t* test for (**C**, **D**). *$p < 0.05$, **$p < 0.01$. Source data are available online for this figure.

 © The Author(s)
